# Al(ii) transfer harnessing a well-defined cadmium precursor†

**DOI:** 10.1039/d6sc00437g

**Published:** 2026-02-23

**Authors:** Dominic Herle, Frerik Wurm, Crispin Lichtenberg, Fabian Dankert

**Affiliations:** a Institute of Chemistry, University of Kassel Heinrich-Plett Str. 40 34132 Kassel Germany Fabian.Dankert@uni-kassel.de; b Department of Chemistry, Philipps-University Marburg Hans-Meerwein Str. 4 35032 Marburg Germany crispin.lichtenberg@chemie.uni-marburg.de; c mar.quest | Marburg Center for Quantum Materials and Sustainable Technologies 35032 Marburg Germany

## Abstract

Low-valent aluminum chemistry continues to expand the boundaries of main-group reactivity, yet the selective generation and transfer of Al(ii) fragments remain underexplored. Controlled Al(ii) transfer may establish a basis for selective substrate alumination and subsequent functionalization. More broadly, findings in the field offer conceptual guidance for the development of synthetically and potentially catalytically relevant main-group platforms. Here, we now show that heterometals can act as structural templates that tame and direct Al(ii) reactivity within covalent Al/Cd frameworks. The trimetallic compound [({N(TMS)_2_})(Cp*)Al]_2_Cd (1^tri^; Cp* = C_5_Me_5_) proved as an excellent candidate, selectively transferring aluminum complex fragments through cadmium extrusion. This reactivity was verified through reactions with free radicals, dichalcogenides, and benzophenone (-derivatives), the latter representing spin-trapped versions of the fleeting Al(ii) radical [({N(TMS)_2_})(Cp*)Al]˙. In contrast, [{N(TMS)_2_}(Cp*)Al–Cd{N(TMS)_2_}] (1^bi^) retains cadmium and instead promotes cadmium transfer, *i.e.* to form chalcogenophenolates, underscoring its nuclearity-dependent reactivity. Corroborating these experimental observations, DFT studies provide insight into the formation pathways, electronic structure, and stability of the resulting compounds.

## Abstract 2

A cadmium template enables controlled Al(ii) release from a trimetallic Al/Cd platform while also supporting Cd-centered transfer when nuclearity shifts.

## Graphical abstract

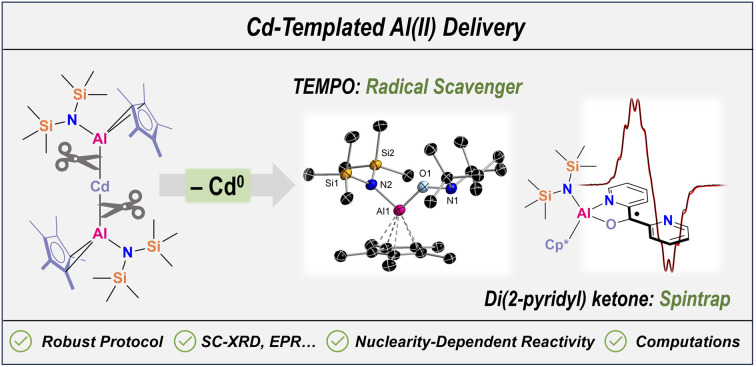

## Introduction

**Scheme 1:**
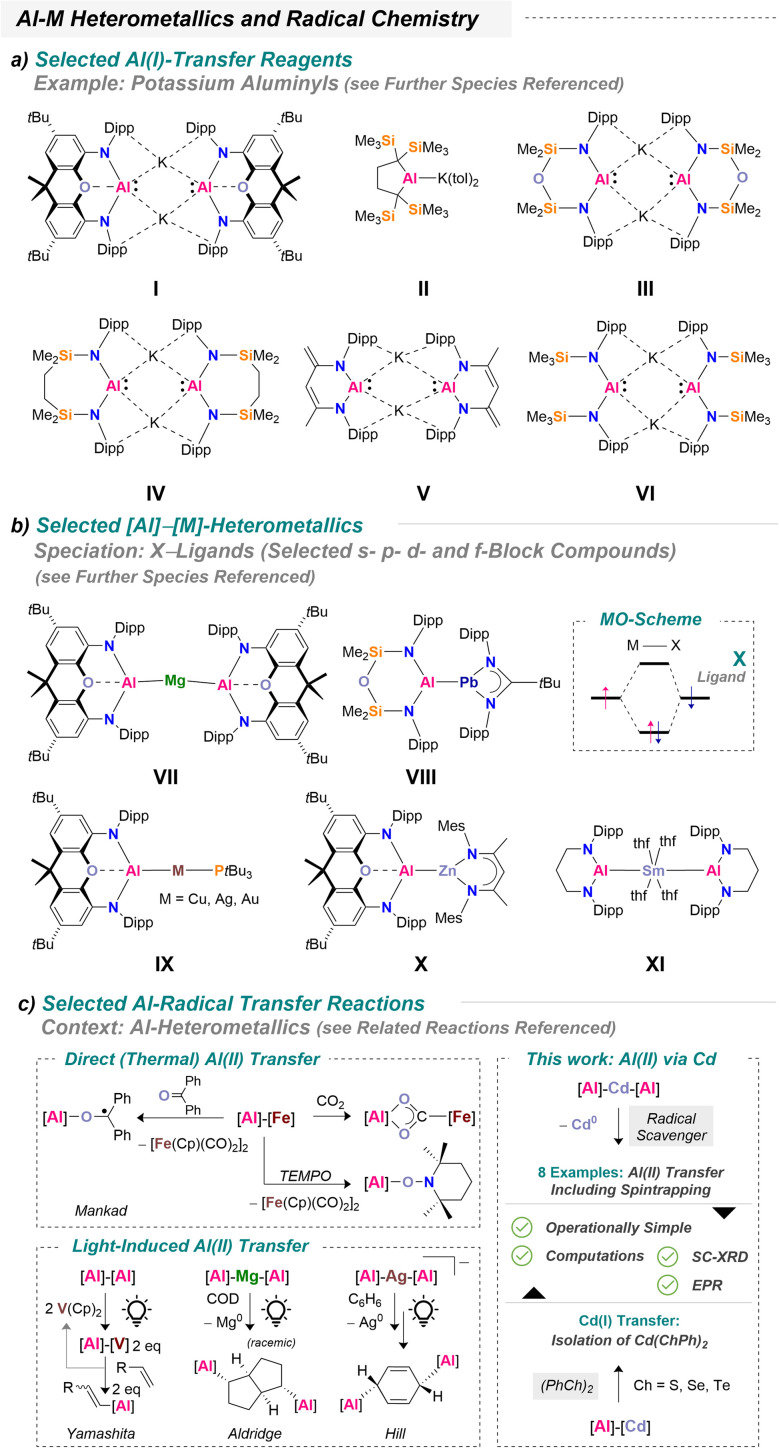
Selected [R_2_Al(i)]^−^ transfer reagents (a: top), selected accessible heterometallic compounds (b: middle) and recent contributions to radical chemistry including an outline of this study (c: bottom).

Aluminum chemistry has long been dominated by the +III oxidation state, whose classical Lewis acidic behavior is well established – *i.e.* in fundamental molecular chemistry to drive hydroelementation reactions or engage in FLP (= frustrated Lewis pair) chemistry.^1,2^ However, low-valent aluminum species are continuing to emerge as a rich source of unusual electronic structures and unconventional reactivity. Within modern main-group chemistry, aluminylenes (*i.e.*, [R–Al]^0^), metal aluminyls (*i.e.* M^+^[R_2_Al]^−^, also referred to as “alumanyls”), and neutral dialumanes (-enes) (*i.e.*, {[R_2_Al]_2_}^0^ and {[R–Al]_2_}^0^) have become central players in contemporary research.^3–5^ Their inherent capacity to serve as electron reservoirs for small-molecule fragments has strongly shaped current perspectives on bond-activation pathways. Consequently, the utilization of low-valent aluminum in reduction chemistry now occupies a substantial and steadily expanding chemical space. In recent years, an important subfield, namely heterometallic aluminum compounds, has rapidly gained momentum.^6–9^ Among these, alkali-metal aluminyls are widely recognized as [R_2_Al(i)]^−^ transfer reagents. The landmark K_2_[Al(NON)]_2_ system (I) reported by Aldridge and Goicoechea (NON = 4,5-bis(2,6-diisopropylanilido)-2,7-di-*tert*-butyl-9,9-dimethylxanthene) stands out as one of the first potent examples of an aluminum-centered metallo-ligand.^10^ Since then, the groups of Yamashita (II),^11–13^ Coles (III),^14–16^ Hill (IV),^17,18^ Harder (V)^19^ or Hicks (VI)^20^ have steadily broadened the structural diversity and reactivity landscape of these systems (Scheme 1). These negatively charged Al-based metallo-ligands typically exhibit reactivity at the ambiphilic aluminum center, most prominently, among few other reactivity patterns,^7^ through single-center oxidative addition. Beyond this intrinsic behavior, however, they also provide access to a broader and highly attractive field of research: the formation of (unsupported) heterometallic partnerships. Such architectures are most commonly achieved through salt-metathesis pathways, which have become a defining strategy for generating novel heterometallic aluminum frameworks. A natural conceptual entry point into heterometallic aluminum chemistry is the fundamental molecular-orbital picture in which the aluminum fragment engages as an “X”-type ligand, operating within an electron-shared bonding regime (Scheme 1b). This scenario is prototypically realized in systems where significant covalency develops between aluminum and a second metal center. Under these conditions, a wide spectrum of metal partners were bound, spanning the s-block,^10,12,15,18,20–28^ p-block,^29–32^ transition metals—including early,^11,33,34^ mid-,^35–38^ and late^35–54^—as well as the f-block.^55^ Some representative examples are shown in Scheme 1b: VII–XI.^25,31,40,44,47,55^ An attractive feature of these systems is their ability to undergo synergistic bond activation, with the heterometallic partner often acting as a structure-directing unit in small molecule activation pathways concomitant with aluminum functioning Lewis-acidic.^7,9^ From a fundamental viewpoint, the heterometal may also serve as a template for constructing follow-up organometallic architectures.^41^

Lately, Mankad's group has significantly expanded the accessible reactivity landscape of aluminum-based heterometallics toward radical chemistry under mild conditions by harnessing a [Al]–[Fe] platform.^56^ Typically, Al(ii) radicals can arise *via* complementary pathways. One common approach is ligand-mediated, where redox-active ligands in the aluminum coordination sphere mimic Al(ii) reactivity through low-lying acceptor orbitals.^57–59^ Another route involves alkali-metal reduction of suitable precursors, as demonstrated recently by Sekiguchi^60^ or Andrada and co-workers,^61^ which generates (fleeting) Al(ii) radicals. In contrast, the generation of Al(ii) *via* heterometallic frameworks, through direct electronic communication between aluminum and a transition or main-group metal, is far less explored. Notable examples hitherto include Yamashita's formal Al(ii) transfer at vanadium,^34^ Aldridge's Al(ii) transfer using Mg[Al(NON)]_2_,^25^ and Hill's Birch-type reduction of benzene with a silver bis-aluminyl metallate, where *in situ* EPR spectroscopy supports the involvement of Al(ii).^43^ While photochemical activation is required for all examples, these studies collectively illustrate the potential of heterometallic frameworks as templates for accessing authentic Al(ii) radicals. Despite the interest in Al(ii), its migration or transfer between metal centers remains largely unexplored. Gaining insight into how an Al(ii) fragment can be mobilized, stabilized, and transferred, particularly within heterometallic architectures, promises to reveal novel reactivity patterns and bonding motifs. From a synthetic perspective, harnessing Al(ii) intermediates may as well allow selective substrate alumination and subsequent functionalization, providing entry points to substrate upgrading and diversification strategies.

Recently, we reported bi- (1^bi^ = [{N(TMS)_2_}(Cp*)Al–Cd{N(TMS)_2_}]) and trimetallic (1^tri^ = [({N(TMS)_2_})(Cp*)Al]_2_Cd) cadmium aluminyls, accessed through stoichiometric aluminylene insertion into Cd{N(TMS)_2_}_2_.^53^ These species exhibit a high degree of covalency between Al and Cd, are notably more stable than their zinc congeners,^49,62^ and have been shown to participate in incipient reactivity studies such as Al(i) transmetalation and heterocumulene insertion. While several bonding descriptions and oxidation state assignments are conceivable for these compounds, their overall covalent character suggested that they could serve as ideal candidates for Al(ii) transfer reactions. Building on this concept, we demonstrate that Al/Cd frameworks can act as effective surrogates for Al(ii) under mild, thermal conditions, enabling a range of Al(ii) transfer processes, including spin-trap experiments. Remarkably, all transformations proceed at ambient temperature without the need for photochemical activation.

## Results and discussion

### Al(ii) transfer employing a trimetallic cadmium aluminyl

**Scheme 2:**
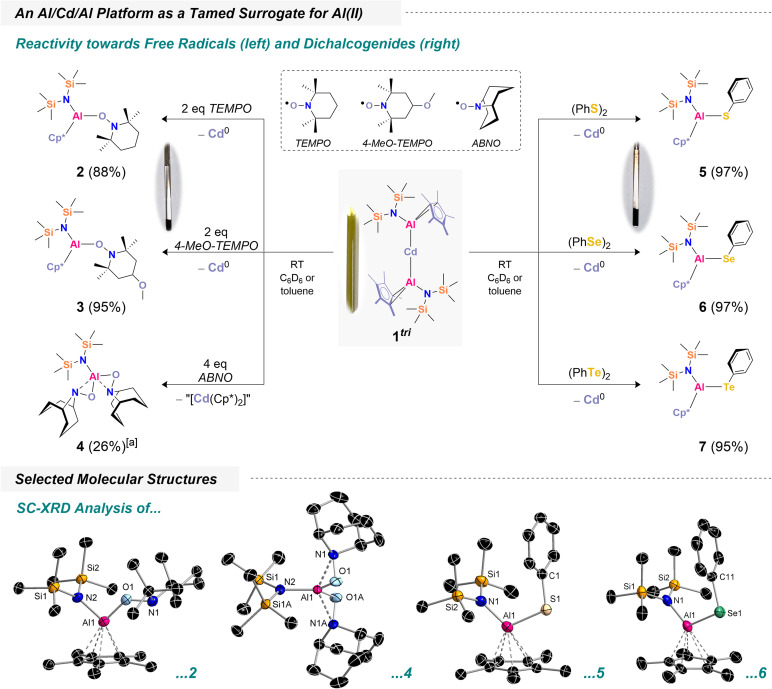
Top: reactivity of a trimetallic cadmium aluminyl towards radical scavengers and selected illustrations of cadmium precipitation (*N*-oxyl: left, dichalcogenide: right). The indicated yields are isolated yields ([a] = isolated yield of a TMS solvate (see SI)). Bottom: selected molecular structures in the crystal (for 3 and 7, see SI). Thermal ellipsoids are drawn at the 50% probability level. Selected bond lengths [Å] and angles [°] in 2: Al1–O1 1.740(5), Al1–N2 1.836(4), N1–O1 1.445(6), N2–Si1 1.737(4), N2–Si2 1.737(3), N2–Al1–O1 99.4(1), N1–O1–Al1 137.4(2); 4 (*): Al1–N2 1.789(9), Al1⋯N1 1.977(8), Al1–O1 1.755(9), (*) metrics of one independent molecule in the asymmetric unit – symmetry generated atoms created through 1 − *x*, *y*, 2 − *z*; 5: Al1–N1 1.817(2), Al1–S1 2.222(1), N1–Si1 1.735(2), N1–Si2 1.733(2), C1–S1 1.777(3), N1–Al1–S1 111.99(8), Si1–N1–Si2 122.5(1), Al1–S1–C1 108.90(9); 6 (**): Al1–N1 1.833(5), Al1–Se1 2.360(2), N1–Si1 1.732(6), N1–Si2 1.733(6), C11–Se1 1.930(6), N1–Al1–Se1 112.0(2), Si1–N1–Si2 123.4(3), Al1–Se1–C11 105.8(2), (**) metrics of one independent molecule in the asymmetric unit.

To explore Al(ii) transfer, we began by reacting 1^tri^ with two equivalents of TEMPO (TEMPO = 2,2,6,6-tetramethylpiperidine-1-oxyl). Upon addition of TEMPO to a yellow solution of 1^tri^, the mixture rapidly turned colorless, accompanied by the formation of large amounts of black precipitates (Scheme 2). ^1^H NMR spectroscopy revealed new resonances at 2.02 ppm and 0.31 ppm, corresponding to Cp* and HMDS moieties, consistent with the selective formation of a new species. Additional signals corresponding to –CH_2_ and CH_3_ groups were observed, and integration indicated a 1 : 1 : 1 ratio of {N(TMS)_2_}, Cp* and the associated TEMPO fragment (Fig. S4). Based on these observations, we proposed the formation of [({N(TMS)_2_})(Cp*)Al(TEMPO)] (2), with concomitant precipitation of elemental cadmium from the solution (see SI). Isolation of 2 was achieved by workup and evaporation from *n*-pentane, affording crystals suitable for X-ray diffraction (XRD) analysis. The structure confirms TEMPO coordination *via* its O atom, with an expectedly short Al–O bond of 1.740(5) Å.

**Scheme 3:**
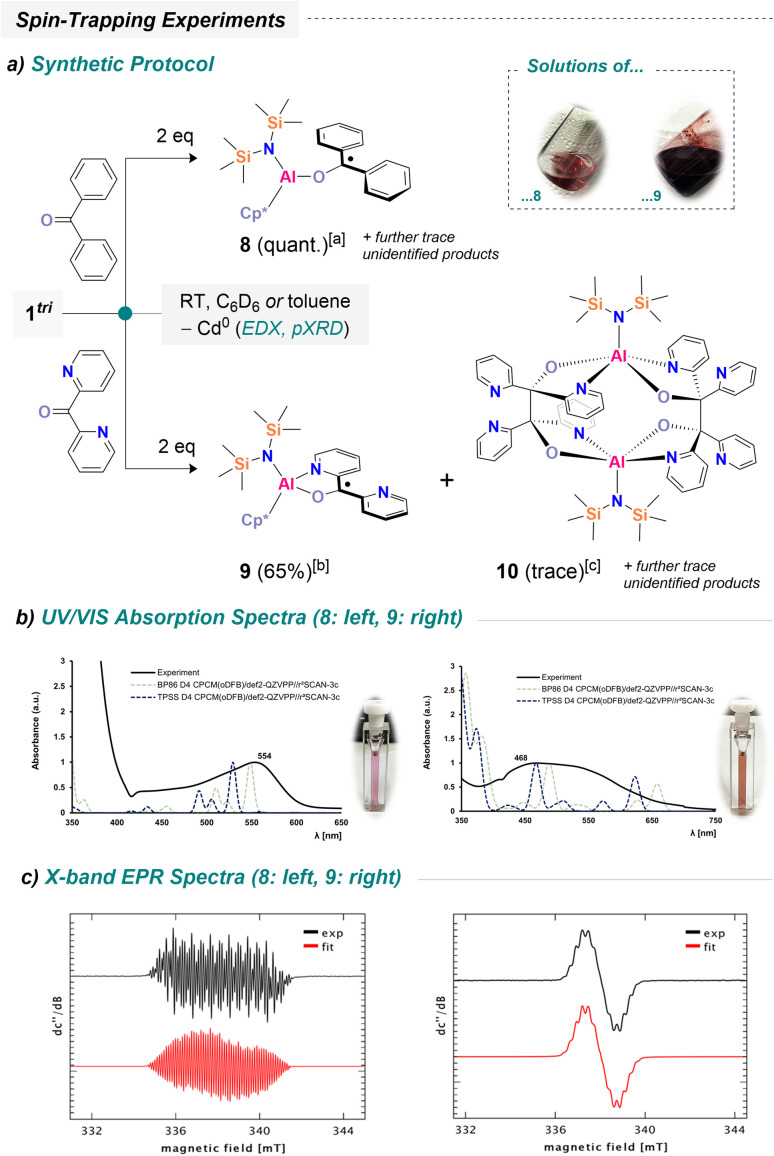
Reactivity of 1^tri^ towards benzophenone derivatives and selected characterization. See (a) synthetic access to neutral radical species with [a] = conversion, [b] = isolated crude yield containing trace amounts of 10, [c] = few single crystals found upon microscopic analysis; (b) UV-vis spectrum of 8 and 9 in *o*DFB including selected simulated spectra derived from TD-DFT at given computational approaches; (c) X-band EPR spectra of 8 and 9: please see Fig. S60–S62 for a detailed description of parameters on the EPR measurements.

Remarkably, the reaction proceeds with high selectivity: no side products were detected, the conversion of 1^tri^ is quantitative, and 2 was isolated in 88% yield as a crystalline solid. Encouraged by this result, we investigated the scope of 1^tri^ as a mild and controlled Al(ii) transfer reagent. As a next example, we explored its reactivity with 4-MeO-TEMPO (MeO = –OCH_3_). Addition of 4-MeO-TEMPO to a yellow solution of 1^tri^ led to observations analogous to those described for TEMPO. ^1^H NMR spectroscopy showed resonances consistent with selective formation of a new species, including a signal at 3.17 ppm corresponding to the –OC*H*_3_ group, confirming the formation of [({N(TMS)_2_})(Cp*)Al(4-MeO-TEMPO)] (3) (Fig. S7). After workup, 3 was isolated in 95% yield, and its molecular structure was determined (Fig. S65). Structural metrics are broadly comparable to those of 2. To further explore the scope of *N*-oxyl derivatives, 1^tri^ was also reacted with ABNO (ABNO = 9-azabicyclo[3.3.1]nonan *N*-oxyl) following the same protocol. Initially, two equivalents of ABNO did not lead to complete consumption of 1^tri^ (see Fig. S55). ^1^H NMR analysis revealed a new main species featuring an [{N(TMS)_2_}]^−^ ligand resonance at 0.46 ppm, formed in a 1 : 1 ratio with unreacted 1^tri^. We therefore hypothesized that full conversion would require four equivalents of ABNO. Upon addition of the additional two equivalents, 1^tri^ was fully consumed. Workup and careful crystallization of the resulting material afforded [({N(TMS)_2_})Al(ABNO)_2_] (4). Through meticulous washing and recrystallization from tetramethylsilane solutions, pure crystalline 4·Si(CH_3_)_4_ was obtained in 26% yield. The molecular structure of unsolvated 4, which was crystallized from *n*-pentane, confirms the formal addition of two radicals to the aluminum center with the Cp* ligands being fully replaced. Notably, 4 exhibits an unusual coordination mode: both the N- and O-atoms of the ABNO fragment coordinate to aluminum. This binding motif is reminiscent of [{C_4_H_3_N(2-CH_2_N*t*Bu)}Al(ON = CMe_2_)]_2_, where an oximate ligand shows a similar coordination mode.^63^ Interestingly, a notable difference was observed during the reaction of 1^tri^ with ABNO: only minimal black precipitate formed. This suggests that the fragmentation of 1^tri^ may follow a distinct pathway here, likely involving a twofold one-electron transfer from each Al–Cd bond to four equivalents of ABNO, accompanied by formation of Cd(ii)- and Cp*-containing side products. Attempts to identify these side products were unsuccessful, and the species consistently appeared as smeary greases. Beyond *N*-oxyl derivatives, we investigated dichalcogenides, (PhCh)_2_ (Ch = S, Se, Te), as potential radical scavengers owing to their relatively low bond dissociation energies. In all cases, the 1 : 1 reaction of 1^tri^ with (PhCh)_2_ proceeded within seconds, accompanied by precipitation of elemental cadmium. For example, in the sulfur case, ^1^H NMR spectroscopy revealed diagnostic Cp* and HMDS resonances at 1.97 and 0.08 ppm, respectively, along with appropriate aromatic signals (see Fig. S14). Integration indicated a 1 : 1 : 1 ratio of Ph-, Cp*-, and [{N(TMS)_2_}]-groups, consistent with selective formation of [({N(TMS)_2_})(Cp*)Al(S–Ph)] (5). X-ray crystallography confirmed that the structural motif mirrors the one observed for TEMPO addition. Similarly, reactions with the Se and Te congeners afforded [({N(TMS)_2_})(Cp*)Al(Se–Ph)] (6) and [({N(TMS)_2_})(Cp*)Al(Te–Ph)] (7). 7 is somewhat exceptional, displaying short Te⋯Te contacts in the solid state, resulting in a dimeric arrangement, [({N(TMS)_2_})(Cp*)Al(Te–Ph)]_2_ (see Fig. S69). Such contacts are known to influence crystal packing, as recently shown for other tellurium organometallics.^64^ We briefly note that we also tried to convert 1^tri^ with a more complex trityl radical (*i.e. via* “Gomberg's dimer”),^65^ yet unsuccessful allegedly due to steric constraints (see Fig. S56). Collectively, the transformations furnishing compounds 2–7 represent authentic Al(ii) transfer events from the Al/Cd framework. To further substantiate the plausibility of Al(ii) transfer, we explored spin-trapping of the plausible radical [({N(TMS)_2_})(Cp*)Al]˙. For this purpose, 1^tri^ was first reacted with two equivalents of benzophenone. The reaction rapidly produced black precipitates, analogous to the previous experiments. Using only one equivalent of benzophenone was insufficient for full conversion, whereas addition of a second equivalent drove the reaction to completion (Fig. S58). Filtration of the precipitate afforded a magenta-colored solution. UV/vis spectroscopy revealed absorption in the 450–600 nm region, with a distinct maximum at 554 nm (Scheme 3b) which almost perfectly matches related aluminum-based ketyl radicals: the absorption maximum of [(NON)Al(OC–Ph_2_)]˙, for instance, was found at 545 nm.^56^^1^H NMR spectroscopic analysis of a freshly prepared sample in C_6_D_6_ solution showed only trace amounts of diamagnetic species (Fig. S58: top), confirming near-quantitative conversion of 1^tri^ and supporting the formation of a radical product.

**Scheme 4:**
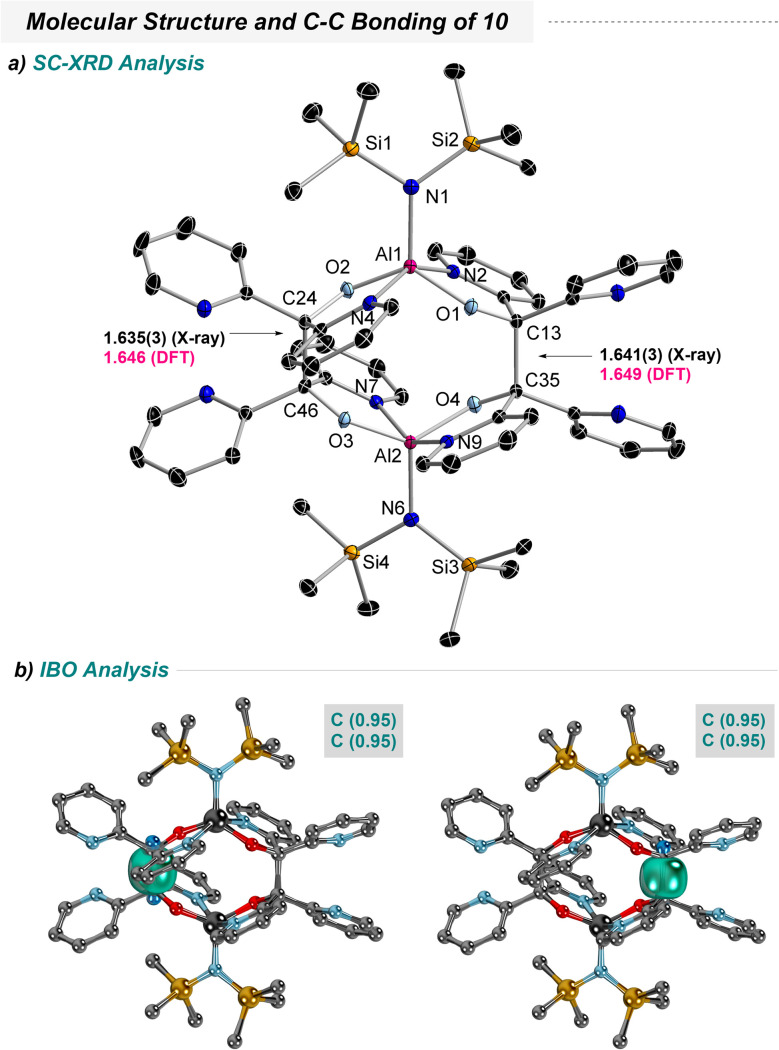
Molecular structure in the solid state as determined by SC-XRD of 10 in the crystal at 100 K with thermal ellipsoids representing the 50% probability level (a) including intrinsic bond orbitals with focus on C–C bonding at the r^2^SCAN-3c level of theory (b). Selected bond lengths [Å] and angles [°] in 10: Al1–N1 1.885(2), Al1–O1 1.786(1), Al1–N2 2.034(1), Al1–O2 1.783(1), Al1–N4 2.022(2), Al2–N6 1.885(2), Al2–N7 2.021(2), Al2–O3 1.787(1), Al2–N9 2.029(2), Al2–O4 1.789(1), C13–C35 1.641(3), C24–C46 1.635(3), Si1–N1–Si2 112.93(9), O1–Al1–O2 129.50(6), N2–Al1–N4 151.76(7), O1–C13–C35 107.3(1), O2–C24–C46 108.3(1), Si3–N6–Si4 113.61(9), O3–Al2–O4 131.77(6), N7–Al2–N9 151.38(7), O3–C46–C24 108.3(1), O4–C35–C13 106.8(1).

The most intuitive pathway involves replacement of cadmium by benzophenone, trapping the spin from the transient Al(ii) center and generating [({N(TMS)_2_})(Cp*)Al(O–CPh_2_)]˙ (8). Unfortunately, all attempts to isolate 8 from the magenta solution were unsuccessful to date. Prolonged storage led to slow decomposition, producing black precipitates and colorless residues (see SI). Remarkably, even at −30 °C under inert conditions, the compound decomposed slowly. To gain further insight, the DFT-optimized structure of 8, combined with time-dependent DFT (TD-DFT) calculations, was utilized to investigate the absorption properties of 8 in the UV/vis region. Indeed, the theoretically predicted spectrum reproduces the experimentally observed UV/vis spectrum reasonably well (Scheme 3b). We note, however, a strong influence of Hartree–Fock contributions (see Table S6: top). NTO (= natural transition orbital) analysis indicates that the dominant excitation corresponds to promotion of the singly occupied molecular orbital into a ligand-centered π* orbital (see Table S6: bottom). Encouraged by these results, 1^tri^ was also reacted with di(2-pyridyl) ketone. The pyridyl substituents were expected to provide additional stabilization through chelating N-coordination at aluminum. Upon addition of two equivalents of the ketone, large amounts of black precipitate formed, similar to previous reactions. Filtration of the resulting dark suspension yielded a deep red-to-purple solution, showing only trace amounts of diamagnetic impurities (see Fig. S59), suggesting formation of [({N(TMS)_2_})(Cp*)Al(OC–Py_2_)]˙ (9; Py = 2-pyridyl). Diluted solutions suitable for UV/vis spectroscopy appear amber-red, with the major absorption observed between 400–700 nm and a distinct maximum at 468 nm (Scheme 3b). Chelation by the pyridyl groups provides significant stabilization (*vide infra*), allowing 9 to be isolated as a dark purple powder in 65% (crude) yield, containing trace amounts of 10 and an additional unidentified species. The successful isolation of this material enabled further investigations such as FD mass spectrometry. The presence of 9 was unambiguously detected in the gas phase (*m*/*z* calcd 506.2598; found 506.2596; Fig. S29–S31, including high-resolution spectra). At this stage, all analytical data indicate the precipitation of elemental cadmium from the reaction solutions. To further substantiate this observation, energy-dispersive X-ray spectroscopy (EDX) as well as X-ray powder diffraction were performed on isolated precipitates. EDX analysis confirmed cadmium as the main component, with only trace amounts of aluminum and oxygen detected (Fig. S63; Table S1). The oxygen likely arises from sample preparation in air. Powder diffraction for which sample preparation was possible under inert conditions is consistent with cadmium as the sole component (Fig. S71). However, most eminently, the proposed structures of 8 and 9 have been investigated by X-band EPR spectroscopy. Both, 8 and 9 gave compound-specific couplings to different nuclei (Scheme 3c). For 8, the spectrum exhibits a reasonably resolved multiplet consistent with hyperfine couplings to ten ^1^H nuclei and one ^27^Al nucleus, indicating delocalization of the unpaired electron over the two phenyl groups of the ligand framework and (very) weak interaction with the aluminum center. It should be noted that these investigations indicate the inequivalence of the phenyl groups in 8 on the time scale of the EPR spectroscopic experiment, as previously observed for related species.^56^ For 9, the main resonance reveals coupling of the unpaired electron with two ^14^N nuclei and eight protons, consistent with spin delocalization over the two spectroscopically inequivalent pyridyl groups. In both cases, the experimental spectra are acceptably reproduced by assuming a single unpaired electron and the coupling constants noted in Fig. S60–S62. Corroborated by DFT calculations, the main contribution of spin density resides at the former carbonyl C atom (Fig. S79) yet is delocalized significantly across the aromatic groups of the ligand, with minor spin density at the aluminum center of 8. Through microscopic investigation of the material obtained during the isolation of 9, we could observe a few slight orange plates that had formed throughout work-up. To our delight, these were suitable for SC-XRD studies and revealed a peculiar decomposition product (Scheme 4a).

The structure refinement unveiled a dimeric structure, in which the Cp* ligands are (i) missing and (ii) are replaced by another equivalent of the former di(2-pyridyl) ketone (Scheme 4). As can further be seen here, the radical anions had undergone C–C coupling to form a dianionic chelate which holds together a dinuclear aluminum complex. As predicted from the spin density distributions in 8 and 9 C–C coupling occurs *via* the highest spin density location. The overall steric constraints of complex 10 force C–C bond elongation to 1.64 Å. Note that a standard C–C bond has a bond length of about 1.50–1.55 Å.^66^ Conclusively, the associated Wiberg bond index reveals a low value of 0.703 for each C–C bond, which may declare these bonds as weak. IBO analysis sheds further light on the nature of these bonds and reveals typical σ-bonding interactions (Scheme 4b). Both C atoms contribute equally according to IAO partial charge distributions of 0.95/0.95 in both C–C bonds of interest and are thus perfectly covalent.

### Influence of nuclearity

**Scheme 5:**
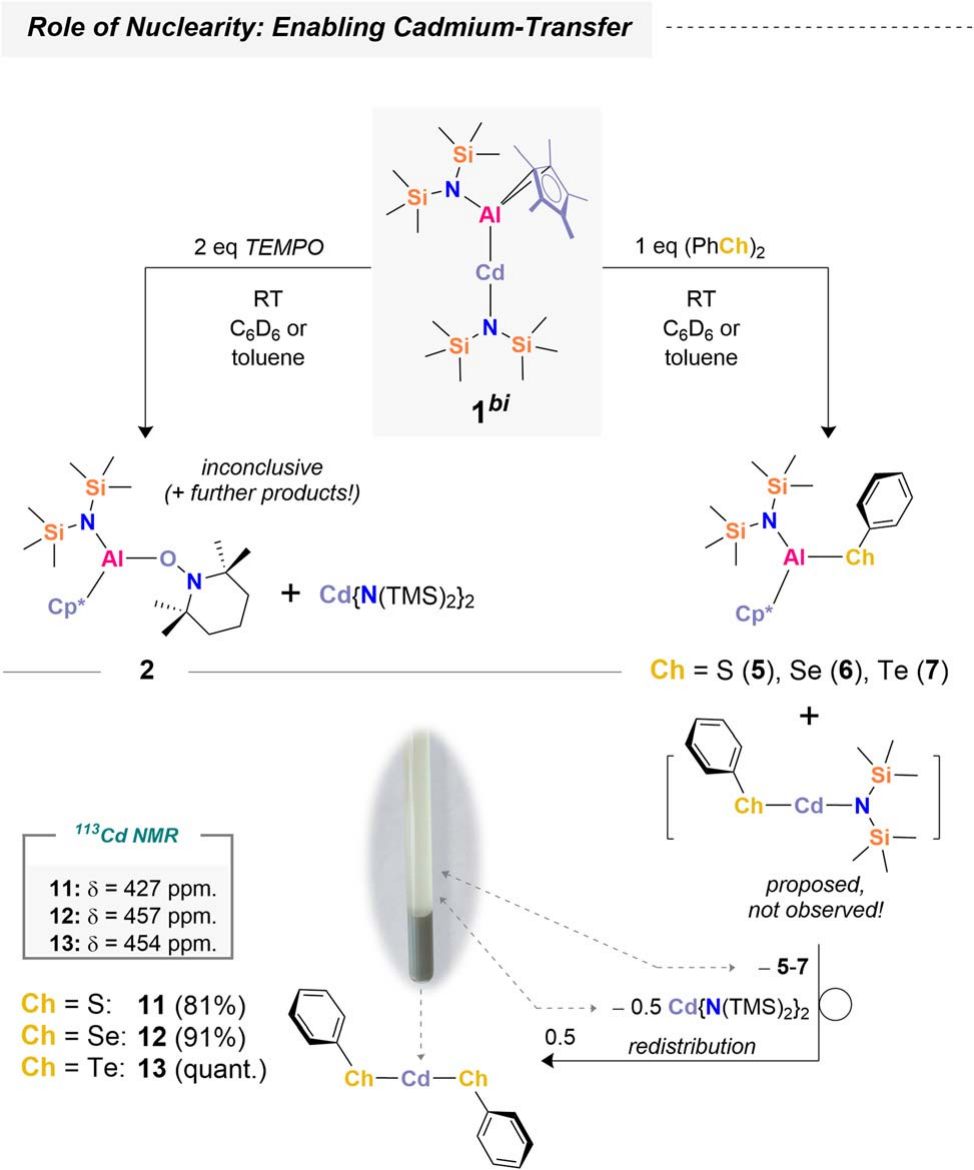
Reactivity of a bimetallic Al/Cd platform towards a free radical (top) and dichalcogenides (bottom) including an exemplary picture of precipitated compound 13 in C_6_D_6_. The ^113^Cd NMR chemical shifts were obtained from pyridine-*d*_5_ as the solvent. Depicted yields are isolated yields after workup (see SI).

Having demonstrated selective Al(ii) transfer *via*1^tri^, we next explored the bimetallic species 1^bi^ to probe the role of nuclearity and investigate Cd(i) transfer – that is, retaining cadmium in the presence of a radical scavenger (Scheme 5). 1^tri^ serves as an efficient reagent, selectively transferring aluminum complex fragments through cadmium extrusion, with convenient work-up by filtration leaving essentially only the desired products in solution (see SI). The bimetallic congener 1^bi^ was expected to show similar aluminum transfer chemistry, but cadmium behavior was anticipated to differ. The absence of a second aluminum center may enhance cadmium stabilization *via* the strongly coordinating [{N(TMS)_2_}]^−^ ligand,^67^ allowing cadmium to be maintained as Cd(i), paralleling the chemistry on the aluminum side. Reaction of 1^bi^ with TEMPO proceeded inconclusively. Although 2 and [Cd({N(TMS)_2_})_2_] were observed as products (Fig. S55), further cadmium-containing species could not be identified. Given the limited success with TEMPO, we turned to dichalcogenides ((PhCh)_2_; Ch = S, Se, Te) as radical scavengers. In these cases, complete conversion of 1^bi^ occurred within seconds, accompanied by formation of grey precipitates. ^1^H NMR spectroscopy confirmed clean formation of the corresponding [({N(TMS)_2_})(Cp*)Al(Ch-Ph)] species, 5–7. Interestingly, formation of [Cd({N(TMS)_2_})_2_] was similarly observed, but integration revealed only 0.5 equivalents, corresponding to a 2 : 1 mixture of 5–7 and cadmium amide (Ch = S: Fig. S33–S35; Ch = Se: Fig. S39–S42; Ch = Te: Fig. S46–S50).

Analysis of the reaction stoichiometry indicates that 0.5 equivalents of Cd(ChPh)_2_ precipitate from solution. The formation of Cd(ChPh)_2_ is expected to proceed *via* heteroleptic precursors [Cd{N(TMS)_2_}(ChPh)], which rapidly redistribute to yield 0.5 equivalents each of [Cd(ChPh)_2_] and [Cd({N(TMS)_2_})_2_]. All three cadmium chalcogenophenolates (Cd(ChPh)_2_; Ch = S (11), Se (12), Te (13)) are poorly soluble in non-coordinating and even many coordinating solvents, due to formation of extended 3D network structures composed of adamantane units.^68–70^ Dissolution of isolated 11–13 in pyridine-*d*_5_ confirmed their formation by NMR spectroscopy (Scheme 5; Ch = S: Fig. S36–S38; Ch = Se: Fig. S43–S46; Ch = Te: Fig. S51–S54). In summary, the bimetallic congener 1^bi^ exhibits similar aluminum transfer reactivity to 1^tri^, but cadmium remains bound in the form of cadmium amide or chalcogenide species, suppressing the formation of elemental Cd^0^. Isolation of the cadmium chalcogenides was achieved in >80% yield after workup.

### Extended quantum chemical calculations

**Scheme 6:**
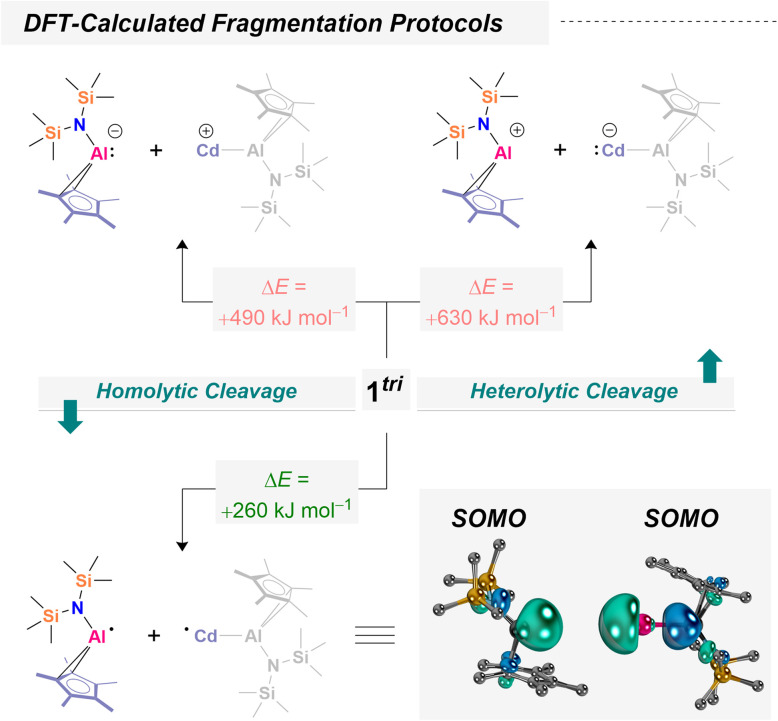
Quantum chemical assessment of vertical homo- and heterolytic cleavage energies of the Al–Cd bond in a trimetallic cadmium aluminyl. Energies and frontier orbitals at the TPSS-D4 CPCM(benzene) def2-QZVPP//r^2^SCAN-3c CPCM(benzene)/H-opt level of theory based on literature-extracted fragments of 1^tri^.^53^ Note: because all atoms except hydrogen were frozen during geometry relaxation, only electronic energies (*E*) were used for fragmentation comparisons; vibrational thermochemistry was not applied. These calculations furthermore shall not imply that homolytic cleavage takes place without a substrate.

**Fig. 1:**
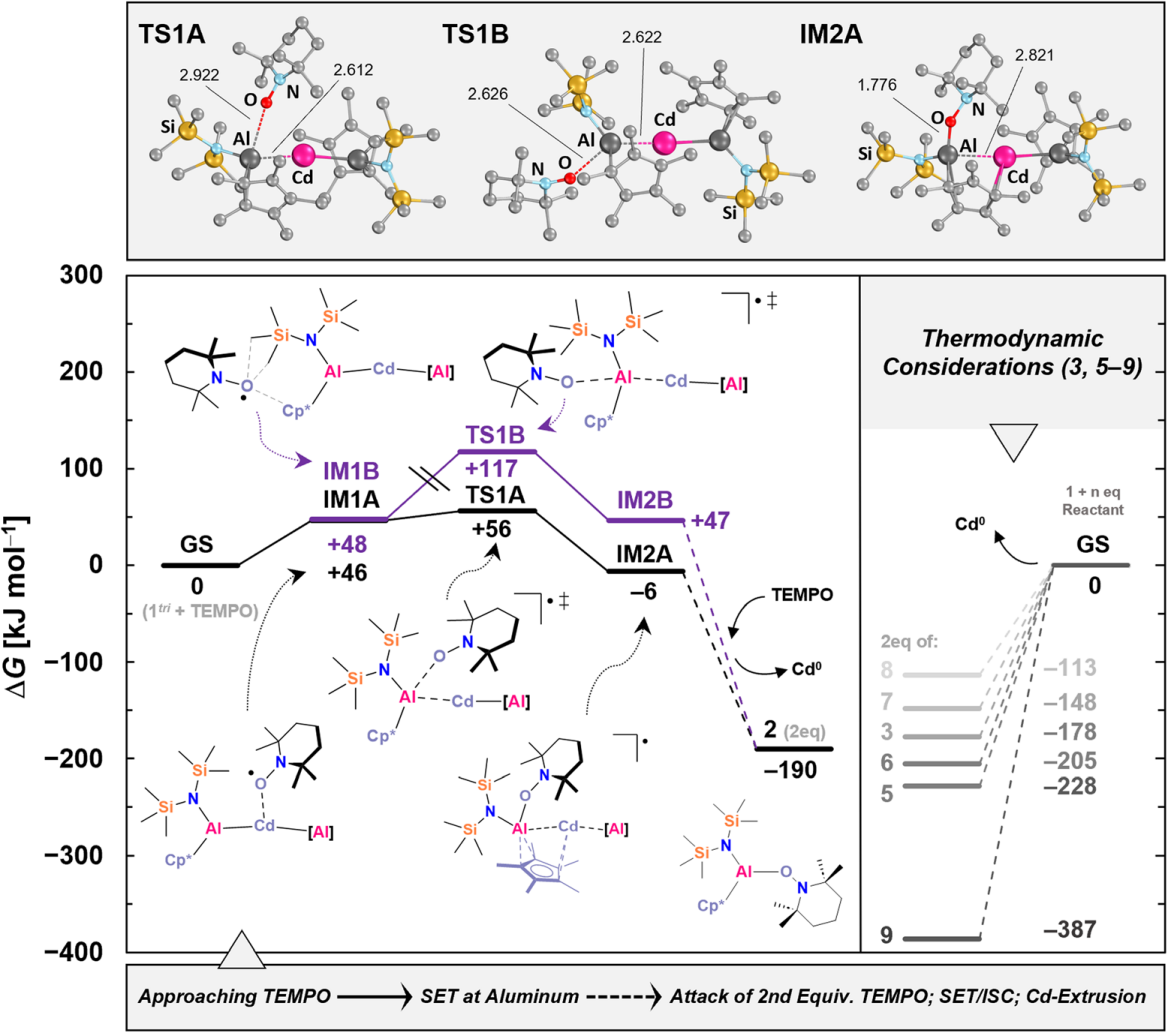
DFT-derived mechanism for Al(ii) transfer at 1^tri^ using TEMPO as well as further thermodynamic considerations at the TPSS-D4 CPCM(benzene) def2-QZVPP//r^2^SCAN-3c level of theory. Indicated bond lengths at transition states and intermediates are given in Å. Cd^0^ was modelled as a small Cd_8_ metal cluster that was extracted from the solid-state structure^71^ of cadmium.

As previously demonstrated for 1^bi^ and 1^tri^, intrinsic bond orbital (IBO) analysis provides an excellent tool to visualize the participation of each atom in bonding.^53^ These analyses reveal that the Al–Cd interaction is strongly covalent, yet its degree of covalency is tunable depending on the ligand environment. Experimentally (*vide supra*), we provided evidence for radical dissociation, confirmed through successful trapping. The plausibility for accessible and transferable Al(ii) fragments within Al–Cd covalent bonds motivated us to further investigate the nature of the Al–Cd interactions computationally. We began with a computational fragmentation study of 1^tri^, following robust models that mimic both homo- and heterolytic bond cleavage. Calculations at the TPSS-D4 CPCM(benzene)/def2-QZVPP//r^2^SCAN-3c/CPCM(benzene)/H-opt level of theory (see SI for details), revealed that homolytic Al–Cd bond dissociation is by far most favorable (as compared to heterolytic [Al]–[Cd] bond cleavage yielding [Al]^+^ + [Cd]^−^ or [Al]^−^ + [Cd]^+^). The bond dissociation energies are favored by more than 250 kJ mol^−1^ (Scheme 6). These results suggest that fragmentation furnishing Al(ii) may indeed yield thermodynamically viable pathways. This is only true though, when offering suitable reagents: a dissociation without radical scavenger is not expected due to the prohibitively high dissociation energy. To complement these thermodynamic considerations, we thus investigated the kinetic accessibility of the Al/Cd bonds in 1^tri^. As a representative system, we focused on the formation mechanism of 2, which (i) is computationally tractable and (ii) reflects a prototypical radical scavenging experiment. Additionally, thermodynamic considerations were extended to the formation of 3 and 5–9, providing a broader view of the energetic landscape associated with Al(ii) transfer and trapping. Our calculations (TPSS-D4 CPCM(benzene)/def2-QZVPP//r^2^SCAN-3c) indicate that TEMPO can approach the trimetallic framework of 1^tri^*via* multiple pathways (Fig. 1).

The chemically intuitive route involves initial coordination of TEMPO to cadmium, which is sterically accessible, forming intermediate IM1A. This step is endergonic by +46 kJ mol^−1^. Subsequently, TEMPO attacks one of the aluminum atoms, activating the Al–Cd bond through a transition state on the doublet surface. This step is slightly endergonic (TS1A: Δ*G*^‡^ = +56 kJ mol^−1^) and leads to intermediate IM2A, which is slightly stabilized relative to the starting materials (Δ*G* = −6 kJ mol^−1^). Spin density analysis of IM2A shows that the unpaired electron is delocalized between both Al centers and Cd, suggestive of a three-electron three center (3e3c) interaction (Fig. S80). Although this intermediate could not be trapped experimentally due to rapid reaction completion, it highlights the potential for peculiar electron deficient bonding between two different metals as an exciting target for future synthetic investigations. Further attack by TEMPO, spin reorganization, and cadmium liberation complete the pathway. The overall energy gain for the addition of two equivalents of TEMPO to 1^tri^, forming two equivalents of 2, is substantial (Δ*G* = −190 kJ mol^−1^; −95 kJ mol^−1^ per equivalent). A second computationally accessible pathway involves a backside attack of TEMPO (IM1B, Δ*G* = +48 kJ mol^−1^) between the supporting ligands [Cp*]^−^ and [{N(TMS)_2_}]^−^. Electron transfer to TEMPO in this scenario is kinetically less favorable, with a transition state associated with a Gibbs energy of Δ*G*^‡^ = +117 kJ mol^−1^ (TS1B), making the pathway inaccessible at ambient temperature. Formation of IM2B (Δ*G* = +47 kJ mol^−1^) is thus less relevant, although the structural motif of IM2B is similar to that of IM2A. Thermodynamic considerations for other experimentally observed products reveal consistent trends. Addition of benzophenone to form 8 yields Δ*G* = −56.5 kJ mol^−1^ per equivalent upon cadmium extrusion, which is the least energetically favored scenario and correlates with the observed decomposition of 8 during work-up. In contrast, formation of 9 is highly favorable (Δ*G* = −193.5 kJ mol^−1^ per equivalent), consistent with its stability in solution and successful isolation. The formation of 3 is energetically similar to that of 2 (Δ*G* = −89 kJ mol^−1^ per equivalent). The chalcogenide species 5–7 display decreasing thermodynamic stability from S (5) to Se (6) to Te (7), in agreement with their observed experimental stabilities: 5 remains stable for months under glovebox conditions at −30 °C, 6 decomposes over a few weeks, and 7 decomposes within approximately one week under the same conditions (see SI: Sections 3.4–3.6).

## Conclusions

In summary, the Al/Cd platform 1^tri^ unlocks an unprecedented regime of heterometal-guided Al(ii) transfer chemistry under exceptionally mild, thermal conditions. The ability to generate and selectively deliver an authentic Al(ii) fragment, without photochemical activation, marks a decisive expansion of reactivity accessible from aluminyl-based architectures. The broad substrate scope, spanning persistent nitroxides, dichalcogenides, and ketone-based spin traps, reveals a generalizable fragmentation manifold in which covalent Al–Cd linkages serve as controlled release points for reactive Al(ii) species concomitant with heterometal extrusion. Together with corroborating EPR analyses and quantum-chemical insight, these findings are firmly established. Notably, the complementary reactivity of the bimetallic partner 1^bi^, yields molecular cadmium-based building blocks as well, underscoring that both metals embedded in the Al/Cd framework can be transferred when paired with an appropriate reaction partner. Overall, our results chart a path toward the broader use of Al(ii) motifs in radical transformations and small-molecule activation chemistry in a predictable and chemoselective manner.

## Author contributions

D. H.: formal analysis, methodology, investigation, data curation, writing – review and editing. F. W.: formal analysis, data curation, writing – review and editing. C. L.: funding acquisition, methodology, validation, visualization, formal analysis, resources, writing – review and editing, data curation. F. D.: conceptualization, investigation, funding acquisition, writing – original draft, writing – review and editing, methodology, validation, visualization, formal analysis, project administration, data curation, supervision, resources. All authors contributed to drafting and revising the manuscript and have approved the final version for publication.

## Conflicts of interest

There are no conflicts to declare.

## Supplementary Material

SC-OLF-D6SC00437G-s001

SC-OLF-D6SC00437G-s002

## Data Availability

CCDC 2516482–2516488 contain the supplementary crystallographic data for this paper.^100*a*–*g*^ Supplementary information (SI): ref. 72–99 have been cited in the SI. See DOI: https://doi.org/10.1039/d6sc00437g.
