# Supplementary material for: Al(ii) transfer harnessing a well-defined cadmium precursor
Source: Chem Sci. 2026 Feb 23;17(16):7997–8006. doi: 10.1039/d6sc00437g (PMC12947373; doi:10.1039/d6sc00437g)
Supplement: SC-017-D6SC00437G-s001 [file SC-017-D6SC00437G-s001.pdf]

Supplementary Information (SI) for Chemical Science.
This journal is © The Royal Society of Chemistry 2026

SUPPORTING INFORMATION

Al(II) Transfer Harnessing a Well-Defined Cadmium Precursor

D. Herle,[a] F. Wurm,[b] C. Lichtenberg,[b] F. Dankert [a]*

Dominic Herle, Frerik Wurm, Prof. Dr. Crispin Lichtenberg, Dr. Fabian Dankert

[a] Institute of Chemistry, University of Kassel, Heinrich-Plett-Str. 40,

34132 Kassel, Germany

[b] Department of Chemistry, Philipps-University Marburg, Hans-Meerwein Str. 4,

35032 Marburg, Germany, mar.quest | Marburg Center for Quantum Materials and Sustainable Technologies, 35032 Marburg

Crispin.Lichtenberg@chemie.uni-marburg.de

* Fabian.Dankert@uni-kassel.de

This file includes:

1 Experimental details 2
2 Spectroscopy of selected starting materials 3
3 Syntheses of compounds and crystallization 6
4 NMR studies: Reactivity of **1***bi* 31
5 Additional NMR studies 49
6 EPR study 54
7 EDX analysis 57
8 X-ray structure elucidation and refinement 59
9 Computational details 69
10 References 94

1 Experimental details

General Information. All manipulations were carried out under oxygen- and moisture-free conditions under an inert atmosphere of argon using standard Schlenk techniques or a glovebox.[1] All reactants were stored and handled in an UNIlab mBraun glovebox with integrated freezer. Solvents and reactants were either obtained from commercial sources, local trade or synthesized according to literature procedures. Activation of molecular sieves (3Å, 4Å) was achieved through several microwave irradiation cycles (700W; one minute each). Further activation followed through applying vacuum for several hours. All sieves were subsequently stored under inert atmosphere. Tetramethylsilane was dried over molecular sieves (3 Å) and subsequently stored under inert atmosphere in an ampule with PTFE valve (FengTecEx). Benzene-d_6 (Deutero) was degassed using the freeze-pump thaw method and subsequently dried over molecular sieves (4 Å). Once transferred to the glovebox, it was stored over a new set of freshly activated molecular sieves. Diphenyldisulfide was purchased from J&K Scientific and used as received. Diphenyldiselenide was purchased from Sigma Aldrich and used as received. Diphenylditelluride was purchased from Acros Organics and used as received. (2,2,6,6-Tetramethylpiperidin-1-yl)oxyl (TEMPO), 4-Methoxy-(2,2,6,6-Tetramethylpiperidine-1-yl)oxyl (4-MeO-TEMPO) and 9-Azabicyclo[3.3.1]nonane N-oxyl (ABNO) were purchased from BLD Pharm and used as received. Benzophenone and Di(2-pyridyl) ketone were purchased from Sigma Aldrich and used as received. $(AlCp^*)_4$ was synthesized *via* reductive elimination according to a procedure reported by FISCHER.[2] $Cd\{N(TMS)_2\}_2$ was synthesized according to a procedure reported by WANNAGAT.[3] NMR-data were recorded on Jeol JNM-ECZL500 or Varian VNMRS-500 MHz spectrometers at 25°C. NMR spectra are referenced internally to the deuterated solvent (^{13}C: C_6D_6 δref = 128.06 ppm) or to protic impurities in the deuterated solvent (^{1}H: C_6HD_5 δref = 7.16 ppm) or externally (^{27}Al: $Al(NO_3)_3$ in H_2O, δref = 0 ppm; ^{29}Si: Tetramethylsilane, δref = 0 ppm, δref = 0 ppm; ^{113}Cd: $CdMe_2$, δref = 0 ppm; 77Se, $SeMe_2$ δref = 0 ppm; ^{125}Te: $TeMe_2$, δref = 0 ppm). The full width at half maximum (FWHM) for the signals in ^{27}Al, ^{113}Cd and ^{125}Te NMR spectra is denoted as $\omega_{1/2}$. CHN elemental microanalyses were conducted with a HEKAtech Euro EA CHNS elemental analyser. UV-VIS spectra were recorded on a Shimadzu UV-2600 spectrometer in quartz cells with a path length of 1 cm. X-band EPR spectroscopic measurements were carried out under inert conditions at 23 °C, using a Bruker ELEXSYS E580 CW/FT EPR spectrometer. The spectral simulations were performed using MATLAB 9.6 (2019a) and the EasySpin 5.2.25 toolbox.[4] HR-LIFDI mass spectra were acquired with a AccuTOF GCv 4G (Jeol) Time of Flight (TOF) mass spectrometer. An internal or external standard was used for drift time correction. The LIFDI ion source and FD-emitters were purchased from Linden ChroMasSpec GmbH (Bremen, Germany).

2 Spectroscopy of selected starting materials

This section reports NMR spectra for selected starting materials that were synthesized and to report references for some of the herein performed NMR studies.

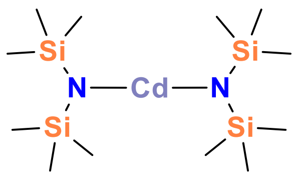

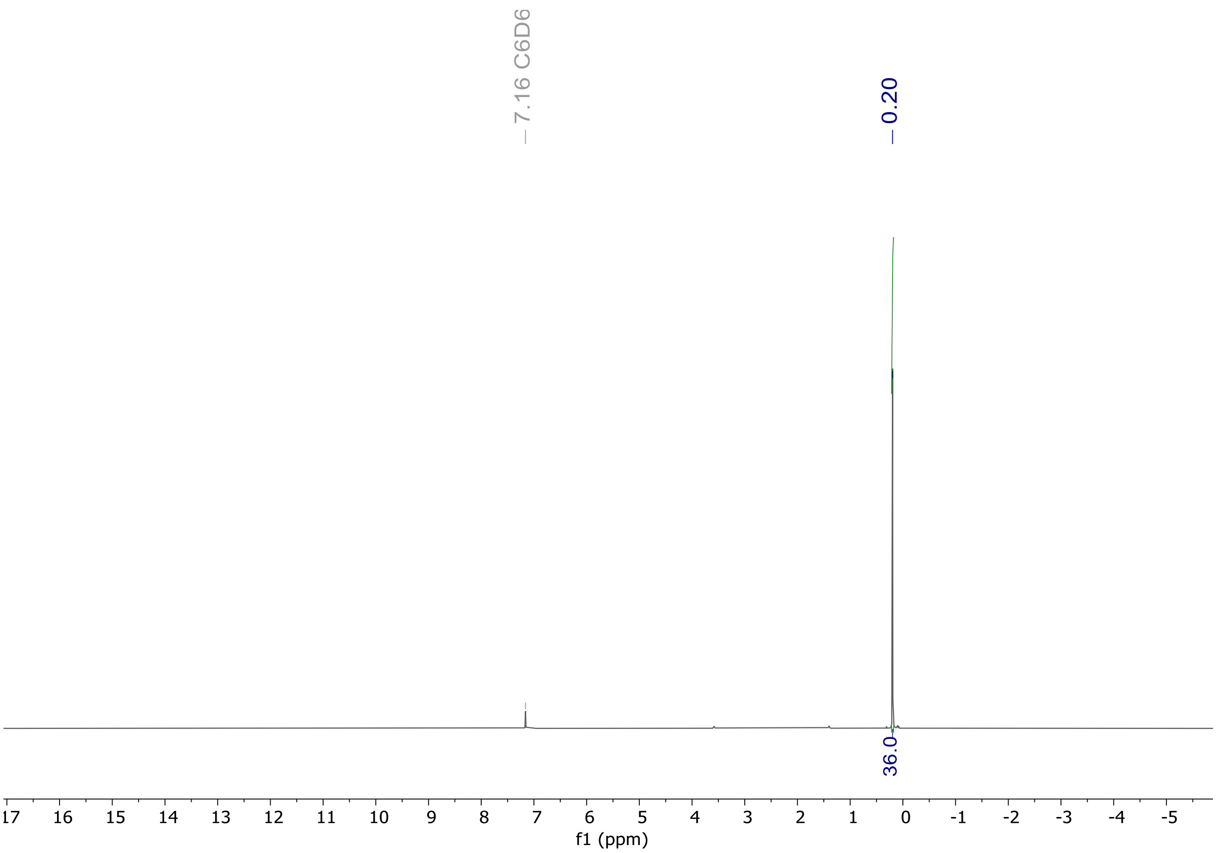

Figure S1: ^{1}H NMR (500 MHz, C_6D_6) of Cd{N(TMS)$_2$}$_2$.

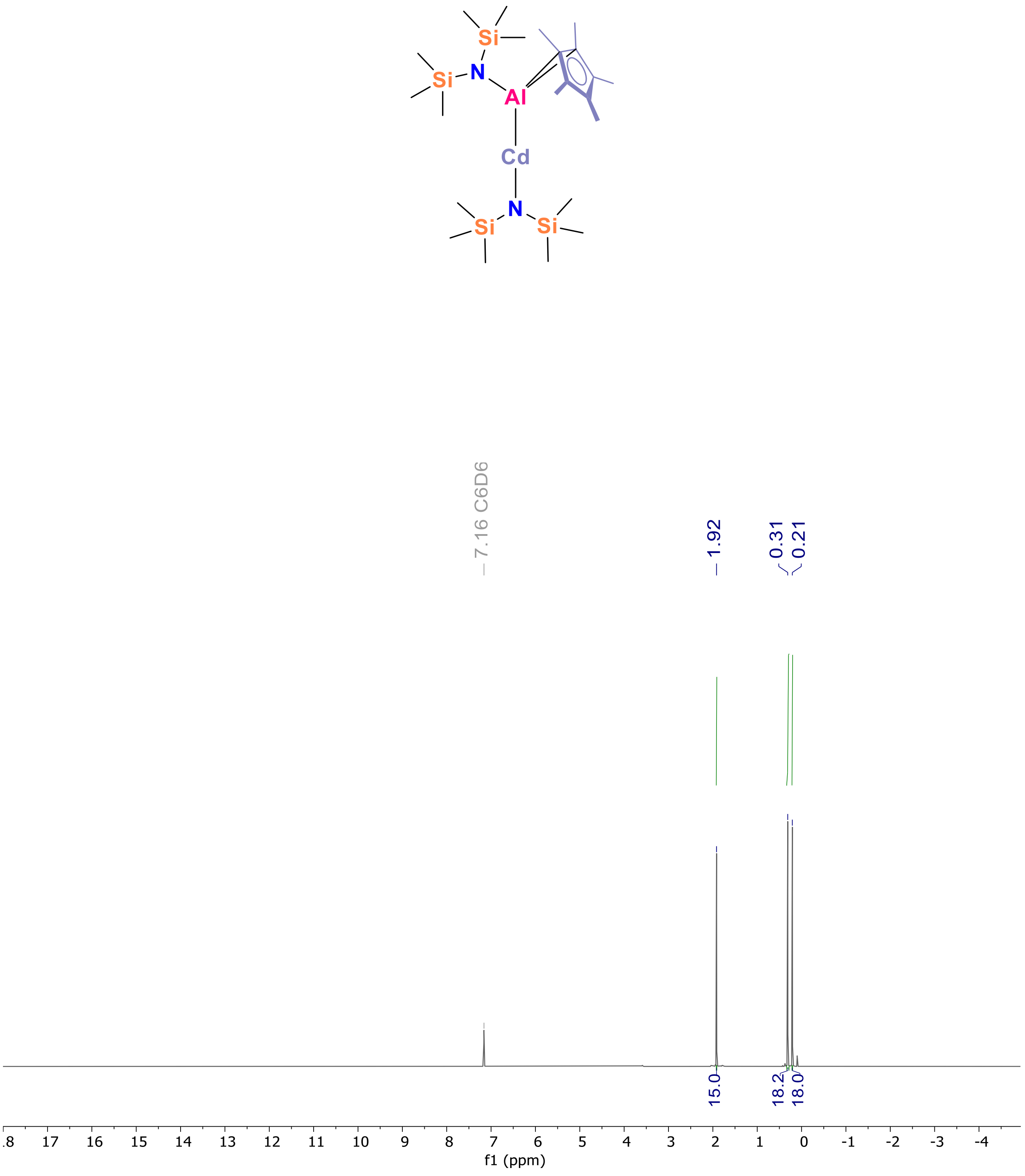

Figure S2: ^{1}H NMR (500 MHz, C_6D_6) of **1*bi***.

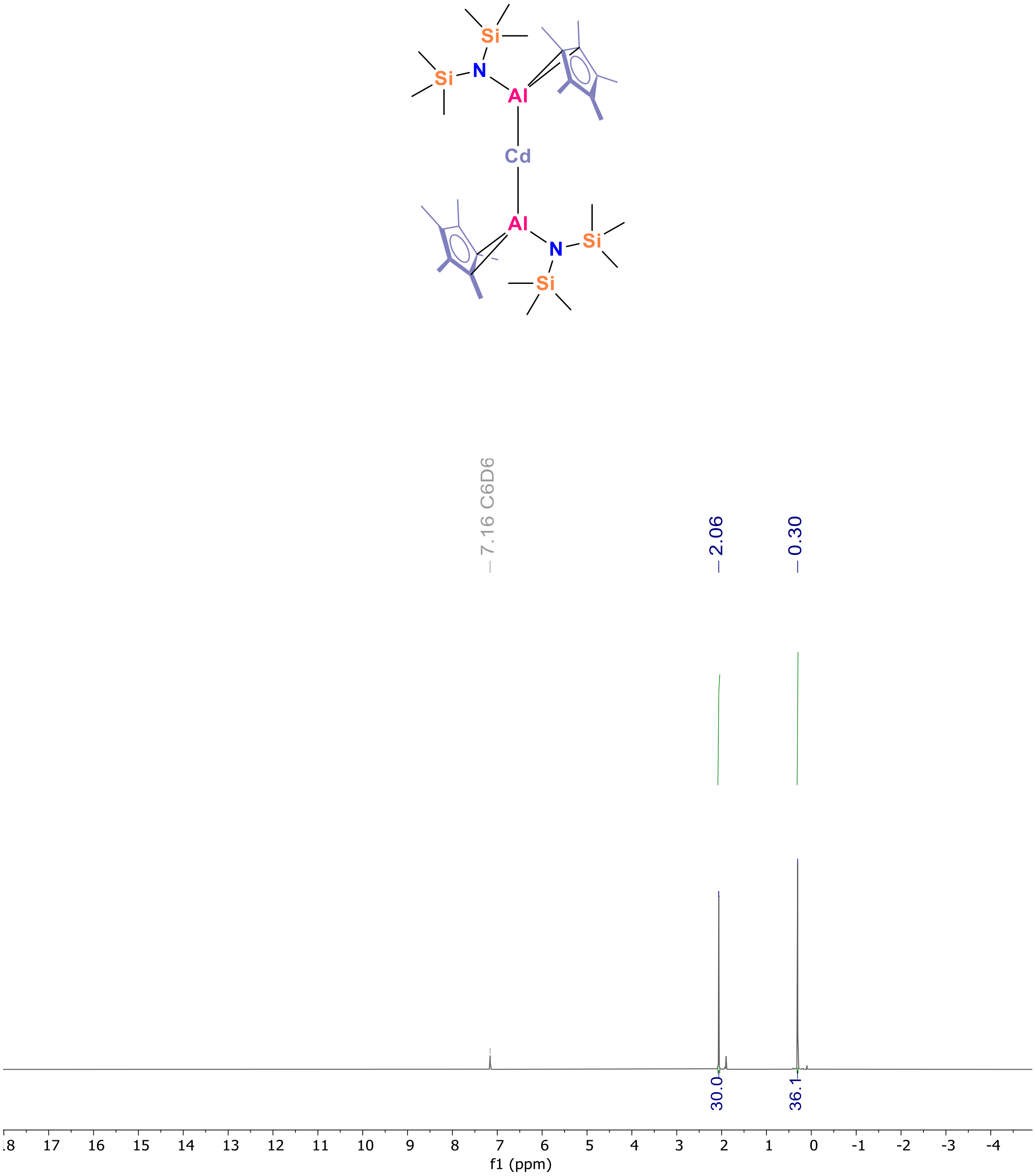

Figure S3: ^{1}H NMR (500 MHz, C_6D_6) of **1*tri***.

3 Syntheses of compounds and crystallization

3.1 [({N(TMS)$_2$})(Cp*)Al(TEMPO}] (2)

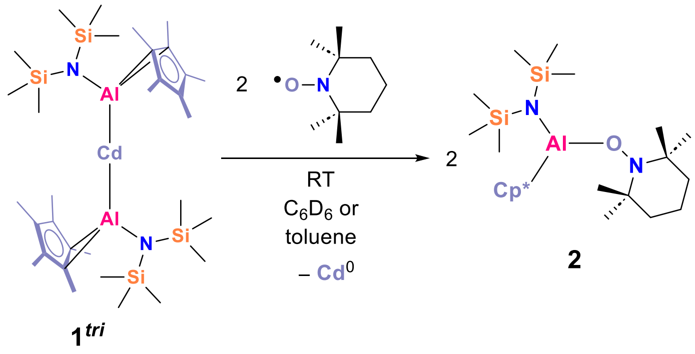

<u>*Upscaled Experiment:*</u>

In an ampoule with PTFE valve (FengTecEx), **1*^{tri}*** was synthesized *in-situ* in a 0.115 mmol scale (based on Cd{N(TMS)$_2$}$_2$) in 3 mL of toluene.[5] Next, TEMPO (32.3 mg, 0.207 mmol, 1.8 eq.) was dissolved in 0.5 mL of toluene and added to the ampoule, leading to the formation of a black precipitate. The solvent was removed under reduced pressure and thoroughly dried in vacuo. In the glovebox, 3 mL of *n*-pentane was then added to extract the product followed by filtration through a PTFE syringe filter (d = 13 mm; pore size 0.22 µm). Slow evaporation of the *n*-pentane yields colorless plates of **2** (Yield: 97 mg, 88 %).

The obtained crystals throughout work-up were suitable for SC XRD studies. *Note*: **2** shows a high stability when storing the compound in the freezer.

<u>*Analytical Data:*</u>

^{1}H NMR (500 MHz, C_6D_6, 298 K) ***δ***: 2.02 (s, 15H, −C*H$_3$* of Cp*), 1.43 (m, 6H, −C*H*$_2$ of TMP–O), 1.20 (br, 12H, −C*H$_3$* of TMP–O), 0.31 (s, 18H, −C*H$_3$* of HMDS) ppm.

^{13}C{^{1}H} NMR (126 MHz, C_6D_6, 298 K) ***δ***: 117.96 (s, *C*$_q$ of Cp*), 59.38 (s, *C$_q$ of TMP–O*), 40.5 (s, −*C*H$_2$ of TMP–O), 34.7 (br, −*C*H$_3$ of), 19.8 (br, –*C*H$_3$ of *TMP–O*), 17.6 (s, −*C*H$_2$ of *TMP*–O), 12.4 (s, −*C*H$_3$ of Cp*), 5.8 (s, −*C*H$_3$ of HMDS) ppm.

^{27}Al NMR (130 MHz, C_6D_6, 298 K) ***δ***: no resonance observed in a spectroscopic range of +300 – -300 ppm.

^{29}Si{^{1}H} NMR (99 MHz, C_6D_6, 298 K) ***δ***: −0.1 (s, *Si* of HMDS) ppm.

Elemental Analysis calc. for $C_{25}H_{51}Al_1N_2OSi_2$ (found) C 62.71 (61.96), H 10.74 (10.87), N 5.85 (5.51).

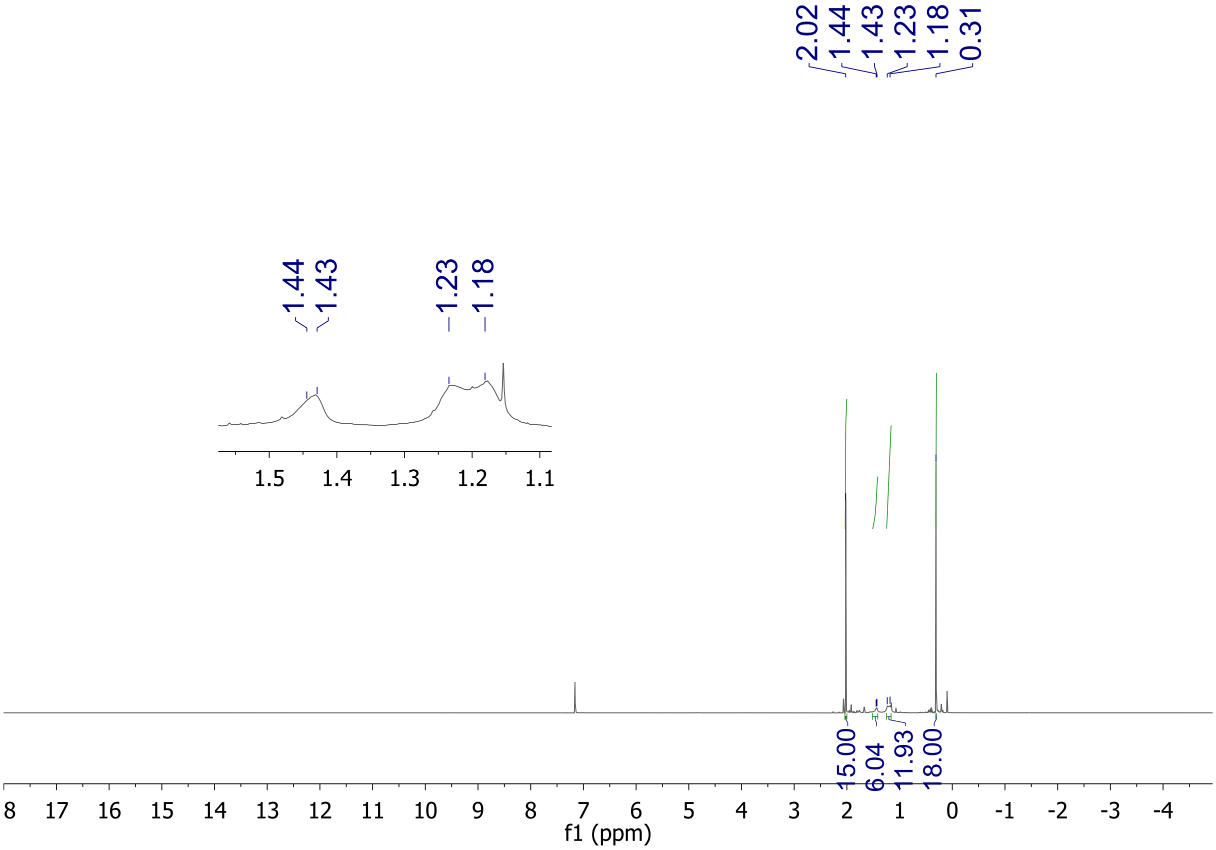

Figure S4: ^{1}H NMR (500 MHz, C_6D_6) of **2**.

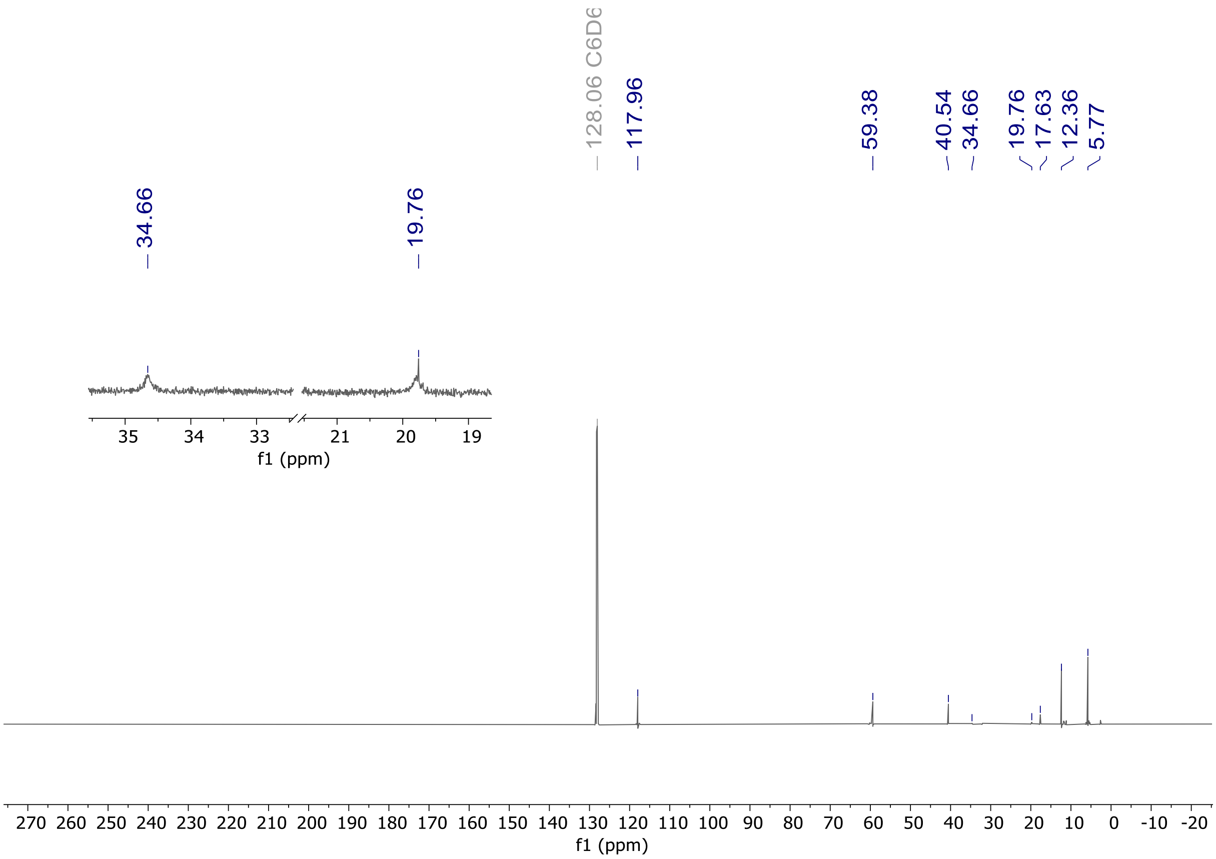

Figure S5: $^{13}C\{^{1}H\}$ NMR (126 MHz, C_6D_6) of **2**.

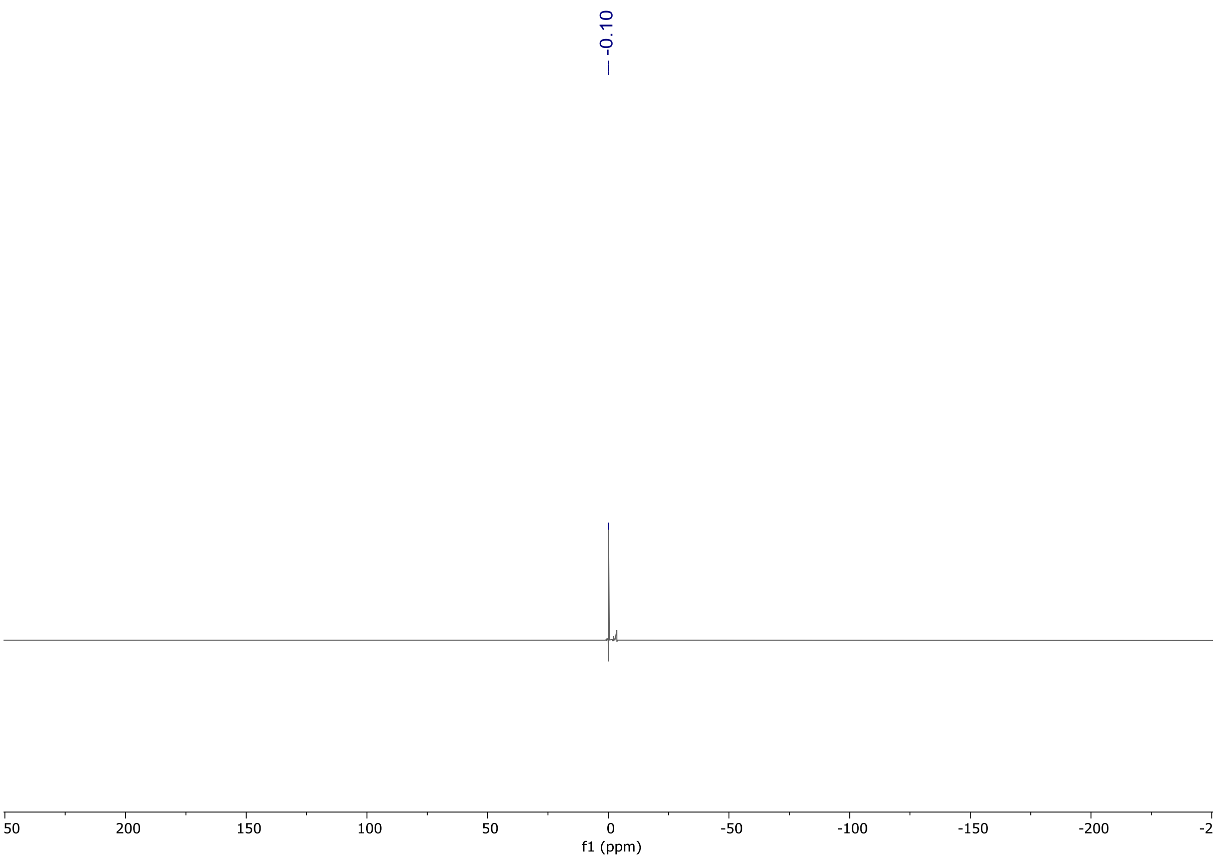

Figure S6: $^{29}Si\{^1H\}$ NMR (99 MHz, C_6D_6) of **2**.

3.2 [{N(TMS)$_2$}(Cp*)Al(4-MeO-TEMPO)] (3)

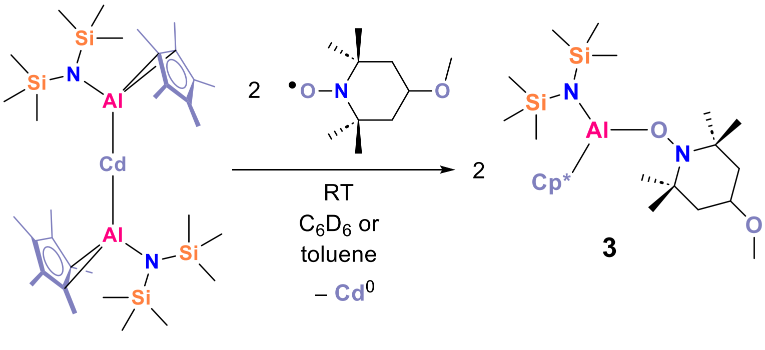

$\mathbf{1}^{tri}$

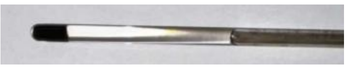

NMR Scale (Exemplarily to show Cd^0 precipitation (right)):
In an NMR tube, **1**^{tri} was synthesized according to literature on a 0.028 mmol scale (based on Cd{N(TMS)$_2$}$_2$) in 0.6 mL C_6D_6.[5] Then, 4-MeO-TEMPO (10.3 mg, 0.055 mmol,1.98 eq.) dissolved in 0.2 mL C_6D_6 was added to the NMR tube. Immediately after addition a black precipitate formed. NMR spectroscopic monitoring showed a near quantitative formation of **3**.

Upscaled Experiment:
In an ampoule with PTFE valve (FengTecEx), **1**^{tri} was synthesized *in-situ* on a 0.073 mmol scale (based on Cd{N(TMS)$_2$}$_2$) in 3 mL of toluene.[5] Next, 4-MeO-TEMPO (27.1 mg, 0.146 mmol, 2 eq.) was dissolved in 0.5 mL of toluene and added to the ampoule, leading to the formation of a black precipitate. The solvent was removed under reduced pressure and thoroughly dried in vacuo. In the glovebox, 3 mL of *n*-pentane was then added to extract the product followed by filtration through a PTFE syringe filter (d = 13 mm; pore size 0.22 µm). Slow evaporation of the *n*-pentane yields colorless plates of **3** (Yield: 72 mg, 97%).

The obtained crystals throughout work-up were suitable for SC XRD studies. *Note*: No signs of decomposition observed while storing in the glovebox integrated freezer.

Analytical Data:

^{1}H NMR (500 MHz, C_6D_6, 298 K) δ: 3.38 (m, 1H, *H*−C−OCH$_3$), 3.17 (s, 3H, O−C*H$_3$*) 2.00 (s, 15H, −C*H*$_3$ of Cp*), 1.54 (m, 4H, −C*H*$_2$), 1.25 (s, 6H, −C*H*$_3$ of), 1.17 (s, 6H, −C*H*$_3$ of 4-MeO-TMP–O), 0.31 (s, 36H, −C*H*$_3$ of HMDS) ppm.

^{13}C{^{1}H} NMR (126 MHz, C_6D_6, 298 K) δ: 117.8 (s, C_q of Cp*), 71.9 (br, *C*-O-CH$_3$), 59.8 (s, C_q of 4-MeO-TMP–O), 55.4 (s, O-*C*H$_3$ of 4-MeO-TMP–O), 45.7 (br, *C*H$_2$ of 4-MeO-TMP–O), 34.7 (br, *C*H$_3$ of 4-MeO-TMP–O), 20.8 (br, *C*H$_3$ of 4-MeO-TMP–O), 12.3 (s, −*C*H$_3$ of Cp*), 5.8 (s, −*C*H$_3$ of HMDS) ppm.

^{27}Al NMR (130 MHz, C_6D_6, 298 K) δ: no resonance observed in a spectroscopic range of +300 – -300 ppm.

$^{29}Si\{^1H\}$ NMR (99 MHz, C_6D_6, 298 K) δ: −0.1 (s, *Si* of HMDS) ppm.

Elemental Analysis calc. for $C_{26}H_{53}Al_1N_2O_2Si_2$ (found) C 61.37 (60.53), H 10.50 (10.60), N 5.51 (5.33).

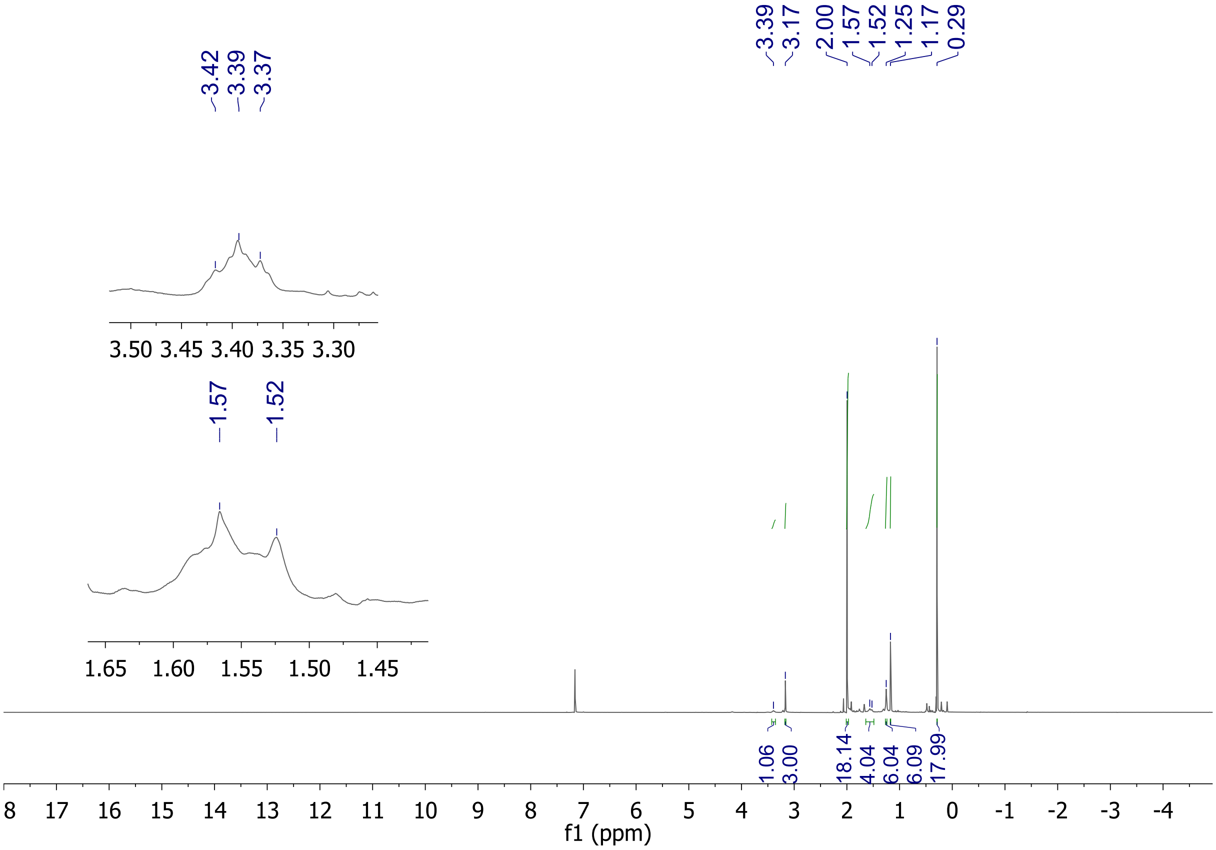

Figure S7: ^{1}H NMR (500 MHz, C_6D_6) of **3**.

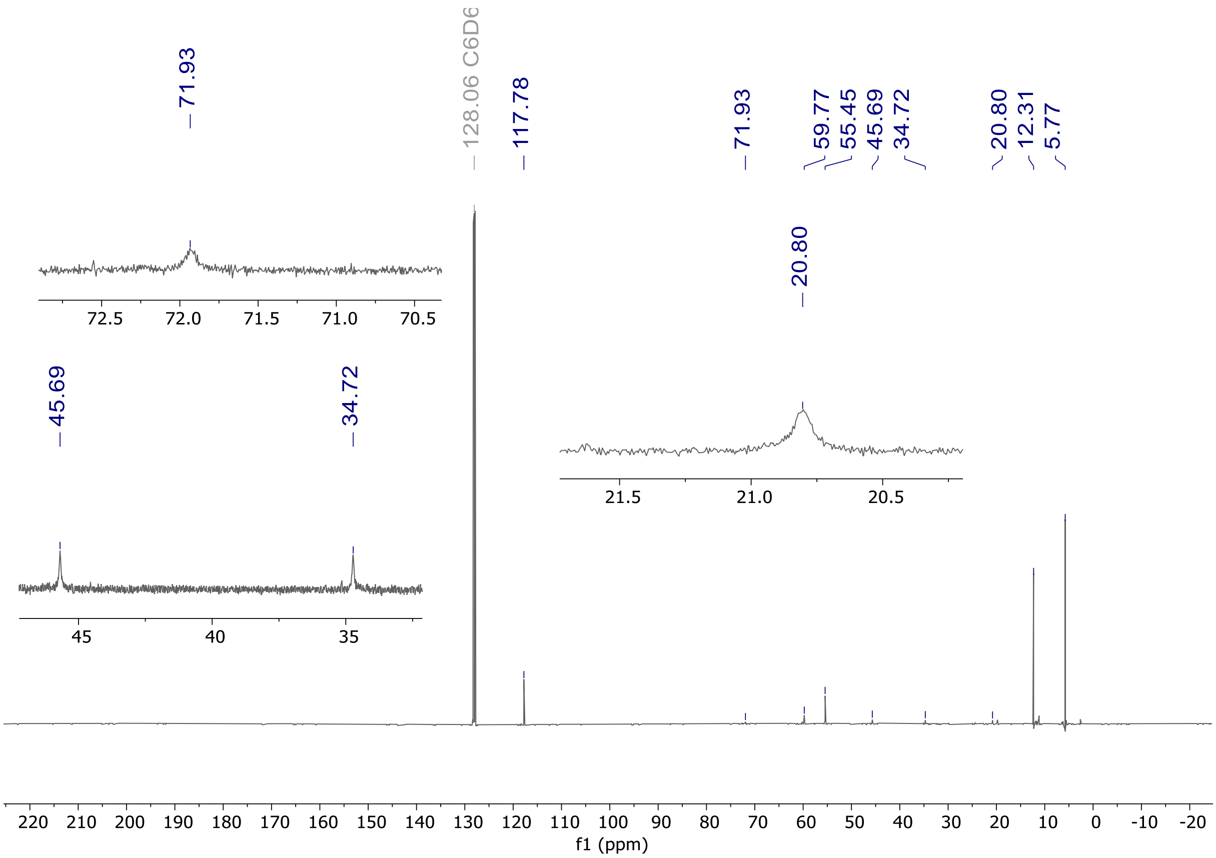

Figure S8: $^{13}C\{^{1}H\}$ NMR (126 MHz, C_6D_6) of **3**.

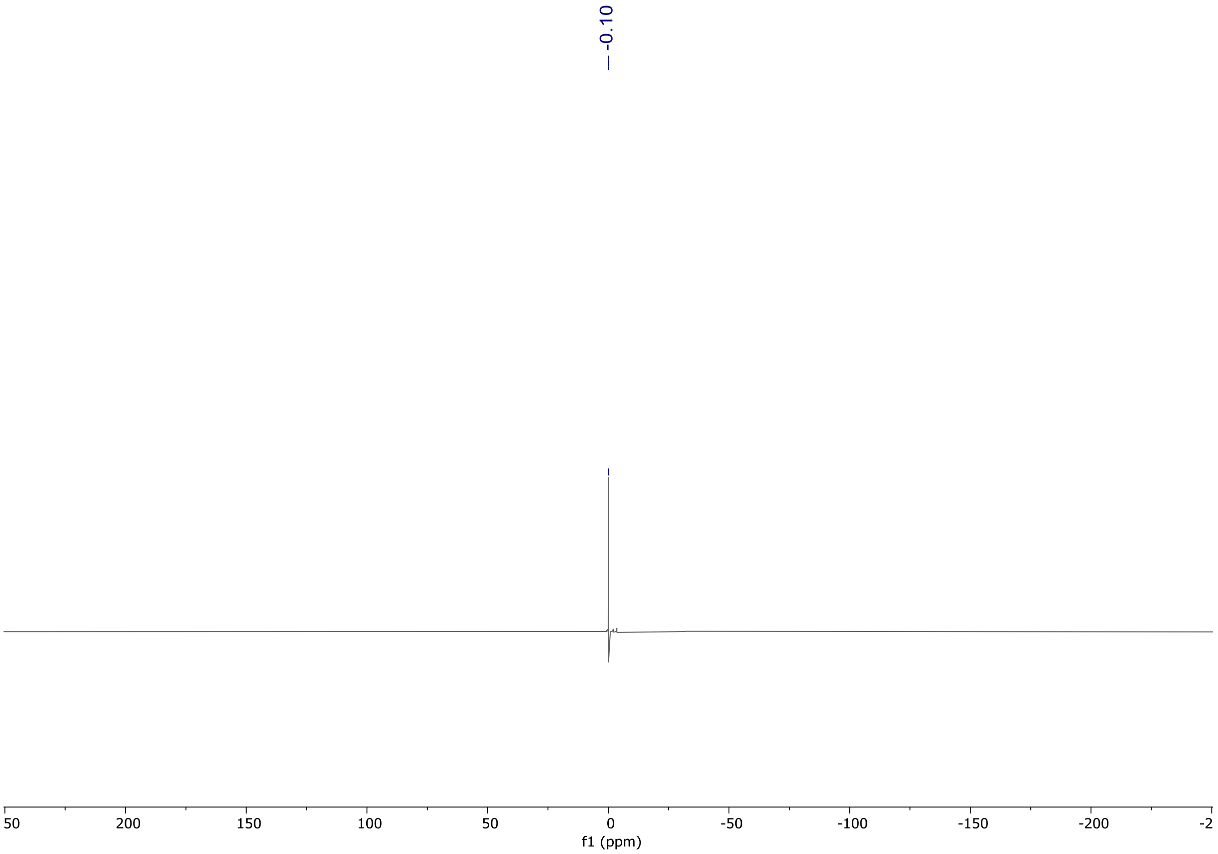

Figure S9: $^{29}Si\{^{1}H\}$ NMR (99 MHz, C_6D_6) of **3**.

3.3 [{N(TMS)$_2$}Al(ABNO)$_2$] (4)

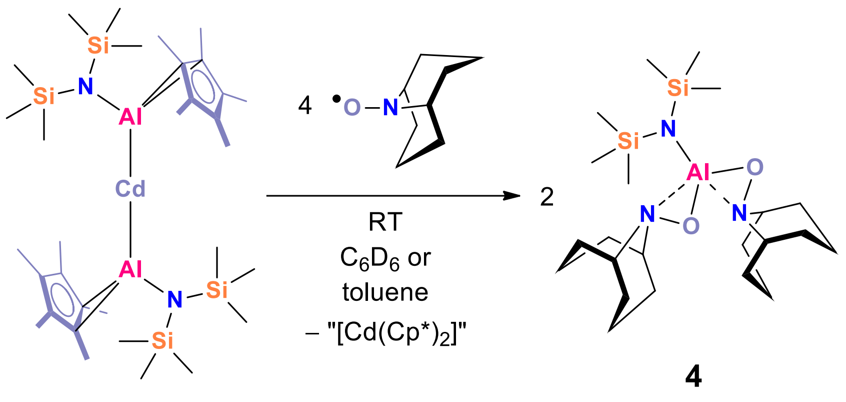

1^{tri} **4**

In an ampoule with PTFE valve (FengTecEx), **1**^{tri} was synthesized *in-situ* on a 0.07 mmol scale (based on Cd{N(TMS)$_2$}$_2$) in 3 mL of toluene.[5] Next, ABNO (41 mg, 0.293 mmol, 4 eq.) was dissolved in 0.5 mL of toluene and added to the ampoule, leading to the formation of some black precipitate. The solvent was removed under reduced pressure and thoroughly dried in vacuo. In the glovebox, 3 mL of *n*-pentane was then added to extract the product followed by filtration through a PTFE syringe filter (d = 13 mm; pore size 0.22 µm). Slow evaporation of the *n*-pentane yields colorless plates and a smeary grease. The mixture was recrystallized from a small amount of Tetramethylsilane. The crystals obtained were then once more washed with 0.5 mL of cold Tetramethylsilane. **4**·Si(CH$_3$)$_4$ was obtained as colorless crystals. (Yield: 21 mg, 26%).

Crystallization from *n*-pentane gave a small amount of suitable crystals for X-ray diffraction.

Analytical Data:

^{1}H NMR (500 MHz, C_6D_6, 298 K) ***δ***: 3.43 (m, 4H, N-C*H*), 2.75 (m, 4H), 2.13 (m, 4H, C*H*$_2$), 1.55 (m, 8H, C*H*$_2$), 1.44 (m, 2H, C*H*$_2$) 1.16 (6H, C*H*$_2$), 0.46 (s, 18H, C*H*$_3$ of HMDS) ppm. (Signals assigned according to ^{1}H-^{13}C-HSQC.)

^{13}C{^{1}H} NMR (126 MHz, C_6D_6, 298 K) ***δ***: 56.3 (s, *C*-N), 31.3 (s, *C*H$_2$), 23.0 (s, *C*H$_2$), 20.0 (s, *C*H$_2$), 19.5 (s, *C*H$_2$), 5.16 (s, *C*H$_3$ of HMDS) ppm. (Signals assigned according to ^{1}H-^{13}C-HSQC)

^{27}Al NMR: (130 MHz, C_6D_6, 298 K): no resonance observed in a spectroscopic range of +300 – -300 ppm.

^{29}Si{^{1}H} NMR (99 MHz, C_6D_6, 298 K) ***δ***: -0.1 (s, *Si* of HMDS) ppm.

Elemental Analysis calc. for $C_{22}H_{46}AlN_3O_2Si_2$·Si(CH$_3$)$_4$ (found) C 56.17 (56.41), H 10.51 (9.8), N 7.56 (7.26).

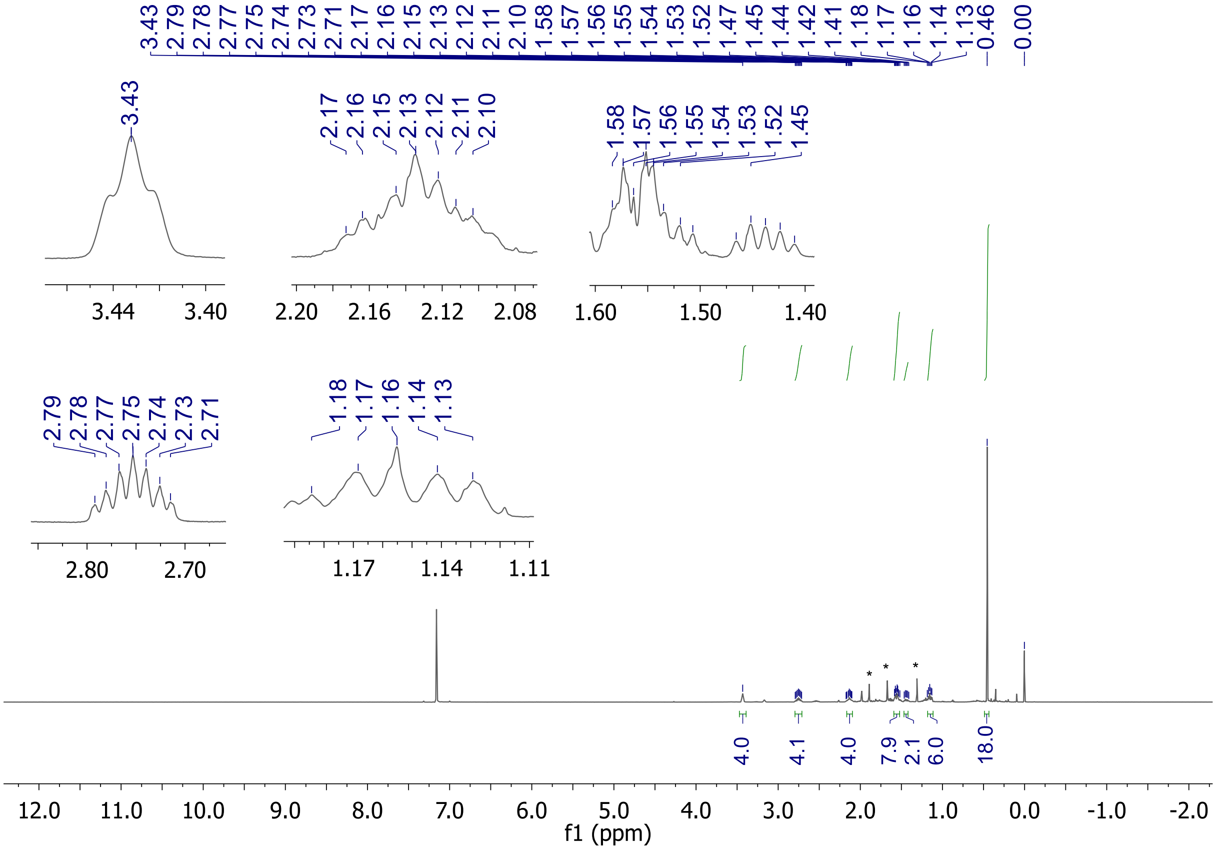

Figure S10: ^{1}H NMR (500 MHz, C_6D_6) of **4**. 0 ppm: Tetramethylsilane. Unidentified *trace* Cp*-based impurities are marked with *.

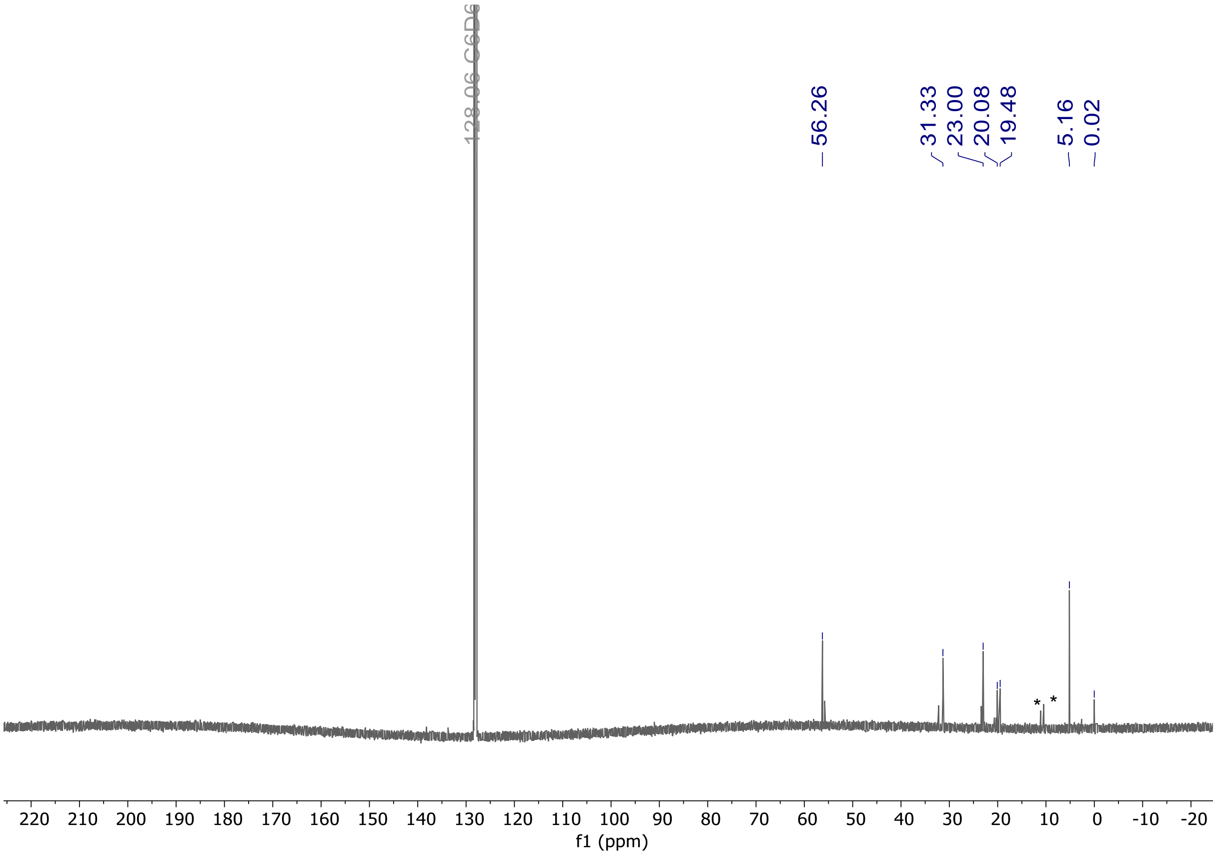

Figure S11: $^{13}C\{^1H\}$ NMR (126 MHz, C_6D_6) of **4**. Unidentified trace Cp* impurities are marked with a *. 0 ppm: Tetramethylsilane.

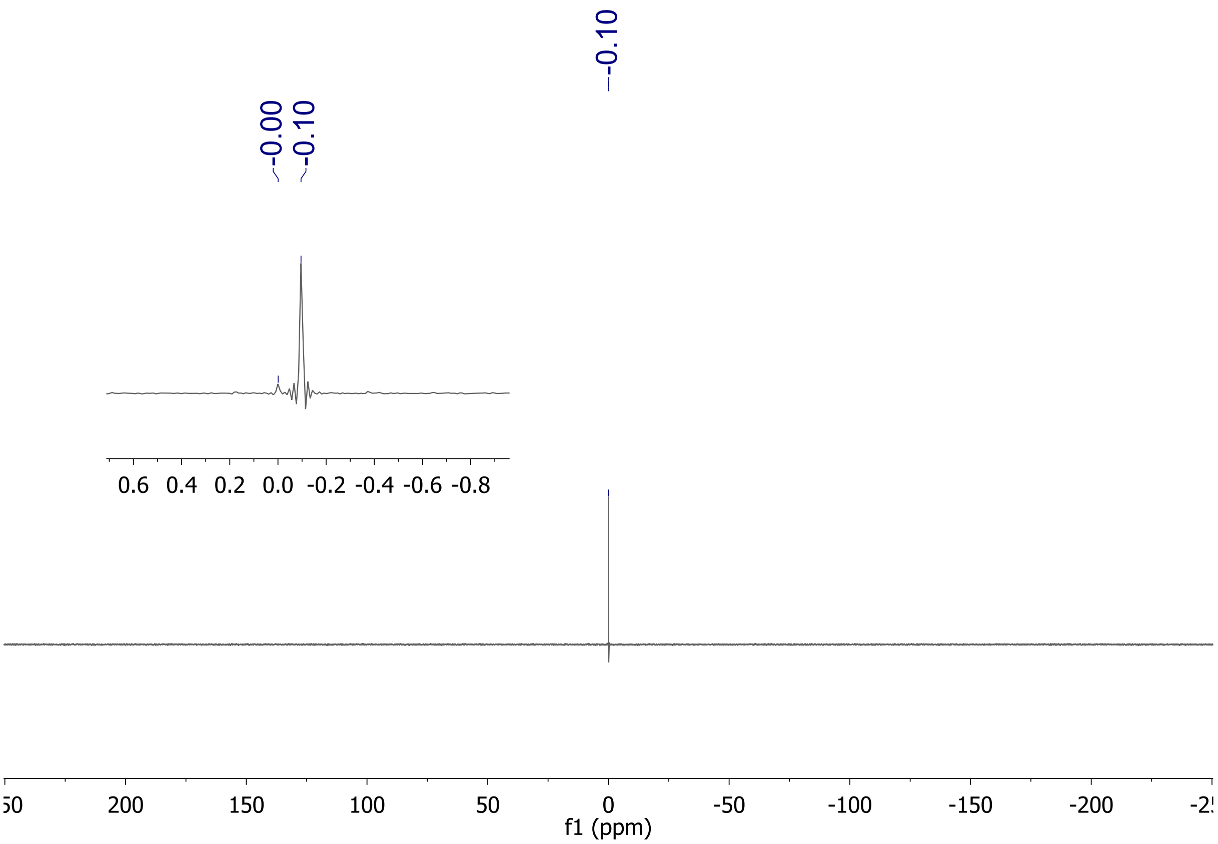

Figure S12: $^{29}Si\{^1H\}$ NMR (99 MHz, C_6D_6) of **4.** 0 ppm: Tetramethylsilane.

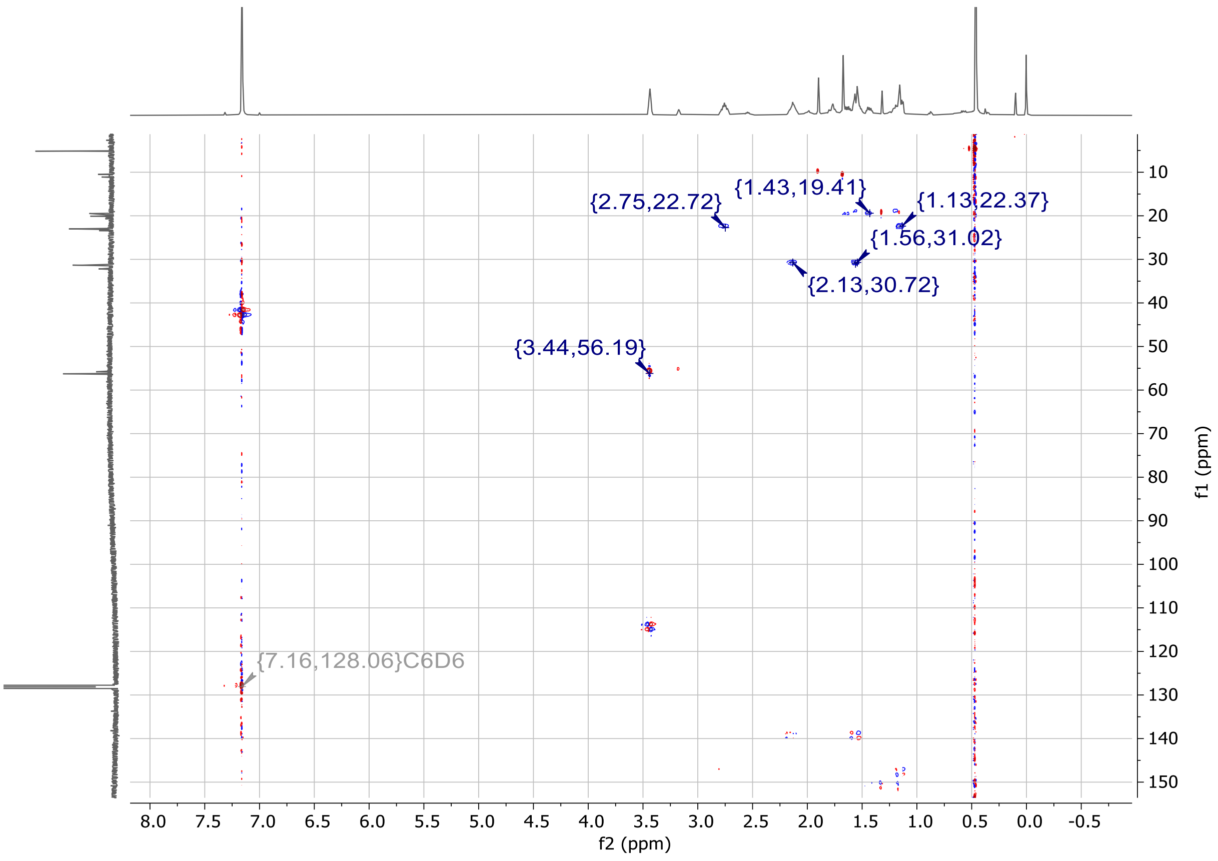

Figure S13: ^1H-^{13}C-HSQC NMR (500; 126 MHz, C_6D_6) of **4**.

3.4 [({N(TMS)$_2$})(Cp*)Al(SPh)] (5)

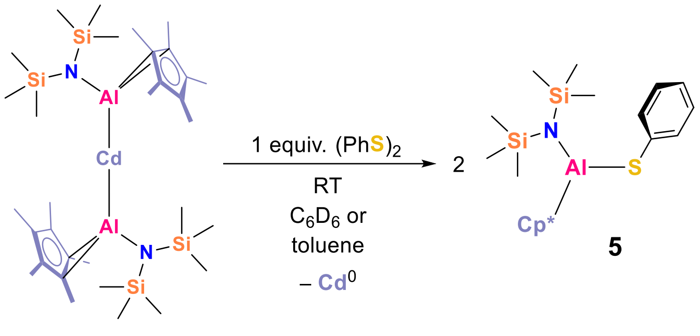

1^{tri}

In an ampoule with PTFE valve (FengTecEx), **1**^{tri} was synthesized *in-situ* on a 0.08 mmol scale (based on Cd{N(TMS)$_2$}$_2$) in 3 mL of toluene.[5] Next, diphenyldisulfide (17.4 mg, 0.08 mmol, 1 eq.) was dissolved in 0.5 mL of toluene and added to the ampoule, leading to the formation of a black precipitate. The solvent was removed under reduced pressure and thoroughly dried in vacuo. In the glovebox, 3 mL of *n*-pentane was then added to extract the product followed by filtration through a PTFE syringe filter (d = 13 mm; pore size 0.22 µm). Slow evaporation of the *n*-pentane yields colorless plates of **5** (yield: 67 mg, 97%). *Note*: Indefinite stability when storing the compound in a glovebox integrated freezer at –30°C.

Analytical Data:

^{1}H NMR (500 MHz, C_6D_6, 298 K) ***δ***: 7.44 (m, 2H, C*H*$_{Ar}$), 6.95 (m, 3H, C*H*$_{Ar}$), 1.97 (s, 15H, –C*H*$_3$ of Cp*), 0.08 (s, 18H, –C*H*$_3$ of HMDS) ppm.

^{13}C{^{1}H} NMR (126 MHz, C_6D_6, 298 K) ***δ***: 136.3 (s, *C*H$_{Ar}$), 135.6 (s, *C*$_q$ of Ar), 128.7 (s, *C*H$_{Ar}$), 126.1 (s, *C*H$_{Ar}$), 117.7 (s, *C*$_q$ of Cp*), 11.7 (s, *C*H$_3$ of Cp*), 4.7 (s, *C*H$_3$ of HMDS) ppm.

^{27}Al NMR (130 MHz, C_6D_6, 298 K) ***δ***: no resonance observed in a spectroscopic range of +300 – -300 ppm.

^{29}Si{^{1}H} NMR (99 MHz, C_6D_6, 298 K) ***δ***: −1.5 (s, *Si* of HMDS) ppm.

Elemental Analysis calc. for $C_{22}H_{38}Al_1NS_1Si_2$ (found) C 61.2 (60.12), H 8.87 (8.44), N 3.24 (2.92), S 7.43 (6.66) *.

*We provide the best value obtained after several measurements of freshly synthesized, crystalline samples.

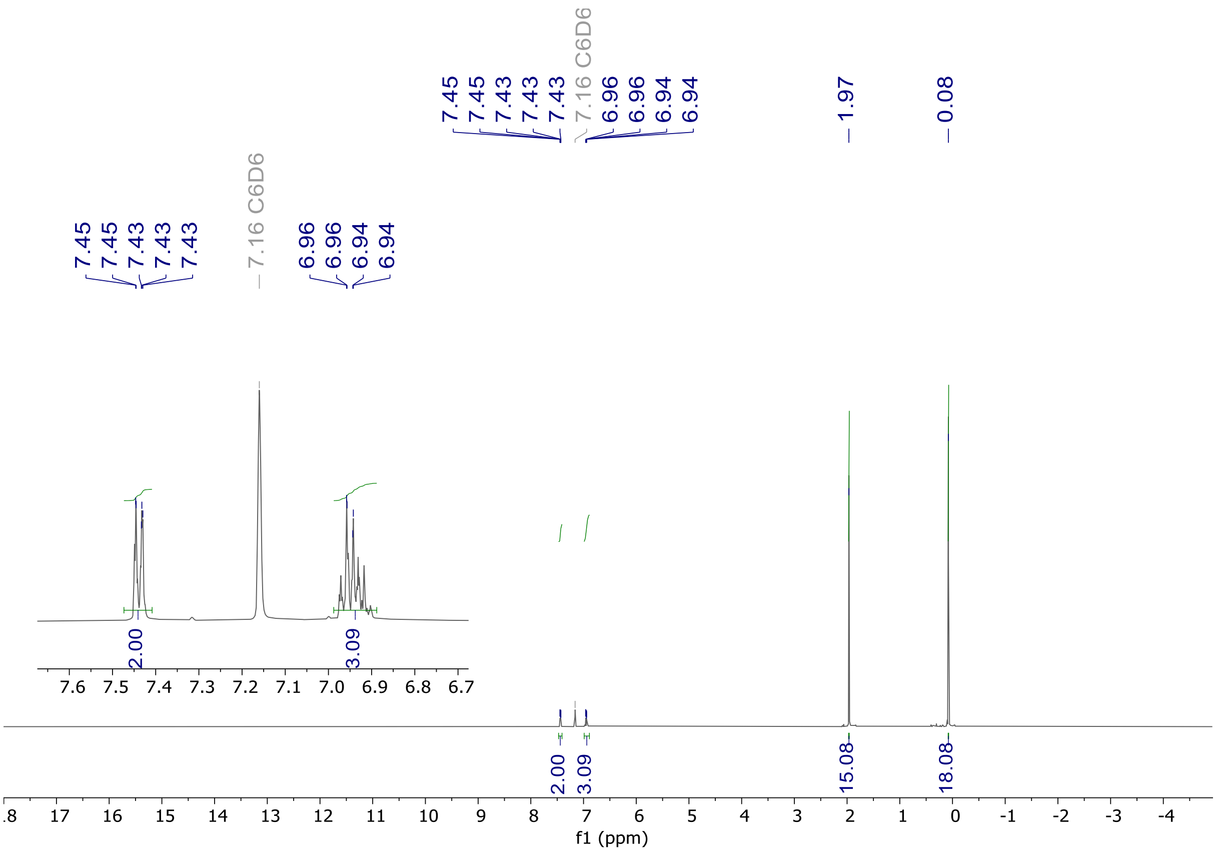

Figure S14: ^{1}H NMR (500 MHz, C_6D_6) of **5**.

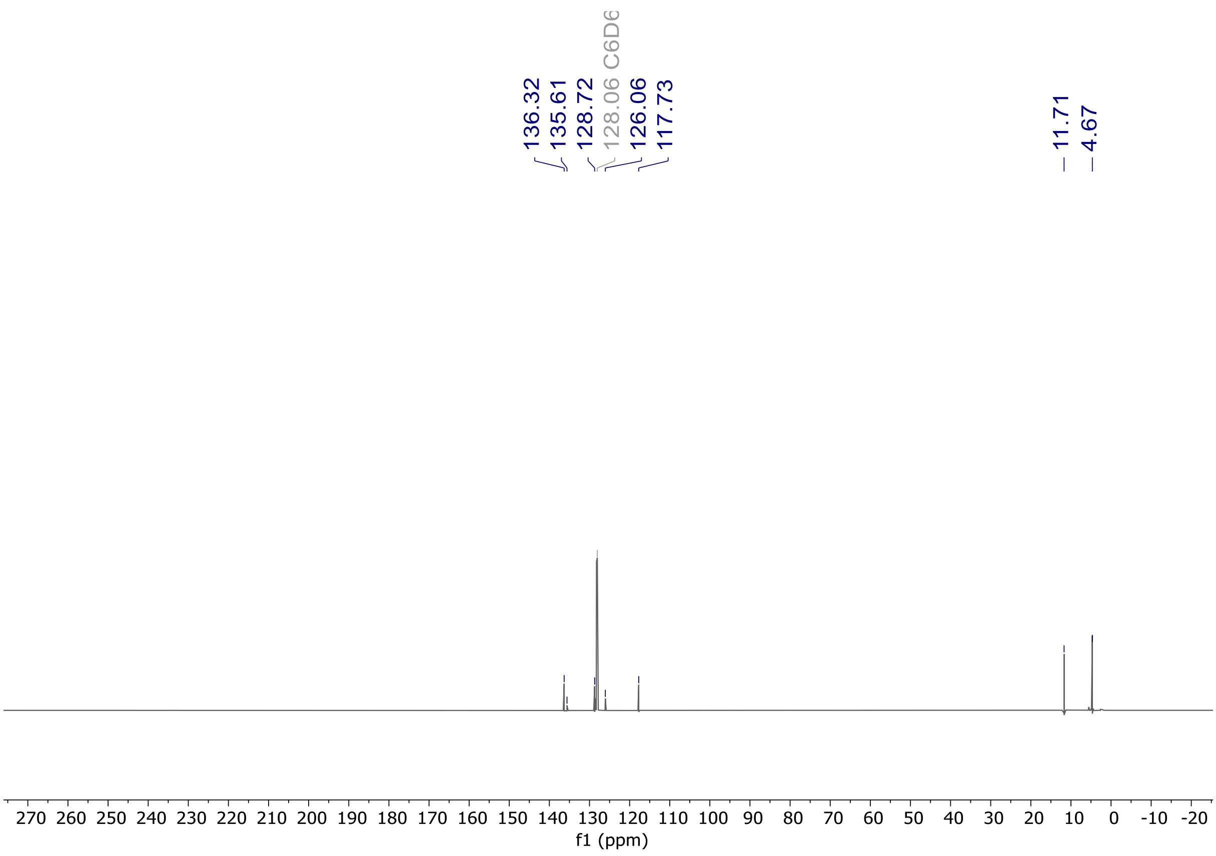

Figure S15: ^{13}C{^{1}H} NMR (126 MHz, C_6D_6) of **5**.

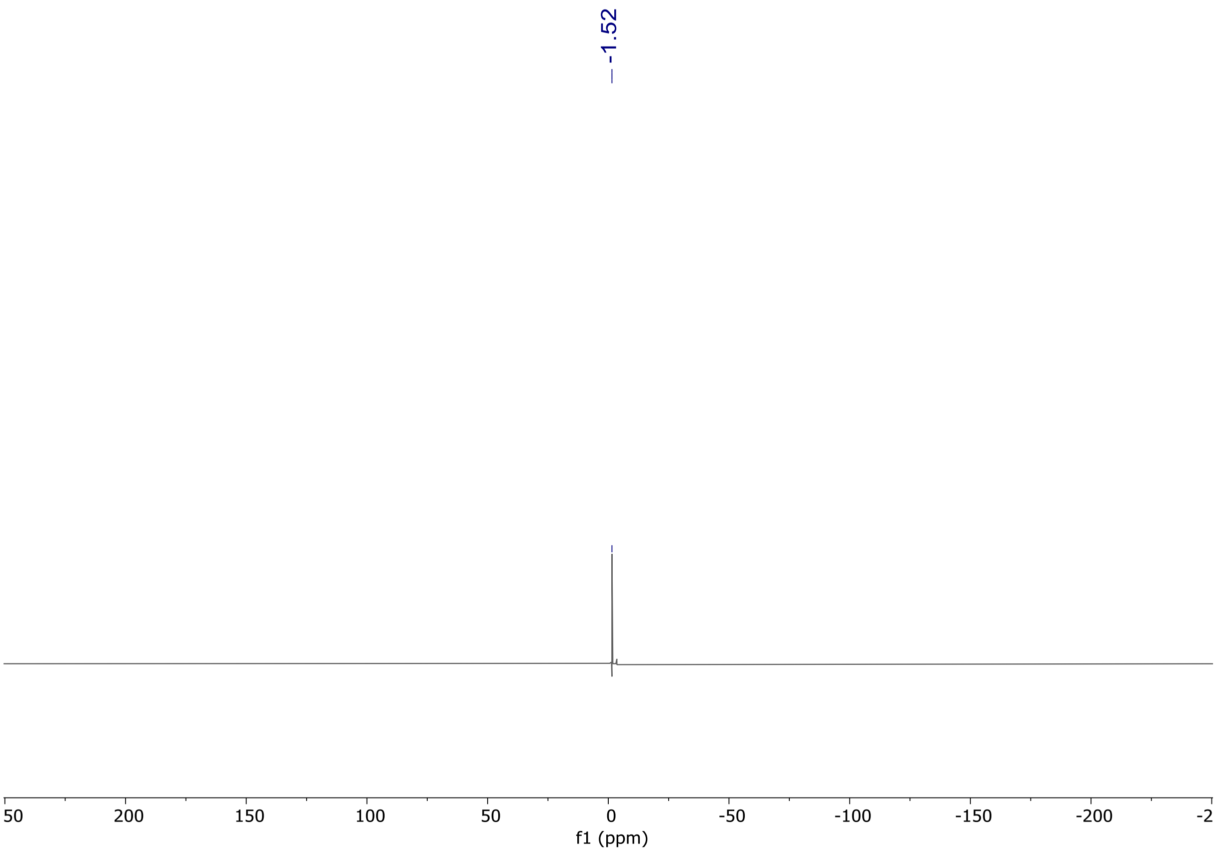

Figure S16: $^{29}Si\{^{1}H\}$ NMR (99 MHz, C_6D_6) of **5**.

3.5 [({N(TMS)2})(Cp*)Al(SePh)] (6)

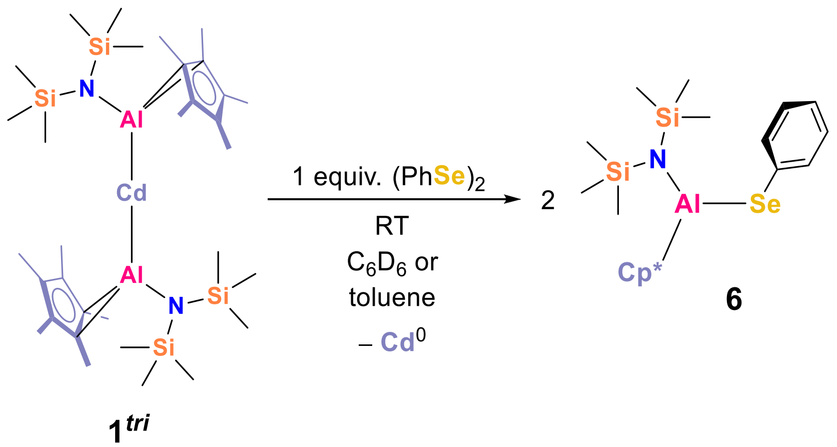

In an ampoule with PTFE valve (FengTecEx), **1**^{tri} was synthesized *in-situ* on a 0.07 mmol scale (based on Cd{N(TMS)2}2) in 3 mL of toluene.[5] Next, diphenyldiselenide (22.8 mg, 0.07 mmol, 1 eq.) was dissolved in 0.5 mL of toluene and added to the ampoule, leading to the formation of a black precipitate. The solvent was removed under reduced pressure and thoroughly dried in vacuo. In the glovebox, 3 mL of *n*-pentane was then added to extract the product followed by filtration through a PTFE syringe filter (d = 13 mm; pore size 0.22 µm). Slow evaporation of the *n*-pentane yields colorless plates of **6** (yield: 68 mg, 97%). *Note*: Even when kept at –30°C in a glovebox integrated freezer, a black coating forms after prolonged storage (> 2 weeks).

<u>*Analytical Data:*</u>

^{1}H NMR (500 MHz, C_6D_6, 298 K) ***δ***: 7.59 (m, 2H, C*H*$_{Ar}$), 6.93 (m, 3H, C*H*$_{Ar}$), 1.95 (s, 15H, –C*H*$_3$ of Cp*), 0.09 (s, 18H, –C*H*$_3$ of HMDS) ppm.

^{13}C{^{1}H} NMR (126 MHz, C_6D_6, 298 K) ***δ***: 138.0 (s, *C*H$_{Ar}$), 129.0 (s, *C*H$_{Ar}$), 126.6 (s, *C*H$_{Ar}$), 118.5 (s, *C*$_q$ of Cp*), 11.8 (s, *C*H$_3$ of Cp*), 4.6 (s, *C*H$_3$ of HMDS) ppm. (One signal (*C*$_q$ of Ar) was not observed.)

^{27}Al NMR (130 MHz, C_6D_6, 298 K) ***δ***: no resonance observed in a spectroscopic range of +300 – -300 ppm.

^{29}Si{^{1}H} NMR (99 MHz, C_6D_6, 298 K) ***δ***: −1.6 (s, *Si* of HMDS) ppm.

^{77}Se{^{1}H} NMR (95 MHz, C_6D_6, 298 K) ***δ***: 9.8 ppm.

Elemental Analysis calc. for $C_{22}H_{38}Al_1N_1Se_1Si_2$ (found) C 55.20 (54.43), H 8.00 (8.18), N 2.93 (2.82)

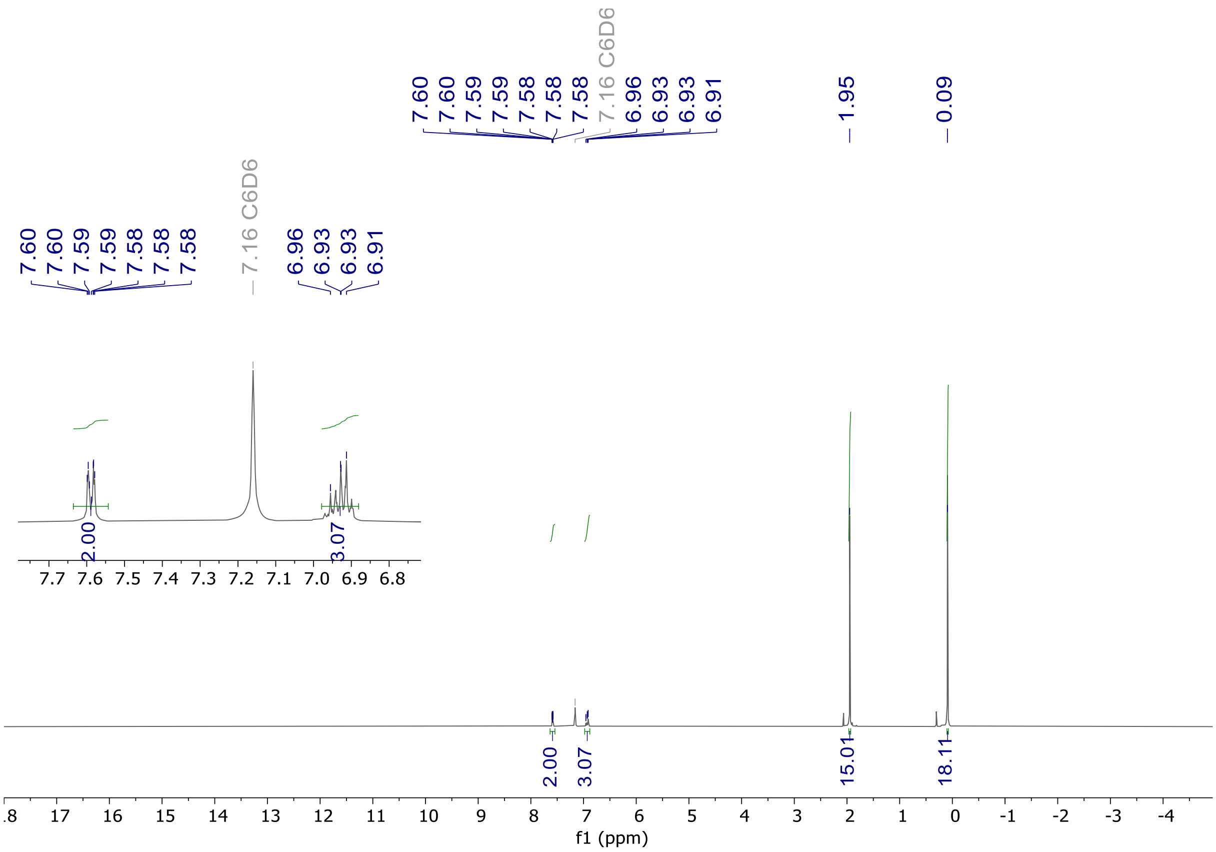

Figure S17: ^{1}H NMR (500 MHz, C_6D_6) of **6**.

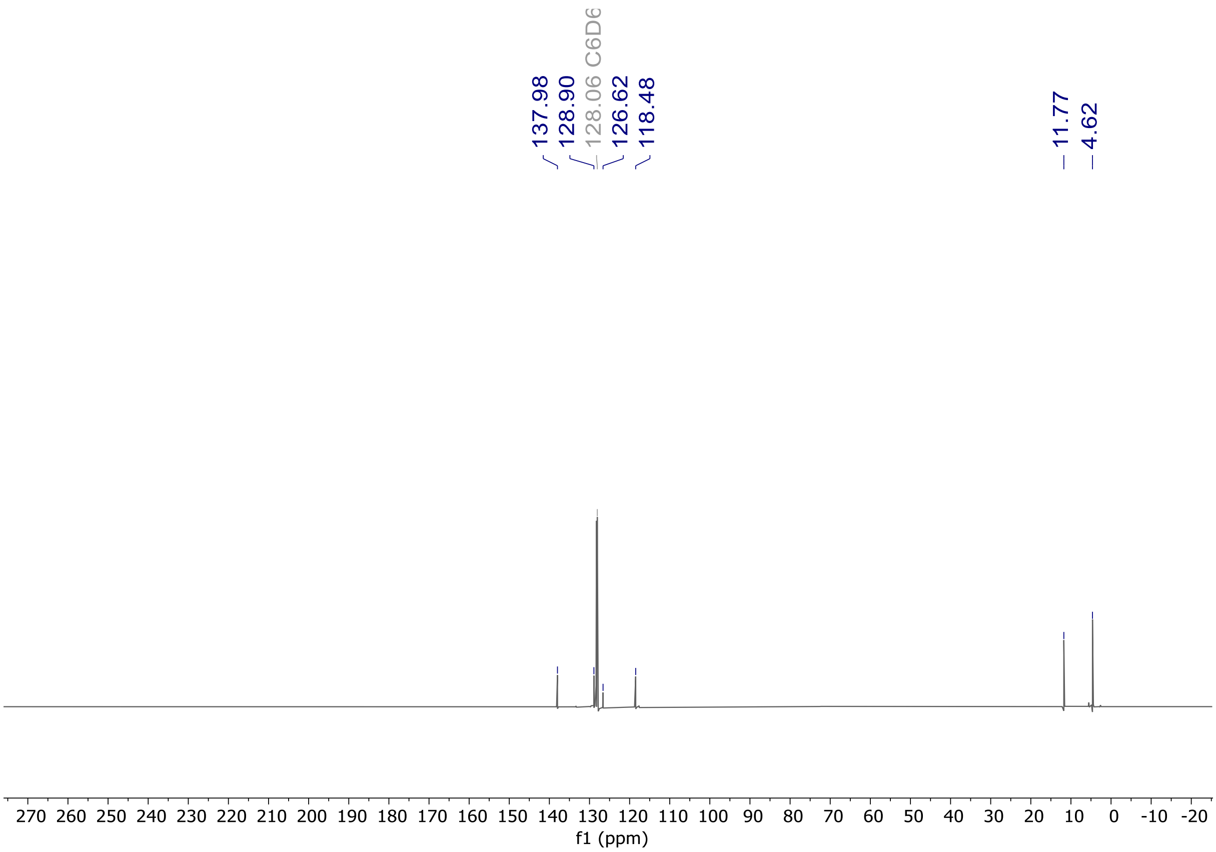

Figure S18: ^{13}C{^{1}H} NMR (126 MHz, C_6D_6) of **6**.

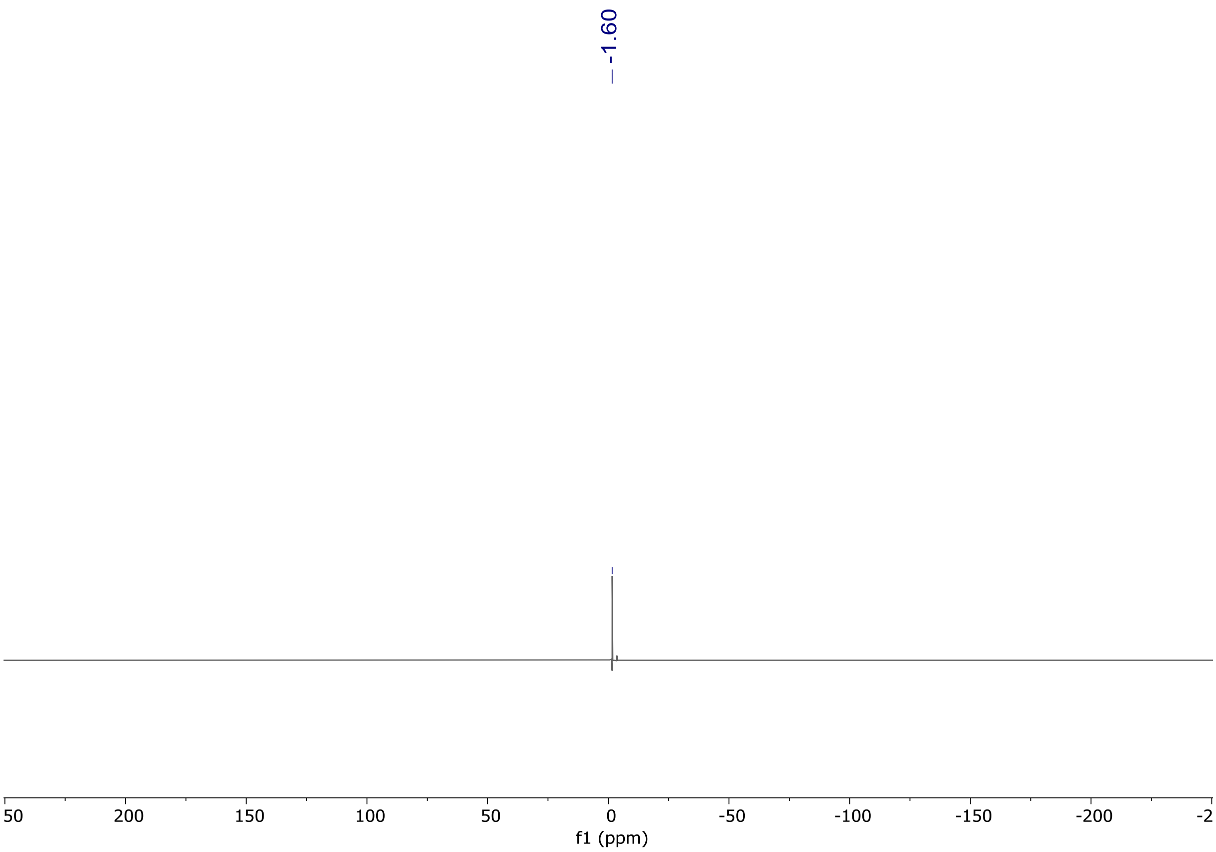

Figure S19: $^{29}Si\{^{1}H\}$ NMR (99 MHz, C_6D_6) of **6**.

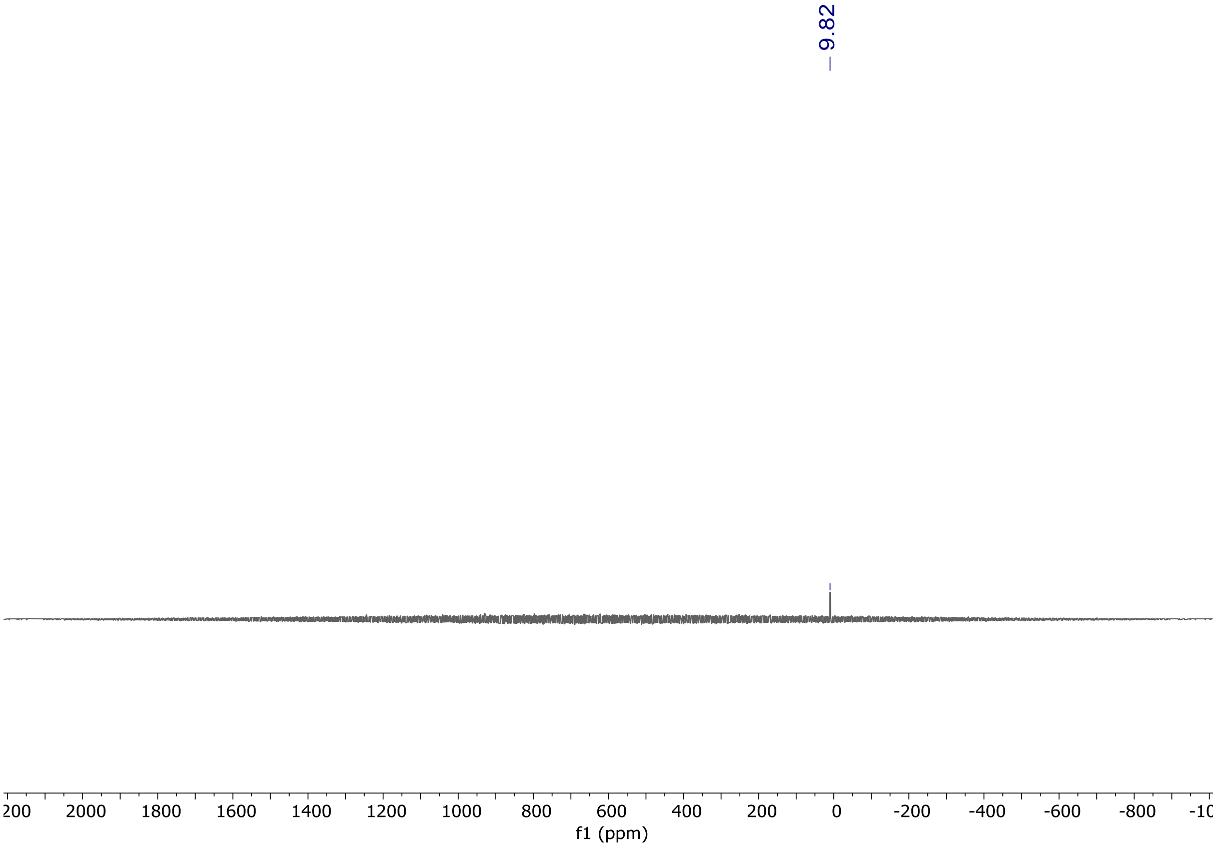

Figure S20: $^{77}Se\{^{1}H\}$ NMR (95 MHz, C_6D_6) of **6**.

3.6 [({N(TMS)$_2$})(Cp*)Al(TePh)] (7)

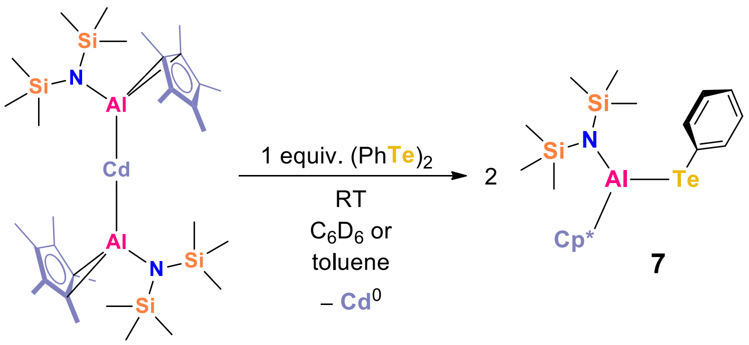

$\mathbf{1}^{tri}$

In an ampoule with PTFE valve (FengTecEx), $\mathbf{1}^{tri}$ was synthesized on a 0.08 mmol scale (based on Cd{N(TMS)$_2$}$_2$) in 3 mL of toluene.[5] Next, diphenylditelluride (32.7 mg, 0.079 mmol, 1 eq.) was dissolved in 0.5 mL of toluene and added to the ampoule, leading to the formation of a black precipitate. The solvent was removed under reduced pressure and thoroughly dried in vacuo. In the glovebox, 4 mL of *n*-pentane was then added to extract the product followed by filtration through a PTFE syringe filter (d = 13 mm; pore size 0.22 µm). Slow evaporation of the *n*-pentane yields colorless plates of **7** (Yield: 79 mg, 95%). *Note*: Prolonged storing causes the compound to turn orange and decompose (less than one week), even when storing the compound at –30 °C in a glovebox integrated freezer.

<u>*Analytical Data:*</u>

^{1}H NMR (500 MHz, C_6D_6, 298 K) δ: 7.81 (m, 2H, CH_{Ar}), 6.99 (m, 1H, CH_{Ar}), 6.84 (m, 2H, CH_{Ar}), 1.91 (s, 15H, –CH_3 of Cp*), 0.12 (s, 18H, –CH_3 of HMDS) ppm.

^{13}C{^{1}H} NMR (126 MHz, C_6D_6, 298 K) δ: 142.5 (s, CH$_{Ar}$), 129.0 (s, CH$_{Ar}$), 127.3 (s, CH$_{Ar}$), 119.4 (s, C_q of Cp*), 106.0 (s, C_q of Ar), 11.9 (s, CH$_3$ of Cp*), 4.7 (s, CH$_3$ of HMDS) ppm.

^{27}Al NMR (130 MHz, C_6D_6, 298 K) δ: 79.2 ($\omega_{1/2}$ = 1800 Hz) ppm.

^{29}Si{^{1}H} NMR (99 MHz, C_6D_6, 298 K) δ: −1.6 (s, *Si* of HMDS) ppm.

^{125}Te NMR (158 MHz, C_6D_6, 298 K) δ: −198 ppm ($\omega_{1/2}$ = 250 Hz). (Note that this resonance was only observed after pulsing for 48 hours)

Elemental Analysis calc. for $C_{22}H_{38}Al_1N_1Si_2Te$ (found) C 50.11 (50.23), H 7.26 (7.39), N 2.66 (2.60)

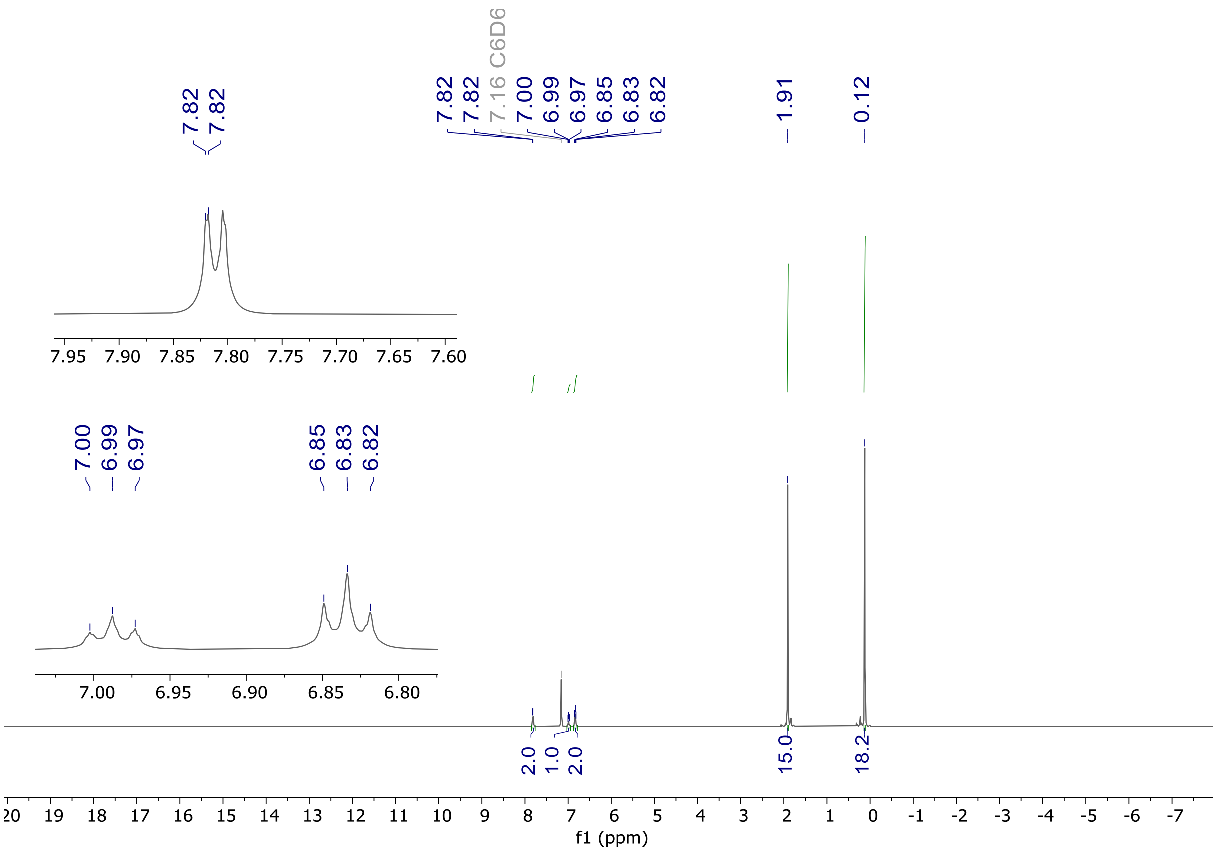

Figure S21: ^{1}H NMR (500 MHz, C_6D_6) of **7**.

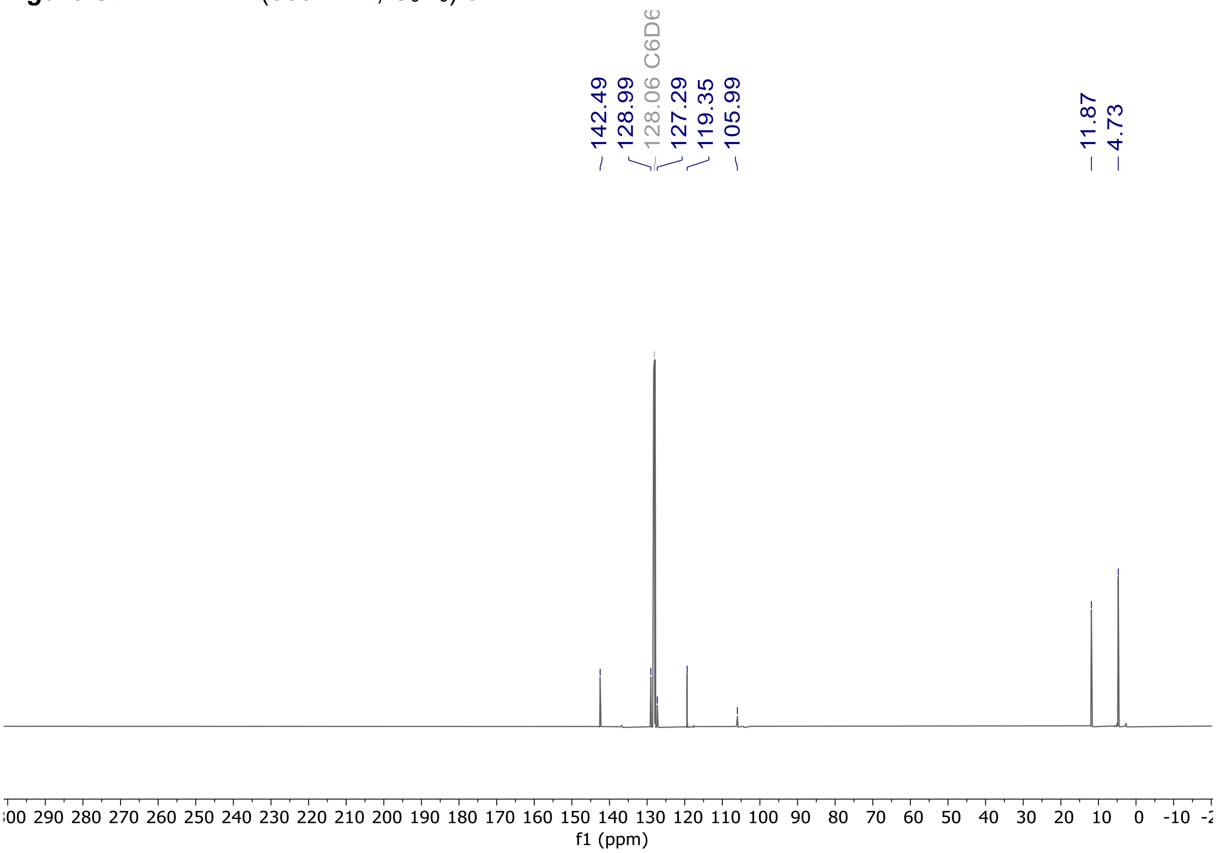

Figure S22: $^{13}C\{^{1}H\}$ NMR (126 MHz, C_6D_6) of **7**.

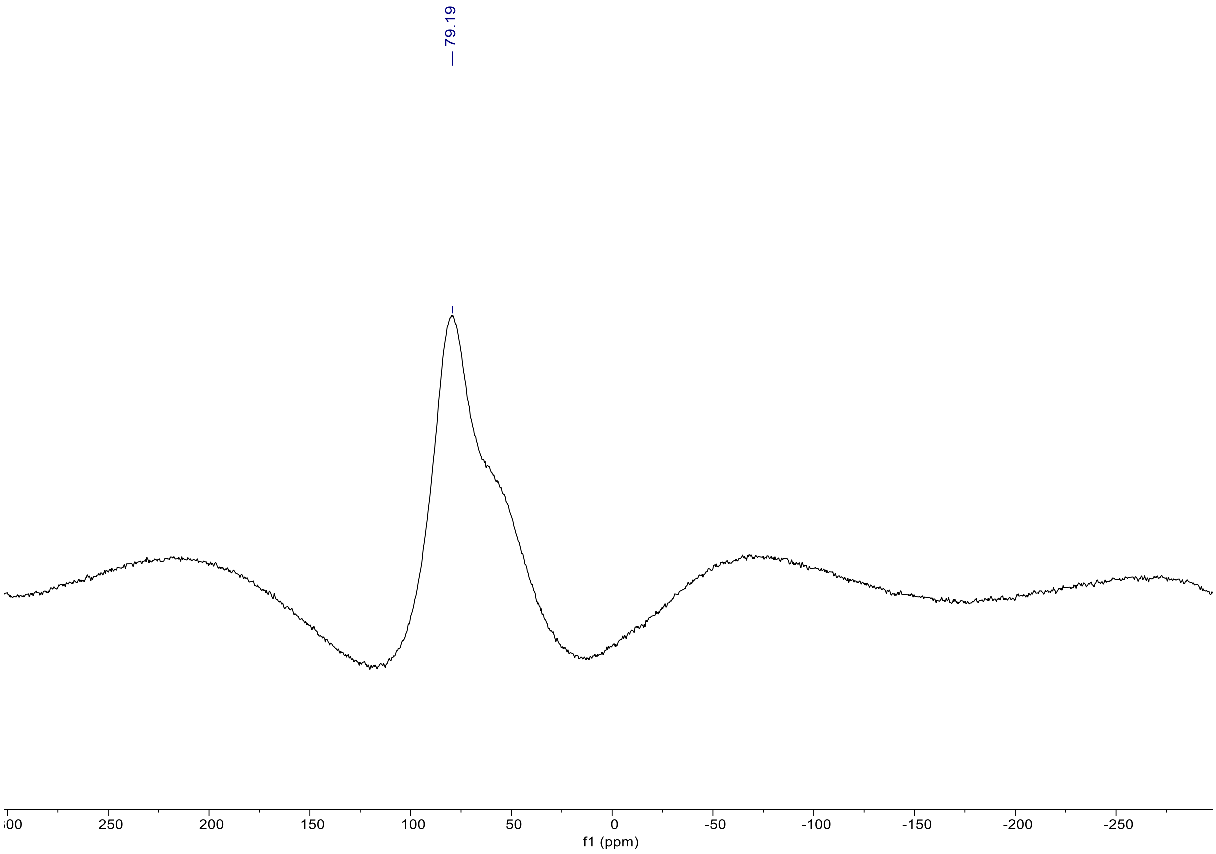

Figure S23: ^{27}Al NMR (130 MHz, C_6D_6) of **7**. 50 ppm: probe head.

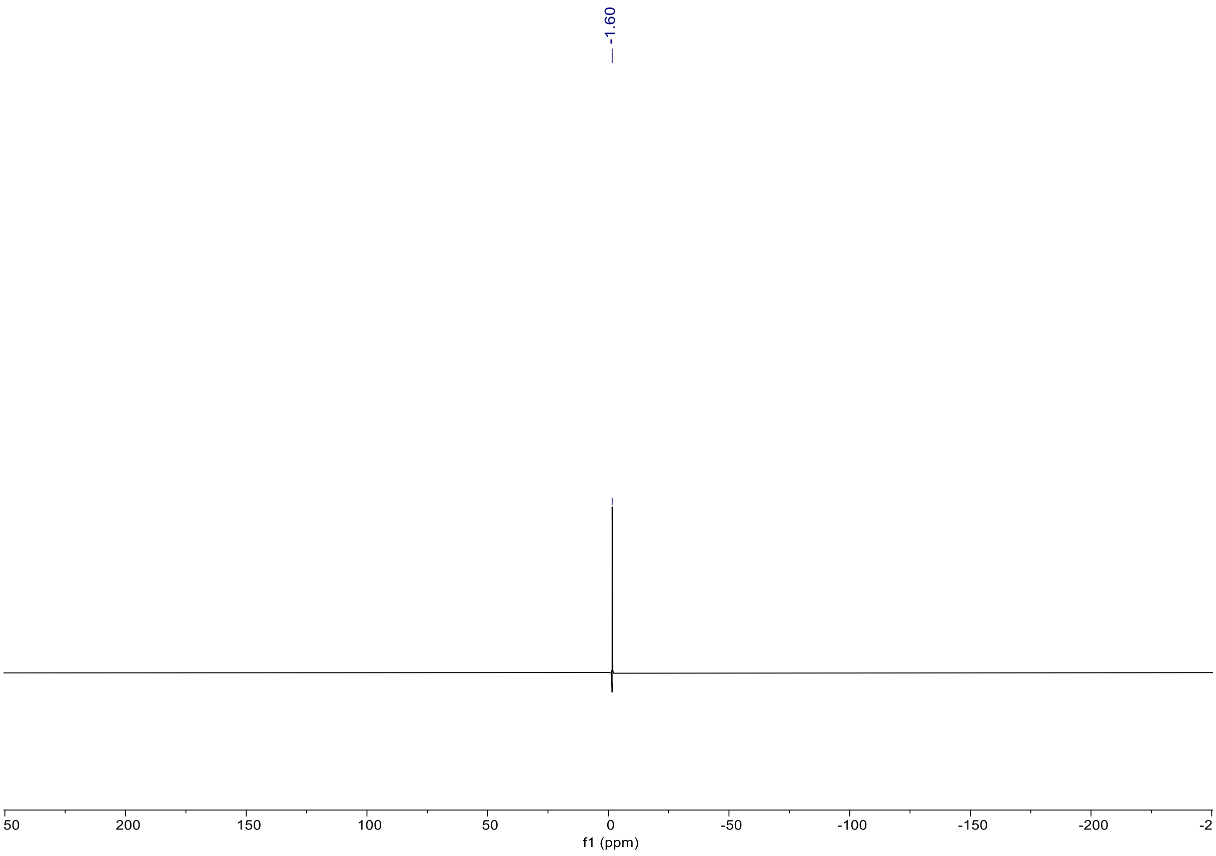

Figure S24: ^{29}Si{^{1}H} NMR (99 MHz, C_6D_6) of **7**.

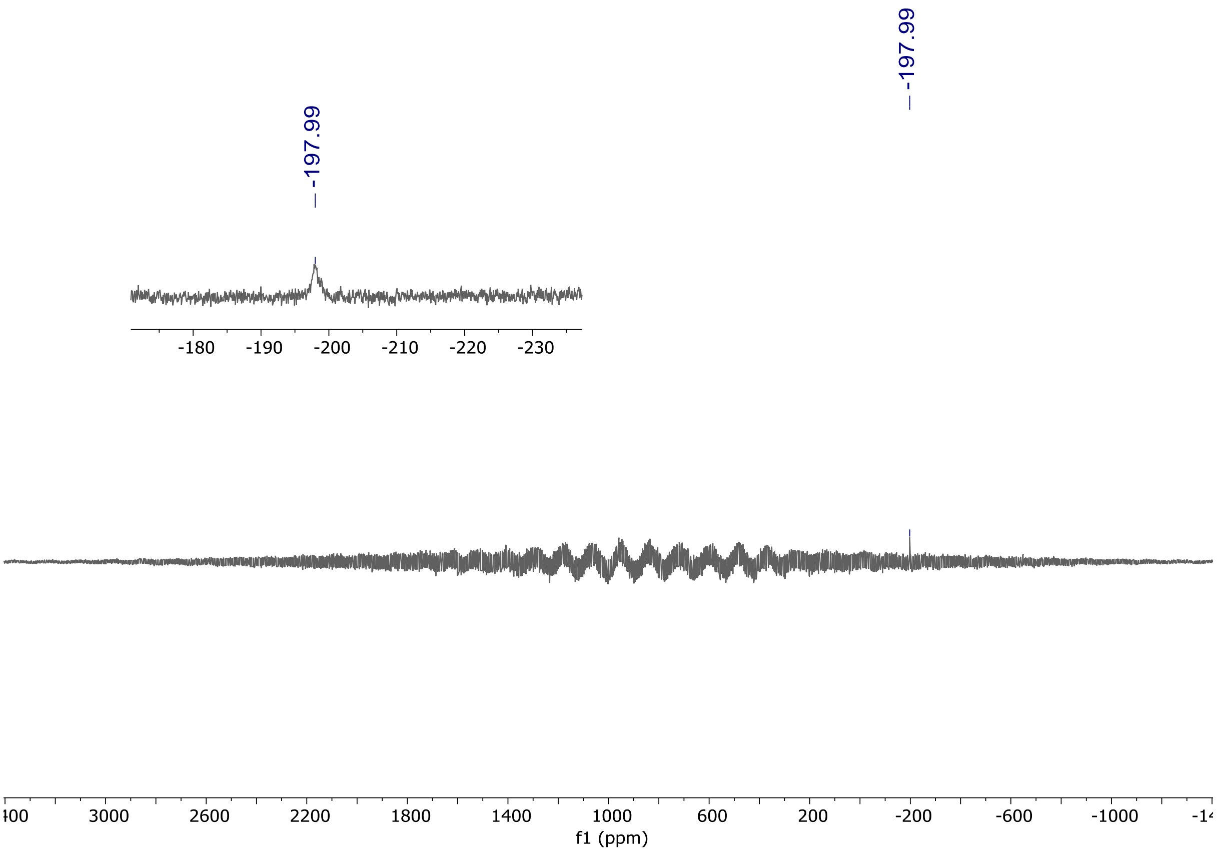

Figure S25: ^{125}Te NMR (158 MHz, C_6D_6) of **7**.

3.7 [({N(TMS)$_2$})(Cp*)Al(O–CPh$_2$)]$^{\cdot}$ (8)

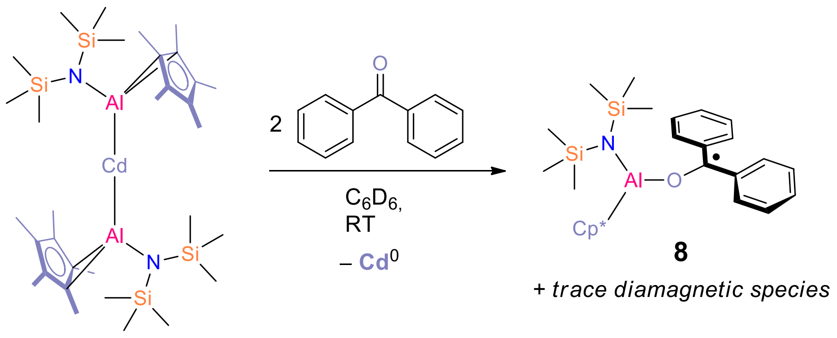

1^{tri}

In an ampoule with PTFE valve (FengTecEx), **1^{tri}** was synthesized on a 0.06 mmol scale (based on Cd{N(TMS)$_2$}$_2$) in 4 mL of toluene.[5] Next, benzophenone (21.7 mg, 0.119 mmol, 2 eq.) was dissolved in 1 mL of toluene and added to the ampoule, leading to the formation of a magenta-colored solution and black precipitates (see Figure S26a). The solvent was removed under reduced pressure and thoroughly dried in vacuo. In the glovebox, 3 mL of *n*-pentane was then added to extract the product followed by filtration through a PTFE syringe filter (d = 13 mm; pore size 0.22 µm), leading to a clear magenta-colored solution (Figure S26b). Storing the solution in the glovebox integrated freezer causes quick (ca. 3-4 hours) decolorization of the solution and formation of black precipitates (see Figure S26c). Conversion of **1^{tri}** is >95% when freshly made.

<u>*Analytical Data:*</u>

UV-VIS (*o*DFB): visible absorption ranging from 450–600 nm: (λ_{max} = 554 nm).

EPR: Please see section 6 for full details on the EPR spectroscopic characterization.

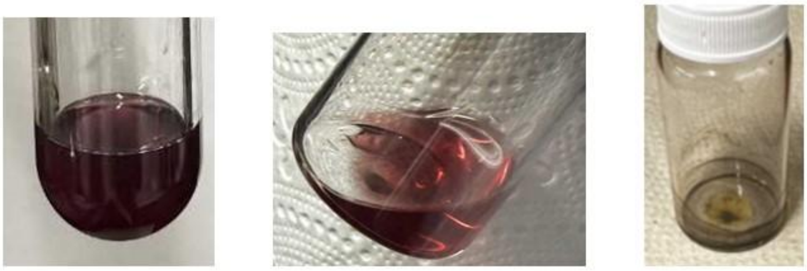

(a) (b) (c)

Figure S26: (a) Fresh solution of **8** after addition of benzophenone to **1^{tri}**. (b) Fresh solution of **8** after filtration. (c) Black precipitates after attempts to isolate compound **8**.

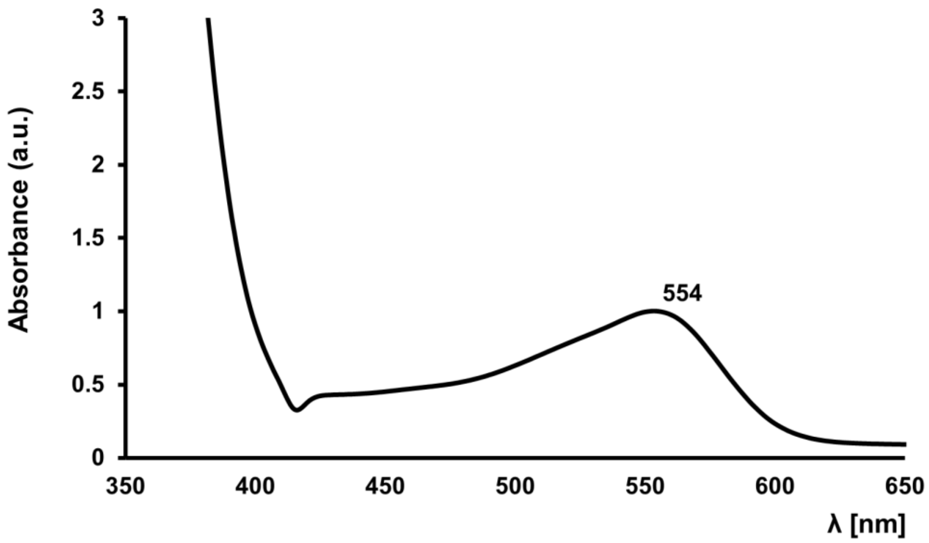

Figure S27: UV–VIS absorption spectrum (350-650 nm) of a freshly prepared solution of **8** in *o*DFB. The dip at around 400 nm does most likely originate from a device-specific switching of light sources during the experiment.

3.8 [({N(TMS)$_2$})(Cp*)Al(O–CPy$_2$)]$^{\cdot}$ (9)

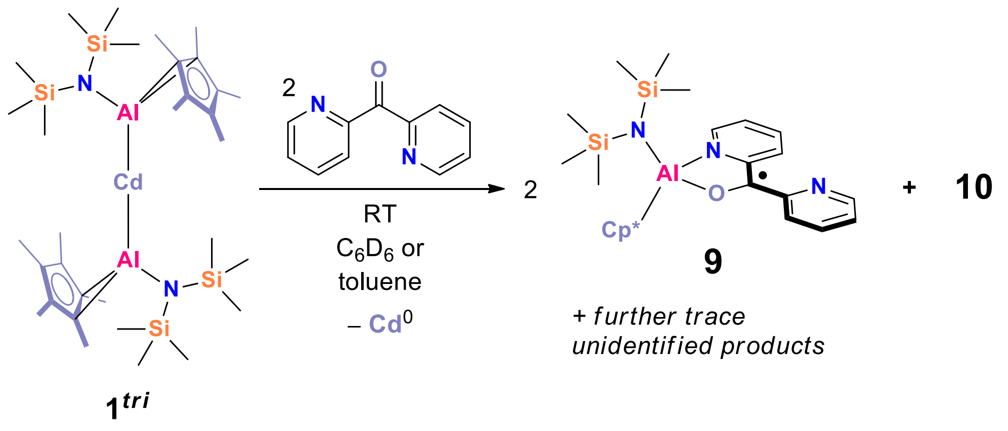

In an ampoule with PTFE valve (FengTecEx), **1**^{tri} was synthesized on a 0.07 mmol scale (based on Cd{N(TMS)$_2$}$_2$) in 4 mL of toluene.[5] Next, di(2-pyridyl) ketone (26 mg, 0.14 mmol, 2 eq.) was dissolved in 1 mL of toluene and added to the ampoule, leading to the formation of an intense dark purple solution and black precipitates (see Figure S28a). The solvent was removed under reduced pressure and thoroughly dried in vacuo. In the glovebox, 5 mL of *n*-pentane was then added to extract the product followed by filtration through a PTFE syringe filter (d = 13 mm; pore size 0.22 µm) (see Figure S28b). Slow evaporation of the *n*-pentane yields a dark purple powder of (crude) **9** (Yield: 47 mg, 65%, containing *trace* amounts of **10**).

Analytical Data:

EPR: Please see section 6 for full details on the EPR spectroscopic characterization.

UV-VIS (*o*DFB): visible absorption ranging from 400–700 nm: (λ_{max} = 468 nm).

LIFDI$^+$ MS:

596.2596 (calc. 596.2598) [M]$^+$ (10);
371.1422 (calc. 371.1424) [M–Cp*]$^+$ (100)

Elemental Analysis calc. for $C_{22}H_{38}Al_1NS_1Si_2$ (found) C 63.99 (63.43), H 8.15 (8.45), N 8.29 (6.45)* *We provide the best value obtained after several measurements of different freshly synthesized batches.

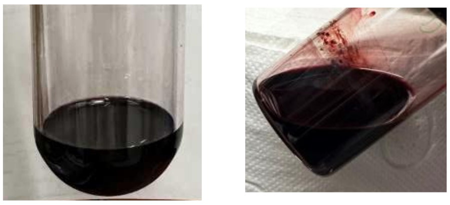

(a) (b)

Figure S28: (a) Solution of **9** after addition of di(2-pyridyl) ketone to **1**^{tri}. (b) Solution of **9** after filtration.

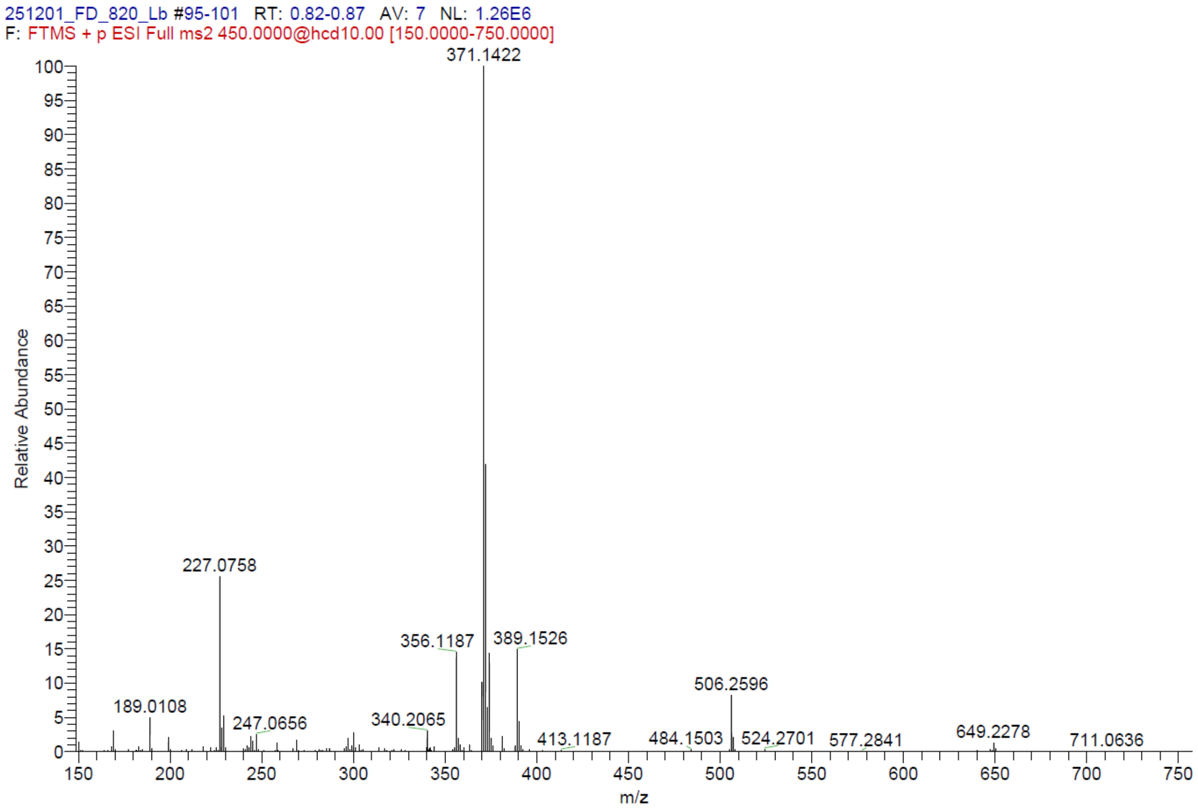

Figure S29: FD MS spectrum of a dissolved sample of **9**.

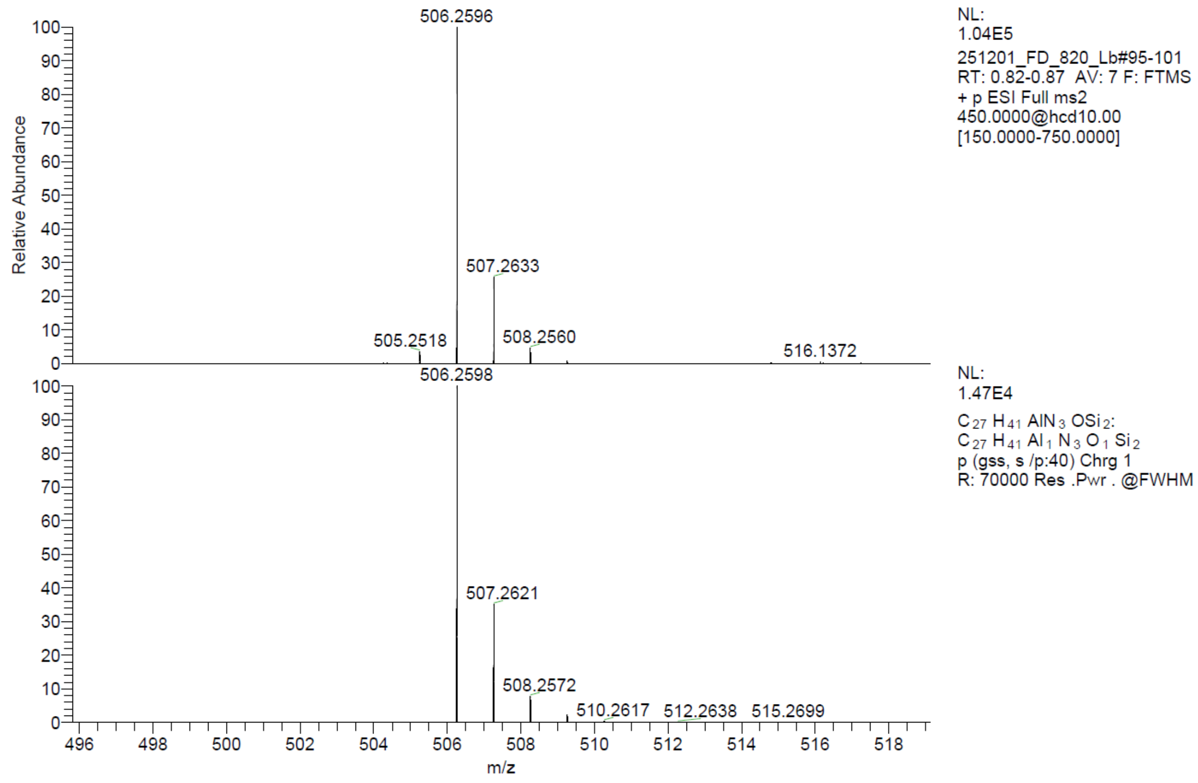

Figure S30: HR-FD MS spectrum of a dissolved sample of **9** indicating $[M]^+$.

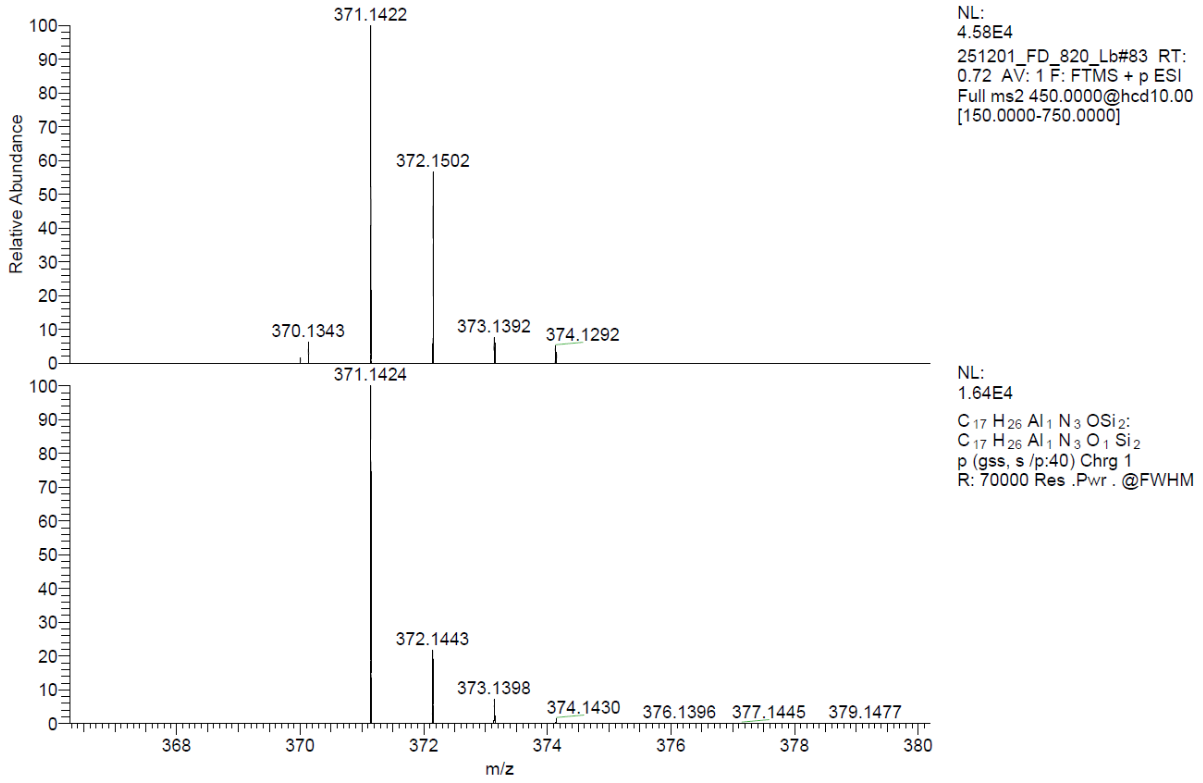

Figure S31: HR-FD MS spectrum of a dissolved sample of **9** indicating $[M\text{-}Cp^*]^+$.

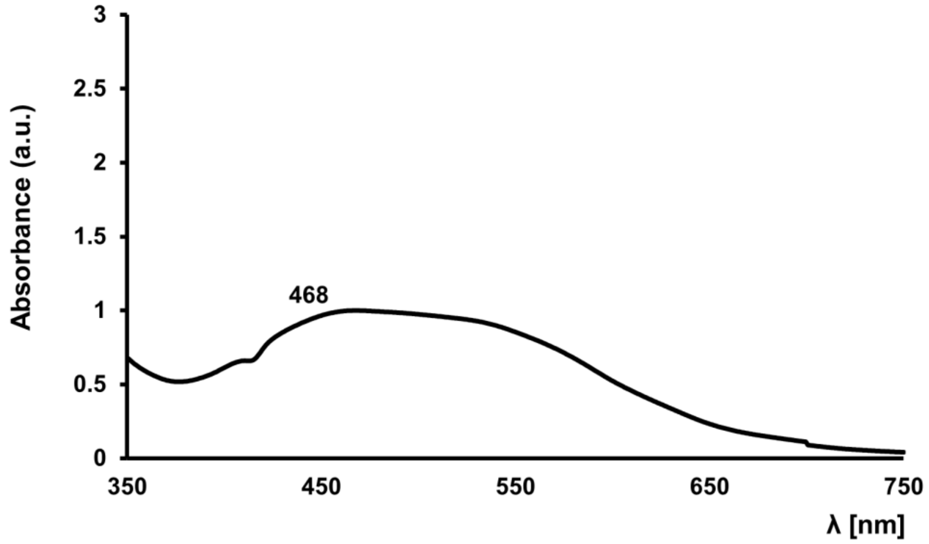

Figure S32: UV–VIS absorption spectrum (350-750 nm) of a freshly prepared solution of **9** in *o*DFB. The dip at around 400 nm does most likely originate from a device-specific switching of light sources during the experiment.

4 NMR studies: Reactivity of 1^{bi}

4.1 Formation of [({N(TMS)$_2$})(Cp*)Al(SPh)] and Cd(SPh)$_2$ starting from 1^{bi}

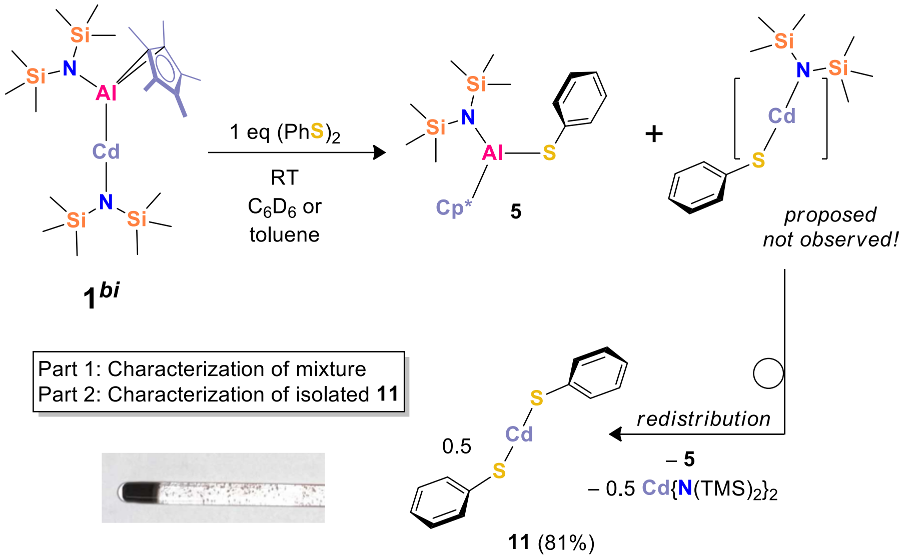

Part 1: In a J. Young NMR tube **1***bi* was synthesized on a 0.05 mmol scale on 0.7 mL of C_6D_6.[5] Next, diphenyl disulfide (10.8 mg, 0.05 mmol, 1 eq.), dissolved in 0.5 mL of C_6D_6, was added to the solution. The instantaneous formation of a dark precipitate was observed. The supernatant was subjected to NMR spectroscopic analysis.

Part 2: The remaining solution was carefully decanted off, and 1.5 ml of *n*-pentane was added. The mixture was sonicated for 10 minutes, before carefully decanting off the solution again. This step was repeated three times in total. The solid was suspended in ca. 0.6 mL *n*-pentane and transferred to a vial inside of the glovebox. Slow evaporation of *n*-pentane affords **11** as a dark beige powder (6.7 mg, 81%). NMR spectra were recorded after dissolving **11** in pyridine-d_5.

<u>*Analytical Data (Part 1):*</u>

^{1}H NMR (500 MHz, C_6D_6, 298 K) ***δ***: 7.44 (m, 2H, C*H*$_{Ar}$), 6.95 (m, 3H, C*H*$_{Ar}$), 1.97 (s, 15H, C*H*$_3$ of Cp*), 0.08 (s, 18H, –C*H*$_3$ of HMDS) ppm.

^{13}C{^{1}H} NMR (126 MHz, C_6D_6, 298 K) ***δ***: 136.3 (s, *C*H$_{Ar}$), 135.6 (s, *C*H$_{Ar}$), 128.7 (s, *C*H$_{Ar}$), 126.06 (s, *C*H$_{Ar}$) 117.7 (s, *C*$_q$ of Cp*), 11.7 (s, *C*H$_3$ of Cp*), 4.7 (s, *C*H$_3$ of HMDS) ppm.

^{27}Al NMR (130 MHz, C_6D_6, 298 K) ***δ***: no resonance observed in a spectroscopic range of +300 – -300 ppm.

^{29}Si{^{1}H} NMR (99 MHz, C_6D_6, 298 K) ***δ***: −1.5 (s, *Si* of HMDS) ppm.

Analytical Data (Part 2):

^{1}H NMR (500 MHz, Pyridine-d_5, 298 K) δ: 7.95 (m, 4H, CH_{Ar}), 7.07 (m, 4H, CH_{Ar}), 6.95 (m, 2H, CH_{Ar}) ppm.

^{13}C{^{1}H} NMR (126 MHz, Pyridine-d_5, 298 K) δ: 145.0 (s, C_q of CH$_{Ar}$), 134.5 (s, CH_{Ar}), 128.92 (s, CH_{Ar}), 123.1 (s, CH_{Ar}) ppm.

^{113}Cd NMR (111 MHz, Pyridine-d_5, 298 K): (111 MHz, Pyridine-d_5, 298 K): 427 ppm.

NMR spectra (Part 1)

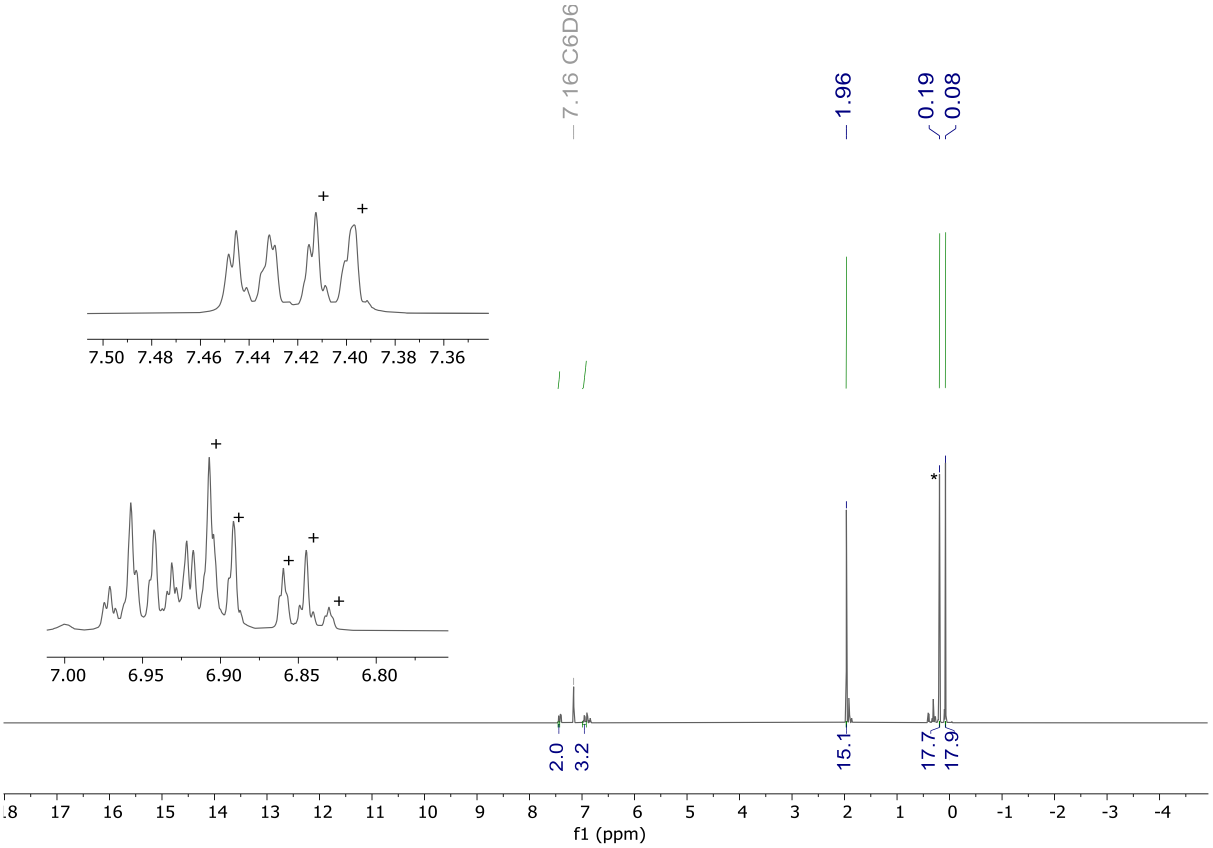

Figure S33: ^{1}H NMR (500 MHz, C_6D_6) of **5**. Trace amounts of excess Ph_2S_2 are marked with a +. Cd{N(TMS)$_2$}$_2$ is marked with *.

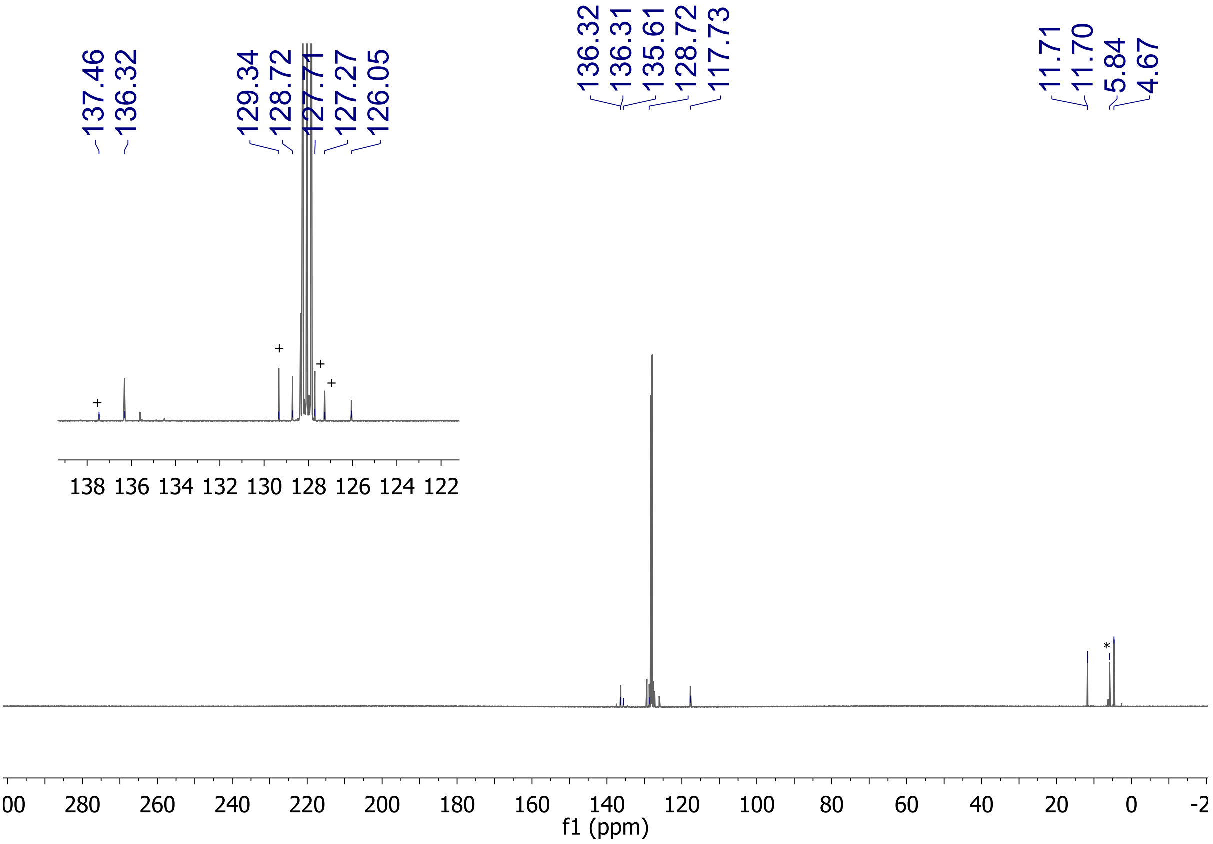

Figure S34: $^{13}C\{^{1}H\}$ NMR (125 MHz, C_6D_6) of **5**. Trace amounts of excess Ph_2S_2 are marked with a +. Cd{N(TMS)$_2$}$_2$ is marked with a *.

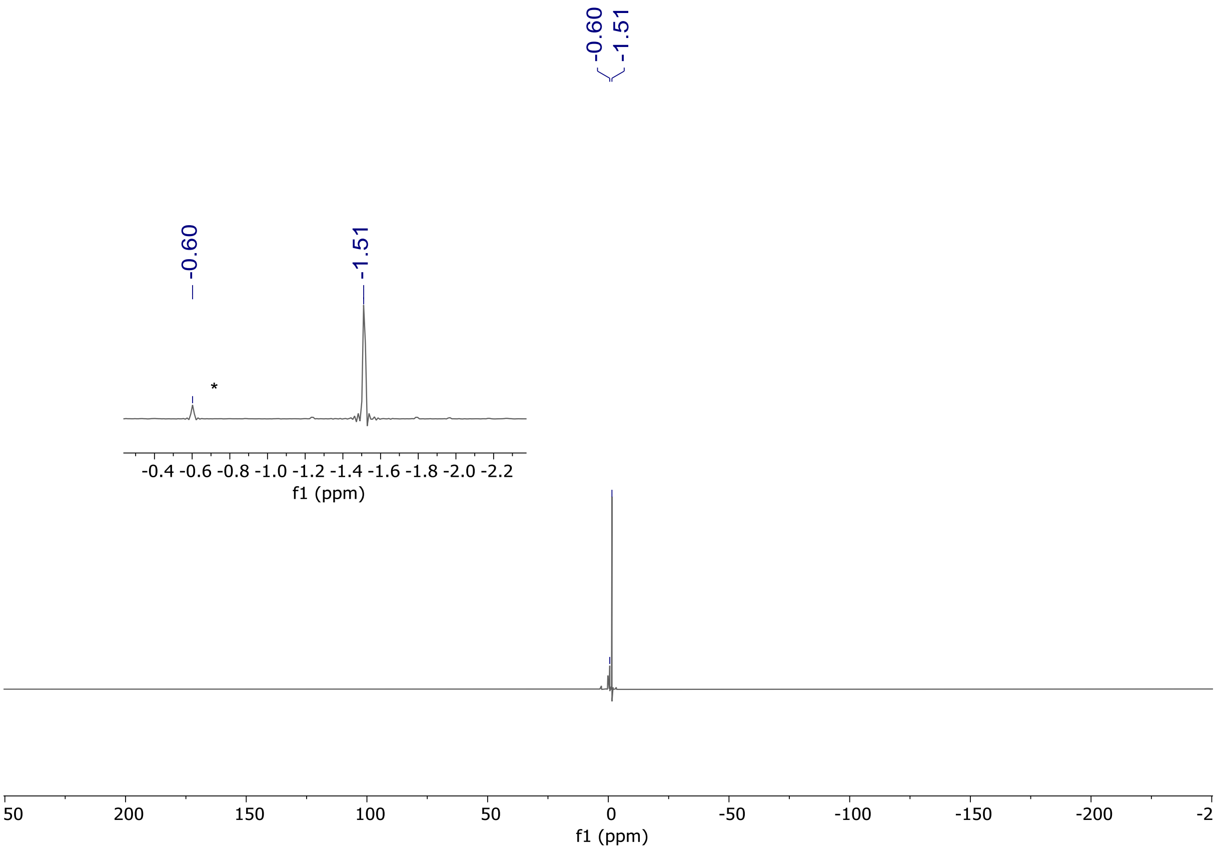

Figure S35: $^{29}Si\{^1H\}$ NMR (99 MHz, C_6D_6) of **5**. $Cd\{N(TMS)_2\}_2$ is marked with a *.

NMR spectra (Part 2)

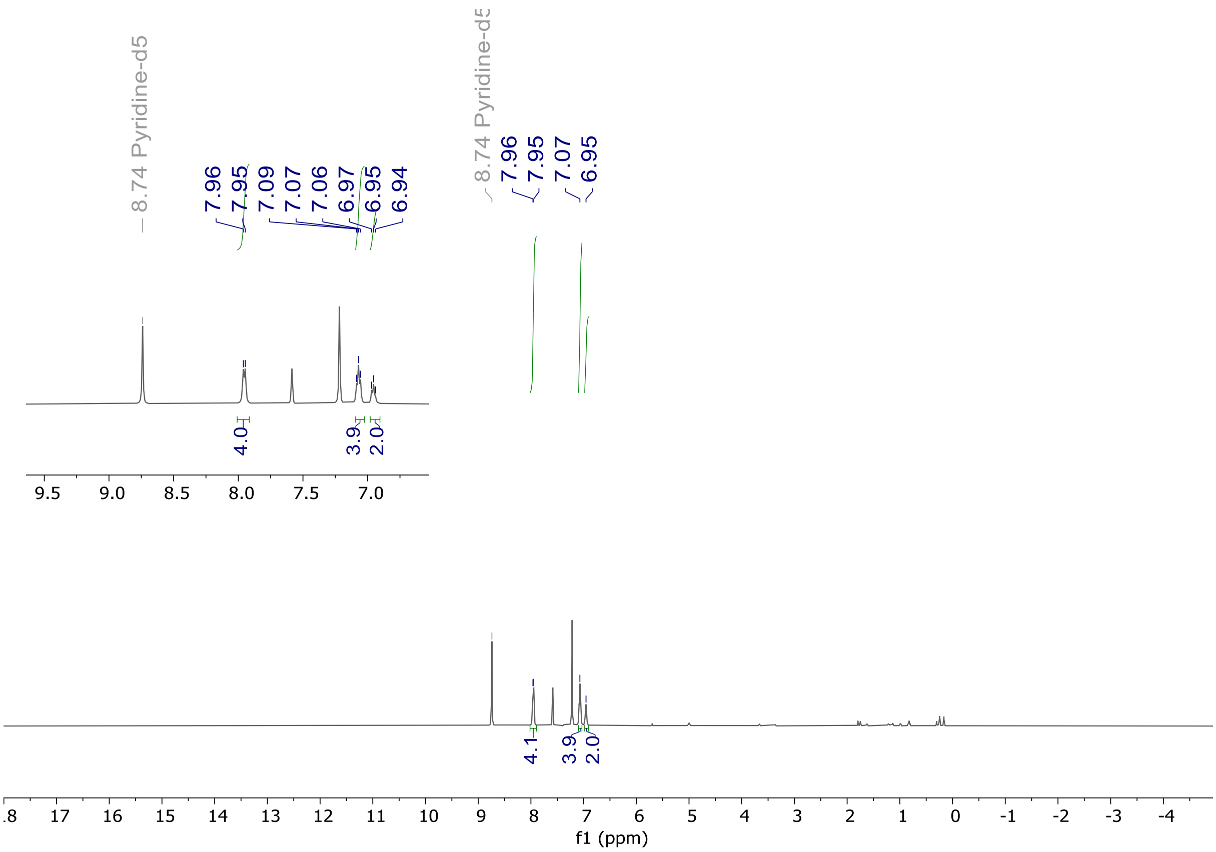

Figure S36: ^{1}H NMR (500 MHz, Pyridine-d_5) of **11**.

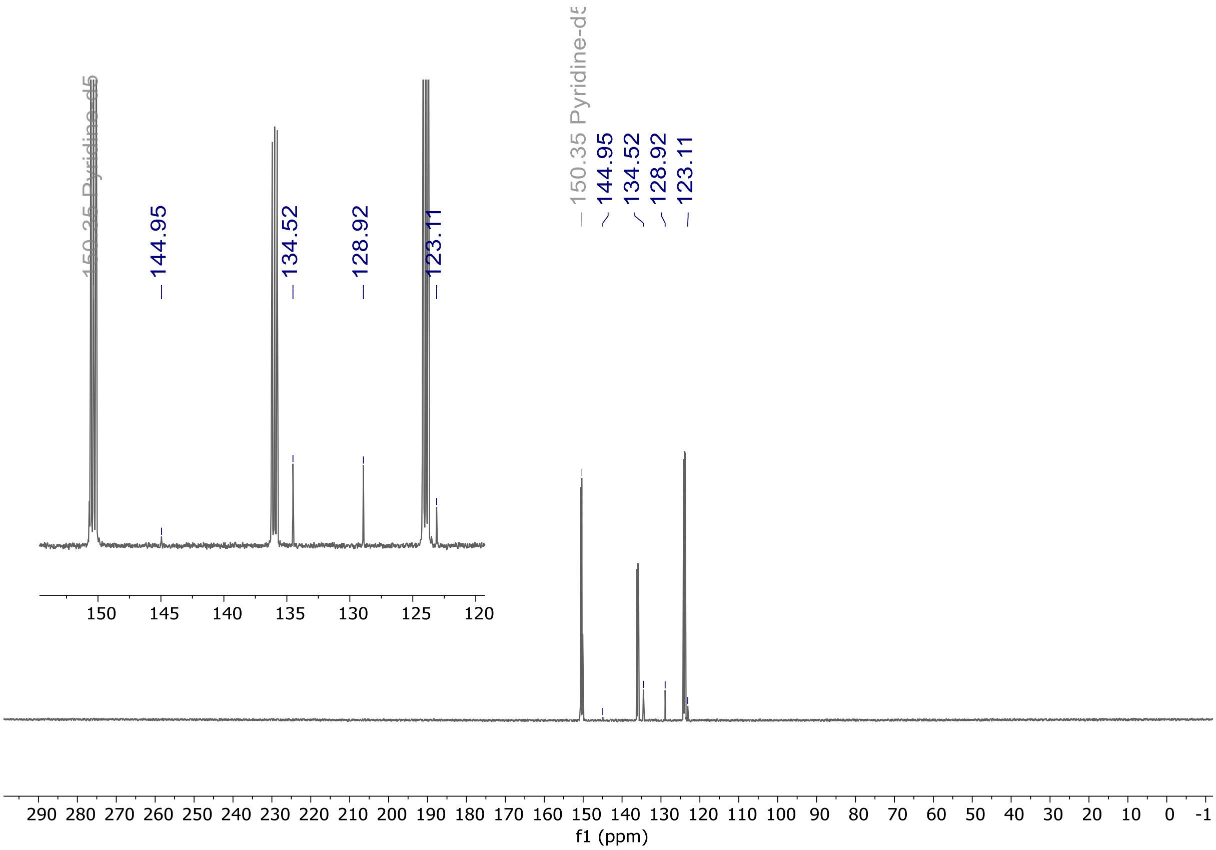

Figure S37: $^{13}C\{^{1}H\}$ NMR (126 MHz, Pyridine-d_5) of **11**.

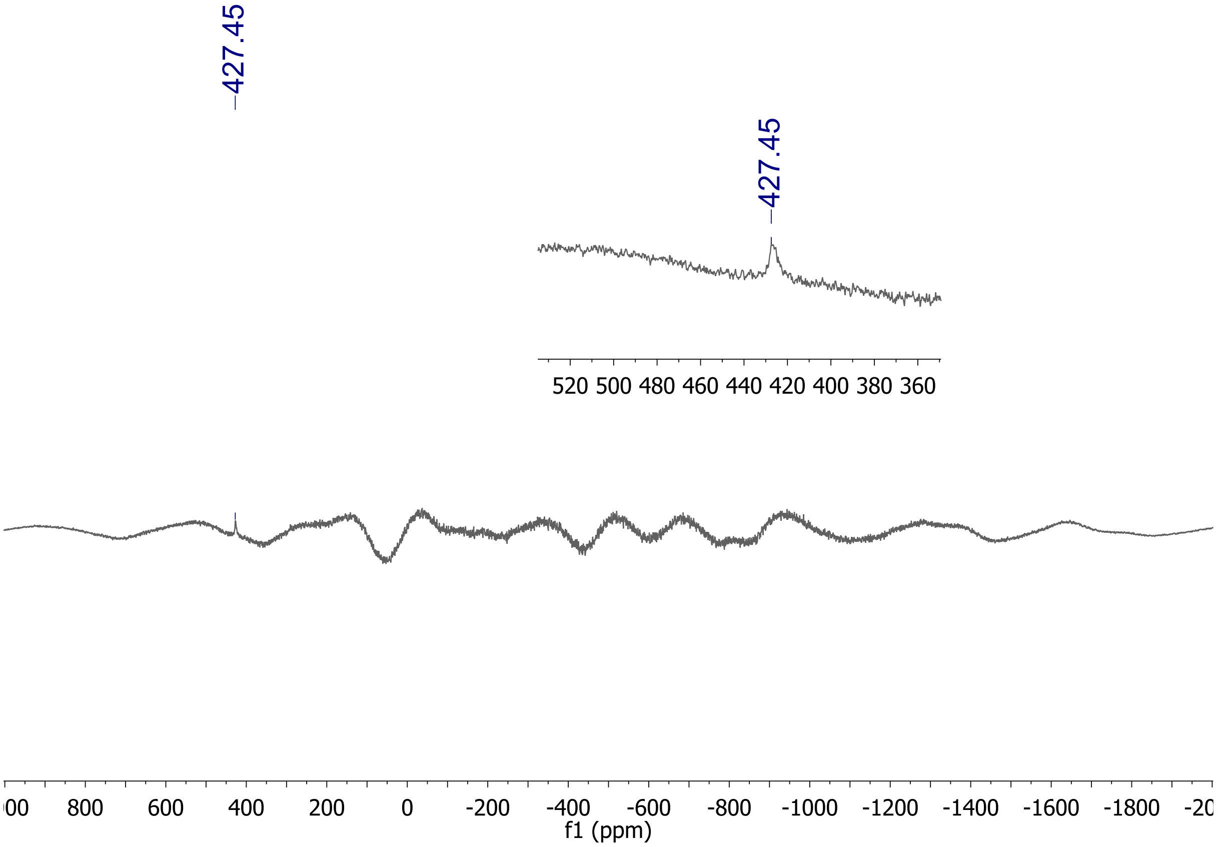

Figure S38: ^{113}Cd NMR (111 MHz, Pyridine-d_5) of **11**.

4.2 Formation of [({N(TMS)$_2$})(Cp*)Al(SePh)] and Cd(SePh)$_2$ starting from 1^{bi}

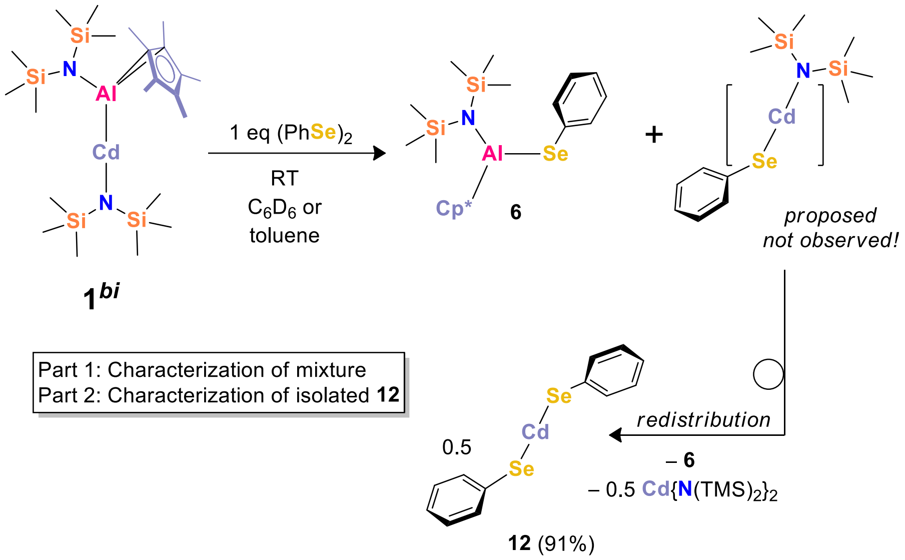

Part 1: In an J. Young NMR tube **1*^{bi}*** was synthesized on a 0.04 mmol scale in 1 mL of C_6D_6.[5] Next, diphenyl diselenide (12.5 mg, 0.04 mmol, 1 eq.), dissolved in 0.2 mL of C_6D_6, was added to the solution. The instantaneous formation of a grey precipitate was observed. The supernatant was subjected to NMR spectroscopic analysis.

Part 2: The remaining solution was carefully decanted off, and 1.5 mL of *n*-pentane was added. The mixture was sonicated for 10 minutes, before decanting off the solution again. This step was repeated three times in total. The solid was suspended in ca. 0.6 ml *n*-pentane and transferred to a vial inside of the glovebox. Slow evaporation of *n*-pentane affords **12** as a pale grey powder (9.1 mg, 91%). NMR spectra were recorded after dissolving **12** in pyridine-d_5.

Analytical Data (Part 1):

^{1}H NMR (500 MHz, C_6D_6, 298 K) ***δ***: 7.59 (m, 2H, C*H*$_{Ar}$), 6.93 (m, 3H, C*H*$_{Ar}$), 1.95 (s, 15H, C*H*$_3$ of Cp*), 0.09 (s, 18H, –C*H*$_3$ of HMDS) ppm.

^{13}C{^{1}H} NMR (126 MHz, C_6D_6, 298 K) ***δ***: 137.9 (s, *C*H$_{Ar}$), 128.9 (s, *C*H$_{Ar}$), 126.6 (s, *C*H$_{Ar}$) 118.5 (s, *C*$_q$ of Cp*), 11.8 (s, *C*H$_3$ of Cp*), 4.6 (s, *C*H$_3$ of HMDS) ppm. One signal of the phenyl group was not detected.

^{27}Al NMR (130 MHz, C_6D_6, 298 K) ***δ***: no resonance observed in a spectroscopic range of +300 – -300 ppm.

^{29}Si{^{1}H} NMR (99 MHz, C_6D_6, 298 K) ***δ***: −1.6 (s, *Si* of HMDS) ppm.

^{77}Se{^{1}H} NMR (95.31 MHz, C_6D_6, 298 K): 9.8 ppm.

Analytical Data (Part 2):

^{1}H NMR (500 MHz, Pyridin-d_5, 298 K) δ: 8.05 (m, 4H, CH_{Ar}), 7.04 (m, 6H, CH_{Ar}) ppm.

^{13}C{^{1}H} NMR (126 MHz, Pyridine-d_5, 298 K) δ: 137.03 (s, CH$_{Ar}$), 133.6 (s, CH$_{Ar}$), 129.2 (s, CH$_{Ar}$), 124.5 (s, CH$_{Ar}$) ppm.

^{77}Se{^{1}H} NMR (95.31 MHz, Pyridine-d_5, 298 K): −31.2 ppm.

^{113}Cd NMR (111 MHz, Pyridine-d_5, 298 K): (111 MHz, Pyridine-d_5, 298 K): 457 ppm.

NMR spectra (Part 1)

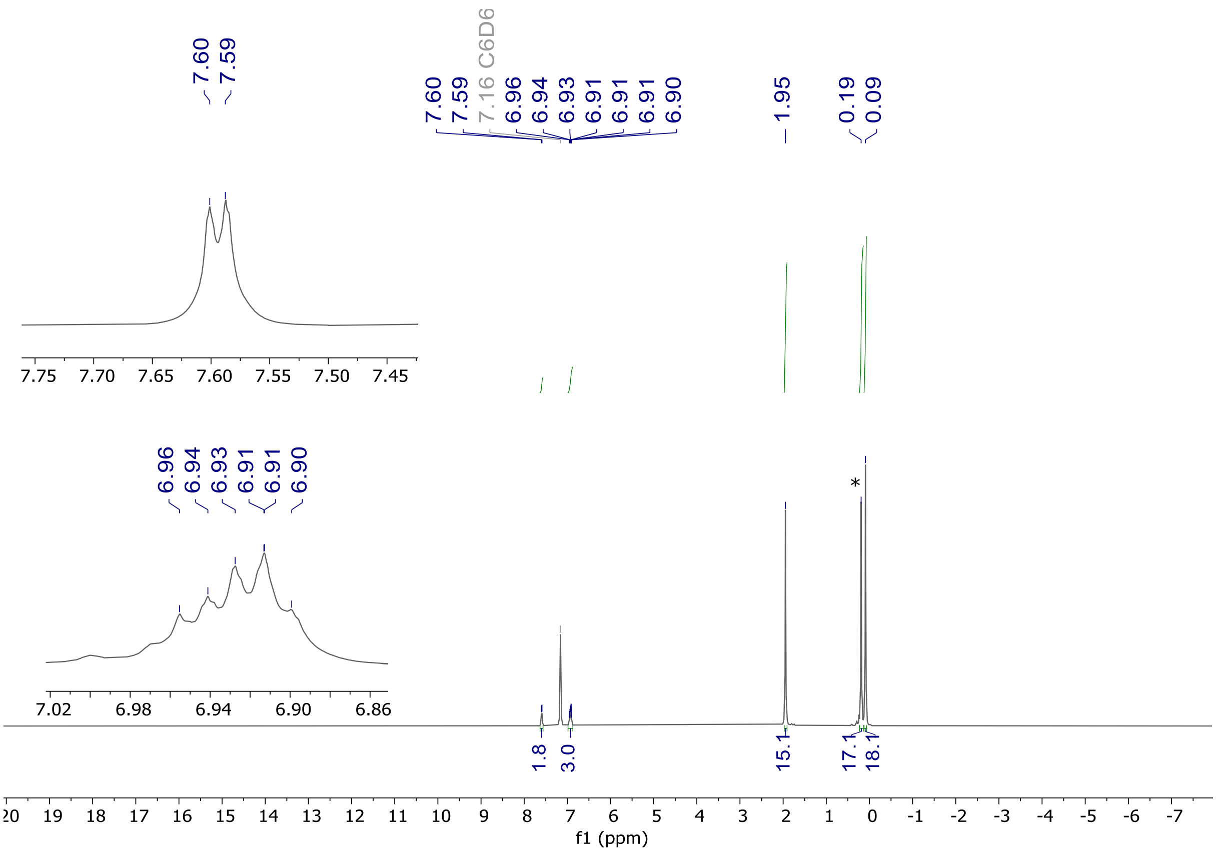

Figure S39: ^{1}H NMR (500 MHz, C_6D_6) of **6**. Cd{N(TMS)$_2$}$_2$ is marked with *.

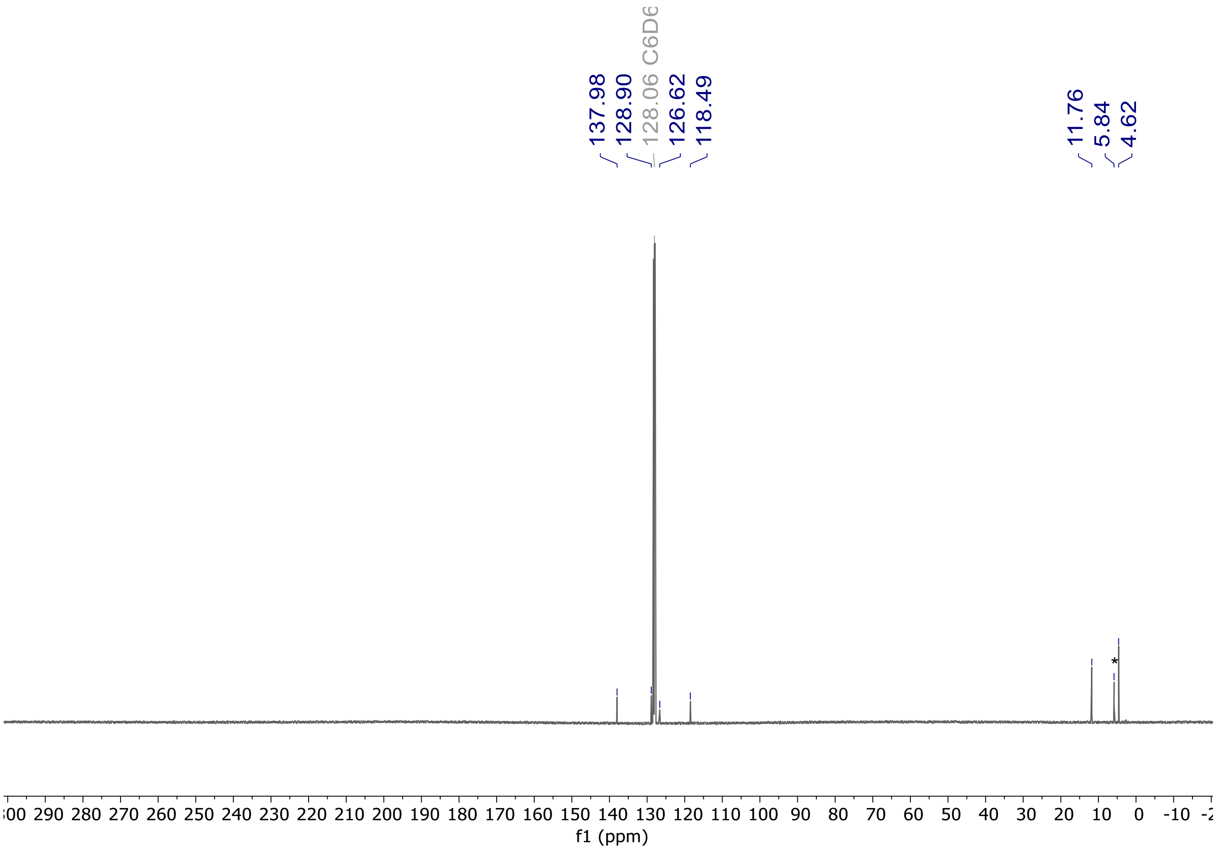

Figure S40: ^{13}C{^{1}H} NMR (126 MHz, C_6D_6) of **6**. Cd{N(TMS)$_2$}$_2$ is marked with *.

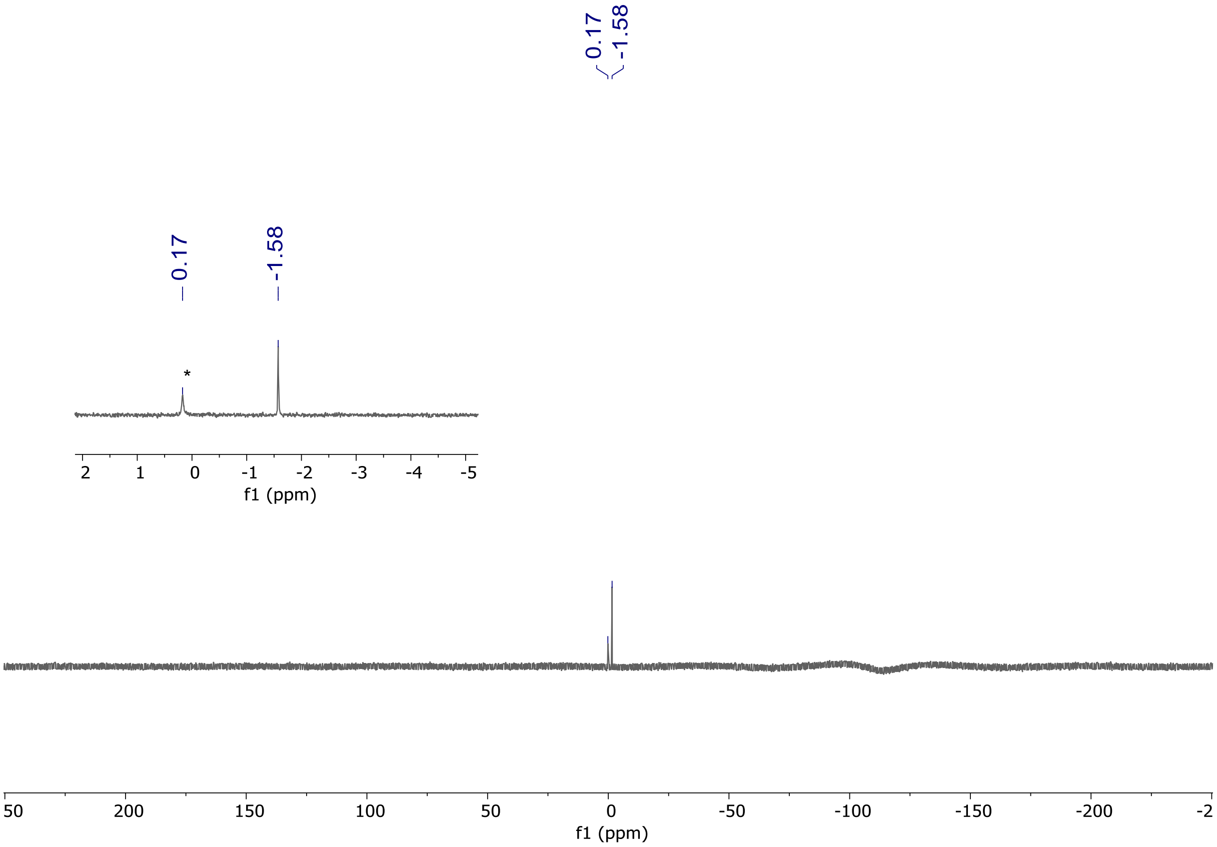

Figure S41: ^{29}Si{^{1}H} NMR (99 MHz, C_6D_6) of **6**. Cd{N(TMS)$_2$}$_2$ is marked with *.

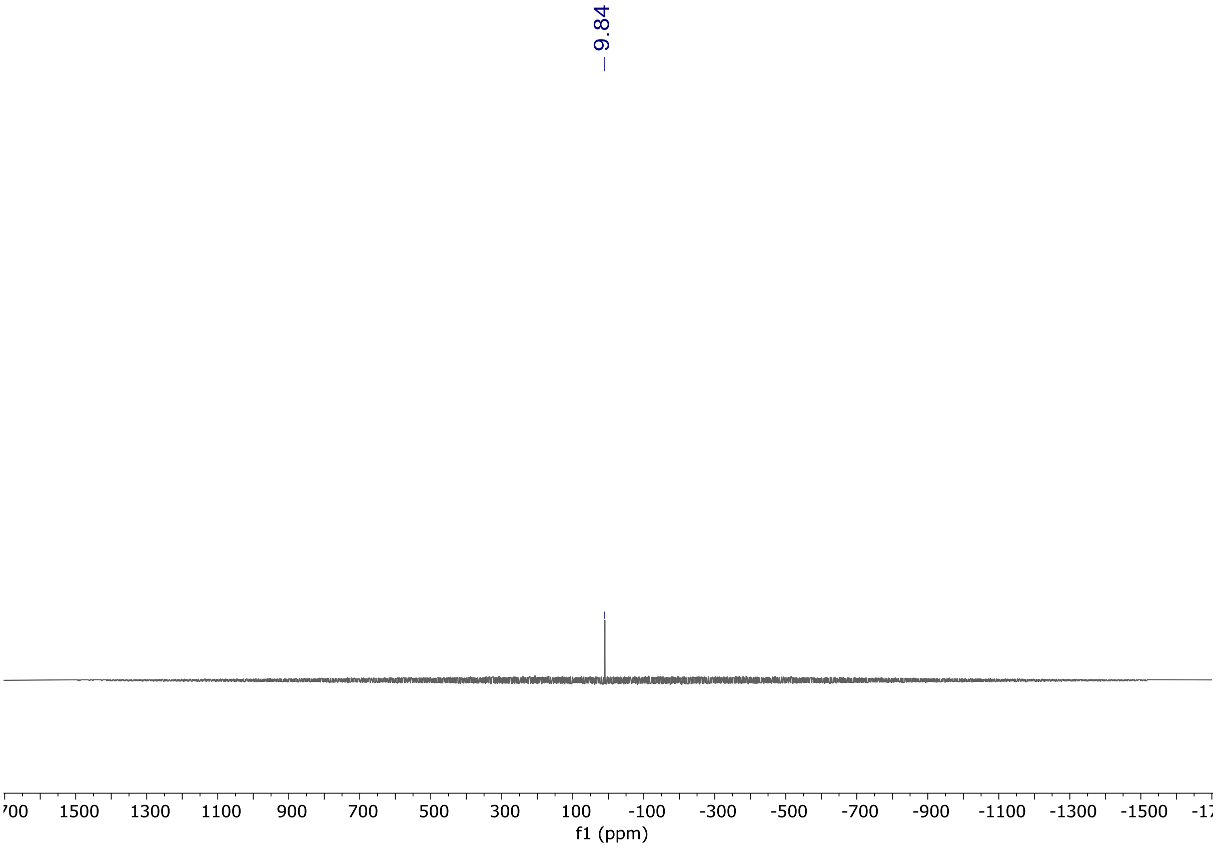

Figure S42: ^{77}Se{^{1}H} NMR (95.31 MHz, C_6D_6) of **6**.

NMR spectra (Part 2)

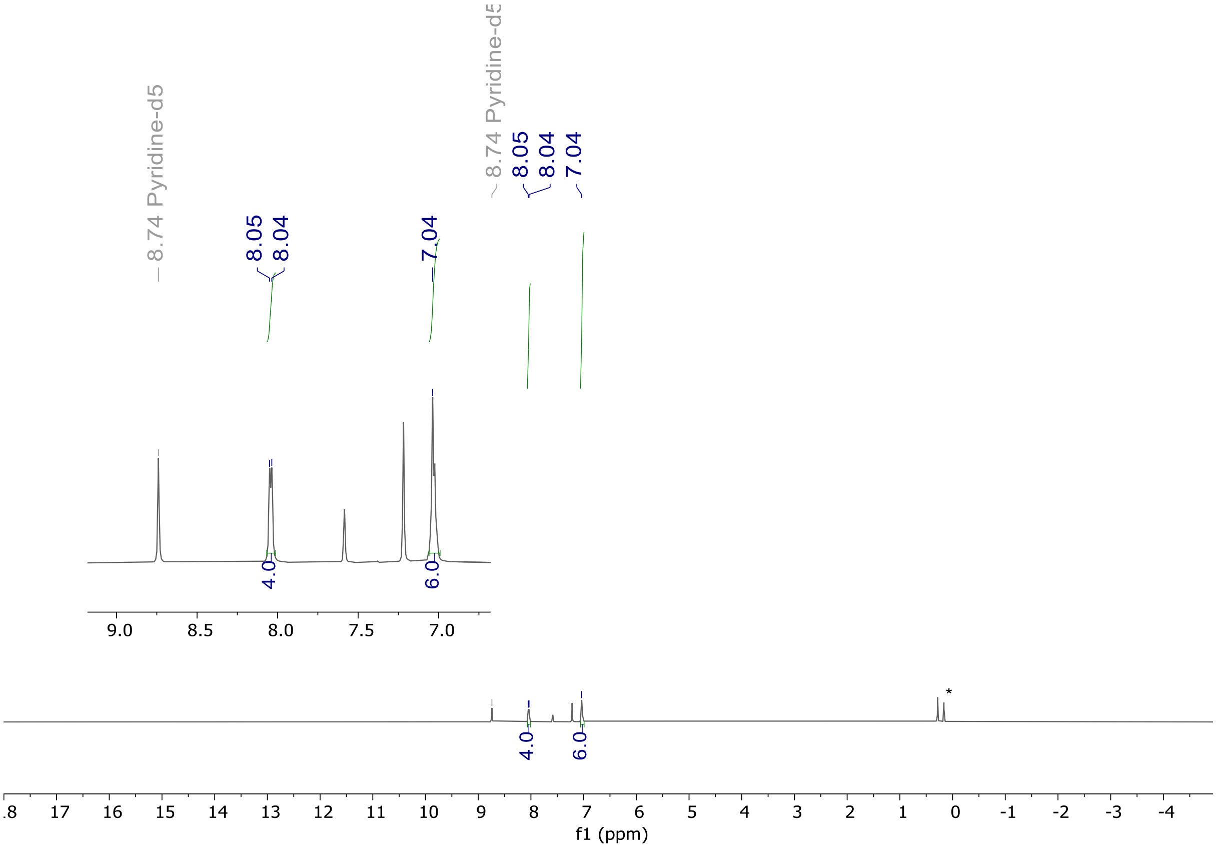

Figure S43: ^{1}H NMR (500 MHz, Pyridine-d_5) of **12**. Trace impurities are marked with *.

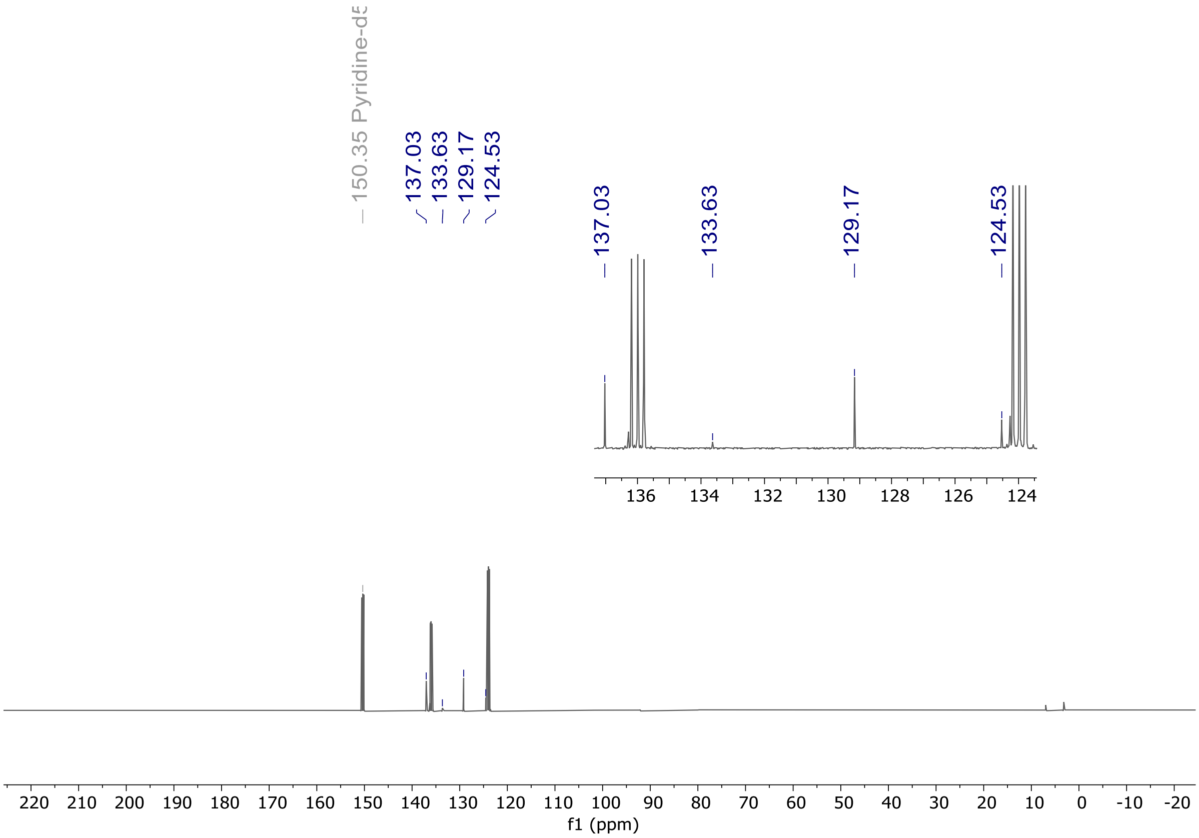

Figure S44: ^{13}C{^{1}H} NMR (126 MHz, Pyridine-d_5) of **12**.

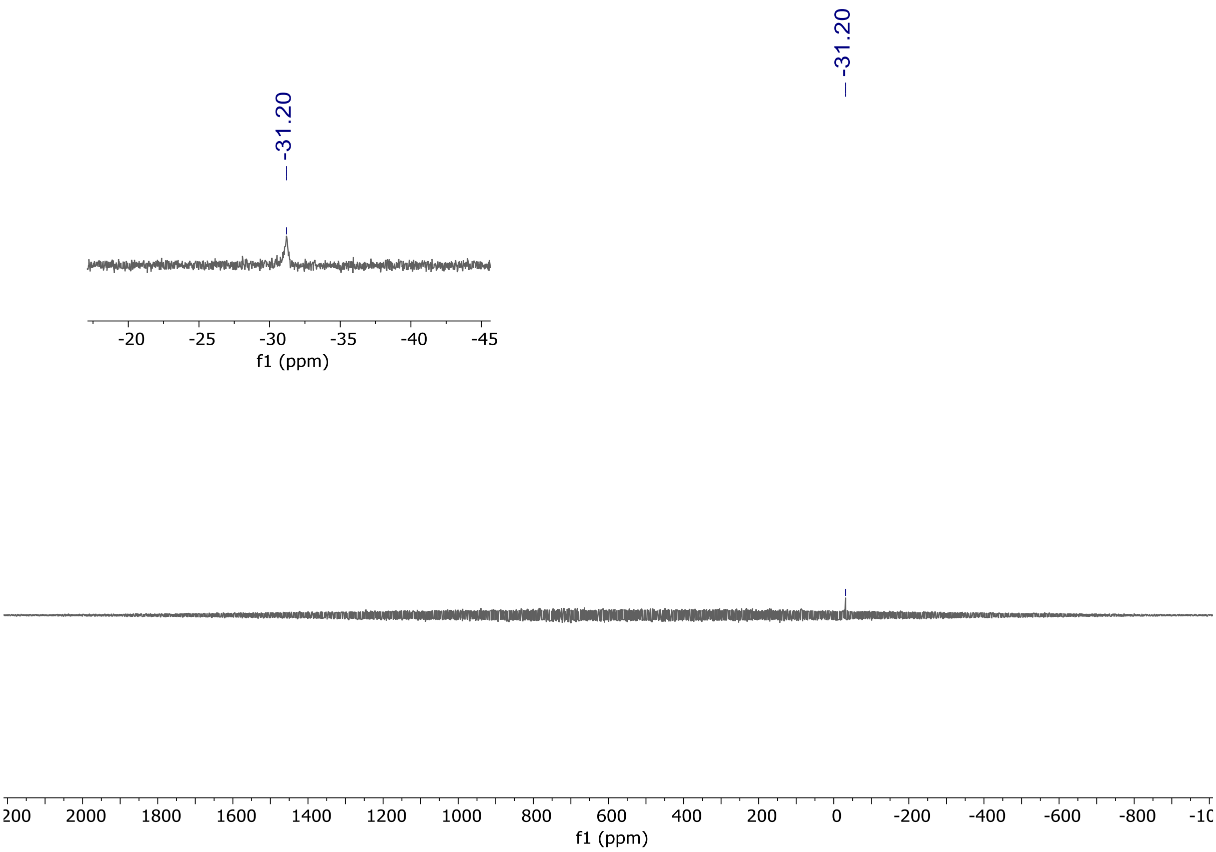

Figure S45: ^{77}Se{^{1}H} NMR (95 MHz, Pyridine-d_5) of **12**.

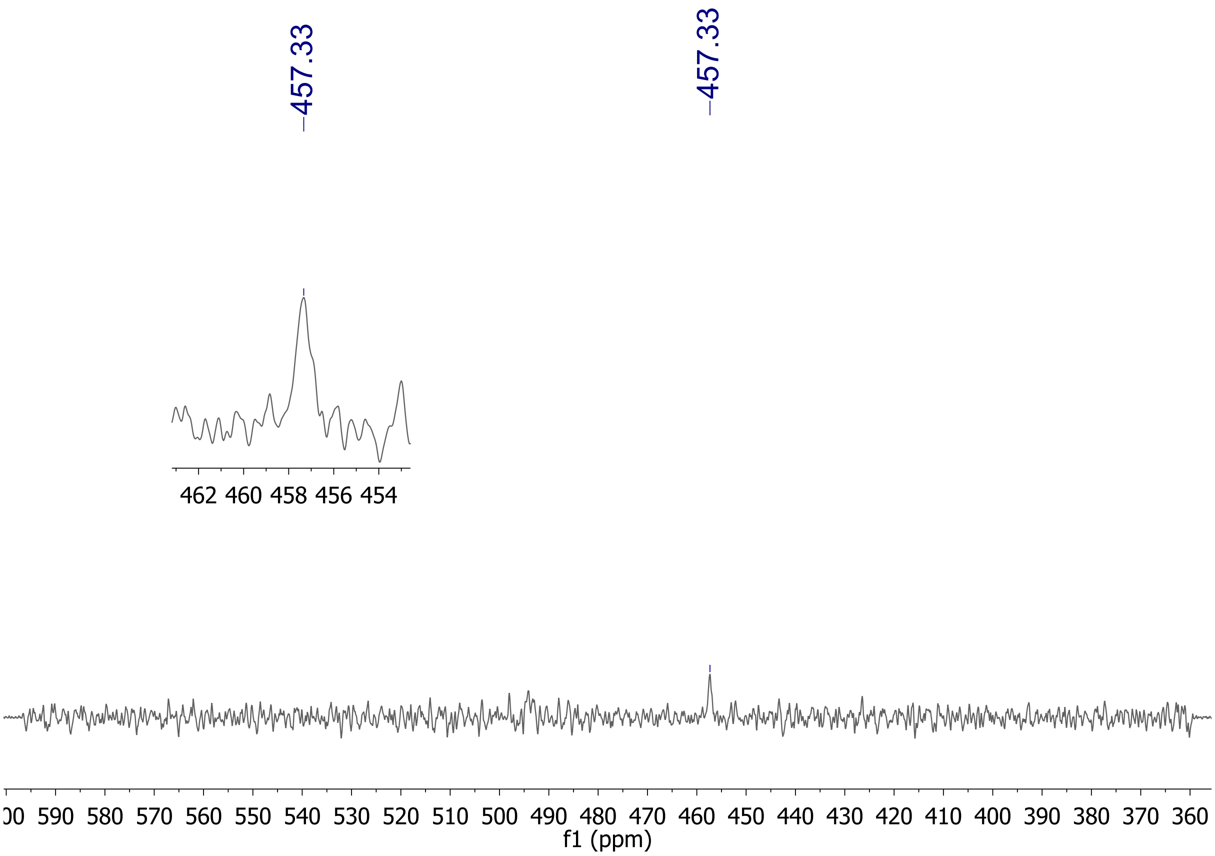

Figure S46: ^{113}Cd NMR (111 MHz, Pyridine-d_5) of **13**.

4.3 Formation of [({N(TMS)$_2$})(Cp*)Al(TePh)] and Cd(TePh)$_2$ starting from 1^{bi}

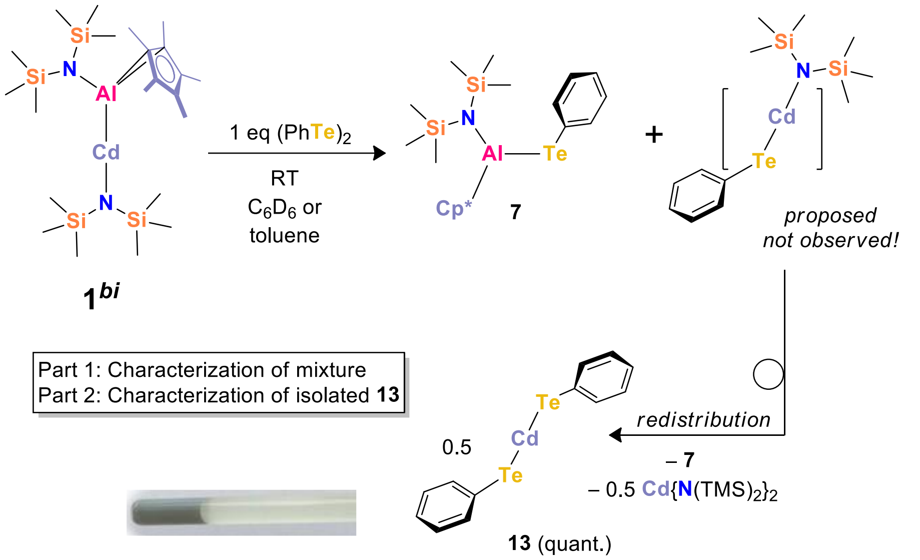

Part 1: In a J. Young NMR tube **1*^{bi}*** was synthesized on a 0.05 mmol scale in 0.7 mL of C_6D_6.[5] Next, diphenyl ditelluride (20.4 mg, 0.05 mmol, 1 eq.), dissolved in 0.5 mL C_6D_6, was added to the solution. The instantaneous formation of a grey precipitate was observed. The supernatant was subjected to NMR spectroscopic analysis.

Part 2: The remaining solution was carefully decanted off, and 1.5 ml of *n*-pentane was added. The mixture was sonicated for 10 minutes, before decanting off the solution again. This step was repeated three times in total. The solid was suspended in ca. 0.6 mL *n*-pentane and transferred to a vial inside of the glovebox. Slow evaporation of *n*-pentane affords **13** as a pale grey powder (13.2 mg, quant.). NMR spectra were recorded after **13** was dissolved in pyridine-d_5.

Analytical Data (Part 1):

^{1}H NMR (500 MHz, C_6D_6, 298 K) ***δ***: 7.81 (br, 2H, CH_{Ar}), 6.99 (br, 1H, CH_{Ar}), 6.84 (br, 2H, CH_{Ar}), 1.91 (s, 15H, CH_3 of Cp*), 0.12 (s, 18H, –CH_3 of HMDS) ppm.

^{13}C{^{1}H} NMR (126 MHz, C_6D_6, 298 K) ***δ***: 142.5 (s, CH_{Ar}), 129.0 (s, CH_{Ar}), 127.3 (s, CH_{Ar}), 119.4 (s, C_q of Cp*), 106.0 (s, C_q of Ph), 11.9 (s, CH_3 of Cp*), 4.7 (s, CH_3 of HMDS) ppm.

^{27}Al NMR (130 MHz, C_6D_6, 298 K) ***δ***: 80.9 ($\omega_{1/2}$ = 1800 Hz) ppm.

^{29}Si{^{1}H} NMR (99 MHz, C_6D_6, 298 K) ***δ***: −1.6 (s, *Si* of HMDS) ppm.

^{125}Te NMR (158 MHz, C_6D_6, 298 K) ***δ***: no signal observed in a spectroscopic range from −1400 to 3400 ppm.

Analytical Data (Part 2):

^{1}H NMR (500 MHz, Pyridine-d_5, 298 K) ***δ***: 8.16 (m, 4H, CH_{Ar}), 7.11 (m, 2H, CH_{Ar}), 6.92 (m, 4H, CH_{Ar}) ppm.

^{13}C{^{1}H} NMR (126 MHz, Pyridine-d_5, 298 K) ***δ***: 142.3 (s, CH$_{Ar}$), 129.3 (s, CH$_{Ar}$), 125.9 (s, CH$_{Ar}$), 106.9 (s, C_q of Ar) ppm.

^{125}Te{^{1}H} NMR (158 MHz, Pyridine-d_5, 298 K) ***δ***: -274 ($ω_{1/2}$ = 250 Hz) ppm.

^{113}Cd NMR (111 MHz, Pyridine-d_5, 298 K): 454 ppm.

NMR spectra (Part 1)

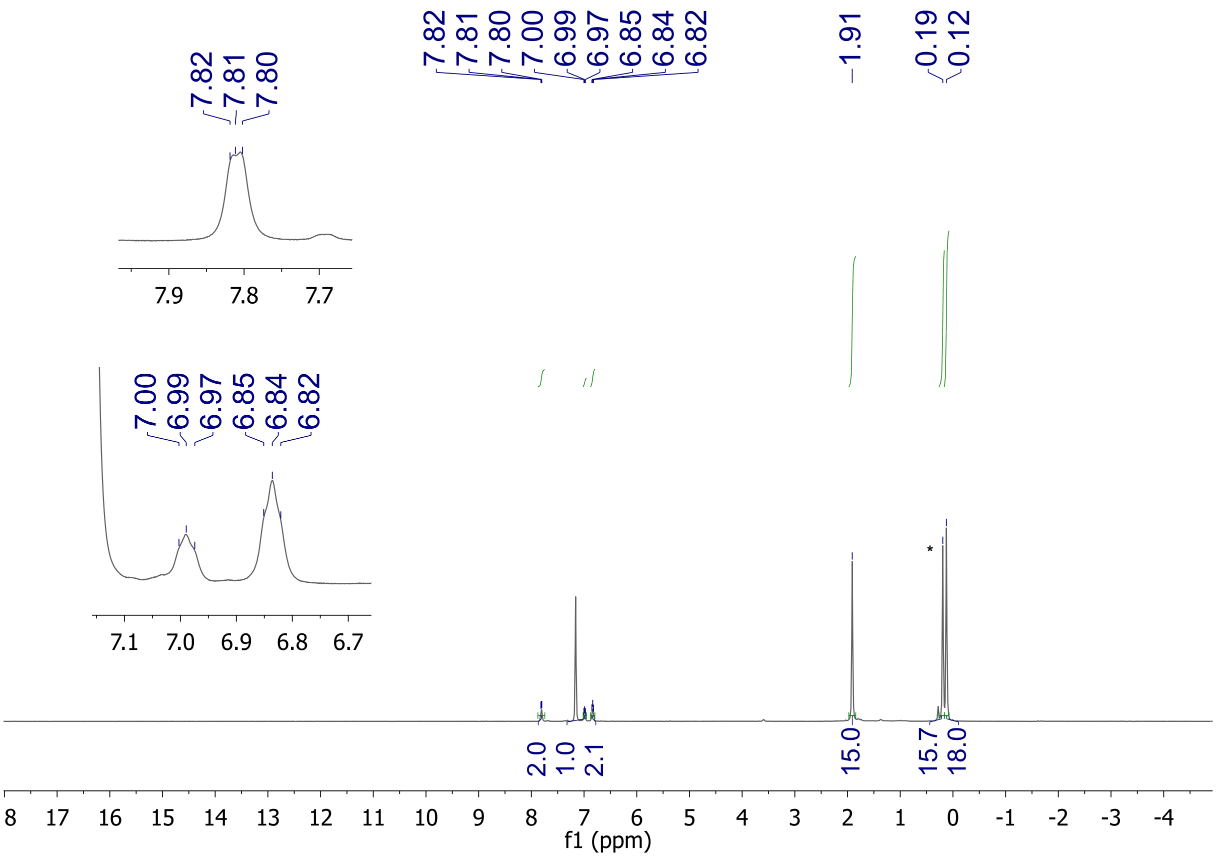

Figure S47: ^{1}H NMR (500 MHz, C_6D_6) of **7**. The signal marked with * belongs to $Cd\{N(TMS)_2\}_2$.

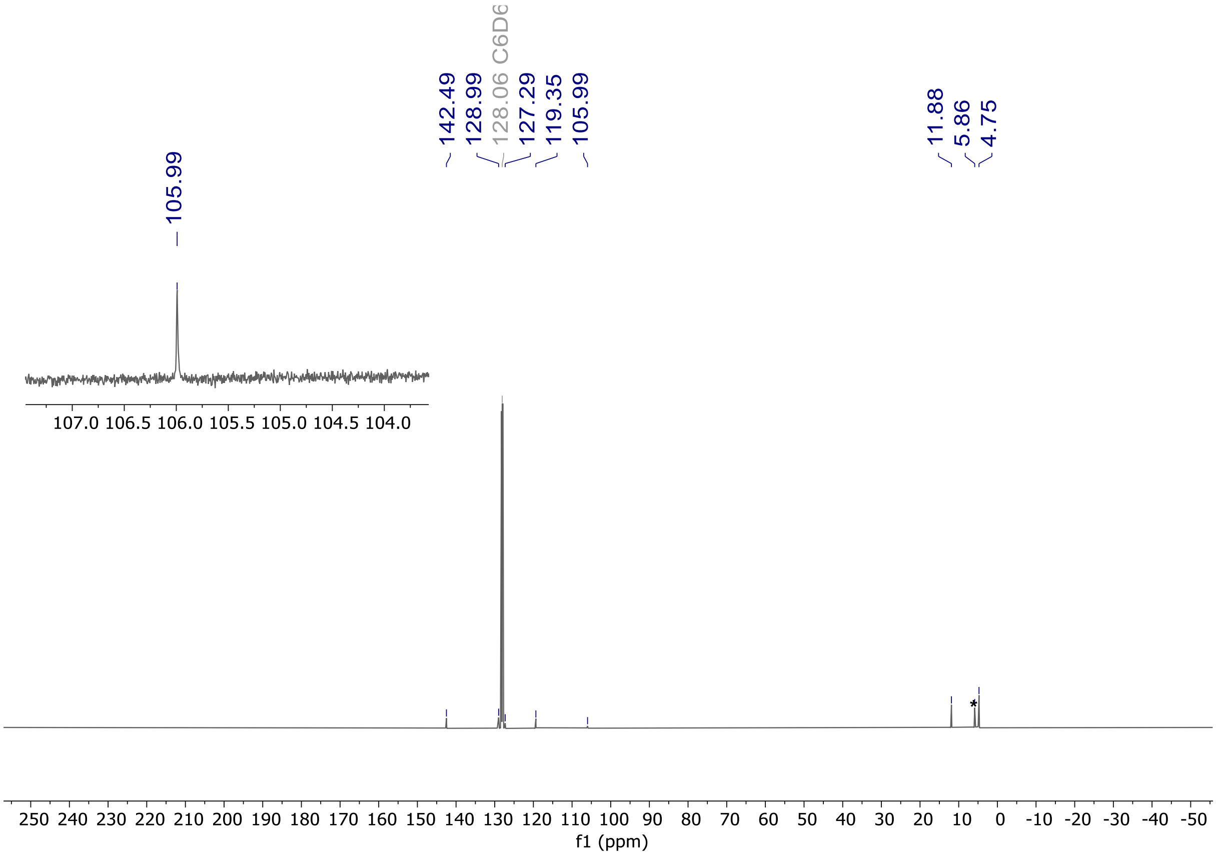

Figure S48: $^{13}C\{^{1}H\}$ NMR (126 MHz, C_6D_6) of **7**. The signal marked with * belongs to $Cd\{N(TMS)_2\}_2$.

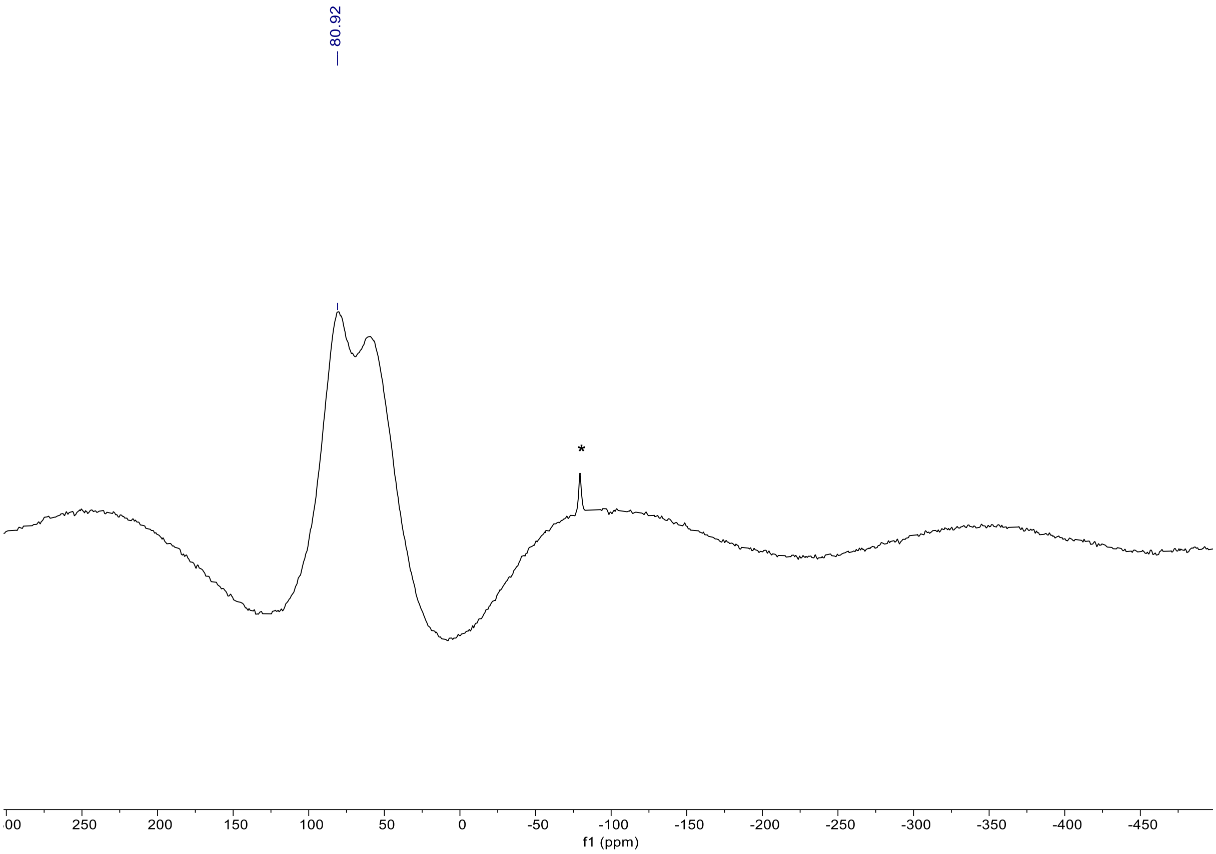

Figure S49: ^{27}Al NMR (130 MHz, C_6D_6) of **7**. The signal marked with * belongs to trace (AlCp*). 50 ppm: probe head.

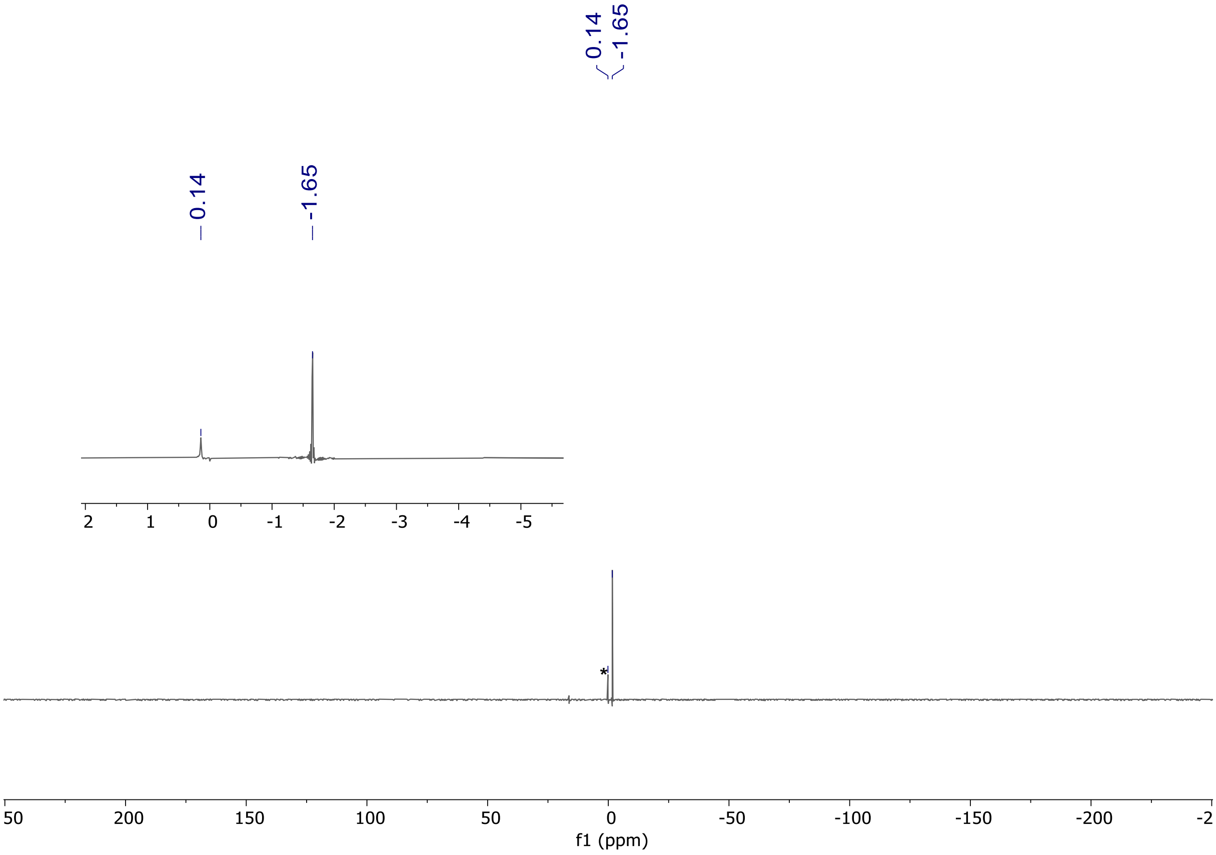

Figure S50: ^{29}Si{^{1}H} NMR (99 MHz, C_6D_6) of **7**. The signal marked with * belongs to $Cd\{N(TMS)_2\}_2$.

NMR spectra (Part 2)

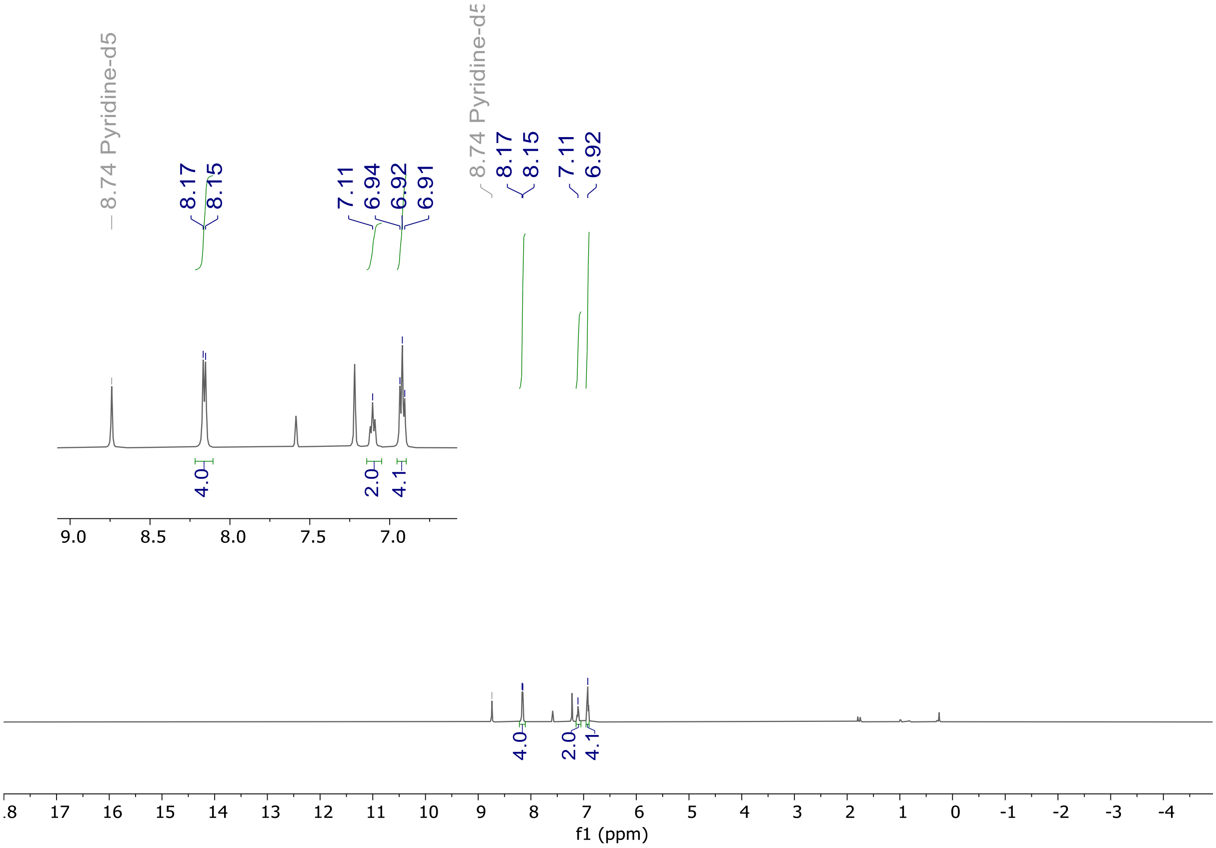

Figure S51: ^{1}H NMR (500 MHz, Pyridine-d_5) of **13**.

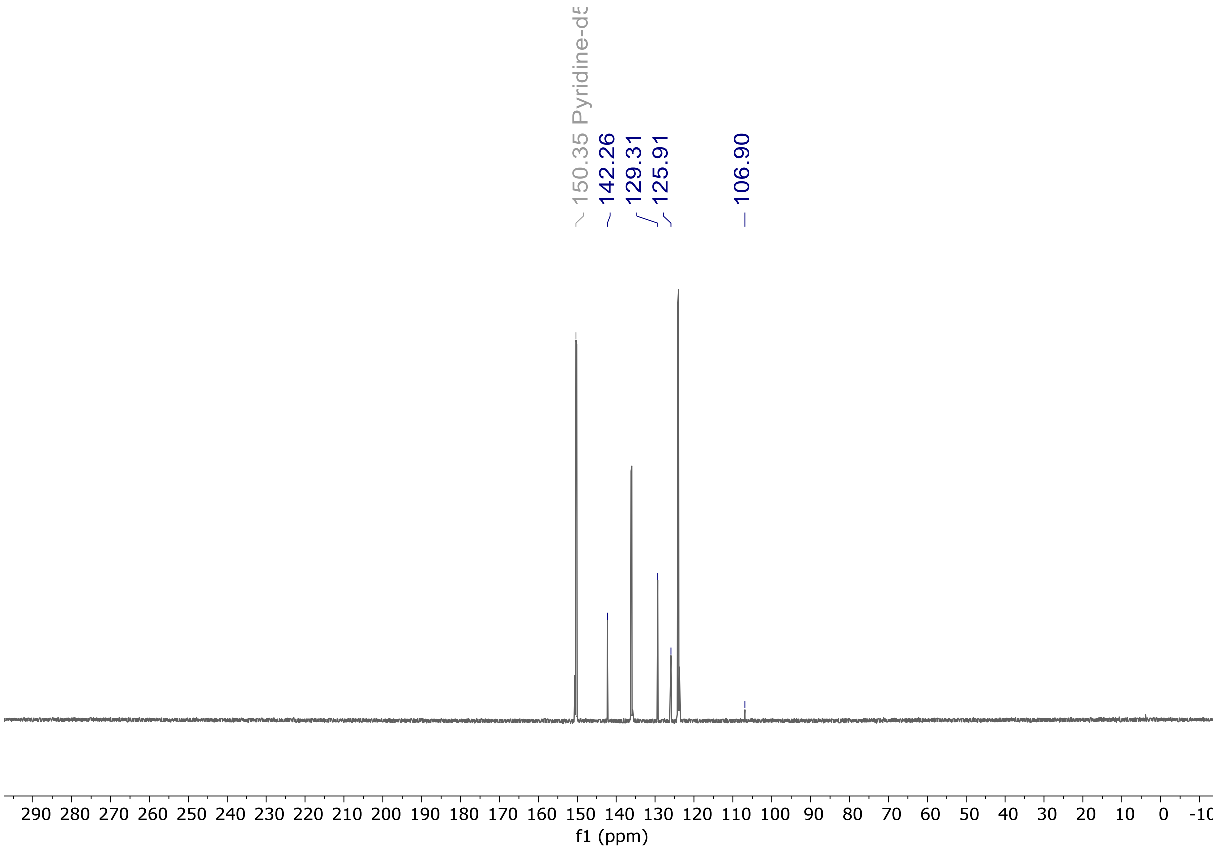

Figure S52: ^{13}C{^{1}H} NMR (126 MHz, Pyridine-d_5) of **13**.

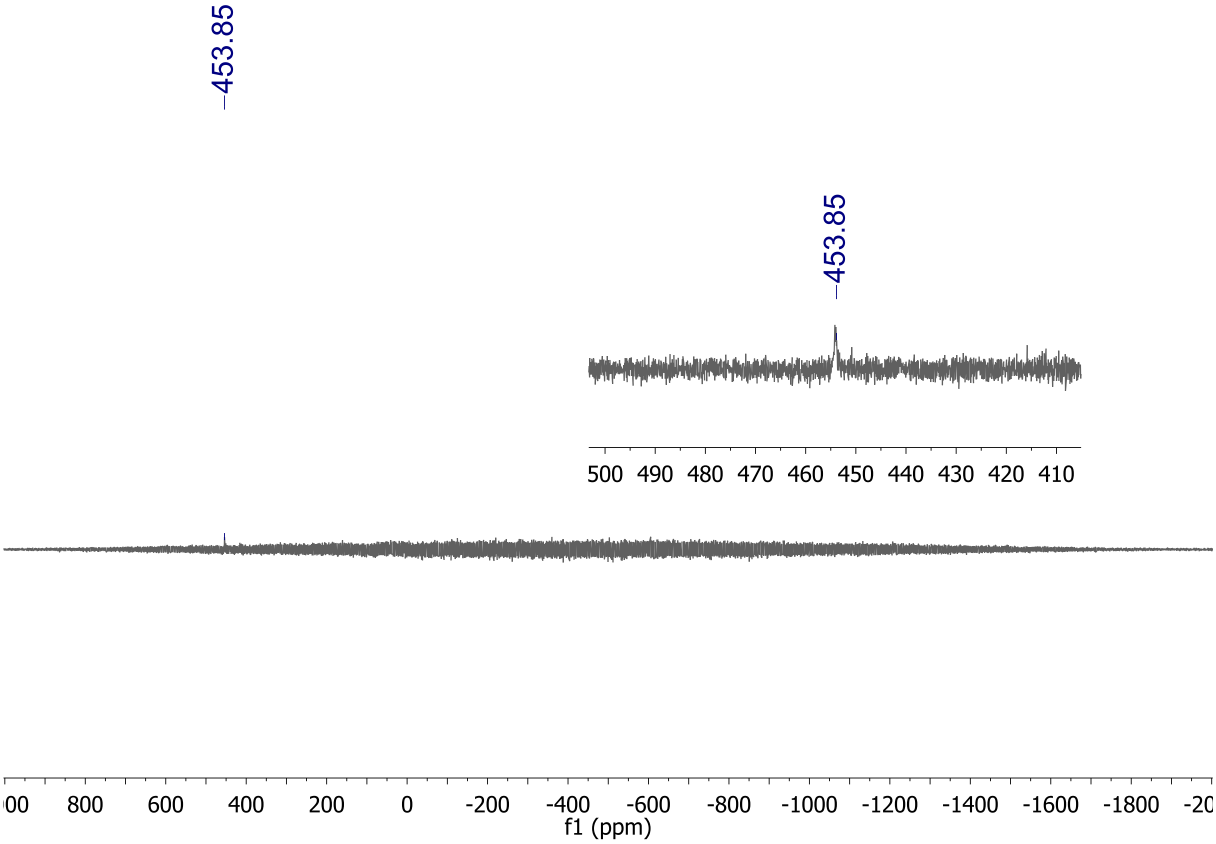

Figure S53: ^{113}Cd NMR (111 MHz, Pyridine-d_5) of **13**.

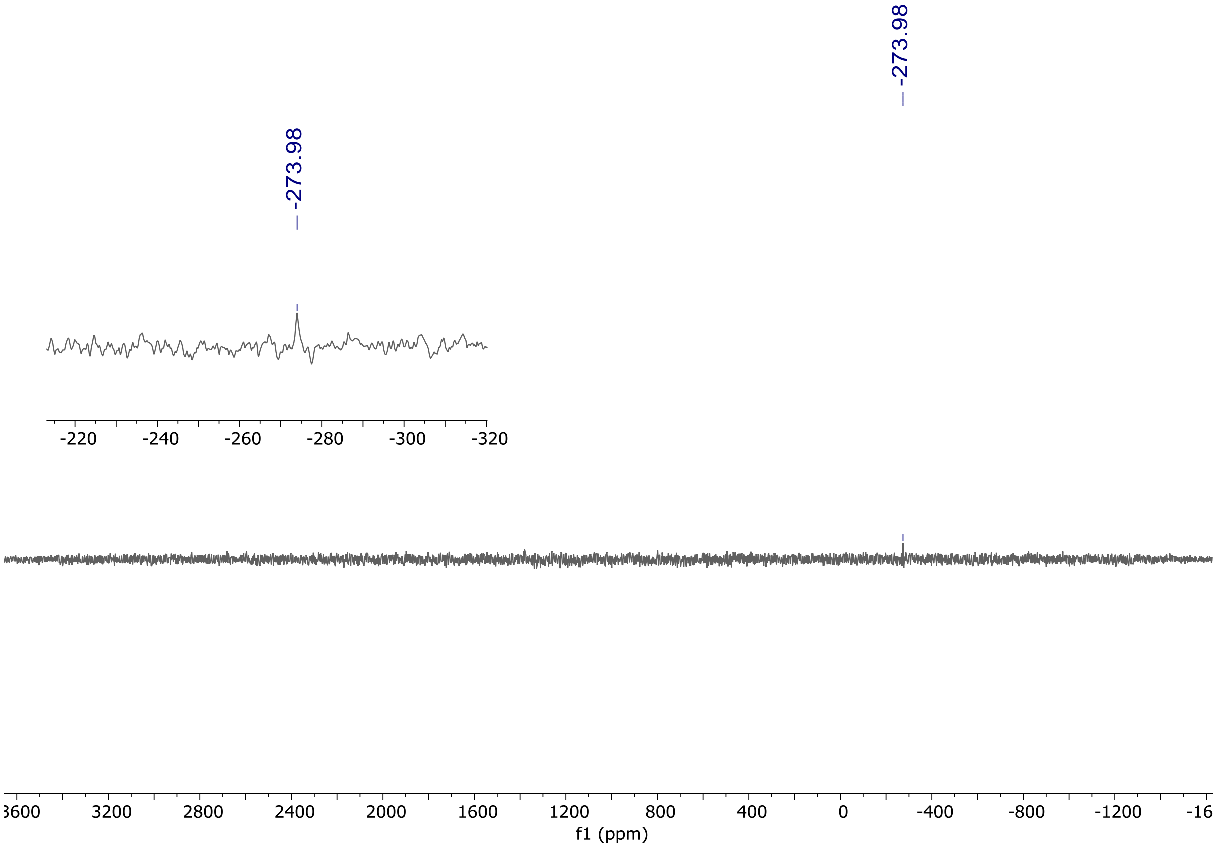

Figure S54: ^{125}Te NMR (158 MHz, Pyridine-d_5) of **13**.

.

5 Additional NMR studies

5.1 Screening of the reaction of 1^{tri} *via* two equiv. of ABNO

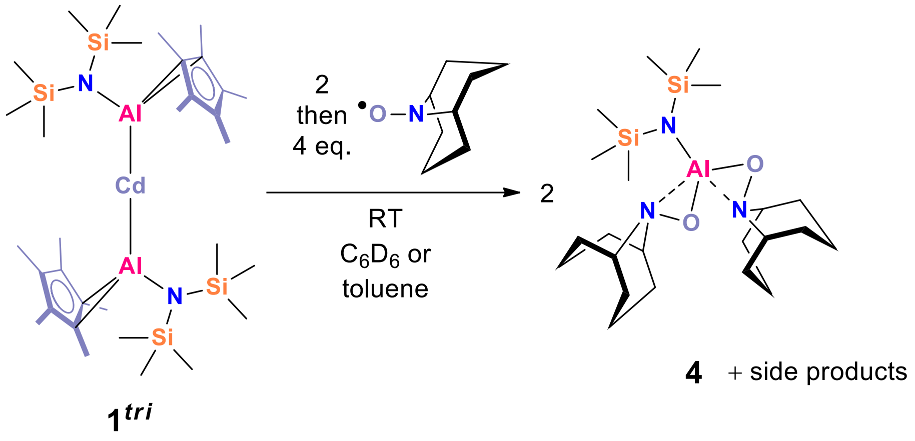

1^{tri} was synthesized in a J. Young NMR tube according to literature on a 0.03 mmol scale (based on Cd{N(TMS)$_2$}$_2$ in 0.7 mL of C_6D_6.[5] After full conversion of Cd{N(TMS)$_2$}$_2$, ABNO (7.3 mg, 0.05 mmol, 1.86 eq.) was added, followed by ^{1}H NMR screening. The conversion to **4** is roughly 50%. The addition of two further equivalents of ABNO (7.9 mg, 0.06 mmol, 2 eq.) leads to near quantitative conversion of **1**^{tri} with simultaneous formation of unidentified Cp* containing side products.

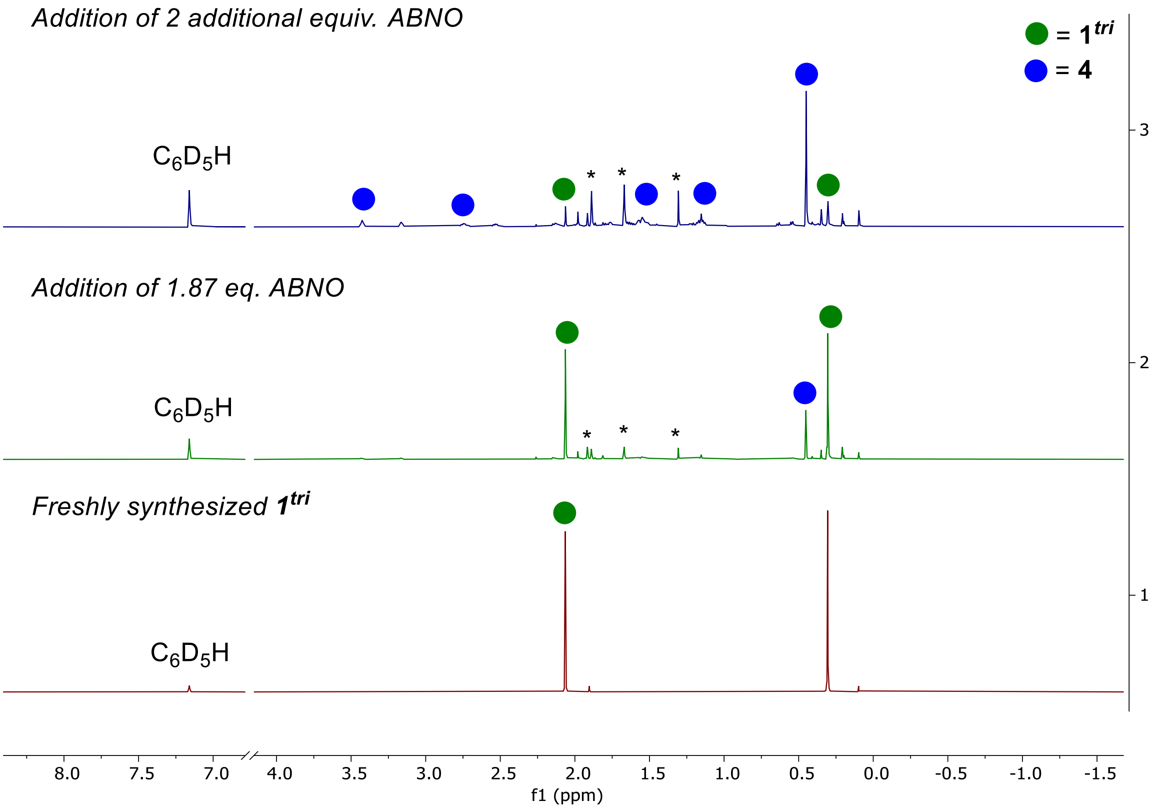

Figure S55: ^{1}H NMR (500 MHz, C_6D_6) screening of the reaction between **1**^{tri} (and two equivalents of ABNO. The conversion of the reaction is ca. 50% and traces of Cp* containing side products can be identified (marked with *). The subsequent addition of two more equivalents ABNO leads to almost full conversion of **1**^{tri}.

5.2 Attempted reaction between 1^{tri} and Gomberg's Dimer

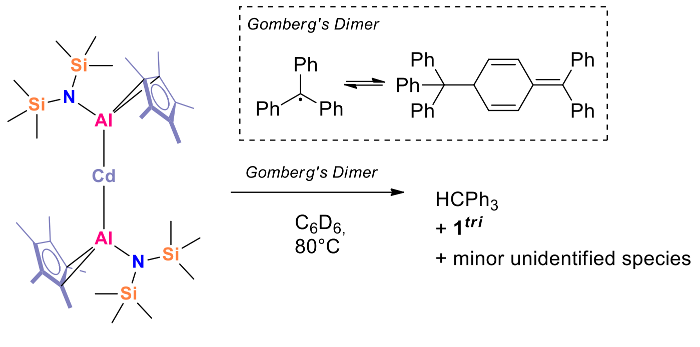

1^{tri}

1^{tri} was synthesized in a J. Young NMR tube on a 0.028 mmol scale (based on $Cd\{N(TMS)_2\}_2$ in 0.7 ml of C_6D_6.[5] After full conversion of $Cd\{N(TMS)_2\}_2$, 1 equivalent of the Gomberg Dimer (containing·0.6 molecules of toluene per dimer)[6] was added (15.2 mg, 0.028 mmol, 1 equiv.). At ambient temperature, no reaction is observed. Prolonged heating causes the slow formation of minor new, mostly unidentified species. Whereas **1**^{tri} is barely consumed, Gomberg's Dimer decomposes to triphenylmethane.

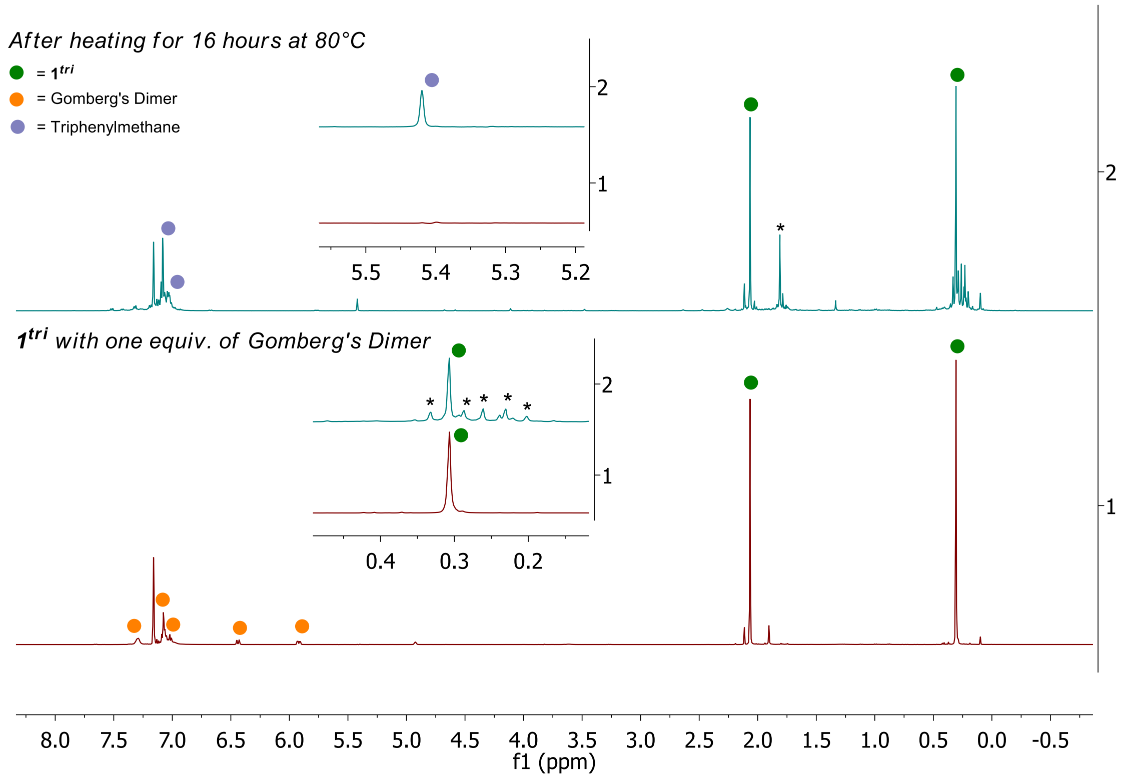

Figure S56: ^1H NMR (500 MHz, C_6D_6) of **1**^{tri} and "*Gomberg's Dimer*" after heating for a prolonged period at 80°C. We note no conversion at ambient temperature. Barely conversion of **1**^{tri} can be noted at elevated temperatures, and *"Gomberg's Dimer"* converts to triphenylmethane. Thus, radical trapping with a triphenylmethylradical is not expedient. Minor amounts of unidentified species can be observed (indicated by *).

5.3 Reaction of 1^{bi} with TEMPO

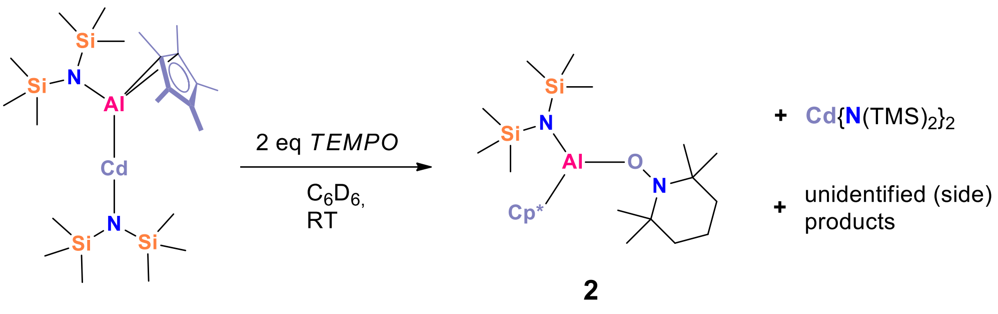

1^{bi} was synthesized in a J. Young NMR tube on a 0.04 mmol scale in 0.7 mL of C_6D_6.[5] After full conversion, TEMPO (12.5 mg, 0.08 mmol, 2 equiv.) was added, followed by ^{1}H NMR screening. The target species **2** was identified yet concomitant with other unidentified side products and Cd{N(TMS)$_2$}$_2$.

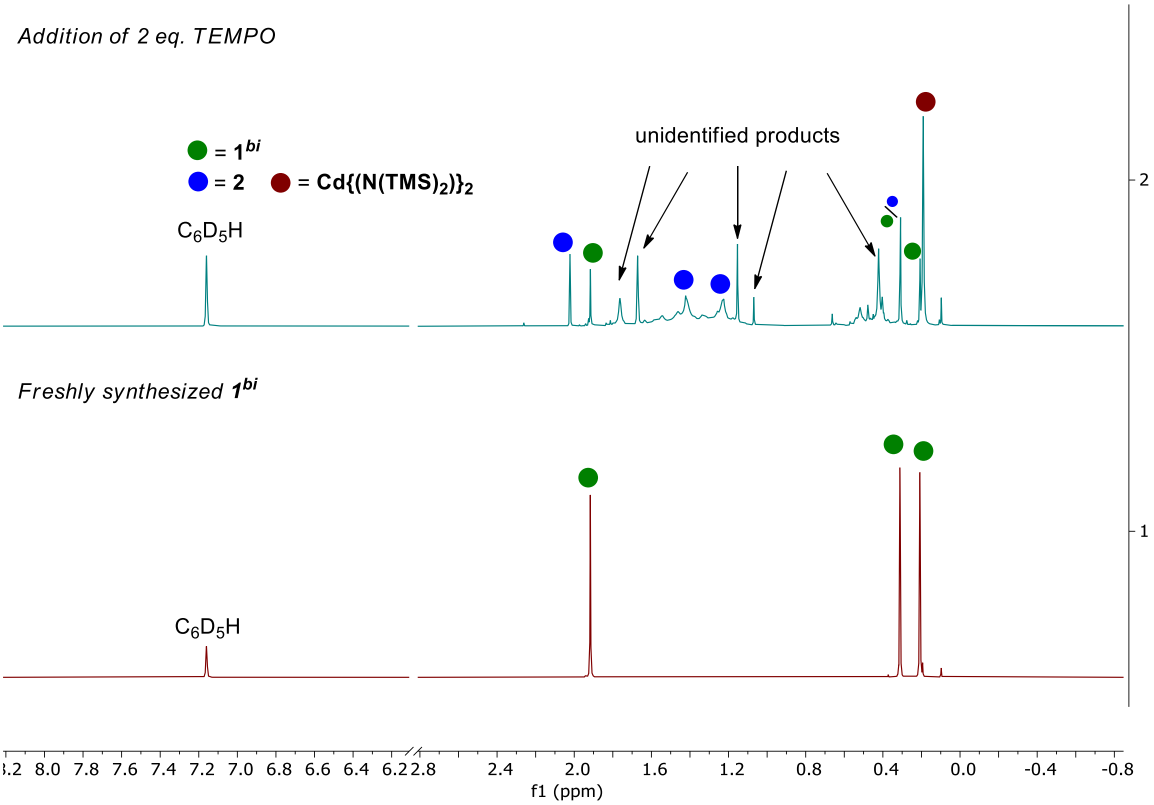

Figure S57: ^{1}H NMR (500 MHz, C_6D_6) of the reaction between **1**^{bi} and two equivalents of TEMPO. Al(**II**) transfer is achieved, yet the reaction is not expedient due to several species remaining unidentified. Identified are Cd{N(TMS)$_2$}$_2$ among **2,** the latter can only be found in minor amounts.

5.4 Reaction monitoring of 1^{tri} with Benzophenone

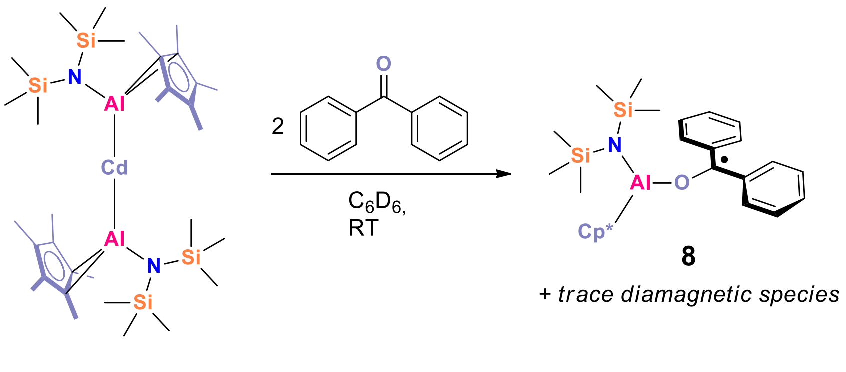

$\mathbf{1}^{tri}$

$\mathbf{1}^{tri}$ was synthesized according to literature on a 0.02 mmol scale (based on Cd{N(TMS)$_2$}$_2$) in 0.8 mL of C_6D_6.[5] After full conversion of Cd{N(TMS)$_2$}$_2$, one equivalent of benzophenone (3.6 mg, 0.02 mmol) and the reaction mixture was subjected to ^{1}H NMR. Quickly afterwards, an additional equivalent of benzophenone (3.6 mg, 0.02 mmol) was added which then leads to almost full consumption of $\mathbf{1}^{tri}$. Only trace amounts of diamagnetic impurities and/or decomposition products are then identified.

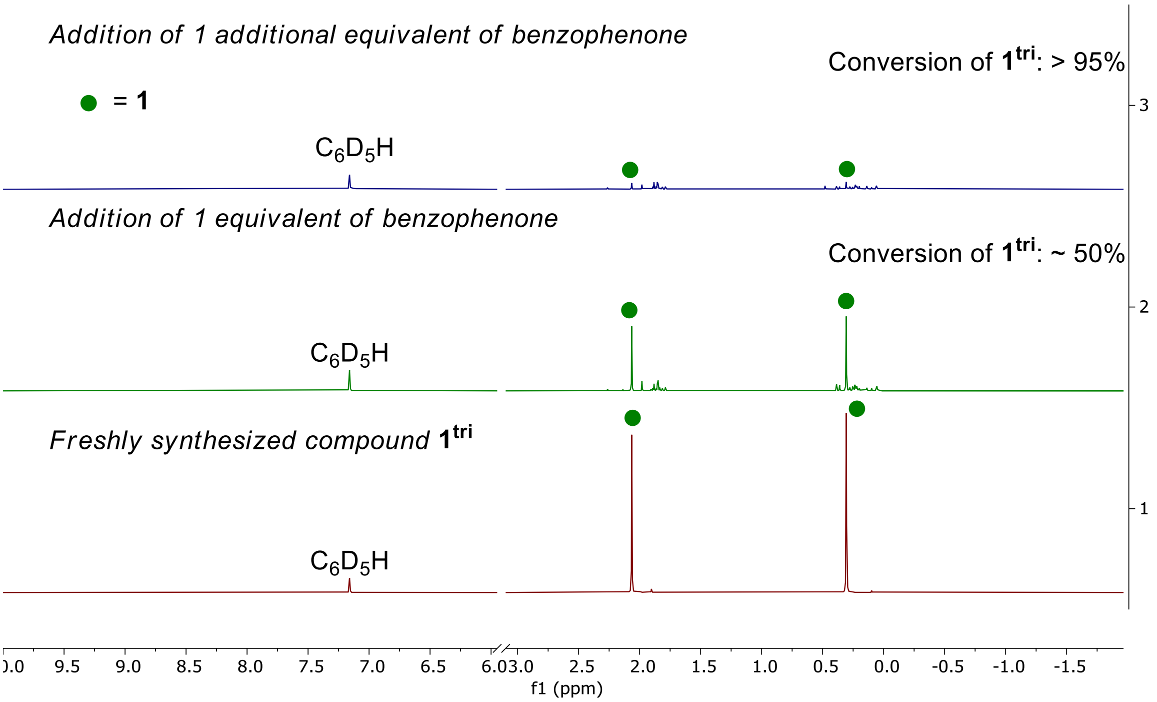

Figure S58: ^{1}H NMR (500 MHz, C_6D_6) of $\mathbf{1}^{tri}$ and subsequent additions of 1 equivalent of benzophenone. After two equivalents $\mathbf{1}^{tri}$ is fully converted to a paramagnetic species and trace diamagnetic side and/or decomposition products.

5.5 Reaction monitoring of 1^{tri} with 2,2-pyridylketone

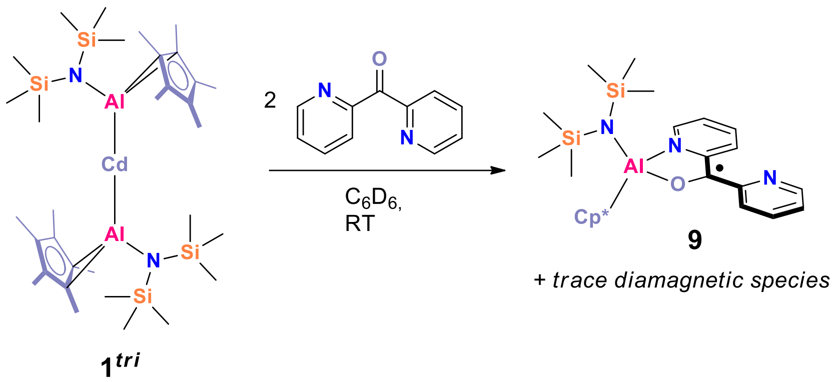

During workup procedures noted in section 3.8, an aliquot (0.4 mL of the filtered *n*-pentane solution) of **9** was transferred to a J. Young NMR tube. The solvent was removed under reduced pressure, and the dark purple residue was analyzed rapidly *via* ^{1}H NMR in C_6D_6. Only trace amounts of diamagnetic species were observed – among these, one can tentatively be assigned to compound **10** (*vide infra*).

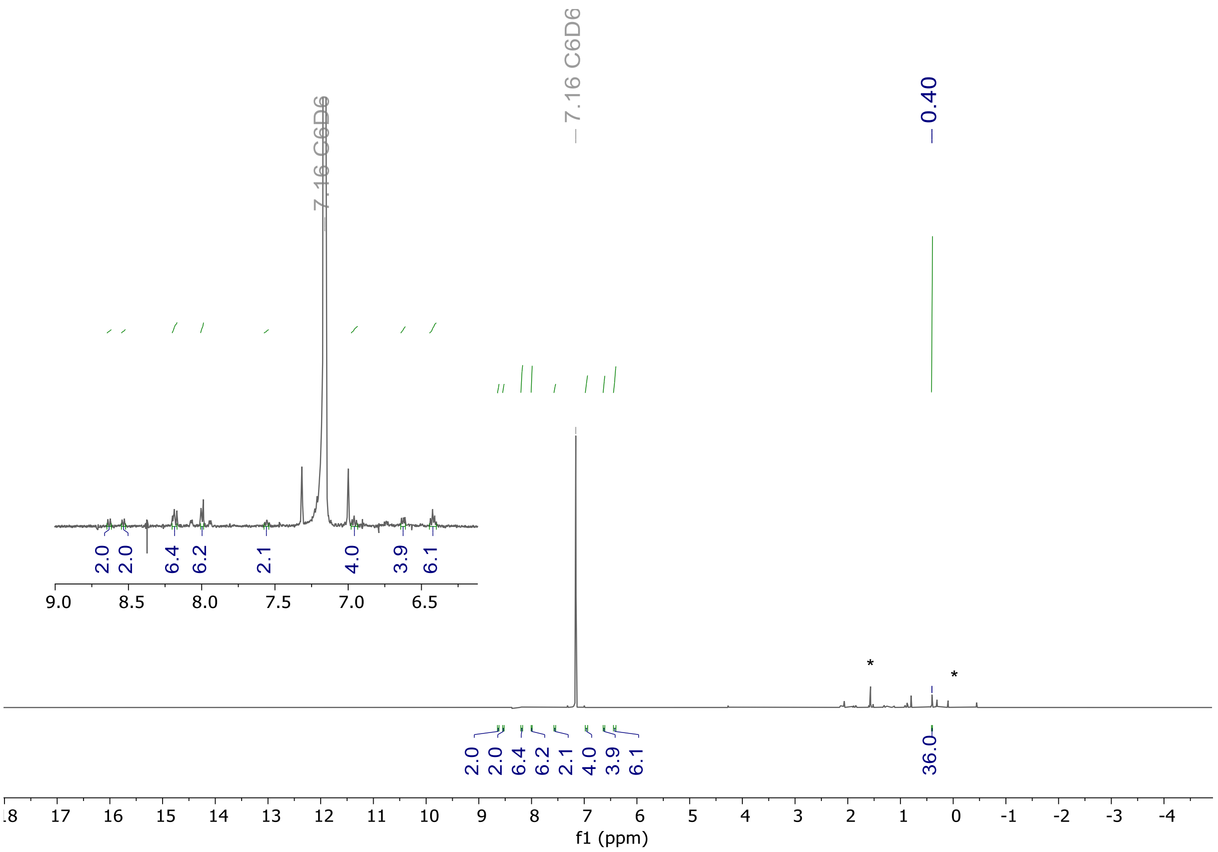

Figure S59: ^{1}H NMR (500 MHz, C_6D_6) of a freshly prepared sample of **9** (including tentatively assigned **10** and further unidentified species marked with *). *Please note that the extremely poor signal to noise ratio is intended to demonstrate that these species are only present in absolute trace amounts and that conversion almost exclusively yields the paramagnetic compound **9**!*

6 EPR study

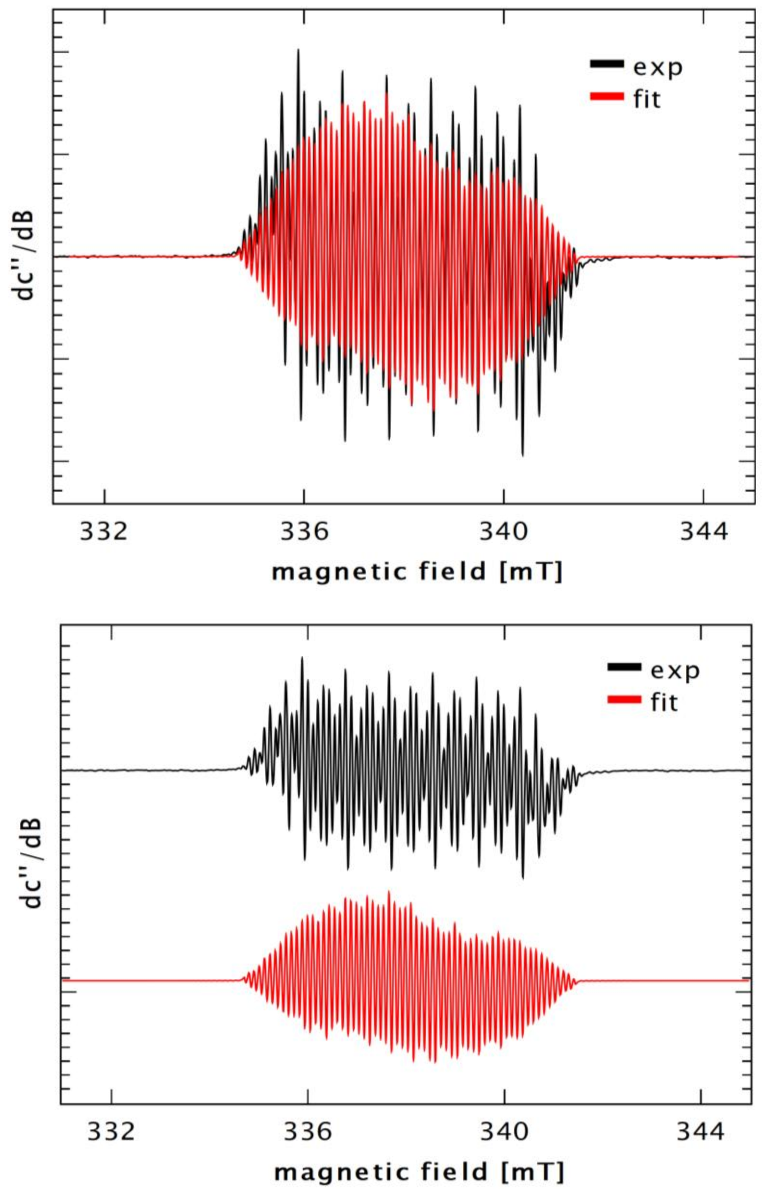

Figure S60: Experimental (black) and simulated (red) continuous-wave (CW) X-band EPR spectra of the liquid phase of the reaction between $\mathbf{1}^{tri}$ (1.0 equiv.) and 2 $OCPh_2$ (2.0 equiv.) in benzene/toluene (1:1) (c(compound **8**)) = ca. 0.036 mmol mL^{-1}). The observed resonance shows coupling constants of a(1 × ^{1}H) = 28.2 MHz (10.1 G, 1.01 mT), a(1 × ^{1}H) = 24.0 MHz (8.56 G, 0.856 mT), a(1 × ^{1}H) = 20.7 MHz (7.39 G, 0.739 mT), a(1 × ^{1}H) = 18.6 MHz (6.64 G, 0.664 mT), a(1 × ^{1}H) = 9.17 MHz (3.27 G, 0.327 mT), a(1 × ^{1}H) = 9.07 MHz (3.24 G, 0.324 mT), a(1 × ^{1}H) = 5.85 MHz (2.09 G, 0.209 mT), a(1 × ^{1}H) = 3.15 MHz (1.12 G, 0.112 mT), a(1 × ^{1}H) = 2.88 MHz (1.03 G, 0.103 mT), a(1 × ^{1}H) = 2.42 MHz (0.864 G, 0.0864 mT), a(1 × ^{27}Al) = 12.6 MHz (4.50 G, 0.450 mT), a(1 × ^{13}C) = 75.5 MHz (26.9 G, 2.69 mT), and a g_{iso} value of 2.0023. Spectrometer settings: microwave frequency = 9.475891 GHz, 0.020 mT modulation amplitude at 100 kHz, microwave power = 1.0 mW, temperature = 23 °C, number of accumulated scans = 1, conversion time = 2 ms.

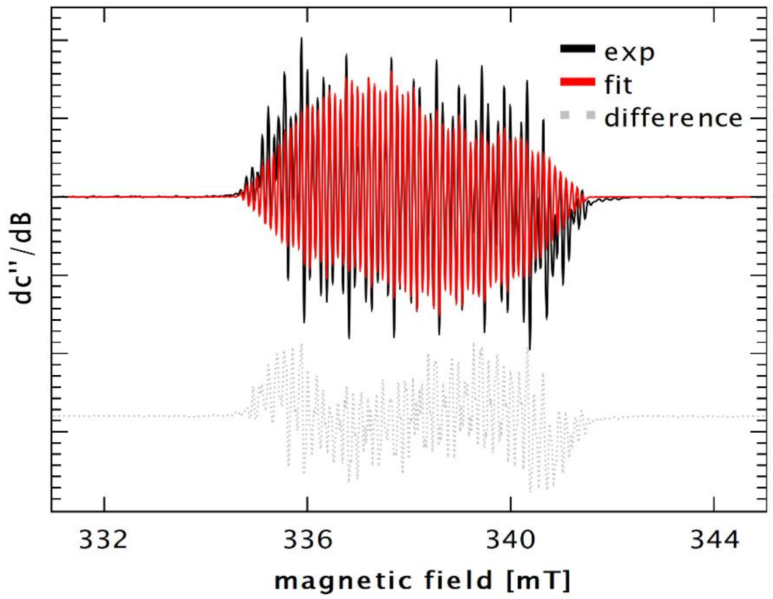

Figure S61: Experimental (black) and simulated (red) continuous-wave (CW) X-band EPR spectra of the liquid phase of the reaction between **1*tri*** (1.0 equiv.) and 2 $OCPh_2$ (2.0 equiv.) in benzene/toluene (1:1) (c(compound **8**)) ca. 0.036 mmol·L^{-1}). The observed resonance shows coupling constants of a(1 × ^{1}H) = 28.2 MHz (10.1 G, 1.01 mT), a(1 × ^{1}H) = 24.0 MHz (8.56 G, 0.856 mT), a(1 × ^{1}H) = 20.7 MHz (7.39 G, 0.739 mT), a(1 × ^{1}H) = 18.6 MHz (6.64 G, 0.664 mT), a(1 × ^{1}H) = 9.17 MHz (3.27 G, 0.327 mT), a(1 × ^{1}H) = 9.07 MHz (3.24 G, 0.324 mT), a(1 × ^{1}H) = 5.85 MHz (2.09 G, 0.209 mT), a(1 × ^{1}H) = 3.15 MHz (1.12 G, 0.112 mT), a(1 × ^{1}H) = 2.88 MHz (1.03 G, 0.103 mT), a(1 × ^{1}H) = 2.42 MHz (0.864 G, 0.0864 mT), a(1 × ^{27}Al) = 12.6 MHz (4.50 G, 0.450 mT), a(1 × ^{13}C) = 75.5 MHz (26.9 G, 2.69 mT), and a g_{iso} value of 2.0023. Spectrometer settings: microwave frequency = 9.475891 GHz, 0.020 mT modulation amplitude at 100 kHz, microwave power = 1.0 mW, temperature = 23 °C, number of accumulated scans = 1, conversion time = 2 ms.

Comment:

The EPR spectroscopic parameters analyzed by fitting the EPR spectrum of compound **8** showed a strong correlation during the fitting procedure, which inherently complicated the determination of unequivocal results. Due to these correlations, the fit does not represent a unique description of the system. While the fit does not ideally replicate peak intensities, the model properly represents the overall coupling pattern. Difficulties in fitting detailed features of the EPR spectra of related systems are apparent from previous reports (see the SI of ref. *J. Am. Chem. Soc.* **2025**, *147*, 12715–12721).

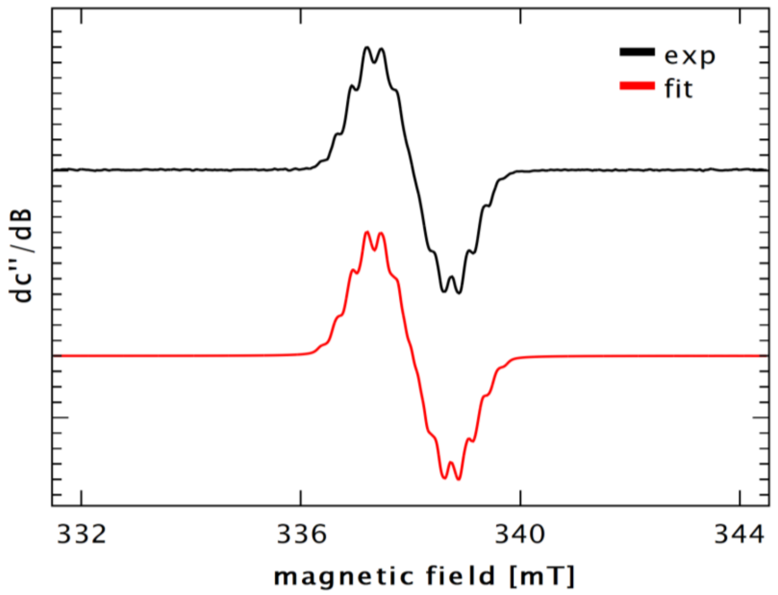

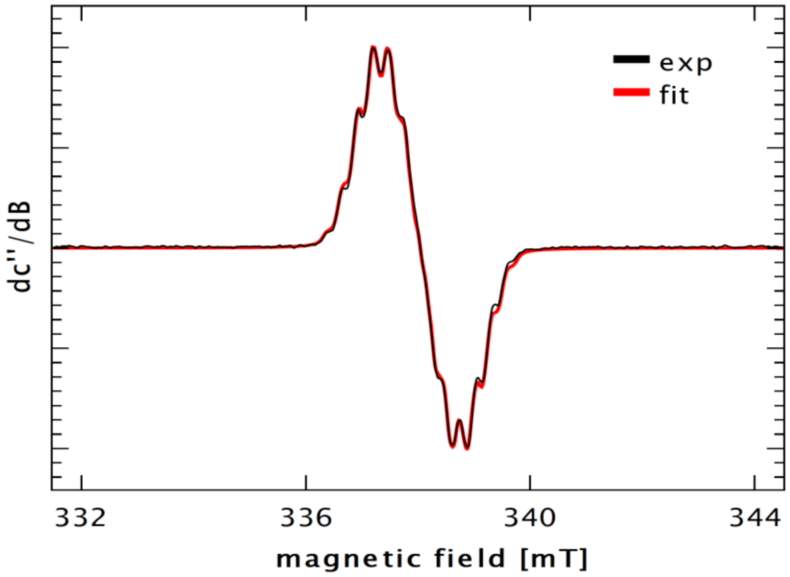

Figure S62: *For* ***9****:* Experimental (black) and simulated (red) continuous-wave (CW) X-band EPR spectra of the liquid phase of the reaction between **1*****tri*** (1.0 equiv.) and 2 di(2-pyridylketone) (2.0 equiv.) in benzene/toluene (1:4) (c(compound **9**) = ca. 0.014 mmol mL^{-1}). The observed resonance shows coupling constants of a(1 × ^{14}N) = 12.9 MHz (4.60 G, 0.460 mT), a(1 × ^{14}N) = 7.11 MHz (2.53 G, 0.253 mT), a(1 × ^{1}H) = 9.86 MHz (3.52 G, 0.352 mT), a(1 × ^{1}H) = 9.55 MHz (3.41 G, 0.341 mT), a(1 × ^{1}H) = 7.42 MHz (2.65 G, 0.265 mT), a(1 × ^{1}H) = 7.02 MHz (2.50 G, 0.250 mT), a(1 × ^{1}H) = 6.87 MHz (2.45 G, 0.245 mT), a(1 × ^{1}H) = 5.40 MHz (1.92 G, 0.192 mT), a(1 × ^{1}H) = 3.02 MHz (1.08 G, 0.108 mT), a(1 × ^{1}H) = 1.95 MHz (0.696 G, 0.0696 mT), and a g_{iso} value of 2.0024. Spectrometer settings: microwave frequency = 9.475395 GHz, 0.0010 mT modulation amplitude at 100 kHz, microwave power = 8.0 mW, temperature = 23 °C, number of accumulated scans = 20, conversion time = 2 ms.

7 EDX analysis

Energy-dispersive X-ray spectroscopy (EDX) was carried out at 5 kV and 2 nA using an Ultim Max detector (Oxford Instruments, Abington, United Kingdom) under high vacuum. For imaging of the area to be examined by EDX, a secondary electron microscope (Crossbeam 550, Zeiss, Oberkochen, Germany) was utilized by applying a voltage of 5 kV and a current of 100 pA using the SE detectors. Therefore, the substance to be analysed was fixed on a carbon-based sample holder and transferred under inert conditions in a sample transfer shuttle (Semilab Conductor Physics Laboratory Co. Ltd., Budapest, Hungary).

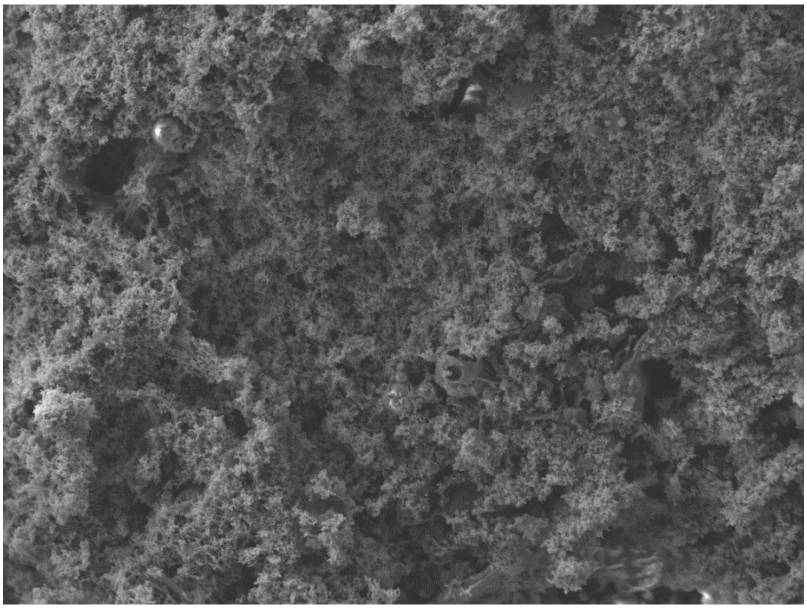

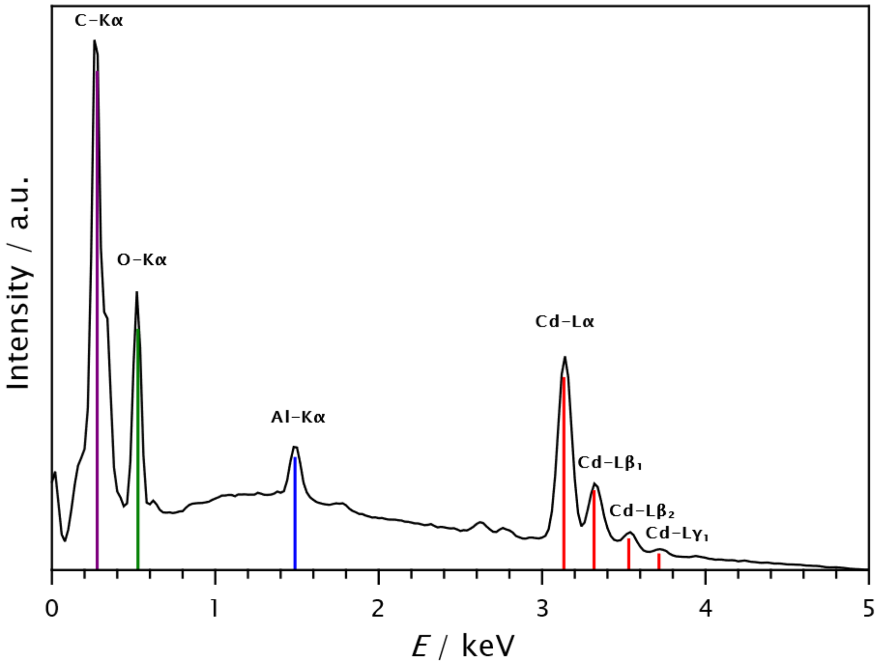

Figure S63: *Top:* SEM-image of the area examined by EDX. Magnification: 1280; FOV: 89 µm. *Bottom:* Recorded EDX-spectrum (black) and theoretical positions of the expected X-ray transition energies for C (violet), O (green), Al (blue) and Cd (red).

Table S1: Sample composition as determined by EDX-analyses. The detection of carbon is due to the carbon-based sample holder.

Element	Mass-%	Atom-%
**C**	3.94	23.21
**O**	3.73	16.51
**Al**	1.08	2.83
**Cd**	91.25	57.46

Note: The theoretical positions of the expected X-ray transition energies were taken from selected literature references.[7,8]

8 X-ray structure elucidation and refinement

X-ray Structure Determination:

X-ray quality crystals were selected in Fomblin YR-1800 perfluoroether (Alfa Aesar) at ambient temperature inside the glovebox. The samples were cooled to 100(2) K during measurement. A Stoe IPDS2 (STOE image plate detector system) with monochromated MoKα (λ = 0.71073 Å; fine focus) radiation or a Stoe StadiVari diffractometer with a DECTRIS PILATUS 200K detector and monochromated CuKα (λ = 1.54186 Å) radiation was employed for a respective measurement. The structures were solved by intrinsic phasing (SHELXT)[9] and refined by full matrix least squares procedures (SHELXL)[10] within the Olex2 platform.[11]

Data reduction was performed using the *X-AREA* program. All absorption corrections have been done by Gaussian integration followed by a scaling of reflection intensities that was done within *STOE LANA*.[12]

All non-hydrogen atoms were refined anisotropically, hydrogen atoms were included in the refinement at calculated positions using a riding model. All special refinement details for disordered structures, molecular structure representations as well as further crystallographic details are summarized down below.

Special Refinement Details:

Compound 2: No special refinement details need to be noted.

Compound 3: We note that the compound crystallizes with two independent molecules in the asymmetric unit. No further special refinement details need to be noted.

Compound 4: We note that the compound crystallizes with half a molecule each in the asymmetric unit (sum: Z' = 1, Z = 4). Each half shows significant disorder. Both, the $\{N(TMS)_2\}^-$ as well as ABNO moieties are disordered for one species whereas the other shows major disorder only for $\{N(TMS)_2\}^-$. To refine this disorder, we had to employ several SADI, ISOR and DELU restraints as well as one EADP constraint (Si1A → Si1). As such we could refine all fragments reasonably including suitable *R*-values (see Table S2). The $\{N(TMS)_2\}^-$ groups were split into two parts each (PART1, PART2, PART5 and PART6) with reasonable occupancies of 0.5 which suits the symmetry generation. The ABNO fragments of one half (attachment to Al2) were as well split into two parts (PART3, PART4) yet a free variable (FVAR2) was assigned. The final occupancies were allowed to refine to a total sum of 1 which yields 0.48 and 0.52. To the overall refinement we also employed a TWIN LAW (1.0, 0.0, 0.0, 0.0, -1.0, 0.0, -1.0, 0.0, -1.0) which allows the absolute structure parameter to refine towards 0 (0.04(2)) and improving reliability factors. The structure representation on this compound (*vide infra*) does, due to the restraints, only give a selection of atom distances.

Compound 5: No special refinement details need to be noted.

Compound 6: We note that the compound crystallizes with two independent molecules in the asymmetric unit. No further special refinement details need to be noted.

Compound 7: No special refinement details need to be noted.

Compound 10: No special refinement details need to be noted.

Molecular Structure Representations:

All molecular structure representations in the ESI as well as the main article have been prepared with the DIAMOND software package.[13] All ellipsoids are represented at the 50% probability level unless stated otherwise.

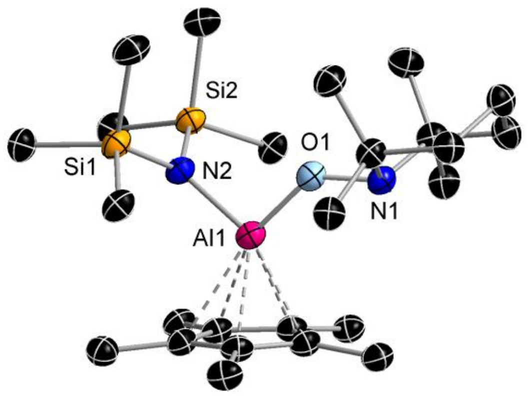

Figure S64: Structure representation of **2**. Selected bond lengths [Å] and angles [°]: Al1-Cp*(centroid) 1.948, Al1-O1 1.740(5), Al1-N2 1.836(4), N1-O1 1.445(6), N2-Si1 1.737(4), N2-Si2 1.737(3), N2-Al1-O1 99.4(1), N1-O1-Al1 137.4(2).

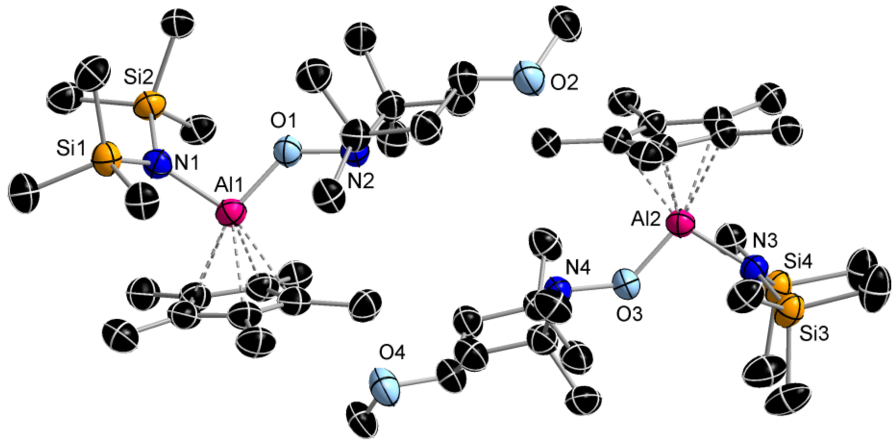

Figure S65: Structure representation of both independent molecules of **3**. Selected bond lengths [Å] and angles [°]: <u>*Molecule 1:*</u> Al1-Cp*(centroid) 1.930, Al1-O1 1.739(2), Al1-N1 1.834(3), N2-O1 1.446(4), N1-Si1 1.727(4), N1-Si2 1.741(3), N1-Al1-O1 98.6(1), N2-O1-Al1 132.8(2); <u>*Molecule 2:*</u> Al2-Cp*(centroid) 1.930, Al2-O3 1.735(3), Al2-N3 1.842(3), N4-O3 1.443(4), N3-Si3 1.733(3), N3-Si4 1.728(3), N3-Al2-O3 98.6(1), N4-O3-Al2 136.5(2).

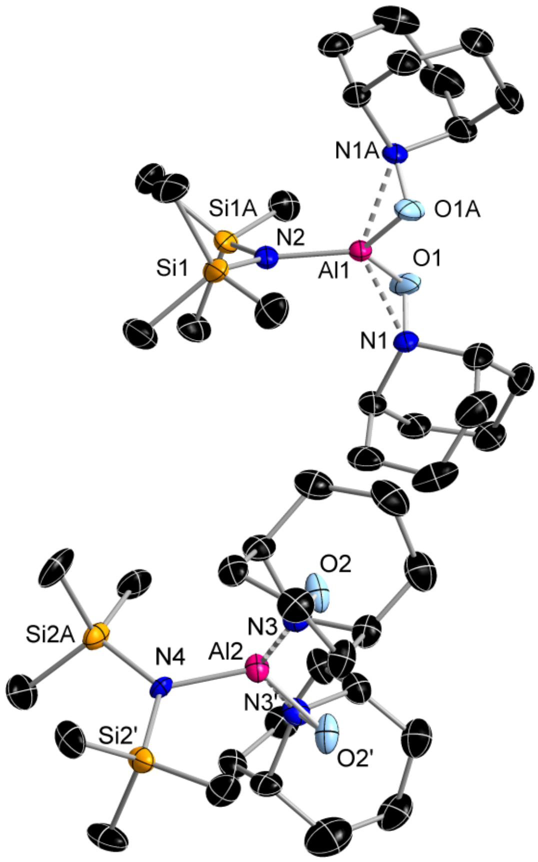

Figure S66: Structure representation of both independent molecules of **4** (30% probability level). Selected bond lengths [Å] and angles [°]: **4**(*): Al1-N2 1.789(9), Al1···N1 1.977(8), Al1-O1 1.755(9), (*) metrics of one independent molecule in the asymmetric unit – symmetry generated atoms created through 1-x, y, 2-z or -x, y, 1-z. A detailed view on atom distances is not provided due to disorder and restraints (see deposited .cif file and Table S2).

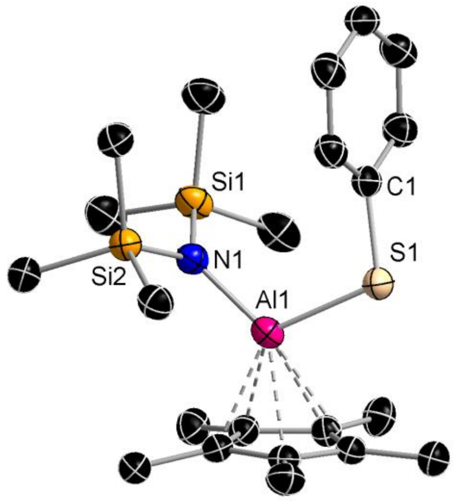

Figure S67: Structure representation of **5**. Selected bond lengths [Å] and angles [°]: Al1-Cp*(centroid) 1.967, Al1-N1 1.817(2), Al1-S1 2.222(1), N1-Si1 1.735(2), N1-Si2 1.733(2), C1-S1 1.777(3), N1-Al1-S1 111.99(8), Si1-N1-Si2 122.5(1), Al1-S1-C1 108.90(9).

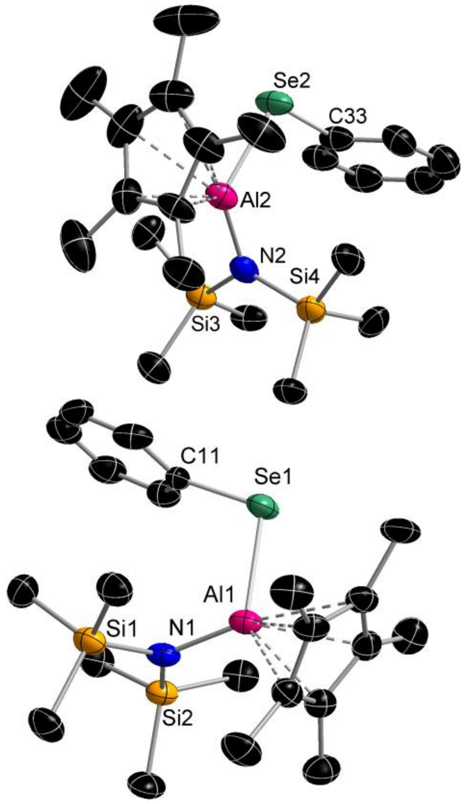

Figure S68:Structure representation of both independent molecules of **6**. Selected bond lengths [Å] and angles [°]: *Molecule 1*: Al1-Cp*(centroid) 1.957, Al1-N1 1.833(5), Al1-Se1 2.360(2), N1-Si1 1.732(6), N1-Si2 1.733(6), C11-Se1 1.930(6), N1-Al1-Se1 112.0(2), Si1-N1-Si2 123.4(3), Al1-Se1-C11 105.8(2). *Molecule 2*: Al2-Cp*(centroid) 1.989, Al2-N2 1.800(6), Al2-Se2 2.347(2), N2-Si3 1.736(6), N2-Si4 1.747(5), C33-Se2 1.927(9), N2-Al2-Se2 115.7(2), Si3-N2-Si4 122.8(3), Al2-Se2-C33 102.4(2).

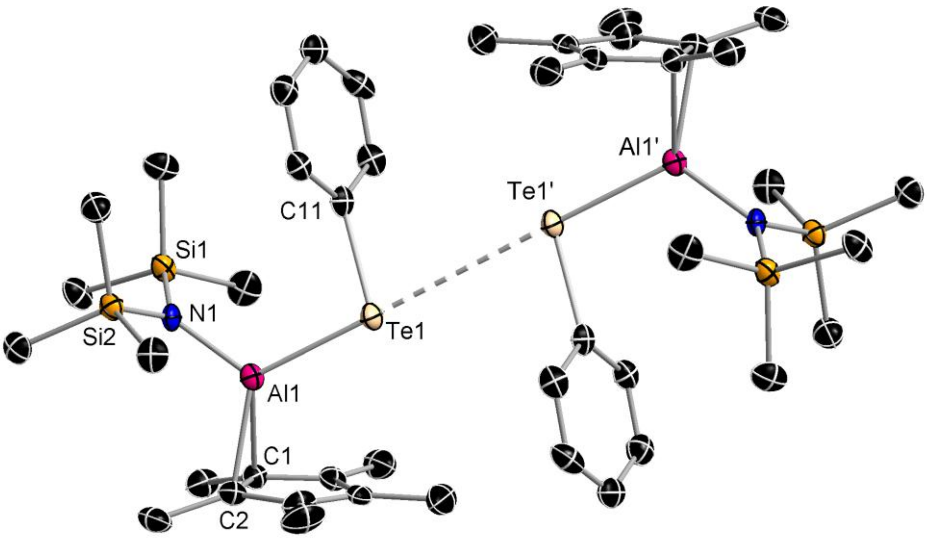

Figure S69: Structure representation of **7** as it's dimer [**7**]$_2$ in the solid state (symmetry generation *via* 2-x, 1-y, 1-z). Selected bond lengths [Å] and angles [°]: Al1-Cp*(centroid) 2.177, Al1-N1 1.812(5), Al1-Te1 2.552(2), Te1···Te1' 3.8633(9) N1-Si1 1.732(5), N1-Si2 1.733(4), C11-Te1 2.136(5), N1-Al1-Se1 117.5(1), Si1-N1-Si2 123.7(2), Al1-Te1-C11 100.3(2).

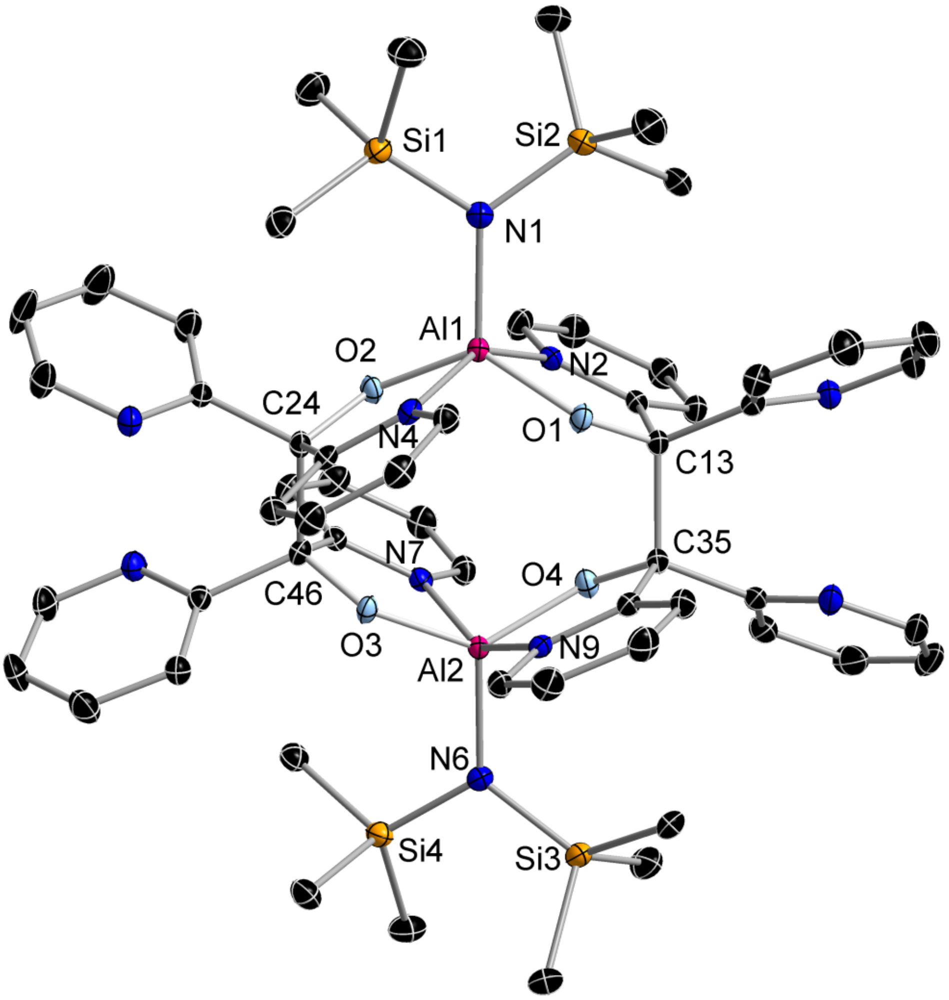

Figure S70: Structure representation of **10**. Selected bond lengths [Å] and angles [°]: Al1-N1 1.885(2), Al1-O1 1.786(1), Al1-N2 2.034(1), Al1-O2 1.783(1), Al1-N4 2.022(2), Al2-N6 1.885(2), Al2-N7 2.021(2), Al2-O3 1.787(1), Al2-N9 2.029(2), Al2-O4 1.789(1), C13-C35 1.641(3), C24-C46 1.635(3), Si1-N1-Si2 112.93(9), O1-Al1-O2 129.50(6), N2-Al1-N4 151.76(7), O1-C13-C35 107.3(1), O2-C24-C46 108.3(1), Si3-N6-Si4 113.61(9), O3-Al2-O4 131.77(6), N7-Al2-N9 151.38(7), O3-C46-C24 108.3(1), O4-C35-C13 106.8(1).

Summary of X-ray Crystallographic Refinement:

Table S2: Crystallographic details #1. * = Restraints were employed to refine disorder.

Compound	***2***	***3***	***4***
Empirical formula	$C_{25}H_{51}AlN_2OSi_2$	$C_{26}H_{53}AlN_2O_2Si_2$	$C_{22}H_{46}AlN_3O_2Si_2$
Formula weight	478.83	508.86	467.78
Temperature/K	100(2)	100(2)	100(2)
Crystal system	triclinic	monoclinic	monoclinic
Space group	*P*–1	$P2_1/c$	*C*2
a/Å	8.7124(6)	15.5390(6)	19.8826(6)
b/Å	9.3436(7)	10.1981(3)	10.2863(3)
c/Å	19.0266(15)	39.1578(16)	16.5219(5)
α/°	77.047(6)	90	90
β/°	89.104(6)	101.267(3)	127.024(2)
γ/°	70.336(6)	90	90
Volume/$Å^3$	1418.40(19)	6085.7(4)	2697.76(15)
Z	2	8	4
$\rho_{calc}g/cm^3$	1.121	1.111	1.152
μ/mm^{-1}	1.564	1.510	1.675
F(000)	528.0	2240.0	1024.0
Crystal size/mm^3	0.08 × 0.06 × 0.03	0.12 × 0.077 × 0.02	0.18 × 0.127 × 0.03
Radiation	CuKα (λ = 1.54186)	CuKα (λ = 1.54186)	CuKα (λ = 1.54186)
2*Θ* range for data collection/°	9.56 to 143.93	4.602 to 132.99	6.702 to 133.908
Index ranges	-10 ≤ h ≤ 10, -10 ≤ k ≤ 11, -23 ≤ l ≤ 17	-15 ≤ h ≤ 18, -12 ≤ k ≤ 11, -33 ≤ l ≤ 46	-17 ≤ h ≤ 23, -10 ≤ k ≤ 12, -19 ≤ l ≤ 18
Reflections collected	13741	26715	13804
Independent reflections	5331 [R_{int} = 0.0750, R_{sigma} = 0.0752]	10567 [R_{int} = 0.0597, R_{sigma} = 0.0684]	4213 [R_{int} = 0.0208, R_{sigma} = 0.0165]
Data/restraints/parameters	5331/0/295	10567/0/627	4213/364/438*
Goodness-of-fit on F^2	1.017	0.986	1.075
Final R indexes [I>=2σ (I)]	R_1 = 0.0797, wR_2 = 0.1974	R_1 = 0.0643, wR_2 = 0.1560	R_1 = 0.0498, wR_2 = 0.1354
Final R indexes [all data]	R_1 = 0.1358, wR_2 = 0.2393	R_1 = 0.1156, wR_2 = 0.1824	R_1 = 0.0507, wR_2 = 0.1366
Largest diff. peak/hole / e $Å^{-3}$	0.51/-0.29	0.44/-0.37	0.25/-0.22
Absolute Structure Parameter	–	–	0.04(2)
CCDC #	2516482	2516483	2516484

Table S3: Crystallographic details #2.

Compound	***5***	***6***	***7***
Empirical formula	$C_{22}H_{38}AlNSSi_2$	$C_{22}H_{38}AlNSi_2Se$	$C_{22}H_{38}AlNSi_2Te$
Formula weight	431.75	478.65	527.29
Temperature/K	100(2)	100(2)	100(2)
Crystal system	monoclinic	triclinic	triclinic
Space group	$P2_1/n$	*P*–1	*P*–1
a/Å	9.7451(5)	9.5257(12)	9.1152(15)
b/Å	16.1889(7)	16.5954(17)	11.564(3)
c/Å	16.4705(8)	16.708(2)	12.5993(17)
α/°	90	97.633(9)	74.993(15)
β/°	104.874(4)	101.799(9)	82.902(12)
γ/°	90	97.348(9)	87.557(17)
Volume/Å^3	2511.4(2)	2529.6(5)	1272.9(4)
Z	4	4	2
ρ_{calc}g/cm^3	1.142	1.257	1.376
μ/mm^{-1}	0.267	3.295	1.305
F(000)	936.0	1008.0	540.0
Crystal size/mm^3	0.13 × 0.107 × 0.06	0.06 × 0.05 × 0.03	0.132 × 0.107 × 0.078
Radiation	MoKα (λ = 0.71073)	CuKα (λ = 1.54186)	MoKα (λ = 0.71073)
2*Θ* range for data collection/°	3.588 to 51.998	7.064 to 144.606	3.37 to 51
Index ranges	-12 ≤ h ≤ 12, -19 ≤ k ≤ 19, -19 ≤ l ≤ 20	-11 ≤ h ≤ 6, -18 ≤ k ≤ 20, -16 ≤ l ≤ 20	-9 ≤ h ≤ 11, -13 ≤ k ≤ 13, -15 ≤ l ≤ 15
Reflections collected	11405	24504	8828
Independent reflections	4905 [R_{int} = 0.0417, R_{sigma} = 0.0456]	9541 [R_{int} = 0.0621, R_{sigma} = 0.0816]	4714 [R_{int} = 0.0530, R_{sigma} = 0.0659]
Data/restraints/parameters	4905/0/255	9541/0/509	4714/0/255
Goodness-of-fit on F^2	1.064	0.993	1.043
Final R indexes [I>=2σ (I)]	R_1 = 0.0486, wR_2 = 0.1270	R_1 = 0.0668, wR_2 = 0.1488	R_1 = 0.0471, wR_2 = 0.0937
Final R indexes [all data]	R_1 = 0.0746, wR_2 = 0.1371	R_1 = 0.1230, wR_2 = 0.1865	R_1 = 0.0746, wR_2 = 0.1017
Largest diff. peak/hole / e Å^{-3}	0.35/-0.30	1.14/-0.66	0.87/-0.82
Absolute Structure Parameter	–	–	–
CCDC #	2516485	2516486	2516487

Table S4: Crystallographic details #3.

Compound	***10***
Empirical formula	$C_{56}H_{68}Al_2N_{10}O_4Si_4$
Formula weight	1111.52
Temperature/K	100
Crystal system	monoclinic
Space group	$P2_1/c$
a/Å	21.3327(4)
b/Å	10.85700(10)
c/Å	25.1255(5)
α/°	90
β/°	95.480(2)
γ/°	90
Volume/Å^3	5792.70(17)
Z	4
ρ_{calc}g/cm^3	1.275
μ/mm^{-1}	1.681
F(000)	2352.0
Crystal size/mm^3	0.12 × 0.08 × 0.05
Radiation	CuKα (λ = 1.54186)
2*Θ* range for data collection/°	4.162 to 140.24
Index ranges	-23 ≤ h ≤ 26, -13 ≤ k ≤ 10, -21 ≤ l ≤ 30
Reflections collected	34975
Independent reflections	10760 [R_{int} = 0.0213, R_{sigma} = 0.0221]
Data/restraints/parameters	10760/0/697
Goodness-of-fit on F^2	1.057
Final R indexes [I>=2σ (I)]	R_1 = 0.0366, wR_2 = 0.0843
Final R indexes [all data]	R_1 = 0.0497, wR_2 = 0.0917
Largest diff. peak/hole / e Å^{-3}	0.36/-0.35
Absolute Structure Parameter	–
CCDC #	2516488

X-ray Powder Diffraction:

X-ray powder diffraction was conducted to further verify the bulk purity of precipitated cadmium metal (*vide infra*). Due to the unavailability of a powder diffractometer at the University of Kassel (Location: AVZ), we have used the above-mentioned Stoe IPDS2 diffractometer (STOE image plate detector system) with monochromated MoKα (λ = 0.71073 Å; fine focus) radiation. We used a representative powder sample of Cadmium which we transferred to a “Marktube” with minimal absorption and placed it onto the goniometer head. All software packages have been the same as mentioned above. The depicted simulated powder diffraction pattern was generated with DIAMOND based on entry 9008490 (Cadmium) using the Crystallography Open Database (see: https://www.crystallography.net/cod/9008490.html).

Cadmium Precipitation and Isolation for pXRD:

In an ampoule with PTFE valve (FengTecEx), **1***tri* was synthesized on a 0.128 mmol scale (based on $Cd\{N(TMS)_2\}_2$; 55.3 mg) in 4 mL of toluene.[5] Next, benzophenone (46.5 mg, 0.256 mmol, 2 eq.) was dissolved in 0.5 mL of toluene and added to the ampoule, leading to the formation of a magenta-colored solution and black precipitates. The magenta-coloured solution was decanted off and the black residue thoroughly washed with *n*-pentane (2x 5 mL n-pentane) until a colorless washing solution is obtained which is also decanted off (we used the highly colored radical **8** here to be able to notice complete extraction of organic material). Then, the black residues were dried through argon flushing using a pipette inside the glovebox. The remaining black precipitates were then transferred to a small vial to afford 12.0 mg of cadmium (83% based on the amount of $Cd\{N(TMS)_2\}_2$ – note that mechanically, not all the cadmium could be transferred to this vial).

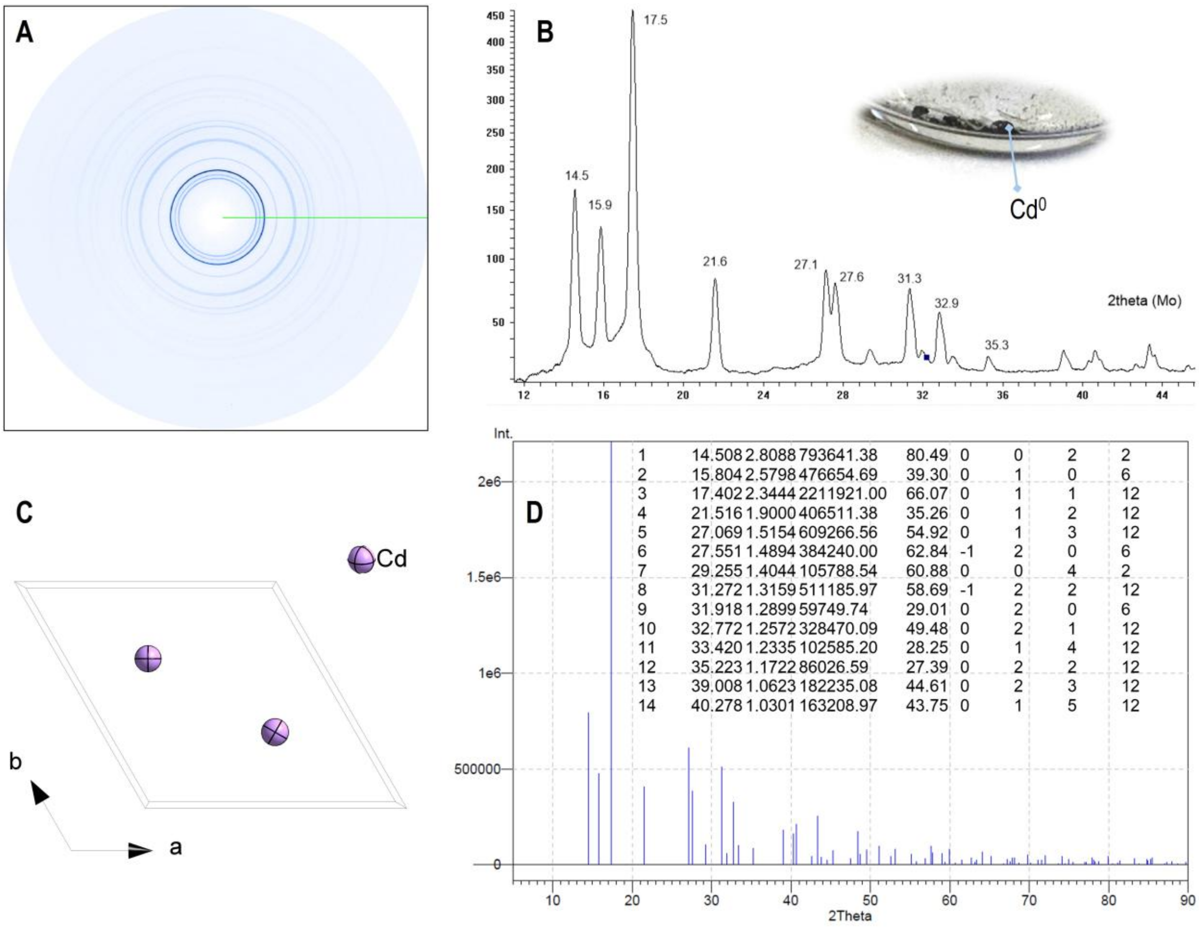

Figure S71: X-ray diffraction analysis on a freshly precipitated cadmium sample: **A)** Illustration of a representative powder frame using MoKα (λ = 0.71073 Å) radiation, **B)** Extracted powder diffractogram including a photograph of the investigated sample, **C)** The illustration of the elemental cell of cadmium (Cd^0) and D) Simulated powder Diffractogram including the first 14 reflections and their 2*Θ* ranges.

9 Computational details

9.1 General computational details

The calculations were performed with and ORCA 5.0.4.[14,15] All structures were verified as true minima by the absence of imaginary eigenvalues in the harmonic vibrational frequency analysis except for transition states which were verified with a single imaginary eigenvalue. We note, though that one very small imaginary eigenvalue could not be suppressed after multiple tries to optimize **IM1B** (–7.8 cm^{-1}) and **Cd_8** (–7.1 cm^{-1}). All geometry optimizations were performed with the r^2SCAN-3c composite electronic-structure method which afforded an excellent fit for the structural parameters for the compounds of interest (see ref.[5]) as well as herein newly experimentally discussed structural parameters, i.e., those found in the solid-state structure of compound **2** (section 9.2).[16] For the fragmentation study, geometry optimizations were performed at the r^2SCAN-3c CPCM(C_6H_6) level of theory. Fragments have been only optimized for H atom positions while constraining the $[Al]^{+/\cdot/-}$ and $[Cp]^{+/\cdot/-}$ parts with a respective charge and multiplicity here ("H opt"). Tighter than default convergence criteria (*tightopt*) were chosen for both, the optimization of the structural parameters as well as the respective single-point calculations (*tightscf*). The energies of all structures were refined through single-point calculations using TPSS[17]/def2-QZVPP[18] including the dispersion correction D4.[19] The RIJCOSX[20–22] approximation in combination with an auxiliary basis set (def2/J[18,23]) was used to speed up the calculations. Implicit correction for solvation effects was conducted for all single point calculations with the CPCM (= conductor-like polarizable continuum) solvation model.[24] Correction for solvation in benzene gives consistent results. Accounting for relativistic effects, effective core potentials (=ECPs)[22] were automatically included in all calculations which contain the element cadmium. The representation of calculated structures as well as (intrinsic) orbitals has been performed with the ChemCraft and IBOview software packages.[25–27] Time dependent DFT spectra have been obtained at different computational methods (see table S6) but most suitably at the TPSS-D4 CPCM(C_6H_6)/def2-QZVPP//r^2SCAN-3c and BP86-D4 CPCM(C_6H_6)/def2-QZVPP//r^2SCAN-3c level of theory.[28] Natural Transition Orbitals were accessed using the "DoNTO true" and extracted with the orca_plot module as .cube files. We assessed the Wiberg Bond Index (Wiberg Bond Order) for compound **10**'s long C-C bond with Multiwfn V.3.8 (depiction in main manuscript only) using an ORCA generated "molden" input file at the r^2SCAN-3c level of theory.[29]

9.2 Benchmarking

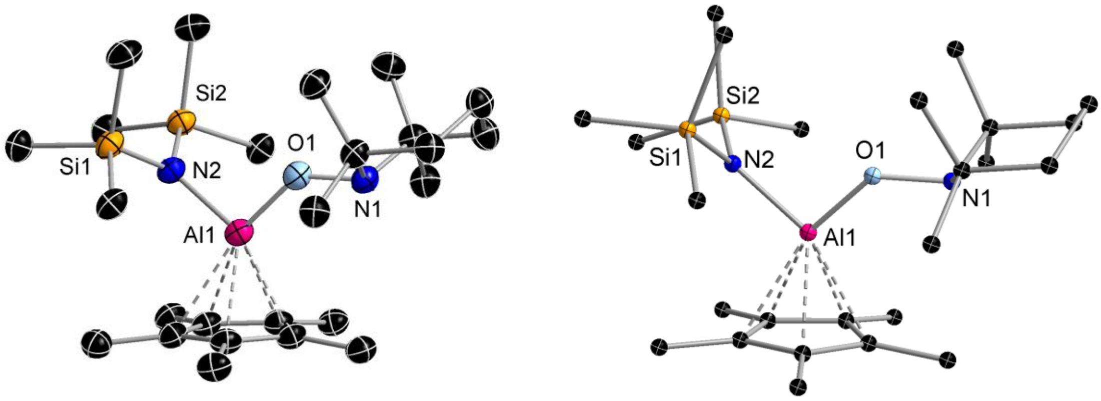

Figure S72: Comparison of structural parameters of **2** as obtained by SC-XRD at 100 K (left) with the calculated structure at the r^{2}SCAN-3c level of theory (right). All H atoms are omitted for clarity. Ellipsoids of the X-ray determined structure are set to a probability level of 50%.

Table S5: Selected atom distances and angles of **1** as obtained by SC-XRD (100 K) and as calculated with the r^{2}SCAN-3c composite method.

**Bond**	**Atom distances [Å] and angles [°]**	
	X-ray	***DFT (r^{2}SCAN-3c)***
Al1-Cp*(centroid)	1.948	1.926
Al1-O1	1.740(5)	1.752
Al1-N2	1.836(4)	1.851
N1-O1	1.445(6)	1.438
N2-Si1	1.737(4)	1.744
N2-Si2	1.737(3)	1.739
N2-Al1-O1	99.4(1)	98.90
N1-O1-Al1	137.4(2)	135.18

Table S6: Benchmarking of various functionals for reproducing experimental data of the visible absorption spectra of compound **8** (TD-DFT benchmarking: top) including Natural Transition Orbitals relevant for visible absorption (NTO analysis: bottom). The solvent oDFB was introduced using the CPCM instruction with EPSILON 14.26 and REFRAC 1.443. Note: Several absorption maxima of different intensities are derived from the computations – in here we only refer to the ones of interest in the VIS region (*vide infra*). The most suitable approaches are marked with " * ". These approaches were then also employed to generate TD DFT spectra for **9** (section 9.3).

Computational Approach	***λ_{max} (exp.) / nm***	***λ_{max} (calc.) ^{a} / nm***
r^{2}SCAN-3c CPCM(oDFB)//r^{2}SCAN-3c		465, 485, **501**
B3LYP-D4 def2-TZVP CPCM(oDFB)//r^{2}SCAN-3c		479, 496
CAM-B3LYP-D4 def2-TZVP CPCM(oDFB)//r^{2}SCAN-3c		449
O3LYP-D4 def2-TZVP CPCM(oDFB)//r^{2}SCAN-3c		514, 526
Pbe0-D4 def2-TZVP CPCM(oDFB)//r^{2}SCAN-3c	<u>*Note:*</u><br>broad absorption<br>-<br>distinct<br>maximum at **554**	479
wB97X-D4 def2-TZVP CPCM(oDFB)//r^{2}SCAN-3c		431
TPSSH-D4 def2-TZVP CPCM(oDFB)//r^{2}SCAN-3c		495, 505
BP86-D4 def2-TZVP CPCM(oDFB)//r^{2}SCAN-3c		515, 540, 553
TPSS-D4 def2-TZVP CPCM(oDFB)//r^{2}SCAN-3c		489, 504, 526
BP86-D4 def2-QZVPP CPCM(oDFB)//r^{2}SCAN-3c *		516, 542, 555
TPSS-D4 def2-QZVPP CPCM(oDFB)//r^{2}SCAN-3c *		492, 505, 529

a = Only suitable absorptions in the visible range are depicted.

Natural Transition Orbital Analysis
(BP86-D4 def2-TZVP CPCM(oDFB)//r^{2}SCAN-3c)

Natural transition orbital (NTO) analysis was performed for the lowest five doublet excited states to characterize their dominant electronic transitions. For **STATE1**, **STATE2**, **STATE3**, and **STATE4**, the excitations are all dominated by the same underlying orbital promotion involving the singly occupied molecular orbital, corresponding to the **136a → 137a** transition. STATE1 and STATE4 are essentially single-configurational in nature, with leading NTO weights of **0.99** and **0.93**, respectively. In contrast, STATE2 and STATE3 show comparable contributions from the α- and β-spin manifolds (α: 136a → 137a; β: 135b → 136b, with weights ≈ 0.5 each), reflecting different TDDFT eigenstates built from the same orbital subspace and leading to a small energetic splitting between these states. The difference in orbital numbering between α and β channels arises from the singly occupied nature of the doublet reference state; the corresponding α and β NTOs are spatially equivalent. As a result, only the α-spin NTOs (136a → 137a) are shown for clarity. STATE5 is qualitatively different and is dominated by a β-spin excitation (135b → 136b, NTO weight 0.998), indicating a distinct electronic character compared to the lower-lying states. NTO Plots (contour value 0.03, visualization through *ChemCraft*):

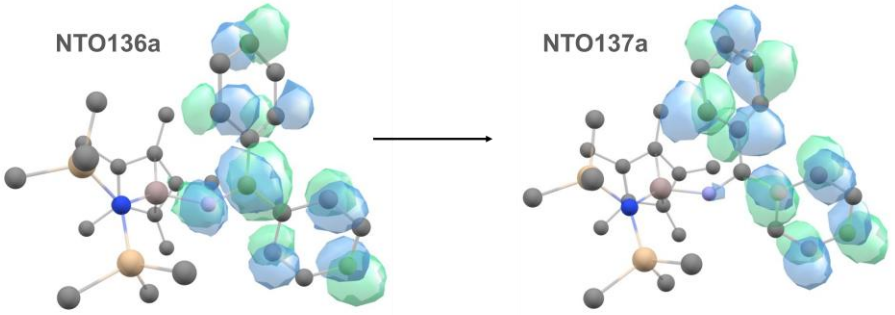

9.3 Representation of simulated absorption spectra

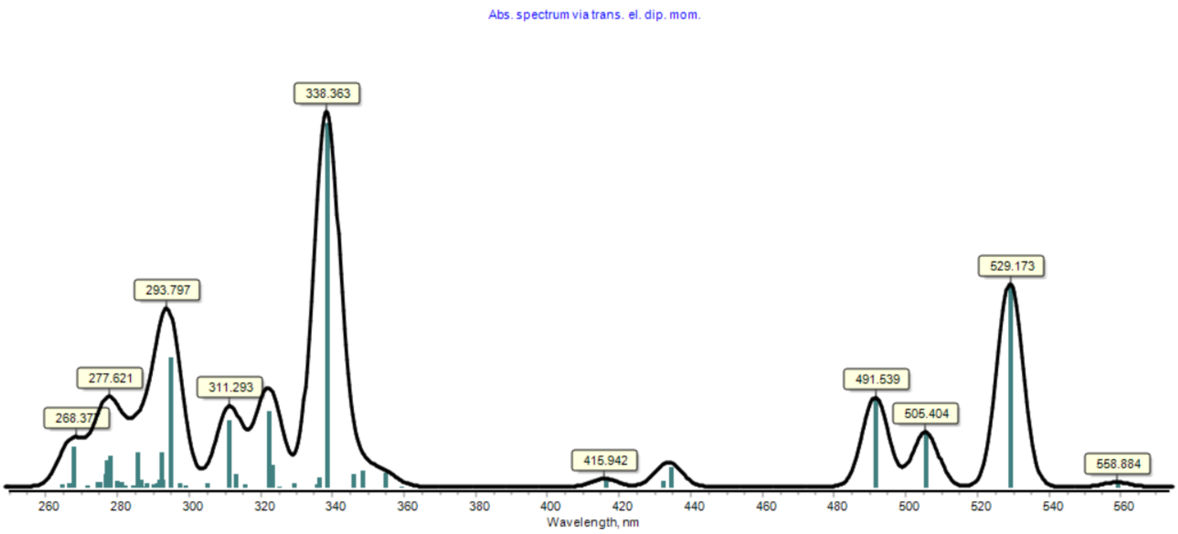

Figure S73: TD-DFT derived absorption of **8** at the TPSS-D4 CPCM(oDFB)/def2-QZVPP//r^{2}SCAN-3c level of theory (visualization through *ChemCraft*).

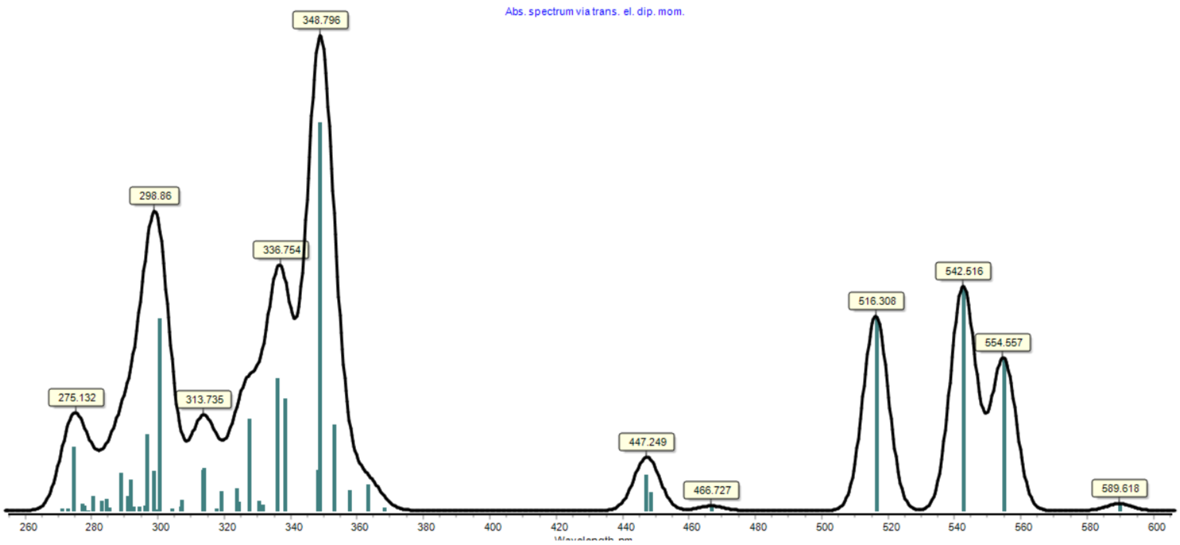

Figure S74: TD-DFT derived absorption of **8** at the BP86-D4 CPCM(C_6H_6)/def2-QZVPP//r^{2}SCAN-3c level of theory (visualization through *ChemCraft*).

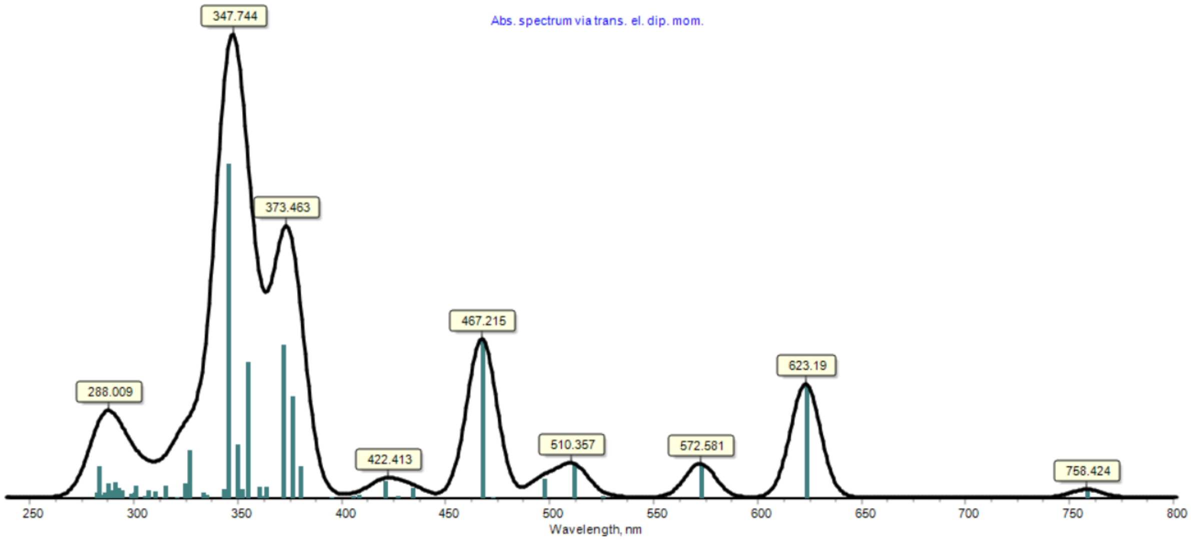

Figure S75: TD-DFT derived absorption of **9** at the TPSS-D4 CPCM(oDFB)/def2-QZVPP//r^{2}SCAN-3c level of theory (visualization through *ChemCraft*).

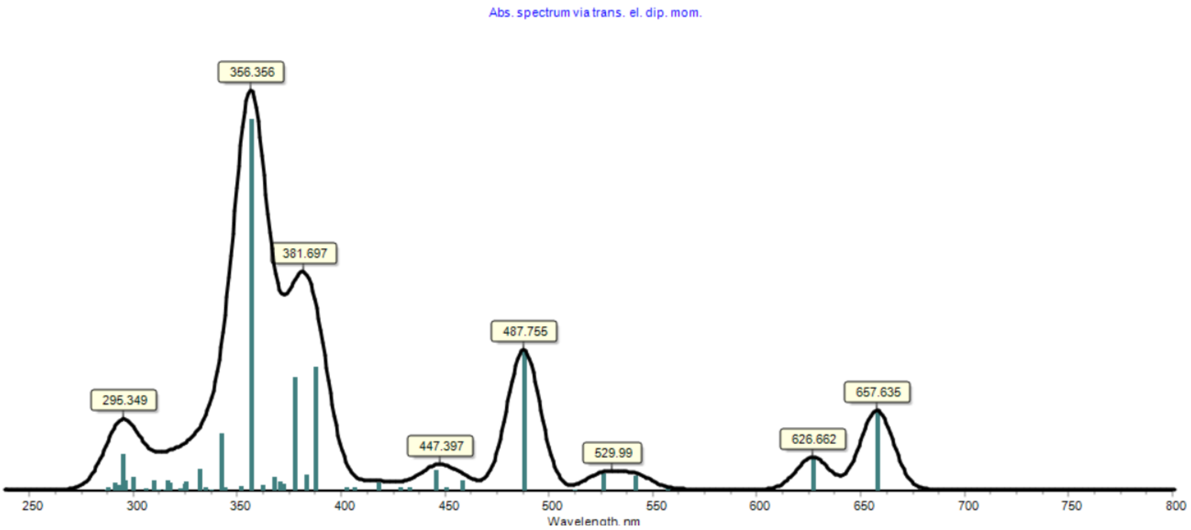

Figure S76: TD-DFT derived absorption of **9** at the BP86-D4 CPCM(C_6H_6)/def2-QZVPP//r^{2}SCAN-3c level of theory (visualization through *ChemCraft*).

9.4 Representation of selected IBOs

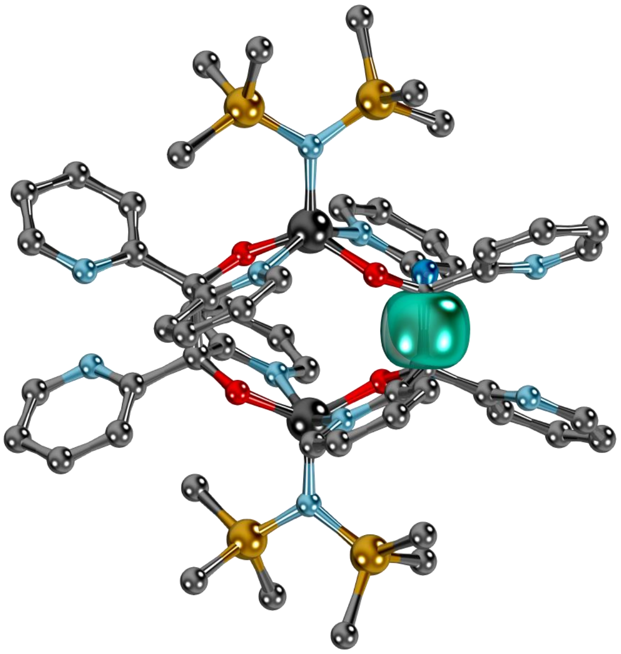

$q_{\sigma\text{-IBO1}}$(C, C) = 0.95, 0.95

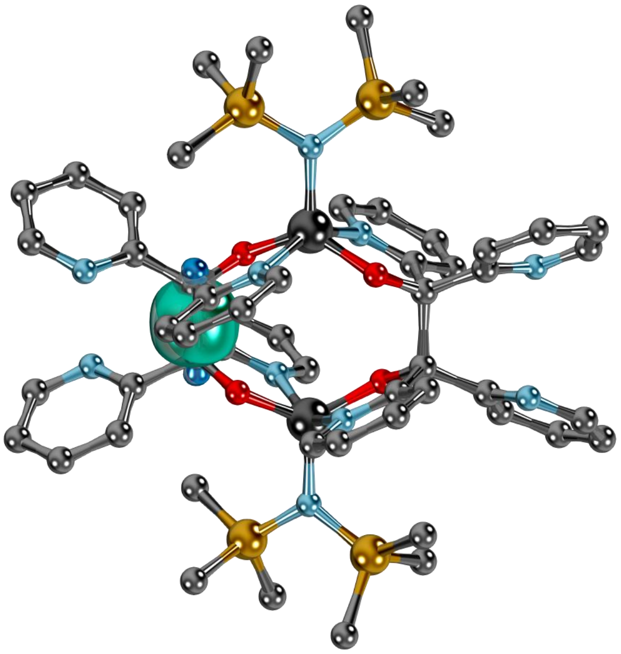

$q_{\sigma\text{-IBO2}}$(C, C) = 0.95, 0.95

Figure S77: Enlarged representation of calculated IBOs (r^{2}SCAN-3c) of **10.**

9.5 Representation of selected frontier orbitals

9.5.1 $[(\{N(TMS)_2\})Al(O–Py_2)]_2$ (10)

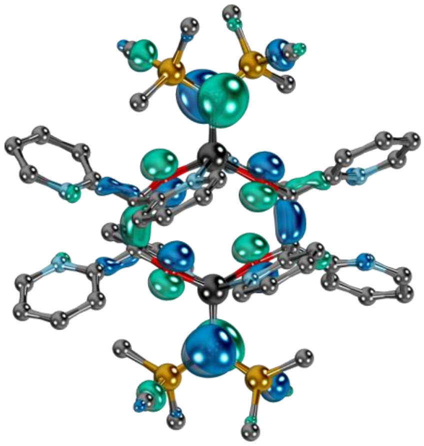

HOMO {–4.9334 eV}

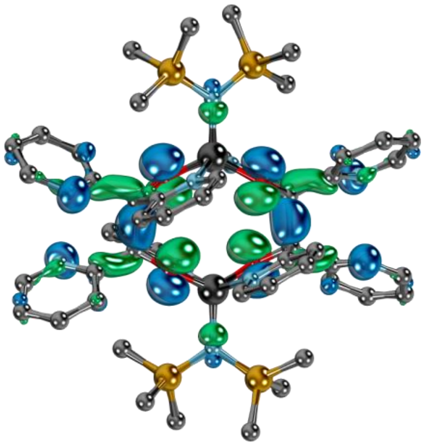

HOMO–2 {–5.3389 eV}

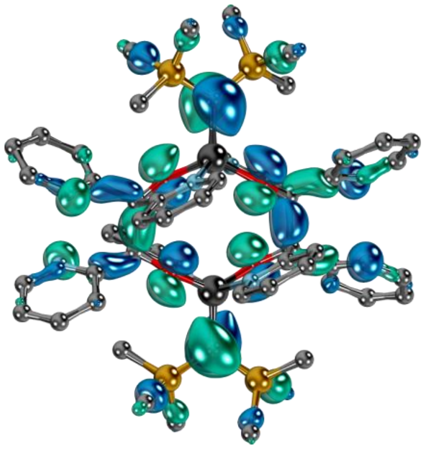

HOMO–3 {–5.3634 eV}

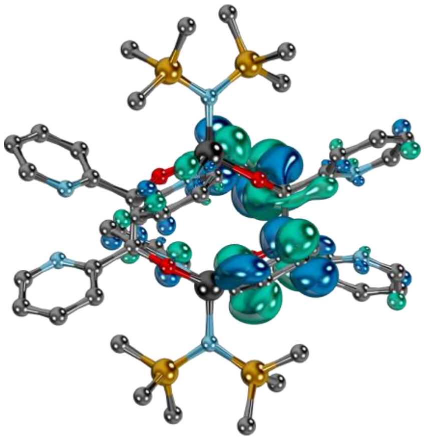

LUMO {–2.0055 eV}

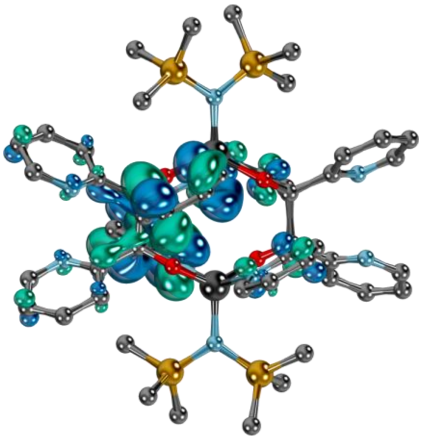

LUMO+1 {–1.9946 eV}

Figure S78: Calculated frontier orbitals of **10** with notable contributions to the C-C bond formation of relevance. Values denoted with { } are derived by single point calculation according to the TPSS-D4 CPCM(oDFB)/def2-TZVP//r^2SCAN-3c level of theory.

9.6 Spin Density Plots

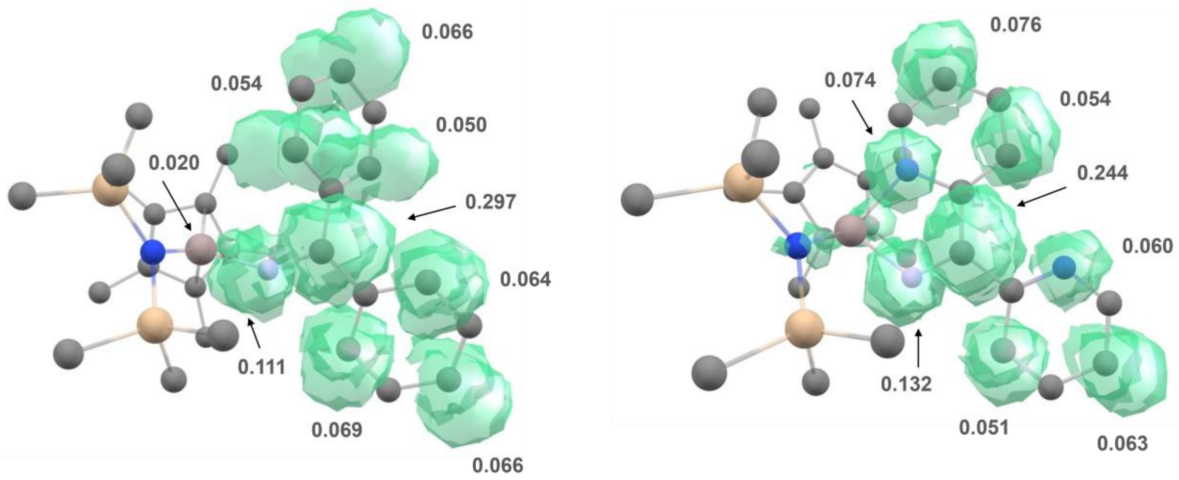

Figure S79: Spin Density Plots of **8** (left; contour value 0.0004) and **9** (right; contour value 0.0006) at the TPSS D4 CPCM(C_6H_6)/def2-QZVPP//r^{2}SCAN-3c level of theory including in the Löwdin spin populations marked in grey Visualization through *ChemCraft*.

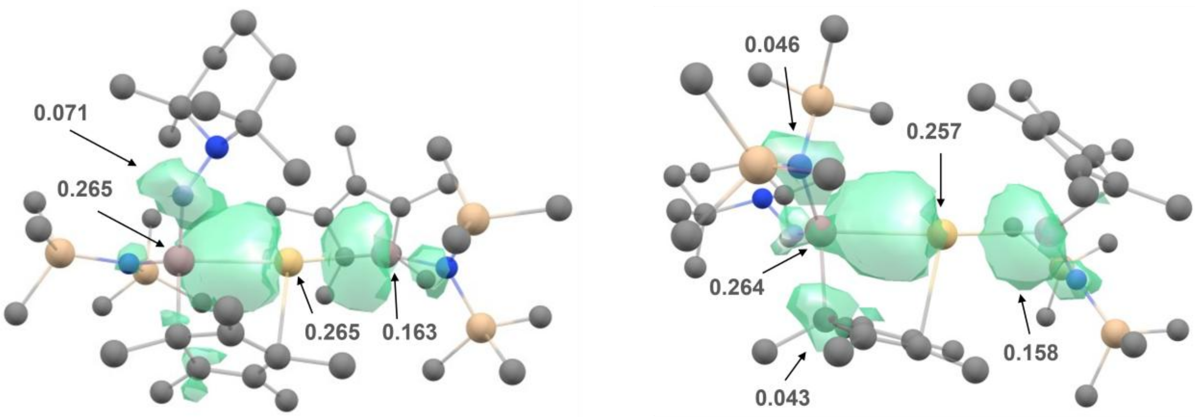

Figure S80: Spin Density Plots of **IM2A** (left; contour value 0.0018) and **IM2B** (right; contour value 0.0018) at the TPSS D4 CPCM(C_6H_6)/def2-QZVPP//r^{2}SCAN-3c level of theory including in the Löwdin spin populations marked in grey. Visualization through *ChemCraft*.

9.7 Energies of optimized structures

Table S7: Energies of the reactants. (d) = calculated on the doublet surface.

Compound	*E* [Eh][a]	*G* [Eh][a]	*E*(SP) [Eh][b]
**1^{tri}**	-3179.2279	-3178.42222	-3180.88075
**[TEMPO]$^{\bullet}$** (d)	-483.59747	-483.37322	-484.05252
**[4-MeO-TEMPO]$^{\bullet}$** (d)	-598.09644	-597.84365	-598.64674
**Benzophenone**	-576.50238	-576.34832	-577.01923
**Di(2-pyridyl) ketone**	-608.56949	-608.43994	-609.10533
**Ph-S-S-Ph**	-1259.53108	-1259.38808	-1260.11274
**Ph-Se-Se-Ph**	-5266.27755	-5266.13774	-5266.71592
**Ph-Te-Te-Ph**	-999.45646	-999.32028	-999.50346
**Cd_8**	-1342.76466	-1342.81511	-1341.78577

[a] r^2SCAN-3c
[b] TPSS D4 def2-QZVPP CPCM(C_6H_6)//r^2SCAN-3c

Table S8: Energies of all relevant species for the fragmentation study of **1^{tri}**.

Compound	*E* [Eh]	*G* [Eh]	*E*(SP) [Eh]
**1^{tri}**	-3179.23577 [a]	-	-3180.88109 [b]
**$[Al]^{-}$**	-1505.70017 [c]	-	-1506.59103 [d]
**[Al]$^{\bullet}$** (d)	-1505.64202 [c]	-	-1506.52227 [d]
**$[Al]^{+}$**	-1505.44223 [c]	-	-1506.32327 [d]
**$[Cd]^{-}$**	-1673.55163 [c]	-	-1674.31800 [d]
**[Cd]$^{\bullet}$** (d)	-1673.50000 [c]	-	-1674.25968 [d]
**$[Cd]^{+}$**	-1673.34153 [c]	-	-1674.10361 [d]

[a] r^2SCAN-3c CPCM(C_6H_6)
[b] TPSS D4 def2-QZVPP CPCM(C_6H_6)//r^2SCAN-3c CPCM(C_6H_6)
[c] r^2SCAN-3c CPCM(C_6H_6)/H-opt
[d] TPSS D4 def2-QZVPP CPCM(C_6H_6)//r^2SCAN-3c CPCM(C_6H_6)/H-opt

Table S9: Energies of ground- and transition states.

Compound	*E* [Eh][a]	*G* [Eh][a]	*E*(SP) [Eh][b]
**IM1A** (d)	-3662.84187	-3661.78206	-3664.94537
**IM1B** (d)	-3662.83463	-3661.77964	-3664.94006
**TS1A ($TS^{IM1A\to IM2A}$)** (d)	-3662.83875	-3661.77735	-3664.94329
**TS1B ($TS^{IM1B\to IM2B}$)** (d)	-3662.81570	-3661.75246	-3664.92178
**IM2A** (d)	-3662.86470	-3661.79960	-3664.97081
**IM2B** (d)	-3662.84788	-3661.78040	-3664.95303
**2**	-1989.34517	-1988.69801	-1990.68436
**3**	-2103.84148	-2103.16556	-2105.27645
**5**	-2135.50301	-2135.02499	-2136.67907
**6**	-4138.87083	-4138.39447	-4139.97629
**7**	-2005.44986	-2004.97414	-2006.36027
**8**	-2082.23433	-2081.65995	-2083.63390
**9**	-2114.35569	-2113.80469	-2115.77313
**10**	-4666.09710	-4665.05279	-4669.22579

[a] r^2SCAN-3c
[b] TPSS D4 def2-QZVPP CPCM(C_6H_6)//r^2SCAN-3c

9.8 XYZ coordinates

107
1^{tri}

Cd	0.449879000	3.120629000	6.602126000
Al	-0.103594000	3.943049000	4.204849000
Al	0.936282000	2.289784000	9.008934000
C	-0.351415000	6.055393000	3.750332000
N	-0.053410000	2.927531000	2.659169000
C	-1.589703000	5.593906000	4.358358000
C	0.523670000	6.424434000	4.814833000
C	0.865874000	0.158980000	9.425527000
N	1.017358000	3.348657000	10.524948000
C	2.221829000	0.495700000	9.010410000
C	0.131361000	-0.150397000	8.238630000
C	-0.236525000	6.420019000	2.297110000
Si	1.485040000	2.736205000	1.867485000
Si	-1.429928000	1.944989000	2.255887000
C	-1.443404000	5.739349000	5.771896000
C	-2.895423000	5.504507000	3.622278000
C	-0.158121000	6.235389000	6.043490000
C	1.910092000	6.972508000	4.679611000
C	0.515705000	-0.251446000	10.829393000
Si	-0.469902000	3.655801000	11.375015000
Si	2.484818000	4.238843000	10.810267000
C	2.266175000	0.370830000	7.592342000
C	3.393571000	0.482690000	9.948922000
C	0.994935000	-0.026028000	7.130495000
C	-1.310060000	-0.551051000	8.189543000
H	0.771253000	6.757274000	2.043354000
H	-0.925539000	7.241613000	2.060408000
H	-0.482585000	5.579122000	1.638065000
C	2.364672000	1.126640000	2.309323000
C	2.656062000	4.110101000	2.417249000
C	1.316794000	2.826798000	-0.007217000
C	-0.983630000	0.163209000	1.827646000
C	-2.581857000	1.850875000	3.750522000
C	-2.379081000	2.627273000	0.775806000
C	-2.481027000	5.384878000	6.790547000
H	-2.794132000	5.008181000	2.653152000
H	-3.291790000	6.511528000	3.430915000
H	-3.652624000	4.963740000	4.197435000
C	0.423357000	6.489069000	7.403296000
H	2.242511000	6.988896000	3.639515000
H	2.643085000	6.388028000	5.248212000
H	1.954539000	8.001374000	5.057968000
H	-0.552686000	-0.456704000	10.934427000
H	1.050775000	-1.172524000	11.097139000
H	0.785369000	0.511911000	11.568270000
C	-1.144445000	5.404976000	11.155773000
C	-1.817488000	2.516437000	10.700915000
C	-0.290582000	3.348000000	13.225353000
C	2.199842000	6.069045000	11.165115000
C	3.562906000	4.166683000	9.258615000
C	3.472697000	3.575743000	12.274517000
C	3.446654000	0.642886000	6.714570000
H	3.133058000	0.914558000	10.918863000
H	3.722747000	-0.549905000	10.130084000
H	4.253474000	1.034844000	9.558417000
C	0.637316000	-0.241104000	5.689341000
H	-1.846893000	-0.228301000	9.085368000
H	-1.825155000	-0.116351000	7.326427000
H	-1.414541000	-1.641641000	8.116347000
H	2.505573000	1.047767000	3.393747000
H	3.358053000	1.105578000	1.844284000
H	1.824115000	0.235158000	1.979194000
H	2.310328000	5.101636000	2.111383000
H	3.640666000	3.948614000	1.961759000
H	2.804434000	4.123216000	3.504915000
H	0.727227000	1.994918000	-0.408566000
H	2.303150000	2.788873000	-0.484864000
H	0.829144000	3.759213000	-0.313904000
H	-0.369325000	0.082960000	0.924306000
H	-1.905101000	-0.403132000	1.644498000
H	-0.447497000	-0.327304000	2.647490000
H	-2.094288000	1.331269000	4.584890000
H	-3.486619000	1.287245000	3.492311000
H	-2.904870000	2.832619000	4.112705000
H	-2.728325000	3.652509000	0.935389000
H	-3.256499000	2.007407000	0.554779000
H	-1.744641000	2.635047000	-0.117655000
H	-2.027914000	5.102567000	7.747663000
H	-3.105057000	4.547875000	6.459904000
H	-3.151471000	6.231601000	6.990748000
H	0.207242000	5.678095000	8.110379000
H	0.024755000	7.411490000	7.846320000
H	1.511396000	6.596635000	7.357893000
H	-1.175219000	5.695083000	10.099418000
H	-2.171356000	5.446273000	11.539624000
H	-0.563022000	6.160826000	11.690519000
H	-1.603439000	1.458582000	10.876600000
H	-2.764841000	2.746345000	11.203560000
H	-1.983426000	2.656241000	9.624551000
H	0.405058000	4.061556000	13.682427000
H	-1.254936000	3.458758000	13.735387000
H	0.086594000	2.339733000	13.430023000
H	1.738501000	6.240259000	12.143843000
H	3.168838000	6.583388000	11.170137000
H	1.571551000	6.544389000	10.404310000
H	3.106666000	4.735218000	8.438799000
H	4.542263000	4.614520000	9.467524000
H	3.743445000	3.149432000	8.892587000
H	3.922196000	2.597952000	12.076998000
H	4.285695000	4.267325000	12.527412000
H	2.836514000	3.474361000	13.160973000
H	3.195152000	1.323613000	5.890675000
H	4.273711000	1.089404000	7.273114000
H	3.817697000	-0.283259000	6.256449000
H	0.750433000	0.666201000	5.079019000
H	1.282675000	-1.003496000	5.234661000
H	-0.396317000	-0.580954000	5.578504000

29
[TEMPO]$^{\bullet}$

C	-5.925274000	1.490195000	0.075317000
C	-5.717807000	3.002162000	0.232922000
N	-4.620568000	0.770742000	0.234410000
C	-6.531853000	1.145211000	-1.294789000
C	-6.859493000	0.985089000	1.178783000
C	-4.525529000	3.532851000	-0.552782000
H	-5.566441000	3.227251000	1.298013000
H	-6.646410000	3.502266000	-0.069195000
C	-3.262609000	2.830164000	-0.071696000
H	-4.431133000	4.614737000	-0.401504000
H	-4.669608000	3.383178000	-1.630051000
C	-3.308964000	1.307406000	-0.251919000
H	-3.120536000	3.053840000	0.994974000
H	-2.375132000	3.204614000	-0.596895000
C	-2.210190000	0.659404000	0.595414000
C	-3.111528000	0.907894000	-1.723516000
H	-6.401362000	1.122180000	2.163330000
H	-7.083094000	-0.075215000	1.047308000
H	-7.794005000	1.555165000	1.145613000
H	-5.980414000	1.600487000	-2.121503000
H	-7.564985000	1.506308000	-1.338745000
H	-6.534792000	0.059969000	-1.431730000
H	-2.387243000	0.841523000	1.660093000
H	-1.243663000	1.095804000	0.322067000
H	-2.174210000	-0.419134000	0.431143000
H	-3.781613000	1.449491000	-2.396227000
H	-3.291946000	-0.164785000	-1.839042000
H	-2.081472000	1.124048000	-2.026884000
O	-4.694025000	-0.499375000	0.355471000

33
[4-MeO-TEMPO]$^{\bullet}$

C	-5.937861000	1.410768000	0.089016000
C	-5.698033000	2.913415000	0.267855000
N	-4.652093000	0.659298000	0.245789000

C	-6.549226000	1.092262000	-1.286084000
C	-6.888428000	0.917381000	1.184313000
C	-4.497883000	3.432464000	-0.500554000
H	-5.535864000	3.136621000	1.330825000
H	-6.600610000	3.452701000	-0.042281000
C	-3.253901000	2.698149000	-0.014726000
O	-4.430175000	4.835659000	-0.245164000
H	-4.636944000	3.276178000	-1.585098000
C	-3.326313000	1.179560000	-0.215719000
H	-3.133702000	2.924279000	1.053538000
H	-2.353814000	3.056286000	-0.528292000
C	-2.250157000	0.503937000	0.639665000
C	-3.112885000	0.791774000	-1.689163000
H	-6.432222000	1.037645000	2.171782000
H	-7.131783000	-0.137137000	1.041489000
H	-7.810797000	1.506407000	1.151254000
H	-5.979699000	1.530823000	-2.109747000
H	-7.568841000	1.488907000	-1.333012000
H	-6.588460000	0.008114000	-1.425842000
H	-2.434268000	0.685373000	1.703095000
H	-1.270919000	0.918329000	0.378327000
H	-2.239422000	-0.574282000	0.469156000
H	-3.773019000	1.339829000	-2.366495000
H	-3.295650000	-0.279461000	-1.813758000
H	-2.078635000	1.006229000	-1.978953000
O	-4.752731000	-0.610622000	0.341710000
C	-3.551423000	5.536575000	-1.109540000
H	-3.662523000	6.598919000	-0.878369000
H	-3.812958000	5.372291000	-2.167358000
H	-2.498289000	5.255989000	-0.960019000

24
Benzophenone

O	2.909736000	2.441400000	3.714794000
C	2.650563000	1.664793000	2.806198000
H	0.394345000	2.818304000	3.579995000
C	0.199146000	1.834153000	3.165117000
C	-1.087344000	1.317946000	3.113827000
H	-1.923328000	1.908487000	3.477201000
C	-1.306544000	0.039038000	2.603853000
H	-2.313166000	-0.368202000	2.572650000
C	-0.234815000	-0.719842000	2.143154000
H	-0.401651000	-1.723822000	1.763970000
C	1.052772000	-0.195169000	2.169267000
H	1.889599000	-0.793503000	1.821083000
C	1.278363000	1.088043000	2.679000000
C	3.704411000	1.286844000	1.817021000
C	3.403831000	0.994514000	0.482260000
H	2.371689000	1.009421000	0.144760000
C	4.425978000	0.722990000	-0.420611000
H	4.188148000	0.515205000	-1.459745000
C	5.750559000	0.719609000	0.006196000
H	6.547174000	0.496433000	-0.697893000
C	6.055936000	1.011745000	1.334706000
H	7.090060000	1.013442000	1.666678000
C	5.040377000	1.308335000	2.231970000
H	5.258381000	1.561472000	3.264998000

22
2-(Pyridyl) ketone

O	2.973093000	2.142663000	3.873302000
C	2.697846000	1.479562000	2.891486000
N	0.320875000	1.793309000	3.064961000
C	-0.932887000	1.352409000	2.976524000
H	-1.710620000	2.056894000	3.270556000
C	-1.270560000	0.070556000	2.546005000
H	-2.310476000	-0.240081000	2.511691000
C	-0.248310000	-0.788774000	2.165765000
H	-0.466729000	-1.797182000	1.826205000
C	1.062329000	-0.331913000	2.215030000
H	1.886467000	-0.964671000	1.906346000
C	1.299459000	0.962316000	2.681956000
C	3.764048000	1.163164000	1.877610000
N	3.377802000	0.746149000	0.663896000
C	4.327663000	0.527821000	-0.247345000

H	3.986933000	0.200327000	-1.229055000
C	5.685732000	0.699578000	0.006435000
H	6.416268000	0.502147000	-0.772280000
C	6.077625000	1.127475000	1.270843000
H	7.128254000	1.271803000	1.505874000
C	5.101338000	1.371885000	2.224289000
H	5.339389000	1.723671000	3.222595000

24
Ph-S-S-Ph

H	0.451135000	0.429045000	1.859896000
C	0.250865000	0.505182000	0.795060000
C	-0.941584000	0.017060000	0.270853000
H	-1.677458000	-0.438617000	0.927093000
C	-1.193985000	0.113920000	-1.096885000
H	-2.126717000	-0.263246000	-1.506293000
C	-0.258331000	0.699627000	-1.940498000
H	-0.444792000	0.779103000	-3.007145000
C	0.940444000	1.185689000	-1.413250000
S	2.168360000	1.940535000	-2.477291000
C	1.196712000	1.087814000	-0.044254000
H	2.136054000	1.461359000	0.352097000
C	6.055399000	-0.302807000	-0.563008000
C	5.522379000	-1.298213000	0.254373000
C	5.329894000	0.173066000	-1.648417000
H	7.042070000	0.101355000	-0.355652000
C	4.260291000	-1.818711000	-0.014205000
S	3.105068000	0.246723000	-3.307536000
C	4.062459000	-0.349354000	-1.915866000
C	3.526086000	-1.346577000	-1.098457000
H	6.093986000	-1.668930000	1.100342000
H	5.734601000	0.950263000	-2.289478000
H	3.842254000	-2.593780000	0.621610000
H	2.537019000	-1.739595000	-1.313886000

24
Ph-Se-Se-Ph

H	0.516326000	0.247895000	1.816681000
C	0.265454000	0.397306000	0.770376000
C	-0.952176000	-0.053540000	0.270872000
H	-1.657235000	-0.554804000	0.927740000
C	-1.268681000	0.138793000	-1.072615000
H	-2.220030000	-0.210581000	-1.463566000
C	-0.371416000	0.782175000	-1.917709000
H	-0.610481000	0.932075000	-2.966004000
C	0.850476000	1.231053000	-1.414335000
Se	2.121774000	2.119532000	-2.562187000
C	1.171314000	1.040066000	-0.069643000
H	2.128474000	1.384102000	0.310144000
C	6.124222000	-0.348204000	-0.555680000
C	5.528009000	-1.257988000	0.314972000
C	5.440391000	0.091878000	-1.683724000
H	7.126523000	0.020099000	-0.356319000
C	4.245009000	-1.730681000	0.056613000
Se	3.177040000	0.213052000	-3.493461000
C	4.153427000	-0.383448000	-1.939808000
C	3.553963000	-1.297463000	-1.071698000
H	6.065800000	-1.599008000	1.194961000
H	5.896614000	0.805619000	-2.362645000
H	3.777171000	-2.440049000	0.733362000
H	2.550272000	-1.656979000	-1.277115000

24
Ph-Te-Te-Ph

H	0.423969000	-0.023657000	1.707436000
C	0.125868000	0.251445000	0.699541000
C	-1.148972000	-0.060707000	0.236711000
H	-1.850143000	-0.580773000	0.883087000
C	-1.525623000	0.295321000	-1.055734000
H	-2.520079000	0.053444000	-1.420195000
C	-0.630567000	0.961244000	-1.888319000
H	-0.922561000	1.231193000	-2.898655000
C	0.646753000	1.274250000	-1.422073000
Te	2.049585000	2.289151000	-2.675890000

C	1.025769000	0.921348000	-0.125654000
H	2.022179000	1.161196000	0.233659000
C	6.328318000	-0.374359000	-0.547236000
C	5.721756000	-1.204744000	0.391770000
C	5.648628000	-0.014436000	-1.707441000
H	7.335141000	-0.003326000	-0.377134000
C	4.432470000	-1.678998000	0.169433000
Te	3.278459000	0.053378000	-3.693827000
C	4.355700000	-0.490476000	-1.928513000
C	3.746855000	-1.326176000	-0.990441000
H	6.255031000	-1.482605000	1.296393000
H	6.119719000	0.639122000	-2.435309000
H	3.955309000	-2.327817000	0.898716000
H	2.738668000	-1.692116000	-1.161146000

8
Cd_8

Cd	-0.422675000	1.859115000	0.847435000
Cd	1.826402000	0.663070000	4.758888000
Cd	-1.939568000	4.599851000	1.507965000
Cd	0.175915000	3.278098000	3.542352000
Cd	2.802950000	1.881451000	2.075309000
Cd	4.918416000	0.559688000	4.109690000
Cd	1.152461000	4.496498000	0.858769000
Cd	3.401560000	3.300448000	4.770191000

107
1^{tri} (CPCM C_6H_6)

Cd	0.460568000	3.132514000	6.597641000
Al	-0.111411000	3.952620000	4.199343000
Al	0.961048000	2.273746000	8.997634000
C	-0.359335000	6.066439000	3.714781000
N	-0.065928000	2.922598000	2.659893000
C	-1.592996000	5.604263000	4.330034000
C	0.524493000	6.428602000	4.772483000
C	0.889452000	0.140285000	9.420852000
N	1.019499000	3.323100000	10.525447000
C	2.244310000	0.478501000	9.002124000
C	0.149679000	-0.162277000	8.235890000
C	-0.246545000	6.421489000	2.259375000
Si	1.467637000	2.730370000	1.858755000
Si	-1.445326000	1.937086000	2.271187000
C	-1.438707000	5.748118000	5.745313000
C	-2.902718000	5.515095000	3.600198000
C	-0.149005000	6.238584000	6.007760000
C	1.911914000	6.973666000	4.638243000
C	0.543005000	-0.266920000	10.826572000
Si	-0.478901000	3.617912000	11.361816000
Si	2.484693000	4.204420000	10.848888000
C	2.283600000	0.359258000	7.582253000
C	3.420196000	0.454256000	9.935973000
C	1.010046000	-0.033350000	7.124211000
C	-1.292579000	-0.561254000	8.184750000
H	0.760346000	6.759889000	2.003422000
H	-0.940361000	7.237666000	2.017545000
H	-0.490868000	5.575251000	1.606556000
C	2.355901000	1.126013000	2.305817000
C	2.642602000	4.107136000	2.391593000
C	1.285865000	2.805746000	-0.015640000
C	-1.002640000	0.150941000	1.856116000
C	-2.593392000	1.858501000	3.769458000
C	-2.400331000	2.603094000	0.786677000
C	-2.477395000	5.383556000	6.760076000
H	-2.802977000	5.023262000	2.628529000
H	-3.301870000	6.522044000	3.413336000
H	-3.655652000	4.969740000	4.176634000
C	0.469632000	6.477272000	7.352717000
H	2.238787000	7.008775000	3.596603000
H	2.643899000	6.376493000	5.195570000
H	1.961993000	7.994433000	5.038244000
H	-0.525700000	-0.467966000	10.936548000
H	1.077962000	-1.188219000	11.094634000
H	0.818094000	0.497319000	11.562582000
C	-1.153387000	5.368992000	11.157571000
C	-1.819327000	2.487206000	10.661415000
C	-0.321960000	3.290179000	13.211118000
C	2.204802000	6.041194000	11.174347000
C	3.612655000	4.110080000	9.334553000
C	3.418764000	3.551405000	12.352584000
C	3.456870000	0.646785000	6.699209000
H	3.179354000	0.915350000	10.897653000
H	3.719560000	-0.583212000	10.140488000
H	4.292405000	0.971449000	9.526206000
C	0.640430000	-0.230530000	5.683684000
H	-1.813894000	-0.303302000	9.110071000
H	-1.822557000	-0.072152000	7.359960000
H	-1.396435000	-1.644641000	8.038769000
H	2.535261000	1.075403000	3.386568000
H	3.331515000	1.092107000	1.804710000
H	1.798754000	0.230226000	2.016893000
H	2.294241000	5.098527000	2.087841000
H	3.622052000	3.941159000	1.925937000
H	2.799627000	4.120924000	3.478216000
H	0.691699000	1.970447000	-0.403441000
H	2.270233000	2.763197000	-0.497564000
H	0.796361000	3.736705000	-0.324735000
H	-0.386595000	0.066342000	0.954100000
H	-1.926938000	-0.412200000	1.675778000
H	-0.468273000	-0.334943000	2.680117000
H	-2.102197000	1.348957000	4.608157000
H	-3.496424000	1.289244000	3.516420000
H	-2.917517000	2.844219000	4.120292000
H	-2.729540000	3.637648000	0.928885000
H	-3.290157000	1.991317000	0.593018000
H	-1.775671000	2.577744000	-0.113596000
H	-2.027179000	5.096318000	7.716693000
H	-3.094328000	4.545340000	6.419119000
H	-3.154670000	6.224584000	6.961503000
H	-0.004242000	5.876146000	8.135407000
H	0.385936000	7.529385000	7.657811000
H	1.537525000	6.229792000	7.353097000
H	-1.202425000	5.657205000	10.101225000
H	-2.173519000	5.407751000	11.560043000
H	-0.560125000	6.122281000	11.683196000
H	-1.609686000	1.427509000	10.831995000
H	-2.771570000	2.718359000	11.154784000
H	-1.968593000	2.636462000	9.583768000
H	0.359255000	4.006952000	13.684829000
H	-1.296700000	3.384855000	13.705081000
H	0.063264000	2.283007000	13.407506000
H	1.716533000	6.225832000	12.137515000
H	3.178040000	6.547405000	11.198543000
H	1.600149000	6.508503000	10.389161000
H	3.188377000	4.677546000	8.497063000
H	4.586944000	4.553210000	9.575863000
H	3.797398000	3.088915000	8.982469000
H	3.790127000	2.531395000	12.213263000
H	4.282303000	4.193766000	12.566249000
H	2.775676000	3.553109000	13.239942000
H	3.190973000	1.333734000	5.884828000
H	4.283448000	1.094766000	7.257586000
H	3.831297000	-0.271685000	6.228220000
H	0.793958000	0.674210000	5.079011000
H	1.250180000	-1.019301000	5.224081000
H	-0.408541000	-0.520685000	5.574285000

53
[Al]⁻ (H Opt)

Al	0.937565000	2.288917000	9.008598000
C	0.862644000	0.158856000	9.428184000
N	1.018591000	3.349839000	10.523193000
C	2.219779000	0.492459000	9.014388000
C	0.129143000	-0.150879000	8.240752000
C	0.509842000	-0.248890000	10.832167000
Si	-0.469228000	3.660967000	11.370821000
Si	2.487319000	4.237708000	10.809185000
C	2.265787000	0.365454000	7.596561000
C	3.390241000	0.478635000	9.954480000
C	0.994429000	-0.029716000	7.133588000
C	-1.312951000	-0.548926000	8.190317000

H	-0.555951000	-0.477606000	10.928730000
H	1.063152000	-1.151667000	11.130440000
H	0.744986000	0.533129000	11.567347000
C	-1.140227000	5.411076000	11.148152000
C	-1.818031000	2.523133000	10.696580000
C	-0.292946000	3.355515000	13.221842000
C	2.205265000	6.068955000	11.161008000
C	3.567340000	4.161301000	9.259081000
C	3.472014000	3.574898000	12.275709000
C	3.447928000	0.634053000	6.719975000
H	3.124844000	0.908788000	10.925184000
H	3.733064000	-0.550435000	10.141675000
H	4.247330000	1.041943000	9.569548000
C	0.638331000	-0.246209000	5.692274000
H	-1.814402000	-0.356123000	9.142844000
H	-1.858008000	0.007102000	7.417920000
H	-1.427161000	-1.619304000	7.965784000
H	-1.145951000	5.676838000	10.084360000
H	-2.174255000	5.460560000	11.514061000
H	-0.559209000	6.168176000	11.682823000
H	-1.570049000	1.465740000	10.821783000
H	-2.762408000	2.712767000	11.226481000
H	-1.975581000	2.702514000	9.626821000
H	0.418796000	4.058932000	13.671287000
H	-1.251516000	3.471636000	13.745324000
H	0.078460000	2.341671000	13.412628000
H	1.723528000	6.239003000	12.130619000
H	3.171337000	6.590825000	11.176879000
H	1.583522000	6.530417000	10.386248000
H	3.074478000	4.679952000	8.429823000
H	4.533812000	4.649854000	9.452368000
H	3.766265000	3.139283000	8.922924000
H	3.842430000	2.560480000	12.097710000
H	4.340132000	4.213356000	12.489460000
H	2.847288000	3.546996000	13.176231000
H	3.223222000	1.401505000	5.967446000
H	4.307385000	0.983215000	7.300643000
H	3.757437000	-0.270546000	6.178127000
H	0.460991000	0.702611000	5.165156000
H	1.444925000	-0.766357000	5.161242000
H	-0.270311000	-0.849742000	5.594300000

53
[Al]$^{\cdot}$ (H Opt)

Al	0.936553000	2.290224000	9.008482000
C	0.863506000	0.159400000	9.424508000
N	1.018413000	3.348615000	10.524802000
C	2.219989000	0.494631000	9.009892000
C	0.128997000	-0.148807000	8.237302000
C	0.512421000	-0.250981000	10.828154000
Si	-0.468746000	3.657285000	11.374484000
Si	2.486832000	4.236992000	10.810804000
C	2.264628000	0.370076000	7.591803000
C	3.391424000	0.479998000	9.948759000
C	0.993064000	-0.025167000	7.129460000
C	-1.312878000	-0.547747000	8.187665000
H	-0.557515000	-0.446664000	10.935026000
H	1.040737000	-1.176839000	11.094344000
H	0.790090000	0.507566000	11.568899000
C	-1.141158000	5.407315000	11.155491000
C	-1.817469000	2.519687000	10.699685000
C	-0.290357000	3.348795000	13.224800000
C	2.203898000	6.067445000	11.166041000
C	3.565314000	4.163965000	9.259466000
C	3.473480000	3.572350000	12.275185000
C	3.445688000	0.640973000	6.714467000
H	3.129905000	0.903249000	10.921914000
H	3.724073000	-0.552950000	10.123526000
H	4.249072000	1.035973000	9.558552000
C	0.635634000	-0.239443000	5.688144000
H	-1.835891000	-0.277411000	9.108518000
H	-1.839069000	-0.069092000	7.355142000
H	-1.416333000	-1.633188000	8.056680000
H	-1.157604000	5.695954000	10.098010000
H	-2.172589000	5.440477000	11.527839000
H	-0.566047000	6.159751000	11.702020000
H	-1.602446000	1.460598000	10.866299000
H	-2.759738000	2.748913000	11.212730000
H	-1.990822000	2.669854000	9.626045000
H	0.408801000	4.058406000	13.682648000
H	-1.259411000	3.469556000	13.723748000
H	0.075928000	2.336620000	13.431043000
H	1.734183000	6.237264000	12.141108000
H	3.177195000	6.573637000	11.178996000
H	1.583956000	6.544689000	10.399842000
H	3.096895000	4.708391000	8.430517000
H	4.532366000	4.639629000	9.465221000
H	3.773582000	3.144352000	8.915465000
H	3.894593000	2.579683000	12.089804000
H	4.306020000	4.251615000	12.496358000
H	2.848523000	3.508714000	13.173286000
H	3.166012000	1.227660000	5.832118000
H	4.229399000	1.185433000	7.248713000
H	3.887155000	-0.296421000	6.350051000
H	0.635111000	0.699266000	5.117928000
H	1.355836000	-0.908415000	5.202661000
H	-0.355780000	-0.689218000	5.586624000

53
[Al]$^{+}$ (H Opt)

Al	0.935887000	2.290815000	9.009291000
C	0.864461000	0.159468000	9.422910000
N	1.018338000	3.347501000	10.526768000
C	2.220405000	0.495997000	9.007584000
C	0.129176000	-0.147808000	8.235943000
C	0.514760000	-0.252748000	10.826363000
Si	-0.468317000	3.654291000	11.378011000
Si	2.486453000	4.236433000	10.812609000
C	2.263970000	0.373110000	7.589316000
C	3.392609000	0.480985000	9.945484000
C	0.992270000	-0.022366000	7.127545000
C	-1.312499000	-0.547560000	8.187011000
H	-0.557664000	-0.418647000	10.941987000
H	1.018342000	-1.201871000	11.056715000
H	0.838304000	0.477988000	11.573398000
C	-1.141961000	5.404167000	11.161587000
C	-1.816901000	2.516661000	10.702987000
C	-0.288244000	3.343768000	13.227823000
C	2.202704000	6.066302000	11.170193000
C	3.563721000	4.165851000	9.260316000
C	3.474688000	3.570692000	12.275421000
C	3.444155000	0.645734000	6.711338000
H	3.133115000	0.888810000	10.924249000
H	3.717423000	-0.557509000	10.099833000
H	4.248980000	1.032235000	9.549497000
C	0.633802000	-0.235189000	5.686272000
H	-1.848515000	-0.242046000	9.088414000
H	-1.825398000	-0.114062000	7.323841000
H	-1.401954000	-1.638274000	8.104749000
H	-1.176636000	5.703773000	10.107654000
H	-2.168432000	5.421196000	11.546781000
H	-0.568996000	6.156684000	11.709197000
H	-1.649838000	1.459115000	10.923813000
H	-2.764027000	2.802722000	11.175664000
H	-1.976387000	2.626512000	9.618881000
H	0.384584000	4.068062000	13.699508000
H	-1.279094000	3.463416000	13.681269000
H	0.075537000	2.336435000	13.455870000
H	1.744998000	6.238964000	12.149970000
H	3.183391000	6.557205000	11.177195000
H	1.585723000	6.556248000	10.409988000
H	3.108563000	4.728676000	8.434474000
H	4.529878000	4.636581000	9.477173000
H	3.783812000	3.149476000	8.909219000
H	3.916388000	2.585865000	12.101379000
H	4.292204000	4.278195000	12.458541000
H	2.869875000	3.518379000	13.186985000
H	3.149478000	1.158954000	5.790716000
H	4.200869000	1.249296000	7.218940000
H	3.922303000	-0.296818000	6.414561000

H	0.721447000	0.687536000	5.099310000
H	1.305209000	-0.969935000	5.228242000
H	-0.387533000	-0.606606000	5.578283000

54
[Cd]⁻ (H Opt)

Cd	0.458178000	3.121514000	6.600347000
Al	-0.101836000	3.943443000	4.204419000
C	-0.356651000	6.055261000	3.751343000
N	-0.051331000	2.928812000	2.658166000
C	-1.592529000	5.589850000	4.361281000
C	0.519171000	6.426368000	4.814513000
C	-0.245347000	6.420920000	2.298103000
Si	1.486309000	2.742396000	1.863748000
Si	-1.425648000	1.942418000	2.256783000
C	-1.444224000	5.735047000	5.774637000
C	-2.899247000	5.496959000	3.627406000
C	-0.159943000	6.234737000	6.044254000
C	1.903738000	6.978589000	4.677172000
H	0.751169000	6.801127000	2.055564000
H	-0.967950000	7.207152000	2.037678000
H	-0.438887000	5.564590000	1.637549000
C	2.371429000	1.135211000	2.303289000
C	2.654228000	4.119464000	2.412154000
C	1.314565000	2.833384000	-0.010626000
C	-0.974847000	0.162163000	1.826918000
C	-2.574720000	1.844207000	3.753347000
C	-2.379348000	2.622607000	0.778667000
C	-2.479051000	5.377040000	6.794900000
H	-2.794616000	5.003735000	2.656001000
H	-3.307452000	6.500651000	3.436126000
H	-3.651132000	4.945685000	4.200929000
C	0.423130000	6.489479000	7.403183000
H	2.231019000	7.006142000	3.634899000
H	2.636259000	6.385424000	5.238997000
H	1.952878000	8.003083000	5.070517000
H	2.419716000	1.023539000	3.393382000
H	3.399312000	1.150261000	1.918993000
H	1.873092000	0.249620000	1.897290000
H	2.279039000	5.111622000	2.144338000
H	3.631840000	3.988391000	1.929019000
H	2.809081000	4.101629000	3.497842000
H	0.710418000	2.007577000	-0.404095000
H	2.296683000	2.788398000	-0.499204000
H	0.830399000	3.771333000	-0.307583000
H	-0.359688000	0.088524000	0.923000000
H	-1.891535000	-0.415294000	1.650125000
H	-0.429189000	-0.315337000	2.648350000
H	-2.055082000	1.388965000	4.605625000
H	-3.449907000	1.223566000	3.519634000
H	-2.938849000	2.824331000	4.076411000
H	-2.701045000	3.656972000	0.940095000
H	-3.273923000	2.021864000	0.567912000
H	-1.749713000	2.612017000	-0.118745000
H	-2.022247000	5.009427000	7.719784000
H	-3.142080000	4.586400000	6.427337000
H	-3.110739000	6.239351000	7.052383000
H	-0.162215000	6.008552000	8.191153000
H	0.464899000	7.566533000	7.623764000
H	1.446060000	6.102710000	7.484139000

54
[Cd]• (H Opt)

Cd	0.458976000	3.121718000	6.600665000
Al	-0.101575000	3.943205000	4.204711000
C	-0.357087000	6.054893000	3.751425000
N	-0.050947000	2.928413000	2.658567000
C	-1.592751000	5.589176000	4.361564000
C	0.518750000	6.426387000	4.814448000
C	-0.246069000	6.420420000	2.298130000
Si	1.486655000	2.742375000	1.863986000
Si	-1.425011000	1.941555000	2.257461000
C	-1.444320000	5.734578000	5.774885000
C	-2.899527000	5.495804000	3.627856000
C	-0.160158000	6.234690000	6.044291000
C	1.903132000	6.979014000	4.676877000
H	0.760726000	6.759225000	2.042672000
H	-0.938697000	7.239800000	2.063658000
H	-0.493138000	5.580118000	1.638913000
C	2.372317000	1.135509000	2.303603000
C	2.654220000	4.119860000	2.412095000
C	1.314658000	2.833096000	-0.010378000
C	-0.973720000	0.161388000	1.827744000
C	-2.573875000	1.843165000	3.754174000
C	-2.379096000	2.621286000	0.779382000
C	-2.478915000	5.376373000	6.795313000
H	-2.793897000	5.010960000	2.653500000
H	-3.302803000	6.501983000	3.446215000
H	-3.650618000	4.943013000	4.199216000
C	0.422999000	6.489764000	7.403122000
H	2.237730000	6.991481000	3.637488000
H	2.634044000	6.402830000	5.256637000
H	1.940826000	8.009829000	5.052182000
H	2.441335000	1.018338000	3.391989000
H	3.393172000	1.154372000	1.902641000
H	1.871125000	0.249847000	1.902288000
H	2.300587000	5.112315000	2.118423000
H	3.632898000	3.963253000	1.941686000
H	2.816402000	4.124231000	3.497928000
H	0.719784000	2.003399000	-0.408639000
H	2.302986000	2.788922000	-0.483854000
H	0.832800000	3.768877000	-0.316762000
H	-0.362931000	0.086141000	0.921400000
H	-1.896381000	-0.404769000	1.649086000
H	-0.431768000	-0.326757000	2.645102000
H	-2.089213000	1.311465000	4.582519000
H	-3.480935000	1.286149000	3.488892000
H	-2.892832000	2.823613000	4.124237000
H	-2.728938000	3.646413000	0.939515000
H	-3.255941000	1.996774000	0.568424000
H	-1.748457000	2.625052000	-0.117008000
H	-2.026888000	5.030906000	7.730541000
H	-3.139686000	4.581793000	6.433982000
H	-3.112720000	6.239591000	7.039597000
H	-0.138001000	5.978154000	8.190118000
H	0.416628000	7.561767000	7.643218000
H	1.465785000	6.156656000	7.467481000

54
[Cd]⁺ (H Opt)

Cd	0.459989000	3.120402000	6.600255000
Al	-0.101181000	3.942607000	4.204692000
C	-0.357024000	6.054415000	3.752150000
N	-0.050783000	2.928324000	2.658207000
C	-1.592502000	5.588364000	4.362409000
C	0.519006000	6.425659000	4.815100000
C	-0.246367000	6.420427000	2.298949000
Si	1.486664000	2.742713000	1.863226000
Si	-1.424827000	1.941446000	2.257083000
C	-1.443775000	5.733322000	5.775746000
C	-2.899431000	5.495087000	3.628960000
C	-0.159610000	6.233487000	6.045031000
C	1.903298000	6.978482000	4.677404000
H	0.771842000	6.702771000	2.025934000
H	-0.894307000	7.283070000	2.094747000
H	-0.564841000	5.605433000	1.641389000
C	2.372599000	1.135801000	2.302126000
C	2.654199000	4.120148000	2.411526000
C	1.314244000	2.834026000	-0.011070000
C	-0.973436000	0.161469000	1.826687000
C	-2.573349000	1.842443000	3.754017000
C	-2.379312000	2.621553000	0.779435000
C	-2.478107000	5.374671000	6.796285000
H	-2.795335000	5.012365000	2.654358000
H	-3.292365000	6.505224000	3.451068000
H	-3.652092000	4.948826000	4.202882000
C	0.423820000	6.488183000	7.403816000
H	2.261793000	6.935316000	3.647546000
H	2.623426000	6.447161000	5.309260000
H	1.919743000	8.029796000	4.991277000

H	2.464511000	1.014042000	3.388475000
H	3.385757000	1.159044000	1.883235000
H	1.868663000	0.249342000	1.906948000
H	2.335006000	5.109667000	2.073294000
H	3.641173000	3.925380000	1.974974000
H	2.802099000	4.161368000	3.500562000
H	0.732407000	1.998709000	-0.415488000
H	2.311213000	2.793291000	-0.465367000
H	0.834064000	3.766694000	-0.327871000
H	-0.367946000	0.084012000	0.917361000
H	-1.902513000	-0.393406000	1.647519000
H	-0.435020000	-0.337817000	2.639449000
H	-2.110849000	1.273202000	4.570507000
H	-3.491089000	1.313662000	3.470061000
H	-2.880598000	2.819268000	4.146081000
H	-2.748473000	3.640517000	0.932984000
H	-3.244328000	1.977924000	0.579709000
H	-1.754132000	2.627322000	-0.120298000
H	-2.029235000	5.036388000	7.736079000
H	-3.145835000	4.586256000	6.436085000
H	-3.104402000	6.242909000	7.039536000
H	-0.091773000	5.922026000	8.186428000
H	0.340696000	7.547950000	7.677820000
H	1.490263000	6.237669000	7.444632000

136
IM1A

Cd	1.348199000	3.050666000	6.661999000
Al	0.437920000	3.685673000	4.253206000
Al	1.423194000	2.176538000	9.128353000
C	-0.009922000	5.679400000	3.467202000
N	0.567080000	2.485793000	2.835309000
C	-1.225455000	5.107681000	4.023606000
C	0.675977000	6.331999000	4.533611000
C	-0.062021000	0.559423000	9.470673000
N	1.890034000	3.103323000	10.672461000
C	1.300000000	0.038782000	9.475930000
C	-0.520375000	0.514000000	8.127712000
C	0.222224000	5.897855000	2.000571000
Si	2.181000000	2.221855000	2.245385000
Si	-0.754983000	1.506565000	2.274945000
C	-1.225403000	5.410886000	5.418093000
C	-2.435096000	4.786666000	3.194920000
C	-0.069465000	6.156056000	5.721586000
C	1.924037000	7.139070000	4.350532000
C	-0.907343000	0.671773000	10.707077000
Si	0.849338000	4.405433000	11.174461000
Si	3.492682000	2.921954000	11.325334000
C	1.618163000	-0.307400000	8.123804000
C	1.927654000	-0.541619000	10.710627000
C	0.502578000	-0.024242000	7.315772000
C	-1.837965000	1.006668000	7.619118000
H	1.247052000	6.226746000	1.803721000
H	-0.451394000	6.674744000	1.612163000
H	0.049053000	4.985831000	1.418619000
C	2.968322000	0.671340000	2.974199000
C	3.280095000	3.686745000	2.727998000
C	2.248763000	2.100947000	0.364654000
C	-0.295548000	-0.305583000	2.006112000
C	-2.143407000	1.479758000	3.566316000
C	-1.425808000	2.089885000	0.605275000
C	-2.259571000	4.990335000	6.413275000
H	-2.204850000	4.151210000	2.336904000
H	-2.869000000	5.717153000	2.803441000
H	-3.210822000	4.283899000	3.777507000
C	0.307644000	6.574701000	7.108954000
H	2.745761000	6.549286000	3.926958000
H	2.273797000	7.561715000	5.294786000
H	1.742780000	7.975521000	3.663279000
H	-1.861422000	1.162152000	10.496503000
H	-1.134902000	-0.327513000	11.103570000
H	-0.407538000	1.232672000	11.505099000
C	1.488038000	6.123780000	10.702658000
C	-0.838659000	4.232571000	10.344405000
C	0.574995000	4.404480000	13.039540000
C	4.339013000	4.583345000	11.633063000
C	4.557541000	1.949972000	10.104340000
C	3.545698000	2.030869000	12.989386000
C	2.922854000	-0.867349000	7.648975000
H	1.794783000	0.114201000	11.575197000
H	1.453697000	-1.503322000	10.952319000
H	2.997766000	-0.730118000	10.590790000
C	0.406398000	-0.240022000	5.836012000
H	-2.342302000	1.645095000	8.350473000
H	-1.714696000	1.589924000	6.698303000
H	-2.513346000	0.173749000	7.381452000
H	3.008554000	0.746745000	4.067552000
H	3.992516000	0.532430000	2.607152000
H	2.400097000	-0.229246000	2.723824000
H	2.893187000	4.628564000	2.322675000
H	4.284314000	3.539610000	2.309876000
H	3.387720000	3.797344000	3.815054000
H	1.706636000	1.231251000	-0.020987000
H	3.290055000	2.010488000	0.032146000
H	1.822532000	2.995304000	-0.104218000
H	0.458741000	-0.455266000	1.226576000
H	-1.196543000	-0.849102000	1.693665000
H	0.066932000	-0.770738000	2.929047000
H	-2.071790000	0.567278000	4.168023000
H	-3.130033000	1.485172000	3.089214000
H	-2.114304000	2.325780000	4.262084000
H	-1.627352000	3.164542000	0.560913000
H	-2.357616000	1.564557000	0.362107000
H	-0.706142000	1.864862000	-0.189946000
H	-1.807008000	4.515186000	7.291745000
H	-2.970208000	4.277990000	5.984703000
H	-2.833462000	5.852803000	6.777018000
H	-0.533970000	7.058166000	7.619128000
H	1.142416000	7.280782000	7.114784000
H	0.600735000	5.718342000	7.733597000
H	1.918135000	6.130859000	9.694979000
H	0.660486000	6.843781000	10.713155000
H	2.252631000	6.491879000	11.393009000
H	-1.406793000	3.380671000	10.728474000
H	-1.429014000	5.137771000	10.531138000
H	-0.757835000	4.118840000	9.256494000
H	1.502629000	4.599597000	13.589541000
H	-0.141687000	5.185967000	13.320054000
H	0.177659000	3.443078000	13.384214000
H	3.898124000	5.109409000	12.487358000
H	5.397201000	4.414006000	11.868107000
H	4.286460000	5.249111000	10.766511000
H	5.614750000	2.027438000	10.388415000
H	4.303331000	0.885758000	10.100422000
H	4.456969000	2.313817000	9.073660000
H	3.299239000	0.968094000	12.909445000
H	4.552255000	2.103819000	13.420020000
H	2.847916000	2.481455000	13.703708000
H	2.837123000	-1.938786000	7.424615000
H	3.267673000	-0.365999000	6.737747000
H	3.707372000	-0.750401000	8.401289000
H	0.278494000	0.691218000	5.266781000
H	1.302324000	-0.732410000	5.447159000
H	-0.451752000	-0.879777000	5.592875000
C	6.330735000	2.933882000	5.716547000
C	7.035046000	3.906149000	4.755543000
C	7.224966000	5.309402000	5.319335000
H	6.431413000	3.974261000	3.840104000
H	7.998155000	3.463089000	4.473131000
C	5.877036000	5.885516000	5.739779000
H	7.679449000	5.951872000	4.555917000
H	7.924799000	5.300052000	6.164127000
C	5.160966000	5.024230000	6.789659000
H	5.228601000	5.964205000	4.855396000
H	5.992167000	6.897011000	6.148641000
N	5.127305000	3.611928000	6.294407000
C	7.262949000	2.456106000	6.843307000
C	5.849349000	1.717109000	4.927642000
O	4.170377000	2.860707000	6.702674000
C	3.718661000	5.494513000	6.960443000
C	5.860851000	5.112727000	8.156974000

H	5.093107000	2.009170000	4.196566000
H	5.419002000	0.956872000	5.582889000
H	6.702987000	1.285059000	4.394990000
H	7.795928000	3.273729000	7.331991000
H	8.009334000	1.774651000	6.421533000
H	6.687689000	1.910710000	7.598378000
H	6.944084000	5.002757000	8.090581000
H	5.470976000	4.349776000	8.835285000
H	5.652501000	6.094665000	8.595545000
H	3.127123000	5.302057000	6.059257000
H	3.720618000	6.572522000	7.151307000
H	3.237151000	4.996387000	7.808174000

136
IM1B

Cd	1.072626000	3.417944000	6.746546000
Al	-0.150466000	3.923560000	4.513856000
Al	2.266548000	2.837109000	8.969147000
C	-0.718301000	5.970141000	4.000259000
N	-0.358980000	2.803227000	3.054548000
C	-1.726270000	5.442377000	4.906358000
C	0.324268000	6.508961000	4.805848000
C	2.993092000	0.819714000	9.313124000
N	2.326949000	3.790904000	10.555873000
C	3.923116000	1.448050000	8.389055000
C	1.930782000	0.278640000	8.534987000
C	-0.965179000	6.210154000	2.538547000
Si	0.940764000	2.744026000	1.899563000
Si	-1.701149000	1.699502000	3.033085000
C	-1.282531000	5.719705000	6.238031000
C	-3.149845000	5.178556000	4.504768000
C	-0.033988000	6.357685000	6.170394000
C	1.576086000	7.177737000	4.331967000
C	3.297472000	0.521887000	10.751850000
Si	0.889148000	3.750573000	11.533669000
Si	3.676859000	4.822102000	10.914270000
C	3.427322000	1.210270000	7.067198000
C	5.351910000	1.776243000	8.717963000
C	2.214974000	0.513005000	7.160251000
C	0.767763000	-0.512407000	9.048717000
H	-0.051518000	6.494975000	2.009969000
H	-1.689230000	7.025220000	2.404445000
H	-1.367850000	5.321012000	2.041382000
C	1.927844000	1.135320000	1.931077000
C	2.184444000	4.109490000	2.295114000
C	0.322701000	2.989179000	0.135969000
C	-1.215252000	-0.068129000	2.591734000
C	-2.457269000	1.623299000	4.762463000
C	-3.029094000	2.196960000	1.786839000
C	-2.028485000	5.340343000	7.479109000
H	-3.223998000	4.584982000	3.589375000
H	-3.674249000	6.126587000	4.320600000
H	-3.702900000	4.650760000	5.287227000
C	0.830263000	6.796240000	7.314606000
H	1.626676000	7.229261000	3.241923000
H	2.478767000	6.661823000	4.682063000
H	1.626434000	8.204959000	4.714056000
H	2.410909000	0.195364000	11.301777000
H	4.039573000	-0.286114000	10.817254000
H	3.705870000	1.392059000	11.275526000
C	-0.077953000	5.372958000	11.527893000
C	-0.286484000	2.439207000	10.858817000
C	1.271714000	3.311475000	13.329382000
C	3.174177000	6.442088000	11.738798000
C	4.535746000	5.298604000	9.301854000
C	4.918363000	4.014381000	12.086403000
C	4.093540000	1.676765000	5.810496000
H	5.457385000	2.263579000	9.690586000
H	5.954488000	0.857674000	8.749190000
H	5.800501000	2.433754000	7.966826000
C	1.335735000	0.068902000	6.027982000
H	0.797530000	-0.619370000	10.135770000
H	-0.196109000	-0.062740000	8.780783000
H	0.782024000	-1.521856000	8.619065000
H	2.210044000	0.855727000	2.952178000
H	2.852662000	1.263482000	1.355085000
H	1.384034000	0.292022000	1.496436000
H	1.746350000	5.111119000	2.255762000
H	2.998028000	4.079115000	1.560065000
H	2.639324000	3.983126000	3.286142000
H	-0.318541000	2.158718000	-0.181391000
H	1.163369000	3.039220000	-0.566450000
H	-0.257070000	3.913661000	0.036737000
H	-0.916654000	-0.172643000	1.543056000
H	-2.078457000	-0.726540000	2.749573000
H	-0.394382000	-0.435021000	3.217577000
H	-1.755932000	1.162705000	5.468670000
H	-3.366282000	1.009866000	4.750122000
H	-2.734195000	2.604447000	5.162366000
H	-3.399802000	3.215876000	1.936948000
H	-3.888275000	1.517847000	1.848959000
H	-2.638875000	2.141555000	0.764251000
H	-1.358194000	5.218832000	8.336343000
H	-2.568075000	4.396239000	7.349086000
H	-2.768873000	6.104033000	7.752396000
H	1.885353000	6.550868000	7.142527000
H	0.538254000	6.326742000	8.258702000
H	0.776006000	7.883446000	7.461356000
H	-0.178925000	5.766930000	10.510138000
H	-1.088482000	5.202233000	11.919109000
H	0.385092000	6.152536000	12.139354000
H	0.111862000	1.425739000	10.974867000
H	-1.233379000	2.482897000	11.410875000
H	-0.526548000	2.593916000	9.798657000
H	2.018827000	3.983369000	13.766948000
H	0.367489000	3.382661000	13.946742000
H	1.652819000	2.285599000	13.403106000
H	2.728185000	6.293658000	12.728222000
H	4.065886000	7.066485000	11.873780000
H	2.461607000	7.003689000	11.125298000
H	3.882790000	5.955988000	8.715015000
H	5.464112000	5.845535000	9.505524000
H	4.794912000	4.441815000	8.670351000
H	5.383537000	3.113647000	11.673907000
H	5.723365000	4.716932000	12.334584000
H	4.428799000	3.726145000	13.024164000
H	3.394137000	1.700590000	4.968412000
H	4.510654000	2.684120000	5.924008000
H	4.922143000	1.014782000	5.524579000
H	1.327210000	0.779415000	5.193481000
H	1.660475000	-0.899982000	5.624759000
H	0.298942000	-0.052599000	6.358180000
C	2.091530000	-1.072905000	14.902723000
C	1.970558000	-0.374292000	16.263244000
C	0.603831000	-0.547848000	16.912323000
H	2.159574000	0.699342000	16.120891000
H	2.769671000	-0.756966000	16.910211000
C	-0.460825000	0.027065000	15.987113000
H	0.580534000	-0.026023000	17.876277000
H	0.404182000	-1.604711000	17.128955000
C	-0.496197000	-0.643701000	14.608036000
H	-0.263534000	1.099510000	15.847754000
H	-1.460585000	-0.059739000	16.430533000
N	0.894775000	-0.751557000	14.058707000
C	2.213766000	-2.598246000	15.056501000
C	3.330762000	-0.550634000	14.171981000
O	0.986300000	-1.043193000	12.816367000
C	-1.303214000	0.226516000	13.643147000
C	-1.129619000	-2.043471000	14.672756000
H	3.227387000	0.513142000	13.935654000
H	3.498048000	-1.096227000	13.241793000
H	4.203487000	-0.678705000	14.820724000
H	1.444549000	-3.017451000	15.709104000
H	3.190480000	-2.842576000	15.487675000
H	2.138681000	-3.074699000	14.074507000
H	-0.690476000	-2.666408000	15.455379000
H	-1.004050000	-2.549357000	13.710872000
H	-2.200999000	-1.948007000	14.878701000
H	-0.812224000	1.192912000	13.499800000
H	-2.298923000	0.397597000	14.065676000
H	-1.407378000	-0.253848000	12.668176000

136
TS1A

Cd	1.220444000	3.072407000	6.494578000
Al	0.249645000	3.632843000	4.088330000
Al	1.872483000	2.331811000	8.912974000
C	-0.047665000	5.652801000	3.288682000
N	0.368866000	2.436297000	2.669341000
C	-1.301603000	5.158669000	3.835639000
C	0.684261000	6.215865000	4.367591000
C	0.420962000	0.590302000	9.355953000
N	2.134709000	3.287226000	10.485271000
C	1.820987000	0.207711000	9.176695000
C	-0.199613000	0.514519000	8.097838000
C	0.206925000	5.830556000	1.821043000
Si	1.960903000	2.142347000	2.035057000
Si	-0.982448000	1.453935000	2.185026000
C	-1.308084000	5.486755000	5.232538000
C	-2.533126000	4.937854000	3.004525000
C	-0.102650000	6.128872000	5.545249000
C	2.040693000	6.842836000	4.291538000
C	-0.243986000	0.703868000	10.695712000
Si	0.941331000	4.518676000	10.833829000
Si	3.443537000	3.039294000	11.615548000
C	1.964295000	-0.169466000	7.789935000
C	2.625120000	-0.444705000	10.271156000
C	0.744366000	0.024308000	7.144789000
C	-1.613931000	0.875412000	7.765998000
H	1.229678000	6.165072000	1.628445000
H	-0.468751000	6.592908000	1.409845000
H	0.043055000	4.904147000	1.258464000
C	2.775735000	0.624156000	2.803049000
C	3.082247000	3.624307000	2.395722000
C	1.966023000	1.926954000	0.161745000
C	-0.553009000	-0.372654000	1.976139000
C	-2.318625000	1.506615000	3.522646000
C	-1.698029000	1.996373000	0.522766000
C	-2.436858000	5.170481000	6.163531000
H	-2.348456000	4.299471000	2.137008000
H	-2.905439000	5.900969000	2.628135000
H	-3.340389000	4.483340000	3.585550000
C	0.358401000	6.575128000	6.896779000
H	2.593082000	6.515214000	3.406704000
H	2.645928000	6.598610000	5.171977000
H	1.964210000	7.937678000	4.248719000
H	-1.276893000	1.051903000	10.606753000
H	-0.273977000	-0.278876000	11.186538000
H	0.285069000	1.388410000	11.368692000
C	1.521592000	6.276135000	10.462844000
C	-0.627303000	4.250609000	9.815165000
C	0.386134000	4.508012000	12.638773000
C	4.224569000	4.676187000	12.150423000
C	4.840197000	1.989819000	10.883764000
C	2.899548000	2.186784000	13.214779000
C	3.227964000	-0.721896000	7.214019000
H	2.567120000	0.101792000	11.215370000
H	2.243311000	-1.459045000	10.456143000
H	3.680205000	-0.532326000	10.000719000
C	0.423093000	-0.259682000	5.708417000
H	-2.091772000	1.434486000	8.574701000
H	-1.672524000	1.491354000	6.858951000
H	-2.219380000	-0.021791000	7.578549000
H	2.775568000	0.697233000	3.896309000
H	3.817129000	0.529535000	2.472197000
H	2.253801000	-0.299257000	2.533285000
H	2.820320000	4.479461000	1.763748000
H	4.122988000	3.355312000	2.176136000
H	3.045570000	3.956974000	3.440891000
H	1.430367000	1.029411000	-0.164630000
H	2.998462000	1.839600000	-0.198569000
H	1.509732000	2.790181000	-0.336089000
H	0.166803000	-0.559209000	1.172320000
H	-1.468053000	-0.923936000	1.724889000
H	-0.151580000	-0.800814000	2.900664000
H	-2.082381000	0.795980000	4.322615000
H	-3.292421000	1.222475000	3.106478000
H	-2.438163000	2.491999000	3.984195000
H	-1.907091000	3.069135000	0.468592000
H	-2.631814000	1.461946000	0.309484000
H	-0.994491000	1.763797000	-0.284587000
H	-2.125923000	5.201647000	7.211041000
H	-2.843368000	4.170616000	5.974928000
H	-3.263515000	5.883291000	6.041403000
H	-0.463029000	6.610164000	7.616821000
H	0.802298000	7.576871000	6.855943000
H	1.123791000	5.906140000	7.313638000
H	1.743315000	6.415239000	9.400785000
H	0.728403000	6.986046000	10.729529000
H	2.417399000	6.550794000	11.027682000
H	-1.103486000	3.290012000	10.035298000
H	-1.344573000	5.043655000	10.062967000
H	-0.446013000	4.291152000	8.736124000
H	1.169233000	4.835134000	13.330448000
H	-0.456530000	5.201332000	12.751876000
H	0.047057000	3.516552000	12.958263000
H	3.534727000	5.314543000	12.712206000
H	5.080068000	4.467558000	12.804958000
H	4.592385000	5.251889000	11.293660000
H	5.801866000	2.453029000	11.135966000
H	4.848987000	0.985189000	11.315209000
H	4.801478000	1.898873000	9.793224000
H	2.264893000	1.312624000	13.033052000
H	3.785204000	1.842470000	13.763650000
H	2.345302000	2.860437000	13.874815000
H	3.368004000	-1.772053000	7.506687000
H	3.232727000	-0.681336000	6.121009000
H	4.099565000	-0.164393000	7.573815000
H	0.288538000	0.647108000	5.101817000
H	1.213126000	-0.847382000	5.233122000
H	-0.507108000	-0.835904000	5.628006000
C	6.014458000	2.944542000	5.897442000
C	6.522328000	3.999116000	4.903660000
C	7.055853000	5.259421000	5.570309000
H	5.694861000	4.277698000	4.235812000
H	7.288445000	3.523751000	4.278747000
C	5.946282000	5.878389000	6.409278000
H	7.387784000	5.972262000	4.806388000
H	7.935339000	5.037440000	6.187762000
C	5.421056000	4.946494000	7.508622000
H	5.110689000	6.144858000	5.746275000
H	6.279572000	6.806393000	6.889833000
N	5.152197000	3.589006000	6.937849000
C	7.176405000	2.206941000	6.581796000
C	5.146439000	1.933052000	5.148626000
O	4.484249000	2.779650000	7.681484000
C	4.094676000	5.487429000	8.039179000
C	6.419597000	4.828459000	8.670549000
H	4.228456000	2.408086000	4.790938000
H	4.873978000	1.090937000	5.785357000
H	5.699421000	1.557582000	4.281431000
H	7.922244000	2.890367000	6.995281000
H	7.676994000	1.563137000	5.850732000
H	6.791000000	1.579105000	7.391027000
H	7.427769000	4.574722000	8.333701000
H	6.081890000	4.061165000	9.371910000
H	6.472441000	5.785127000	9.201361000
H	3.338676000	5.477689000	7.246115000
H	4.231520000	6.520534000	8.376022000
H	3.719495000	4.890549000	8.873543000

136
TS1B

Cd	0.910490000	2.883837000	7.520098000
Al	-0.185155000	3.571558000	5.218544000
Al	2.161453000	2.296985000	9.748161000
C	-0.331929000	5.648780000	4.540359000
N	-0.471860000	2.393501000	3.798279000
C	-1.560513000	5.284586000	5.228174000
C	0.583584000	6.096296000	5.533832000
C	3.531020000	0.701198000	9.207222000
N	2.329818000	3.609628000	11.080239000

C	4.111975000	1.539011000	8.155483000
C	2.487368000	-0.079859000	8.545930000
C	-0.258953000	5.907854000	3.064190000
Si	0.682091000	2.226906000	2.504647000
Si	-1.865416000	1.357595000	3.938795000
C	-1.371256000	5.584217000	6.612884000
C	-2.888333000	5.141925000	4.542193000
C	-0.063697000	6.067602000	6.792121000
C	1.990790000	6.554853000	5.311512000
C	4.416611000	0.023881000	10.231741000
Si	0.823903000	4.303015000	11.643532000
Si	3.823219000	4.461843000	11.384049000
C	3.396491000	1.325081000	6.978571000
C	5.403868000	2.287697000	8.260625000
C	2.400531000	0.322074000	7.221466000
C	1.710399000	-1.204416000	9.155417000
H	0.762253000	6.121419000	2.742152000
H	-0.875606000	6.780150000	2.807934000
H	-0.627991000	5.062369000	2.474105000
C	1.576875000	0.563835000	2.449822000
C	2.055797000	3.527178000	2.669680000
C	-0.126373000	2.424922000	0.810283000
C	-1.505676000	-0.459760000	3.578846000
C	-2.526394000	1.436611000	5.709462000
C	-3.282440000	1.828638000	2.783027000
C	-2.425999000	5.395518000	7.658131000
H	-2.779878000	4.679968000	3.557265000
H	-3.352329000	6.127353000	4.392418000
H	-3.591608000	4.533509000	5.119998000
C	0.600138000	6.441205000	8.080864000
H	2.340166000	6.322096000	4.302139000
H	2.684394000	6.089919000	6.022549000
H	2.074046000	7.641464000	5.446455000
H	3.842722000	-0.376897000	11.069072000
H	4.947166000	-0.814273000	9.759170000
H	5.176811000	0.690669000	10.641853000
C	0.771777000	6.198340000	11.646321000
C	-0.651205000	3.793704000	10.579744000
C	0.386603000	3.804155000	13.413816000
C	3.757306000	5.716668000	12.798157000
C	4.330961000	5.411627000	9.832035000
C	5.222584000	3.320200000	11.929702000
C	3.665651000	1.952576000	5.645283000
H	5.551941000	2.755856000	9.236479000
H	6.253208000	1.607937000	8.097942000
H	5.475847000	3.076577000	7.505517000
C	1.454516000	-0.237431000	6.201565000
H	2.013695000	-1.381283000	10.188671000
H	0.630943000	-1.007175000	9.162510000
H	1.869106000	-2.132082000	8.590642000
H	2.121170000	0.355573000	3.376243000
H	2.313924000	0.599834000	1.637127000
H	0.913523000	-0.282075000	2.252894000
H	1.950144000	4.315255000	1.917849000
H	3.032561000	3.053733000	2.518998000
H	2.087190000	4.012867000	3.652584000
H	-0.803245000	1.591498000	0.588900000
H	0.639501000	2.438841000	0.025100000
H	-0.704015000	3.352749000	0.733629000
H	-1.338075000	-0.643459000	2.511990000
H	-2.376109000	-1.057627000	3.876376000
H	-0.635993000	-0.832626000	4.129188000
H	-1.814380000	1.015251000	6.429482000
H	-3.450974000	0.849040000	5.773060000
H	-2.769697000	2.454292000	6.038810000
H	-3.781704000	2.752481000	3.089258000
H	-4.036691000	1.031904000	2.779880000
H	-2.937595000	1.961236000	1.752011000
H	-2.158273000	5.893654000	8.593594000
H	-2.593927000	4.336626000	7.894682000
H	-3.385381000	5.809516000	7.325456000
H	1.250410000	5.644529000	8.473980000
H	-0.133341000	6.669002000	8.858511000
H	1.229887000	7.329617000	7.954433000
H	1.385961000	6.653314000	10.863475000
H	-0.264764000	6.512926000	11.471715000
H	1.079909000	6.616451000	12.608629000
H	-0.729629000	2.720175000	10.394655000
H	-1.568170000	4.121628000	11.089044000
H	-0.620213000	4.290669000	9.604808000
H	1.158912000	4.096592000	14.134147000
H	-0.543353000	4.307148000	13.708792000
H	0.228459000	2.724971000	13.504911000
H	3.234122000	5.333293000	13.681358000
H	4.791900000	5.920523000	13.101427000
H	3.304049000	6.670200000	12.519112000
H	3.650413000	6.259124000	9.684920000
H	5.350833000	5.808445000	9.899205000
H	4.264994000	4.788482000	8.933201000
H	5.324243000	2.407615000	11.342942000
H	6.175311000	3.860868000	11.872862000
H	5.073325000	3.023461000	12.974086000
H	2.755288000	2.009259000	5.040054000
H	4.059896000	2.969777000	5.738229000
H	4.397388000	1.368460000	5.069823000
H	1.033296000	0.536943000	5.548838000
H	1.958439000	-0.963688000	5.548541000
H	0.617391000	-0.755362000	6.680237000
C	1.856322000	0.093823000	13.757478000
C	1.227572000	0.224682000	15.152442000
C	-0.058187000	-0.575020000	15.319749000
H	1.014712000	1.286077000	15.338792000
H	1.982306000	-0.083641000	15.886489000
C	-1.072175000	-0.123033000	14.277677000
H	-0.465129000	-0.414802000	16.325016000
H	0.134441000	-1.651760000	15.236986000
C	-0.578219000	-0.265579000	12.829763000
H	-1.317132000	0.933146000	14.456677000
H	-2.008612000	-0.687293000	14.366954000
N	0.799306000	0.323012000	12.714764000
C	2.496765000	-1.293711000	13.568285000
C	2.932460000	1.165061000	13.588657000
O	1.196910000	0.626879000	11.530468000
C	-1.513256000	0.515543000	11.908434000
C	-0.556141000	-1.735983000	12.379112000
H	2.501032000	2.167553000	13.606739000
H	3.469465000	1.053120000	12.647047000
H	3.647582000	1.070406000	14.412656000
H	1.867478000	-2.106429000	13.935944000
H	3.440862000	-1.325281000	14.121866000
H	2.716245000	-1.475805000	12.513142000
H	-0.047128000	-2.389699000	13.089552000
H	-0.063326000	-1.822111000	11.406900000
H	-1.586663000	-2.091151000	12.274977000
H	-1.514202000	1.578182000	12.163683000
H	-2.529509000	0.129118000	12.035347000
H	-1.224064000	0.405754000	10.860796000

136
IM2A

Cd	1.282478000	3.008682000	6.435830000
Al	0.228363000	3.529048000	4.018661000
Al	2.643948000	2.559510000	8.865095000
C	0.038704000	5.548246000	3.274000000
N	0.324454000	2.321421000	2.630458000
C	-1.222746000	5.109748000	3.869532000
C	0.834285000	6.070463000	4.328199000
C	0.665760000	0.762631000	9.077821000
N	2.341036000	3.419650000	10.484334000
C	2.119531000	0.521227000	8.967317000
C	0.097734000	0.580216000	7.832608000
C	0.233416000	5.739778000	1.798648000
Si	1.858971000	2.069790000	1.830647000
Si	-1.060715000	1.347052000	2.202691000
C	-1.164521000	5.450880000	5.265129000
C	-2.496190000	4.960401000	3.085207000
C	0.081157000	6.030458000	5.532420000
C	2.232526000	6.592587000	4.236816000
C	-0.030142000	0.924873000	10.388695000
Si	1.045102000	4.562732000	10.748267000
Si	3.465758000	3.133003000	11.798518000

C	2.316744000	0.011925000	7.604435000
C	2.815362000	-0.212501000	10.105472000
C	1.119726000	0.095445000	6.920340000
C	-1.336531000	0.774120000	7.446229000
H	1.252703000	6.057291000	1.568446000
H	-0.445556000	6.521432000	1.432360000
H	0.025881000	4.827750000	1.227451000
C	2.757563000	0.544184000	2.468801000
C	2.987721000	3.561334000	2.101921000
C	1.660520000	1.891981000	-0.035754000
C	-0.645660000	-0.474059000	1.943247000
C	-2.315484000	1.404105000	3.610224000
C	-1.873115000	1.925223000	0.599501000
C	-2.280024000	5.200830000	6.229287000
H	-2.366621000	4.347827000	2.189834000
H	-2.852049000	5.947450000	2.758789000
H	-3.292498000	4.510614000	3.684835000
C	0.614672000	6.494135000	6.849223000
H	2.667007000	6.440840000	3.245745000
H	2.887258000	6.104139000	4.970806000
H	2.258464000	7.668425000	4.452463000
H	-1.088808000	1.170165000	10.270496000
H	0.022302000	-0.018449000	10.951671000
H	0.438638000	1.692494000	11.012667000
C	1.561527000	6.364633000	10.513820000
C	-0.424862000	4.285012000	9.595837000
C	0.318907000	4.467524000	12.490230000
C	4.162109000	4.738716000	12.509313000
C	4.954166000	2.092296000	11.247303000
C	2.702002000	2.207280000	13.263337000
C	3.581773000	-0.654330000	7.170198000
H	2.674045000	0.288976000	11.066184000
H	2.404154000	-1.228038000	10.200216000
H	3.891571000	-0.305535000	9.937491000
C	0.827896000	-0.367330000	5.525272000
H	-1.931142000	1.160087000	8.278237000
H	-1.443407000	1.483848000	6.613760000
H	-1.796419000	-0.166814000	7.115477000
H	2.933480000	0.618733000	3.546453000
H	3.732204000	0.440750000	1.976285000
H	2.190173000	-0.373694000	2.287399000
H	2.771376000	4.351100000	1.375598000
H	4.033733000	3.261498000	1.967328000
H	2.912314000	4.002447000	3.102480000
H	1.141886000	0.972959000	-0.327417000
H	2.652817000	1.868003000	-0.503007000
H	1.112157000	2.739756000	-0.461663000
H	0.023123000	-0.642614000	1.092869000
H	-1.573524000	-1.022435000	1.737497000
H	-0.185233000	-0.920605000	2.830424000
H	-1.972226000	0.792033000	4.451438000
H	-3.276250000	0.994200000	3.276889000
H	-2.510542000	2.414289000	3.986676000
H	-2.052577000	3.004284000	0.570561000
H	-2.838333000	1.423261000	0.460113000
H	-1.247315000	1.677541000	-0.264378000
H	-1.941361000	5.238889000	7.266954000
H	-2.733725000	4.216248000	6.068948000
H	-3.077333000	5.946550000	6.109737000
H	-0.154604000	6.505467000	7.623828000
H	1.022634000	7.509385000	6.772404000
H	1.433093000	5.853384000	7.206840000
H	1.750846000	6.607656000	9.464695000
H	0.754270000	7.019440000	10.865537000
H	2.465481000	6.616590000	11.076945000
H	-0.981609000	3.378005000	9.844160000
H	-1.108288000	5.137541000	9.701267000
H	-0.138857000	4.216916000	8.541325000
H	1.007608000	4.822034000	13.263607000
H	-0.565724000	5.115730000	12.523592000
H	-0.001536000	3.455619000	12.758910000
H	3.387489000	5.391548000	12.924772000
H	4.855686000	4.496726000	13.324370000
H	4.718621000	5.309234000	11.757253000
H	5.873862000	2.603312000	11.555643000
H	4.945087000	1.114634000	11.738790000
H	5.032031000	1.929485000	10.167437000
H	2.070609000	1.367283000	12.954389000
H	3.512720000	1.799859000	13.880373000
H	2.097732000	2.854826000	13.904265000
H	3.663541000	-1.647593000	7.635292000
H	3.631627000	-0.796254000	6.086924000
H	4.467797000	-0.087336000	7.476031000
H	0.548591000	0.450228000	4.846987000
H	1.690265000	-0.871828000	5.082043000
H	-0.006516000	-1.081065000	5.524700000
C	5.388761000	3.075383000	6.061928000
C	5.925891000	4.099316000	5.046328000
C	6.746193000	5.211910000	5.678834000
H	5.066853000	4.548659000	4.527342000
H	6.506208000	3.551727000	4.292466000
C	5.895126000	5.894931000	6.737973000
H	7.052031000	5.936246000	4.913869000
H	7.671503000	4.816880000	6.115941000
C	5.365767000	4.929801000	7.812819000
H	5.034767000	6.375001000	6.249910000
H	6.456295000	6.687276000	7.249505000
N	4.640557000	3.817731000	7.120892000
C	6.520204000	2.166990000	6.581661000
C	4.370271000	2.208052000	5.321686000
O	4.222760000	2.854544000	8.106017000
C	4.326849000	5.688700000	8.636118000
C	6.504836000	4.490140000	8.750212000
H	3.587390000	2.839823000	4.887330000
H	3.902534000	1.485074000	5.989933000
H	4.856323000	1.666315000	4.503118000
H	7.423626000	2.722464000	6.838429000
H	6.788536000	1.437979000	5.809667000
H	6.188165000	1.622616000	7.469151000
H	7.425982000	4.244081000	8.219045000
H	6.199869000	3.621085000	9.334647000
H	6.736173000	5.304191000	9.446429000
H	3.476101000	5.958512000	8.002111000
H	4.762742000	6.609912000	9.039246000
H	3.956804000	5.091884000	9.471329000

136
IM2B

Cd	1.023858000	2.760004000	7.675555000
Al	-0.043214000	3.564405000	5.327893000
Al	2.057466000	1.941774000	10.302547000
C	-0.108768000	5.597074000	4.606383000
N	-0.381791000	2.311583000	4.003867000
C	-1.373095000	5.273935000	5.258539000
C	0.778618000	6.050434000	5.628799000
C	3.457617000	0.825739000	9.145319000
N	2.180103000	3.614348000	11.119182000
C	4.023150000	1.690094000	8.081608000
C	2.490208000	-0.045259000	8.423240000
C	-0.001780000	5.884133000	3.137108000
Si	0.622333000	2.150077000	2.582148000
Si	-1.785381000	1.295792000	4.261011000
C	-1.226056000	5.593641000	6.642492000
C	-2.676231000	5.141462000	4.526859000
C	0.085869000	6.058042000	6.859008000
C	2.202511000	6.473912000	5.448370000
C	4.475491000	0.067314000	10.003100000
Si	0.692517000	4.373482000	11.668387000
Si	3.677249000	4.494515000	11.317335000
C	3.378341000	1.425636000	6.893052000
C	5.249869000	2.528368000	8.231834000
C	2.426992000	0.349426000	7.108932000
C	1.821548000	-1.265893000	8.971920000
H	1.029571000	6.075574000	2.834489000
H	-0.589120000	6.780343000	2.895467000
H	-0.386107000	5.066199000	2.519844000
C	1.470262000	0.475316000	2.395870000
C	2.047939000	3.405949000	2.609429000
C	-0.380725000	2.394478000	1.001441000
C	-1.499806000	-0.524210000	3.860339000
C	-2.290245000	1.403129000	6.078692000

C	-3.288514000	1.792473000	3.232098000
C	-2.326647000	5.469544000	7.649190000
H	-2.550808000	4.620897000	3.573804000
H	-3.095970000	6.133867000	4.309017000
H	-3.419596000	4.591571000	5.112110000
C	0.702404000	6.445613000	8.163122000
H	2.595963000	6.176551000	4.472526000
H	2.851555000	6.042346000	6.219312000
H	2.297300000	7.565222000	5.522565000
H	4.010274000	-0.441302000	10.850504000
H	4.975388000	-0.693234000	9.387349000
H	5.256544000	0.721087000	10.397870000
C	0.718361000	6.271188000	11.650377000
C	-0.733788000	3.905821000	10.526824000
C	0.226374000	3.918553000	13.431879000
C	3.689235000	5.714193000	12.763126000
C	4.071278000	5.487871000	9.757468000
C	5.114453000	3.353964000	11.760130000
C	3.662470000	2.013782000	5.545676000
H	5.287152000	3.068844000	9.178636000
H	6.147646000	1.893630000	8.192291000
H	5.340433000	3.262080000	7.425402000
C	1.595560000	-0.287660000	6.040260000
H	2.131972000	-1.461155000	9.997622000
H	0.729031000	-1.174521000	8.978839000
H	2.072697000	-2.141364000	8.359529000
H	2.177004000	0.281651000	3.208923000
H	2.046134000	0.495255000	1.461336000
H	0.780120000	-0.369834000	2.338842000
H	1.979511000	4.095132000	1.761675000
H	3.004021000	2.877083000	2.528861000
H	2.096367000	4.014656000	3.519606000
H	-1.061898000	1.553129000	0.828518000
H	0.291019000	2.449149000	0.136171000
H	-0.982545000	3.309695000	1.019090000
H	-1.462811000	-0.703955000	2.780194000
H	-2.348642000	-1.099238000	4.250976000
H	-0.587013000	-0.929121000	4.305895000
H	-1.528920000	0.982879000	6.747259000
H	-3.213747000	0.829839000	6.227409000
H	-2.497440000	2.428872000	6.411300000
H	-3.780896000	2.691200000	3.612649000
H	-4.024355000	0.979084000	3.255627000
H	-3.029320000	1.970754000	2.183472000
H	-2.108952000	6.049390000	8.549617000
H	-2.491603000	4.433398000	7.970238000
H	-3.272402000	5.838540000	7.236630000
H	1.294812000	5.633770000	8.609321000
H	-0.056214000	6.728533000	8.896259000
H	1.377292000	7.300129000	8.039642000
H	1.361862000	6.717852000	10.887836000
H	-0.305498000	6.614094000	11.453300000
H	1.010481000	6.675162000	12.623686000
H	-0.810791000	2.836920000	10.317584000
H	-1.685813000	4.227505000	10.969065000
H	-0.624027000	4.420238000	9.567577000
H	0.996340000	4.231451000	14.146856000
H	-0.707390000	4.423745000	13.709952000
H	0.090173000	2.836927000	13.523372000
H	3.203362000	5.305672000	13.656430000
H	4.738866000	5.898595000	13.024436000
H	3.233524000	6.679524000	12.534877000
H	3.437445000	6.380860000	9.713093000
H	5.115465000	5.822223000	9.736512000
H	3.880517000	4.906063000	8.848865000
H	5.138904000	2.406262000	11.222495000
H	6.066338000	3.866724000	11.575046000
H	5.067967000	3.118601000	12.829392000
H	2.749310000	2.079598000	4.945015000
H	4.089253000	3.019518000	5.608628000
H	4.368040000	1.388416000	4.981225000
H	1.098614000	0.454517000	5.404372000
H	2.209040000	-0.914005000	5.377741000
H	0.820613000	-0.926792000	6.473480000
C	1.802214000	0.202130000	13.522092000
C	1.167216000	0.262287000	14.922888000
C	-0.129001000	-0.530697000	15.028588000
H	0.965067000	1.314815000	15.164691000
H	1.909755000	-0.099001000	15.646143000
C	-1.099252000	-0.058146000	13.954556000
H	-0.572912000	-0.392809000	16.022333000
H	0.064149000	-1.606005000	14.928249000
C	-0.514836000	-0.123365000	12.530942000
H	-1.383332000	0.982557000	14.163575000
H	-2.021914000	-0.652600000	13.968961000
N	0.761012000	0.651987000	12.549482000
C	2.416356000	-1.190576000	13.262060000
C	2.941680000	1.225327000	13.483208000
O	1.326934000	0.620479000	11.222950000
C	-1.487179000	0.609462000	11.602959000
C	-0.414983000	-1.583105000	12.047547000
H	2.555773000	2.246978000	13.522831000
H	3.540827000	1.114234000	12.575309000
H	3.609719000	1.059895000	14.335949000
H	1.824918000	-2.006575000	13.680238000
H	3.410493000	-1.241343000	13.719781000
H	2.530414000	-1.362348000	12.189549000
H	0.013896000	-2.255660000	12.790987000
H	0.187207000	-1.639264000	11.139561000
H	-1.419026000	-1.953486000	11.812528000
H	-1.593088000	1.652569000	11.911964000
H	-2.472404000	0.133410000	11.653215000
H	-1.142543000	0.575612000	10.564889000

82
compound 2

Si	2.925682000	4.423086000	7.239946000
N	4.398270000	4.787168000	6.379887000
C	1.497100000	5.423304000	6.523140000
C	2.412058000	2.604525000	7.175758000
C	3.063589000	4.909688000	9.059705000
Si	5.882093000	4.126362000	7.000099000
Al	4.226724000	5.443178000	4.658007000
H	1.644575000	6.500352000	6.645564000
H	0.574102000	5.157486000	7.052321000
H	1.326873000	5.209149000	5.462578000
H	2.727795000	2.139033000	6.236935000
H	1.321099000	2.515925000	7.243684000
H	2.841640000	2.025681000	7.998864000
H	3.871474000	4.380787000	9.577170000
H	2.129207000	4.669681000	9.581759000
H	3.244196000	5.984502000	9.175155000
C	7.138082000	3.894318000	5.609110000
C	5.685828000	2.403599000	7.750353000
C	6.656507000	5.190780000	8.366997000
O	3.492590000	4.041329000	3.905582000
C	5.542949000	7.313547000	4.838985000
C	4.207604000	7.693686000	5.112478000
C	3.420017000	7.440276000	3.941387000
C	4.289671000	6.923387000	2.932483000
C	5.598573000	6.802453000	3.501352000
H	6.936040000	2.967164000	5.061251000
H	8.150692000	3.817790000	6.023477000
H	7.145019000	4.713714000	4.883662000
H	5.108950000	2.393017000	8.680689000
H	6.687702000	2.023042000	7.987772000
H	5.218250000	1.701264000	7.052539000
H	7.467085000	5.824141000	7.993927000
H	7.077747000	4.551541000	9.151993000
H	5.913727000	5.843773000	8.837266000
N	3.260332000	3.614474000	2.552000000
C	6.697991000	7.536257000	5.758093000
C	3.740285000	8.293931000	6.400576000
C	1.998070000	7.876703000	3.749620000
C	3.957038000	6.664482000	1.494865000
C	6.852419000	6.526524000	2.727897000
C	1.803672000	3.306892000	2.422677000
C	4.236005000	2.513197000	2.273540000
H	6.382073000	7.505208000	6.803447000
H	7.140352000	8.524780000	5.574127000
H	7.492535000	6.796978000	5.624666000

H	2.676950000	8.543423000	6.363447000
H	4.286606000	9.220580000	6.613771000
H	3.902634000	7.613678000	7.246135000
H	1.567028000	7.458926000	2.838474000
H	1.953370000	8.970370000	3.668119000
H	1.348952000	7.585780000	4.580619000
H	4.750095000	7.059024000	0.849409000
H	3.028713000	7.169900000	1.215781000
H	3.833268000	5.595539000	1.289657000
H	7.651891000	6.124473000	3.356505000
H	7.227912000	7.461107000	2.289886000
H	6.686236000	5.822679000	1.910188000
C	1.568830000	2.676819000	1.039986000
C	1.062305000	4.648124000	2.445821000
C	1.214883000	2.421505000	3.534153000
C	3.899936000	1.891159000	0.907362000
C	5.609640000	3.180429000	2.137420000
C	4.328658000	1.427493000	3.362501000
H	0.503348000	2.427935000	0.958852000
H	1.782906000	3.434035000	0.272012000
C	2.446303000	1.461679000	0.781372000
H	1.199946000	5.159273000	3.400487000
H	-0.011488000	4.487286000	2.299707000
H	1.427105000	5.299172000	1.646036000
H	1.579993000	1.393924000	3.505762000
H	0.124736000	2.388988000	3.430055000
H	1.453234000	2.840351000	4.513217000
H	4.113404000	2.633343000	0.124838000
H	4.584584000	1.048871000	0.746098000
H	5.572919000	3.950297000	1.360834000
H	6.365819000	2.439141000	1.856482000
H	5.927008000	3.640652000	3.075886000
H	4.355415000	1.892737000	4.351480000
H	5.251041000	0.852978000	3.223640000
H	3.496518000	0.721069000	3.333215000
H	2.255978000	1.062083000	-0.222326000
H	2.213111000	0.654552000	1.486353000

86
compound 3

Si	2.936130000	4.453592000	7.261562000
N	4.400848000	4.794790000	6.378467000
C	1.502076000	5.439447000	6.535694000
C	2.416130000	2.636091000	7.237913000
C	3.093538000	4.979429000	9.068329000
Si	5.889451000	4.145441000	7.000460000
Al	4.216277000	5.409826000	4.644241000
H	1.658808000	6.519294000	6.611697000
H	0.588151000	5.201837000	7.093303000
H	1.308783000	5.184481000	5.488212000
H	2.716287000	2.153739000	6.302412000
H	1.325919000	2.552936000	7.323377000
H	2.855125000	2.068990000	8.064144000
H	3.911427000	4.466947000	9.586563000
H	2.167312000	4.745116000	9.607146000
H	3.268793000	6.057556000	9.158901000
C	7.133369000	3.880637000	5.604007000
C	5.695758000	2.439131000	7.787663000
C	6.676472000	5.237243000	8.338181000
O	3.481518000	3.985039000	3.930614000
C	5.539988000	7.283021000	4.776227000
C	4.206691000	7.670285000	5.048357000
C	3.411624000	7.388692000	3.888552000
C	4.275624000	6.846921000	2.887176000
C	5.587842000	6.739562000	3.451450000
H	6.930213000	2.937489000	5.084471000
H	8.149997000	3.819202000	6.010965000
H	7.131074000	4.678873000	4.855238000
H	5.126012000	2.449148000	8.722414000
H	6.698428000	2.060920000	8.025467000
H	5.221193000	1.723484000	7.108293000
H	7.494742000	5.850237000	7.948254000
H	7.090109000	4.613751000	9.139677000
H	5.941808000	5.911158000	8.791292000
N	3.234657000	3.537234000	2.588436000
C	6.698927000	7.521697000	5.686307000
C	3.746749000	8.300948000	6.324515000
C	1.989745000	7.824420000	3.694013000
C	3.937284000	6.559677000	1.456129000
C	6.836648000	6.445111000	2.676908000
C	1.775504000	3.234735000	2.474807000
C	4.201749000	2.425766000	2.318746000
H	6.386835000	7.511158000	6.733169000
H	7.142053000	8.505756000	5.481683000
H	7.491584000	6.778607000	5.563910000
H	2.684114000	8.553199000	6.286349000
H	4.296974000	9.230406000	6.513936000
H	3.910819000	7.640050000	7.184950000
H	1.556186000	7.394455000	2.789824000
H	1.946628000	8.916813000	3.596951000
H	1.341702000	7.546491000	4.530331000
H	4.730816000	6.936472000	0.800753000
H	3.011092000	7.064563000	1.168922000
H	3.807354000	5.487532000	1.272833000
H	7.647334000	6.082045000	3.314694000
H	7.195696000	7.364304000	2.194675000
H	6.672393000	5.704484000	1.891933000
C	1.531023000	2.593100000	1.098255000
C	1.040394000	4.579681000	2.483952000
C	1.187317000	2.370986000	3.604203000
C	3.851963000	1.793099000	0.960478000
C	5.578199000	3.083271000	2.162332000
C	4.302524000	1.356405000	3.423768000
H	0.468708000	2.333366000	1.013858000
H	1.754963000	3.344528000	0.329252000
C	2.397166000	1.365767000	0.868070000
H	1.188982000	5.102502000	3.430587000
H	-0.035059000	4.421767000	2.348850000
H	1.402340000	5.217689000	1.672599000
H	1.561260000	1.346571000	3.609569000
H	0.098566000	2.325282000	3.493013000
H	1.414658000	2.818940000	4.572965000
H	4.057660000	2.535145000	0.177216000
H	4.517618000	0.937146000	0.792452000
H	5.538446000	3.847684000	1.380685000
H	6.326372000	2.334822000	1.879715000
H	5.906461000	3.547778000	3.094941000
H	4.348843000	1.840724000	4.402865000
H	5.218682000	0.773575000	3.280250000
H	3.466915000	0.654208000	3.422701000
O	2.079100000	0.658088000	-0.338575000
H	2.181917000	0.618138000	1.640525000
C	2.302373000	1.348968000	-1.557621000
H	1.957257000	0.680263000	-2.350613000
H	3.366277000	1.568182000	-1.729589000
H	1.732029000	2.286935000	-1.624243000

65
compound 5

S	-3.081144000	10.069437000	7.961318000
Al	-3.597787000	10.524321000	5.836505000
C	-2.138847000	11.496012000	8.514765000
N	-2.868388000	12.009777000	5.084263000
C	-5.416955000	9.836697000	4.949869000
C	-5.964805000	9.391287000	6.181385000
C	-4.291506000	8.938504000	4.632572000
C	-5.232571000	8.264775000	6.628918000
C	-4.227029000	7.975300000	5.694767000
C	-2.710906000	12.368064000	9.445494000
C	-0.811159000	11.690026000	8.127167000
Si	-1.416915000	11.858710000	4.133808000
Si	-3.622472000	13.539714000	5.464367000
C	-6.060559000	10.729408000	3.928368000
C	-7.066583000	10.028915000	6.961774000
C	-3.816339000	8.722432000	3.222069000
C	-5.456713000	7.580031000	7.939870000
C	-3.186340000	6.910850000	5.838725000
H	-3.737477000	12.210075000	9.762844000
C	-1.966781000	13.418643000	9.973574000
H	-0.353827000	11.003608000	7.422495000

C	-0.072406000	12.743463000	8.656007000
C	-0.697056000	10.131115000	4.390066000
C	-1.741424000	12.095226000	2.290874000
C	-0.096616000	13.117572000	4.601427000
C	-5.108868000	13.204664000	6.577980000
C	-4.228358000	14.421361000	3.911591000
C	-2.506518000	14.739419000	6.386302000
H	-6.766556000	11.428832000	4.383363000
H	-6.616491000	10.128814000	3.195645000
H	-5.319667000	11.318712000	3.377737000
H	-7.819511000	9.281986000	7.238905000
H	-7.569923000	10.820024000	6.400488000
H	-6.689061000	10.464739000	7.896821000
H	-2.938856000	8.071900000	3.180106000
H	-3.560678000	9.658734000	2.718454000
H	-4.607808000	8.239550000	2.632896000
H	-5.470991000	8.297964000	8.768139000
H	-4.670489000	6.850160000	8.150061000
H	-6.416160000	7.046972000	7.949119000
H	-3.637535000	5.911627000	5.817689000
H	-2.647461000	7.009644000	6.788854000
H	-2.447748000	6.959770000	5.034447000
C	-0.645863000	13.611869000	9.580678000
H	-2.424483000	14.088244000	10.696339000
H	0.960294000	12.880832000	8.347050000
H	-0.519918000	9.896198000	5.445986000
H	0.267260000	10.057020000	3.873090000
H	-1.339486000	9.346185000	3.979409000
H	-2.498016000	11.405269000	1.903490000
H	-0.821365000	11.938401000	1.714647000
H	-2.090237000	13.112465000	2.082124000
H	-0.342698000	14.118793000	4.231785000
H	0.858619000	12.830141000	4.145435000
H	0.045672000	13.184532000	5.684591000
H	-5.896774000	12.629236000	6.082518000
H	-5.550517000	14.161905000	6.880268000
H	-4.826185000	12.677366000	7.497490000
H	-3.391314000	14.760519000	3.290701000
H	-4.810022000	15.310590000	4.183418000
H	-4.863938000	13.779124000	3.292591000
H	-1.994648000	14.254919000	7.223228000
H	-3.117725000	15.554033000	6.794479000
H	-1.747906000	15.191208000	5.740632000
H	-0.066407000	14.432334000	9.993249000

65
compound 6

Se	-3.074041000	10.006537000	8.073831000
Al	-3.602286000	10.506266000	5.826554000
C	-2.058912000	11.572941000	8.597978000
N	-2.875722000	11.997620000	5.081245000
C	-5.413498000	9.818831000	4.922009000
C	-5.981763000	9.367064000	6.141761000
C	-4.289103000	8.916484000	4.613652000
C	-5.263924000	8.231272000	6.589598000
C	-4.247340000	7.943192000	5.666680000
C	-2.631903000	12.485322000	9.485640000
C	-0.734436000	11.745723000	8.195740000
Si	-1.424803000	11.848954000	4.128496000
Si	-3.632441000	13.526783000	5.460229000
C	-6.037174000	10.725755000	3.900334000
C	-7.092575000	10.004800000	6.908897000
C	-3.788059000	8.709385000	3.210767000
C	-5.521449000	7.522686000	7.882383000
C	-3.223662000	6.862447000	5.809667000
H	-3.658083000	12.345478000	9.812516000
C	-1.886917000	13.561718000	9.959951000
H	-0.277560000	11.026140000	7.524500000
C	0.004548000	12.826261000	8.668921000
C	-0.682977000	10.137249000	4.419037000
C	-1.755575000	12.064622000	2.283858000
C	-0.109801000	13.119898000	4.576639000
C	-5.105292000	13.196873000	6.593001000
C	-4.258231000	14.397361000	3.909041000
C	-2.507764000	14.735110000	6.360810000
H	-6.720323000	11.447787000	4.355154000
H	-6.613086000	10.138854000	3.171978000
H	-5.282100000	11.292099000	3.345326000
H	-7.852586000	9.259721000	7.171175000
H	-7.584335000	10.800336000	6.343611000
H	-6.726539000	10.434756000	7.851314000
H	-2.897837000	8.075530000	3.181209000
H	-3.539219000	9.650591000	2.713752000
H	-4.561412000	8.214736000	2.607543000
H	-5.617909000	8.228339000	8.715447000
H	-4.711430000	6.830636000	8.128356000
H	-6.451151000	6.940719000	7.835788000
H	-3.689044000	5.870391000	5.767798000
H	-2.695364000	6.938669000	6.767882000
H	-2.473417000	6.912129000	5.016394000
C	-0.568038000	13.736901000	9.552489000
H	-2.342838000	14.265786000	10.650421000
H	1.035700000	12.950898000	8.349515000
H	-0.341960000	10.009351000	5.452441000
H	0.186911000	9.997673000	3.765977000
H	-1.379979000	9.321624000	4.200107000
H	-2.496525000	11.357835000	1.897317000
H	-0.831496000	11.925272000	1.709638000
H	-2.125506000	13.073426000	2.070138000
H	-0.358514000	14.116989000	4.198078000
H	0.844593000	12.828952000	4.120782000
H	0.036429000	13.196259000	5.658792000
H	-5.891036000	12.600149000	6.119174000
H	-5.556639000	14.155016000	6.877840000
H	-4.807544000	12.691883000	7.520088000
H	-3.429799000	14.722381000	3.269389000
H	-4.826535000	15.294735000	4.182443000
H	-4.911380000	13.755073000	3.308732000
H	-1.969859000	14.252486000	7.182260000
H	-3.117525000	15.540554000	6.788709000
H	-1.770783000	15.198539000	5.698490000
H	0.011206000	14.577803000	9.922218000

65
compound 7

Te	-3.202087000	9.988553000	8.286862000
Al	-3.740125000	10.545396000	5.829334000
C	-2.072059000	11.750900000	8.763216000
N	-2.904958000	11.969286000	5.062440000
C	-5.600879000	9.878356000	5.006440000
C	-6.062582000	9.273825000	6.210880000
C	-4.481875000	9.054069000	4.518734000
C	-5.272159000	8.136458000	6.481459000
C	-4.318277000	7.988313000	5.454687000
C	-2.668398000	12.759927000	9.521848000
C	-0.728154000	11.864684000	8.406876000
Si	-1.378799000	11.698002000	4.260653000
Si	-3.606954000	13.545628000	5.333910000
C	-6.358511000	10.824784000	4.117194000
C	-7.120917000	9.806778000	7.120000000
C	-4.037316000	9.069120000	3.084064000
C	-5.395205000	7.257564000	7.688980000
C	-3.267702000	6.926620000	5.406039000
H	-3.711265000	12.674132000	9.813410000
C	-1.927612000	13.872258000	9.914501000
H	-0.249704000	11.076534000	7.833865000
C	0.007237000	12.981856000	8.796137000
C	-0.758757000	9.973477000	4.714519000
C	-1.510192000	11.804432000	2.381744000
C	-0.049601000	12.932945000	4.763854000
C	-5.117568000	13.333569000	6.446155000
C	-4.132455000	14.351688000	3.711963000
C	-2.471512000	14.760487000	6.212040000
H	-7.110569000	11.390803000	4.672514000
H	-6.882533000	10.266689000	3.329780000
H	-5.699715000	11.547141000	3.624010000
H	-7.873771000	9.040199000	7.336213000
H	-7.633608000	10.670155000	6.688813000
H	-6.687333000	10.120423000	8.079782000
H	-3.048284000	8.624412000	2.943343000

H	-4.000694000	10.088493000	2.690316000
H	-4.745737000	8.501837000	2.464949000
H	-5.770455000	7.813046000	8.554381000
H	-4.431737000	6.819557000	7.969403000
H	-6.090301000	6.427066000	7.506411000
H	-3.719208000	5.932183000	5.497453000
H	-2.549891000	7.033817000	6.230748000
H	-2.706409000	6.952951000	4.468546000
C	-0.589338000	13.987360000	9.551218000
H	-2.402181000	14.651840000	10.504067000
H	1.053001000	13.061196000	8.511326000
H	-0.541195000	9.883919000	5.784689000
H	0.166782000	9.759271000	4.166309000
H	-1.470457000	9.179059000	4.462003000
H	-2.057762000	10.961990000	1.948843000
H	-0.507971000	11.811229000	1.935784000
H	-2.015659000	12.725076000	2.069759000
H	-0.193397000	13.907369000	4.285087000
H	0.929267000	12.555552000	4.443250000
H	-0.019834000	13.088416000	5.846487000
H	-5.897320000	12.695473000	6.018129000
H	-5.570011000	14.316183000	6.627024000
H	-4.838724000	12.919983000	7.423481000
H	-3.258151000	14.614489000	3.104981000
H	-4.683844000	15.279803000	3.904786000
H	-4.771295000	13.699879000	3.106504000
H	-1.988253000	14.307181000	7.083138000
H	-3.068769000	15.609233000	6.568359000
H	-1.690468000	15.161487000	5.559679000
H	-0.013391000	14.856494000	9.855315000

77
compound 8

Si	2.899226000	3.852242000	7.230248000
N	4.290427000	4.232675000	6.231664000
C	1.430399000	4.887687000	6.657015000
C	2.388660000	2.044122000	7.155173000
C	3.166079000	4.333784000	9.035822000
Si	5.880379000	3.878002000	6.839362000
Al	3.847799000	4.682354000	4.509701000
H	1.615166000	5.957846000	6.800339000
H	0.548236000	4.622167000	7.251899000
H	1.166976000	4.714928000	5.608510000
H	2.167644000	1.754520000	6.122311000
H	1.483928000	1.880544000	7.753399000
H	3.164551000	1.373829000	7.537782000
H	3.945065000	3.753739000	9.539396000
H	2.230965000	4.164563000	9.583781000
H	3.418999000	5.395737000	9.132315000
C	7.151957000	3.742614000	5.437584000
C	5.949522000	2.206301000	7.715286000
C	6.488246000	5.160584000	8.086972000
O	2.814964000	3.326075000	4.035332000
C	5.141431000	6.455856000	3.995326000
C	3.912433000	6.935324000	4.527380000
C	2.857254000	6.505679000	3.659619000
C	3.444221000	5.781952000	2.577899000
C	4.853781000	5.713503000	2.796829000
H	6.761584000	4.023282000	4.455119000
H	7.498320000	2.706004000	5.357999000
H	8.029333000	4.367406000	5.634773000
H	5.304505000	2.138171000	8.596376000
H	6.978499000	2.019709000	8.047880000
H	5.670401000	1.395732000	7.031849000
H	6.387300000	6.189623000	7.725937000
H	7.547173000	4.991108000	8.317676000
H	5.932064000	5.091964000	9.027326000
C	2.700902000	2.494520000	2.996725000
C	6.490273000	6.833310000	4.519228000
C	3.796456000	7.746189000	5.778778000
C	1.408075000	6.875635000	3.740633000
C	2.701174000	5.259400000	1.392572000
C	5.855130000	5.152392000	1.833795000
H	6.596631000	6.635431000	5.589830000
H	6.653946000	7.908479000	4.370484000

H	7.291307000	6.304950000	3.999194000
H	2.759188000	8.016319000	5.989938000
H	4.367818000	8.678061000	5.688303000
H	4.188014000	7.200488000	6.646291000
H	0.759434000	6.046038000	3.441720000
H	1.195708000	7.712803000	3.063763000
H	1.115705000	7.178839000	4.747805000
H	2.500528000	6.076854000	0.687000000
H	1.736747000	4.820829000	1.667352000
H	3.273092000	4.496748000	0.858796000
H	6.782557000	4.855628000	2.330795000
H	6.111032000	5.902994000	1.075200000
H	5.468927000	4.268952000	1.318742000
H	0.421988000	3.651087000	3.759679000
C	0.254245000	2.799519000	3.109885000
C	-1.035592000	2.425399000	2.773465000
H	-1.875575000	2.999172000	3.156062000
C	-1.264454000	1.319550000	1.954324000
H	-2.276862000	1.026957000	1.693506000
C	-0.173129000	0.579148000	1.494721000
H	-0.337952000	-0.306781000	0.887485000
C	1.120976000	0.944946000	1.822322000
H	1.948184000	0.323401000	1.496312000
C	1.374451000	2.084328000	2.623286000
C	3.924576000	2.043127000	2.366940000
C	4.014800000	1.763988000	0.984979000
H	3.133495000	1.874595000	0.360830000
C	5.224878000	1.411931000	0.408476000
H	5.271148000	1.222769000	-0.660624000
C	6.383546000	1.319108000	1.182388000
H	7.328214000	1.040198000	0.725858000
C	6.313022000	1.595329000	2.546786000
H	7.205934000	1.519944000	3.161114000
C	5.108863000	1.958164000	3.131227000
H	5.054756000	2.132286000	4.203695000

75
compound 9

Si	2.384537000	3.646698000	7.152343000
N	3.892975000	4.079156000	6.359342000
C	0.964005000	4.770231000	6.622140000
C	1.910315000	1.852801000	6.822176000
C	2.442113000	3.864644000	9.028204000
Si	5.383469000	3.794210000	7.217404000
Al	3.761688000	4.445166000	4.563882000
H	1.113457000	5.791599000	6.989160000
H	0.036044000	4.394007000	7.070856000
H	0.813935000	4.813353000	5.540510000
H	1.777811000	1.663376000	5.752349000
H	0.970338000	1.599665000	7.327606000
H	2.680953000	1.167053000	7.191200000
H	3.168956000	3.228805000	9.541449000
H	1.450752000	3.600705000	9.417822000
H	2.637076000	4.904826000	9.310186000
C	6.880987000	3.964346000	6.080801000
C	5.487916000	2.032126000	7.889350000
C	5.669916000	4.976557000	8.661649000
O	2.260903000	3.580598000	3.999054000
C	5.789414000	6.446831000	2.924818000
C	5.111924000	6.643697000	4.117703000
C	3.683585000	6.366930000	3.886933000
C	3.571920000	6.088435000	2.449224000
C	4.839706000	6.104292000	1.902746000
H	6.835572000	4.840159000	5.424790000
H	7.012944000	3.067869000	5.466020000
H	7.780242000	4.066985000	6.699604000
H	4.725098000	1.803544000	8.639883000
H	6.466982000	1.870170000	8.357035000
H	5.382713000	1.305137000	7.075440000
H	5.568725000	6.025980000	8.365610000
H	6.689542000	4.838988000	9.042272000
H	4.983466000	4.799423000	9.493632000
C	2.436537000	2.620798000	3.114850000
C	7.262558000	6.582054000	2.686883000
C	5.629755000	7.252208000	5.381344000

C	2.636315000	7.194315000	4.615682000
C	2.296708000	5.780795000	1.726637000
C	5.206686000	5.742030000	0.498562000
H	7.829170000	6.582556000	3.623157000
H	7.499140000	7.517443000	2.162516000
H	7.650153000	5.768284000	2.060190000
H	5.202756000	6.771270000	6.267450000
H	5.360028000	8.316798000	5.434645000
H	6.719607000	7.191124000	5.457018000
H	1.620608000	6.839903000	4.417614000
H	2.692851000	8.242439000	4.291200000
H	2.782305000	7.172010000	5.700279000
H	2.075245000	6.543672000	0.969320000
H	1.447779000	5.738218000	2.413762000
H	2.336574000	4.813698000	1.206810000
H	5.736401000	4.779033000	0.451570000
H	5.871033000	6.491001000	0.050564000
H	4.320309000	5.658760000	-0.136722000
H	-0.039759000	3.033091000	4.004842000
C	0.005998000	2.248005000	3.259635000
C	-1.127723000	1.565490000	2.859583000
H	-2.093913000	1.814133000	3.289617000
C	-1.014840000	0.558147000	1.903970000
H	-1.879732000	-0.001512000	1.562830000
C	0.251726000	0.283210000	1.393158000
H	0.381321000	-0.498640000	0.644427000
N	1.356587000	0.924425000	1.761786000
C	1.248716000	1.903217000	2.687834000
C	3.790827000	2.411420000	2.683943000
C	4.251098000	1.488541000	1.722453000
H	3.529388000	0.825329000	1.265941000
C	5.585965000	1.464740000	1.385716000
H	5.942285000	0.755965000	0.643929000
C	6.476173000	2.361983000	1.991652000
H	7.529925000	2.382161000	1.737927000
C	5.983233000	3.237408000	2.936825000
H	6.626921000	3.958071000	3.425463000
N	4.690005000	3.264975000	3.297458000

144
compound 10

Si	16.711223000	3.547211000	19.650579000
N	15.121113000	3.723562000	18.942072000
C	16.684520000	3.537758000	21.549894000
C	17.573378000	1.980078000	19.038122000
C	17.950794000	4.934313000	19.341557000
Si	13.887742000	2.819812000	19.793565000
Al	14.751222000	4.803726000	17.452900000
H	16.354414000	4.516187000	21.920014000
H	17.713198000	3.389003000	21.901641000
H	16.063163000	2.777033000	22.025533000
H	16.991386000	1.073819000	19.225988000
H	18.553625000	1.861074000	19.515957000
H	17.742293000	2.051685000	17.957845000
H	18.249096000	5.058277000	18.301042000
H	18.854958000	4.653027000	19.896967000
H	17.620982000	5.903354000	19.729730000
C	12.360431000	2.307730000	18.811167000
C	14.474008000	1.111271000	20.384174000
C	13.239900000	3.779211000	21.292931000
O	15.965842000	4.709938000	16.125442000
O	13.231942000	5.762707000	17.590634000
N	15.764101000	6.537549000	17.814786000
N	13.539905000	3.661115000	16.275566000
H	11.707535000	3.123261000	18.501866000
H	11.774232000	1.676255000	19.491514000
H	12.599844000	1.699418000	17.932984000
H	14.602782000	0.447522000	19.520495000
H	13.679112000	0.680142000	21.006139000
H	15.397197000	1.081755000	20.965418000
H	13.927647000	3.739413000	22.142682000
H	12.271942000	3.382394000	21.622414000
H	13.096019000	4.833703000	21.034842000
C	16.914928000	5.680258000	15.897343000
C	12.071284000	5.438775000	16.924387000
C	16.774859000	6.772221000	16.968082000
C	15.476983000	7.403847000	18.796528000
C	12.293018000	4.131979000	16.148474000
C	13.917788000	2.541222000	15.642914000
C	16.564481000	6.397124000	14.453303000
C	18.312574000	5.055334000	15.898352000
C	11.796443000	6.606788000	15.797069000
C	10.912117000	5.316187000	17.917947000
C	17.564651000	7.913516000	17.104289000
C	16.214840000	8.554896000	18.988901000
H	14.633701000	7.139485000	19.429424000
C	11.354689000	3.455343000	15.369072000
C	13.041759000	1.819579000	14.857112000
H	14.950750000	2.238333000	15.792813000
O	15.619047000	7.370808000	14.684040000
C	15.945884000	5.303546000	13.570340000
C	17.808856000	7.015667000	13.809787000
N	19.367511000	5.867414000	16.023038000
C	18.448093000	3.672422000	15.775983000
O	12.524612000	6.286123000	14.673415000
C	12.344695000	7.915007000	16.386778000
C	10.310926000	6.724333000	15.445135000
N	9.795542000	4.703131000	17.510676000
C	11.051261000	5.828851000	19.207248000
H	18.394309000	8.072164000	16.430848000
C	17.279949000	8.805309000	18.125656000
H	15.963907000	9.235819000	19.795265000
C	11.736396000	2.289687000	14.725389000
H	10.345855000	3.835102000	15.297659000
H	13.372690000	0.912691000	14.362245000
Al	13.920713000	7.261306000	14.085220000
N	14.657449000	5.535321000	13.288103000
C	16.584386000	4.161631000	13.086797000
N	18.682851000	6.199541000	13.211466000
C	17.995427000	8.397103000	13.860529000
C	20.587387000	5.329234000	16.020591000
H	17.557906000	3.059920000	15.701631000
C	19.722365000	3.123263000	15.766665000
N	13.388372000	8.393811000	15.698347000
C	11.868881000	8.584768000	17.513977000
N	9.504878000	7.341722000	16.315382000
C	9.842904000	6.199985000	14.240557000
C	8.782260000	4.591333000	18.370015000
H	11.987514000	6.291583000	19.492826000
C	9.989150000	5.712191000	20.092217000
H	17.889738000	9.695897000	18.251797000
H	11.016210000	1.744756000	14.120952000
N	13.549292000	8.352587000	12.603507000
C	13.949789000	4.665411000	12.553538000
C	15.860882000	3.267233000	12.315045000
H	17.631402000	4.004861000	13.301589000
C	19.771478000	6.731948000	12.655310000
C	19.135633000	8.940153000	13.286186000
H	17.241657000	9.013294000	14.335060000
H	21.419517000	6.024620000	16.130540000
C	20.821485000	3.965076000	15.890245000
H	19.855739000	2.048370000	15.677187000
C	14.007283000	9.517866000	16.086629000
C	12.496774000	9.753932000	17.911070000
H	11.010699000	8.195957000	18.042612000
C	8.209513000	7.446634000	16.017849000
H	10.544369000	5.731882000	13.561014000
C	8.491894000	6.308214000	13.943529000
H	7.892998000	4.080591000	18.000554000
C	8.824364000	5.082071000	19.670075000
H	10.074804000	6.097115000	21.104937000
Si	12.074576000	9.279126000	12.429534000
Si	14.648321000	8.554054000	11.256171000
C	14.516850000	3.514876000	12.042765000
H	12.908383000	4.926020000	12.383858000
H	16.344585000	2.376509000	11.923174000
H	20.454898000	6.033565000	12.172253000
C	20.049577000	8.093861000	12.669367000
H	19.302535000	10.013855000	13.307981000
H	21.835296000	3.576900000	15.897087000
C	13.589090000	10.233882000	17.190330000

H	14.851861000	9.828656000	15.477249000
H	12.134313000	10.293942000	18.781669000
H	7.588323000	7.961496000	16.750763000
C	7.650088000	6.943322000	14.848960000
H	8.102762000	5.912867000	13.008973000
H	7.972414000	4.961855000	20.332159000
C	11.168297000	9.766910000	14.011168000
C	12.352731000	10.999682000	11.672184000
C	10.796618000	8.358951000	11.382618000
C	13.770644000	8.618269000	9.572061000
C	15.710074000	10.105482000	11.455145000
C	15.872260000	7.156445000	10.930232000
H	13.921648000	2.831793000	11.446173000
H	20.950266000	8.477177000	12.199867000
H	14.104431000	11.144198000	17.477781000
H	6.588553000	7.057758000	14.652359000
H	10.732296000	8.942019000	14.574052000
H	10.335128000	10.404448000	13.687958000
H	11.787108000	10.363844000	14.688847000
H	12.880040000	11.637601000	12.392147000
H	11.369963000	11.455318000	11.496653000
H	12.903987000	11.037107000	10.731112000
H	11.123881000	8.212075000	10.349513000
H	9.844658000	8.903578000	11.363797000
H	10.602438000	7.371163000	11.814074000
H	13.340661000	7.635098000	9.344155000
H	14.527719000	8.824428000	8.804835000
H	12.978372000	9.360113000	9.457665000
H	15.115819000	11.020904000	11.519955000
H	16.408531000	10.210552000	10.615921000
H	16.305905000	10.028222000	12.370955000
H	16.613707000	6.996549000	11.712331000
H	16.424507000	7.448025000	10.027346000
H	15.381279000	6.201848000	10.714104000

10 References

[1] A. M. Borys, "An Illustrated Guide to Schlenk Line Techniques" *Organometallics* **2023**, *42*, 182–196.

[2] C. Ganesamoorthy, S. Loerke, C. Gemel, P. Jerabek, M. Winter, G. Frenking, R. A. Fischer, "Reductive Elimination: A Pathway to Low-Valent Aluminium Species" *Chem. Commun.* **2013**, *49*, 2858.

[3] H. Bürger, W. Sawodny, U. Wannagat, "Darstellung und Schwingungsspektren von Silylamiden der Elemente Zink, Cadmium und Quecksilber" *J. Organomet. Chem.* **1965**, *3*, 113–120.

[4] S. Stoll, A. Schweiger, "EasySpin, a Comprehensive Software Package for Spectral Simulation and Analysis in EPR" *J. Magn. Reson.* **2006**, *178*, 42–55.

[5] D. Herle, S. Sommer, F. Dankert, "Functional Al/Cd Heterometallics─From Controlled Al(I) Transfer to Nucleophilic Transfer of Cadmium Ions" *J. Am. Chem. Soc.* **2025**, *147*, 33315–33323.

[6] T. Heurich, V. Nesterov, G. Schnakenburg, Z. Qu, S. Grimme, K. Hazin, D. P. Gates, M. Engeser, R. Streubel, "Strong Evidence of a Phosphanoxyl Complex: Formation, Bonding, and Reactivity of Ligated Phosphorus Analogues of Nitroxides" *Angew. Chem. Int. Ed.* **2016**, *55*, 14439–14443.

[7] R. D. Deslattes, E. G. Kessler, P. Indelicato, L. de Billy, E. Lindroth, J. Anton, "X-Ray Transition Energies: New Approach to a Comprehensive Evaluation" *Rev. Mod. Phys.* **2003**, *75*, 35–99.

[8] W. T. Elam, B. D. Ravel, J. R. Sieber, "A New Atomic Database for X-Ray Spectroscopic Calculations" *Radiat. Phys. Chem.* **2002**, *63*, 121–128.

[9] G. M. Sheldrick, "SHELXT – Integrated Space-Group and Crystal-Structure Determination" *Acta Crystallogr. A* **2015**, *71*, 3–8.

[10] G. M. Sheldrick, "Crystal Structure Refinement with SHELXL" *Acta Crystallogr. C* **2015**, *71*, 3–8.

[11] O. V. Dolomanov, L. J. Bourhis, R. J. Gildea, J. A. K. Howard, H. Puschmann, "OLEX2: A Complete Structure Solution, Refinement and Analysis Program" *J. Appl. Crystallogr.* **2009**, *42*, 339–341.

[12] X-AREA; integrated LANA; XRED32. Stoe & Cie: Darmstadt, Germany, **2020**.

[13] K. Brandenburg, *Diamond Version 4.6.8*, **2022**, Bonn.

[14] F. Neese, "The ORCA Program System" *WIREs Comput. Mol. Sci.* **2012**, *2*, 73–78.

[15] F. Neese, "Software Update: The ORCA Program System—Version 5.0" *WIREs Comput. Mol. Sci.* **2022**, *12*, e1606.

[16] S. Grimme, A. Hansen, S. Ehlert, J.-M. Mewes, "r^{2}SCAN-3c: A 'Swiss Army Knife' Composite Electronic-Structure Method" *J. Chem. Phys.* **2021**, *154*, 064103.

[17] J. Tao, J. P. Perdew, V. N. Staroverov, G. E. Scuseria, "Climbing the Density Functional Ladder: Nonempirical Meta–Generalized Gradient Approximation Designed for Molecules and Solids" *Phys. Rev. Lett.* **2003**, *91*, 146401.

[18] F. Weigend, R. Ahlrichs, "Balanced Basis Sets of Split Valence, Triple Zeta Valence and Quadruple Zeta Valence Quality for H to Rn: Design and Assessment of Accuracy" *Phys. Chem. Chem. Phys.* **2005**, *7*, 3297–3305.

[19] E. Caldeweyher, C. Bannwarth, S. Grimme, “Extension of the D3 Dispersion Coefficient Model” *J. Chem. Phys.* **2017**, *147*, 034112.
[20] R. Izsák, F. Neese, “An Overlap Fitted Chain of Spheres Exchange Method” *J. Chem. Phys.* **2011**, *135*, 144105.
[21] F. Neese, F. Wennmohs, A. Hansen, U. Becker, “Efficient, Approximate and Parallel Hartree–Fock and Hybrid DFT Calculations. A ‘Chain-of-Spheres’ Algorithm for the Hartree–Fock Exchange” *Chem. Phys.* **2009**, *356*, 98–109.
[22] K. A. Peterson, D. Figgen, E. Goll, H. Stoll, M. Dolg, “Systematically Convergent Basis Sets with Relativistic Pseudopotentials. II. Small-Core Pseudopotentials and Correlation Consistent Basis Sets for the Post-d Group 16–18 Elements” *J. Chem. Phys.* **2003**, *119*, 11113–11123.
[23] G. L. Stoychev, A. A. Auer, F. Neese, “Automatic Generation of Auxiliary Basis Sets” *J. Chem. Theory Comput.* **2017**, *13*, 554–562.
[24] A. V. Marenich, C. J. Cramer, D. G. Truhlar, “Universal Solvation Model Based on Solute Electron Density and on a Continuum Model of the Solvent Defined by the Bulk Dielectric Constant and Atomic Surface Tensions” *J. Phys. Chem. B* **2009**, *113*, 6378–6396.
[25] G. Knizia, IBOView – A program for chemical analysis; see http://www.iboview.org/.
[26] G. Knizia, J. E. M. N. Klein, “Electron Flow in Reaction Mechanisms—Revealed from First Principles” *Angew. Chem. Int. Ed.* **2015**, *54*, 5518–5522.
[27] *Chemcraft* – graphical software for visualization of quantum chemistry computations. https://chemcraftprog.com.
[28] A. D. Becke, “Density-Functional Exchange-Energy Approximation with Correct Asymptotic Behavior” *Phys. Rev. A* **1988**, *38*, 3098–3100.
[29] T. Lu, F. Chen, “Multiwfn: A Multifunctional Wavefunction Analyzer” *J. Comput. Chem.* **2012**, *33*, 580–592.
